# Supplementary material for: Photocatalytic phosphine-mediated water activation for radical hydrogenation
Source: Nature. 2023 Jun 28;619(7970):506–13. doi: 10.1038/s41586-023-06141-1 (PMC10356606; doi:10.1038/s41586-023-06141-1)
Supplement: Supplementary file 1 — Supplementary Information [file 41586_2023_6141_MOESM1_ESM.pdf]

nature portfolio

https://doi.org/10.1038/s41586-023-06141-1

## Supplementary information

# Photocatalytic phosphine-mediated water activation for radical hydrogenation

In the format provided by the
authors and unedited

# Photocatalytic phosphine-mediated water activation for radical hydrogenation

Jingjing Zhang, Christian Mück-Lichtenfeld and Armido Studer

Organisch-Chemisches Institut, Westfälische Wilhelms-Universität, 48149 Münster, Germany

Email: studer@uni-muenster.de

## Supplementary Information

# Content


1. General information ........ 3

2. Reaction optimization ........ 5

3. The synthesis of starting materials ........ 10

4. Synthesis and characterization of products ........ 19

4.1 Synthesis and characterization of products **2** ........ 19

4.2 Synthesis and characterization of products **4** ........ 28

4.3 Synthesis and characterization of products **6** ........ 33

5. Radical cyclization and ring-opening experiments ........ 45

5.1 Radical cyclization experiments ........ 45

5.2 Cycle-opening experiment ........ 47

6. Deuteration experiments with $CD_3CN$ and $D_2O$ as hydrogen source ........ 49

7. Detection of hydrogen gas $H_2$ ........ 55

8. Gram-scale experiments and recycle of phosphine oxides ........ 58

9. Stern-Volmer quenching experiment and quantum yield measurment ........ 60

10. Chemoselective hydrogenation experiments ........ 63

11. Kinetic experiments ........ 67

11.1 The reaction order of alkenes in our conditions ........ 67

11.2 The reaction order of the HAT catalyst in our conditions ........ 68

11.3 The KIE measurement ........ 69

12. DFT calculations ........ 70

12.1 Methods ........ 70

12.2 DFT calculated energies ........ 71

12.3 Optimized molecular structures ........ 73

12.4 DFT optimized (PBE0-D3/def2-TZVP) cartesian coordinates ........ 87

13. NMR spectra ........ 140

14. References ........ 208

## 1. General information

**Chemicals:** All reactions involving air- or moisture-sensitive reagents or intermediates were carried out in pre-heated glassware using standard Schlenk techniques at ambient room temperature, unless otherwise specified. All commercially available reagents were purchased from Sigma-Aldrich, Alfa Aesar, TCI Chemicals, Acros Organics or ABCR in the highest purity grade and used without further purification. The following solvents (ACROS ExtraDry solvents with ACROSeal® cap) were purchased from ACROS Organics, stored under 3 or 4 Å activated molecular sieves and collected under positive argon pressure: acetonitrile ($CH_3CN$), dimethylformamide (DMF), dimethylacetamide (DMA), chloroform ($CHCl_3$) and methanol ($CH_3OH$). Anhydrous tetrahydrofuran (THF) was refluxed over sodium and freshly distilled from potassium prior to use. Thin layer chromatography (TLC) was performed on Merck silica gel 60 F-254 plates and visualized by fluorescence quenching under UV light. Column chromatography was performed on Merck or Fluka silica gel 60 (40-63 μm).

**NMR Spectroscopy:** $^{1}$H-NMR, $^{13}$C-NMR and $^{19}$F-NMR spectra were recorded using a Bruker DPX 300, Bruker AV 300, Bruker AV 400, Agilent DD2 500 or Agilent DD2 600 spectrometer at 299 K or 300 K. $^{1}$H-NMR chemical shifts are given relative to TMS and residual monoprotic solvent peaks were used as an internal reference for $^{1}$H-NMR ($CDCl_3$: $\delta$ = 7.26 ppm; DMSO: $\delta$ = 2.50 ppm) and $^{13}$C-NMR spectra ($CDCl_3$: $\delta$ = 77.16 ppm; DMSO: $\delta$ = 39.52 ppm). $^{31}$P-NMR spectra are referenced according to the proton signal as the primary reference for the unified chemical shift scale. Coupling constants ($J$) are quoted to the nearest 0.1 Hz. The following abbreviations (or combinations thereof) were used to describe $^{1}$H-, $^{13}$C and $^{31}$P-NMR multiplicities: s = singlet, broad singlet = brs, d = doublet, t = triplet, q = quartet, hept = heptet, m = multiplet.

**Mass Spectrometry:** High-resolution (HRMS) ESI (m/z) spectra were measured on a Bruker MicroTof or ThermoFisher Scientific LTQ XL Orbitrap. High-resolution APCI (m/z) spectra were measured on a Thermo-Fisher Scientific Orbitrap LTQ XL. High-resolution EI (m/z) spectra were measured on a Thermo-Fischer Scientific Exactive GC Orbitrap GC-MS System. MassLinx 4.0 of Water-Micromass was used for data analysis.
**GC-FID:** GC-FID was conducted on an Agilent GC 6890 equipped with a flame ionization detector (FID) and an Agilent HP-5, Methyl Siloxan (Model No: 19091Z-413) column using $H_2$ as carrier gas with a flow rate of 1.5 mL min$^{-1}$. The method used starts with the injection temperature $T_0$, the column is heated to temperature $T_1$ (ramp) and this temperature is held for an additional time $t$ ($T_0$ = 50 °C, $T_1$ = 300 °C, ramp = 10 °C min$^{-1}$, $t$ = 15 min).

**Light Sources:** Photochemical reactions were performed with one LED light (5 W, 455 nm) as light source. The reaction temperature was kept at 20 °C using a circulating water system.

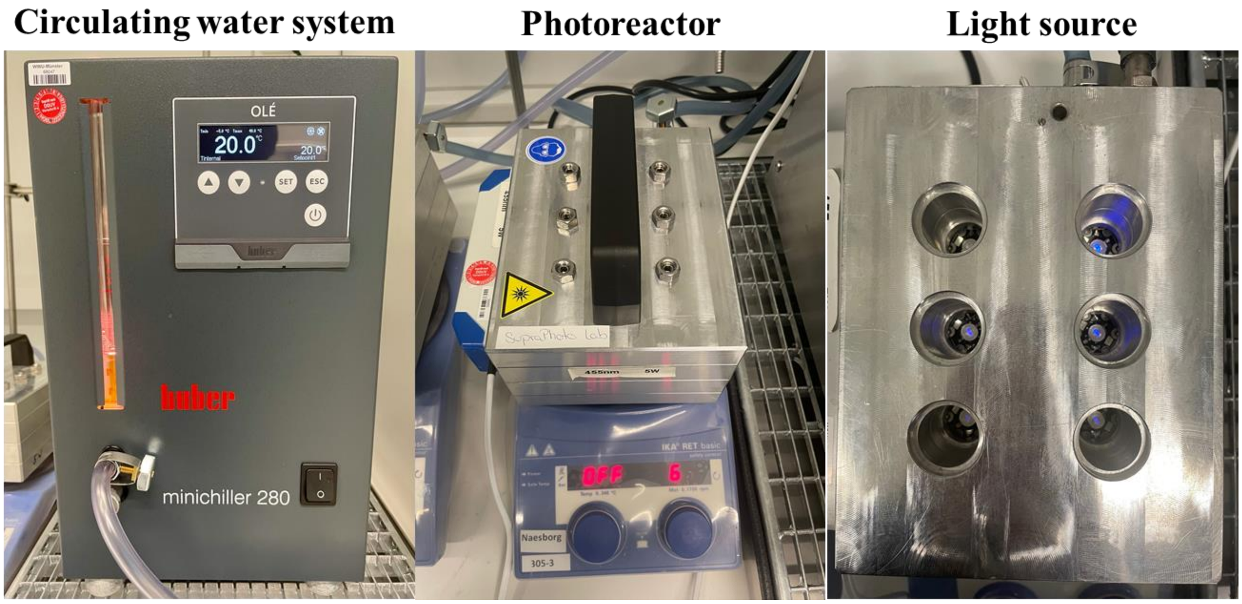


Figure S1. Photoreactors used in this work (455 nm, 5 W or 445 nm, 10 W).

## 2. Reaction optimization

Investigation of commonly used phosphines for reduction of the alkene **1a**:

To an oven dried Schlenk tube with a magnetic stirring bar, the phosphine (0.5 mmol, 2.5 equiv.), **PC1** (0.004 mmol, 2 mol%), deionized water (0.15 mL) and acetonitrile (4.0 mL) were added under argon atmosphere using standard Schlenk techniques at ambient temperature. Then, the alkene **1a** (0.2 mmol, 1.0 equiv.) and the HAT catalyst **HAT1** (0.04 mmol, 0.2 equiv.) were added under argon atmosphere. The tube was sealed and placed in the photoreactor, and then irradiated with a 5 W 455 nm LED at 20 $^oC$ using the standard set-up. After 72 hours, the irradiation was stopped, then the crude yield of **2a** was determined by GC with tetradecane as the internal standard.

Table S1. Investigation of commonly used phosphines for reduction of the alkene **1a**.

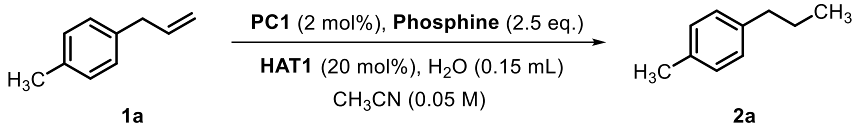


**1a** **2a**

| Phosphine | Yield (**2a**) |
|---|---|
| **P1** | 92% |
| **P2** | 9% |
| **P3** | 6% |
| **P4** | 50% |
| **P5** | 7% |
| **P6** | < 5% |
| **P7** | < 5% |

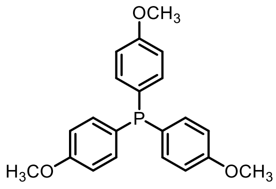


**P1**

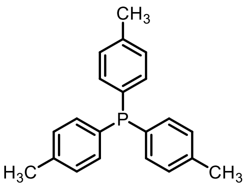


**P2**

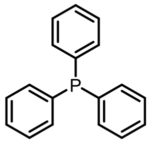


**P3**

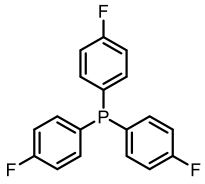


**P4**

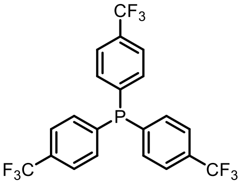


**P5**

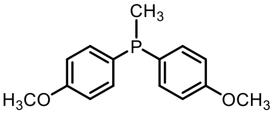


**P6**

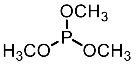


**P7**

Investigation of commonly used photocatalysts for reduction of the alkene **1a**:

To an oven dried Schlenk tube with a magnetic stirring bar, **P1** (0.5 mmol, 2.5 equiv.), the photocatalyst (0.004 mmol, 2 mol%), deionized water (0.15 mL) and acetonitrile (4.0 mL) were added under argon atmosphere using standard Schlenk techniques at ambient temperature. Then, the alkene **1a** (0.2 mmol, 1.0 equiv.) and the HAT catalyst **HAT1** (0.04 mmol, 0.2 equiv.) were added under argon atmosphere. The tube was sealed and placed in the photoreactor, and then irradiated with a 5 W 455 nm LED at 20 $^{o}$C using the standard set-up. After 72 hours, the irradiation was stopped, then the crude yield of **2a** was determined by GC with tetradecane as the internal standard.

Table S2. Investigation of commonly used photocatalysts for reduction of the alkene **1a**.

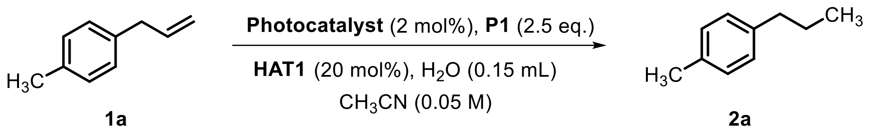


| Photocatalyst | Yield (**2a**) |
|---|---|
| **PC1** | 92% |
| **PC2** | 32% |
| **PC3** | < 5% |
| **PC4** | 0 |
| **PC5** | 0 |

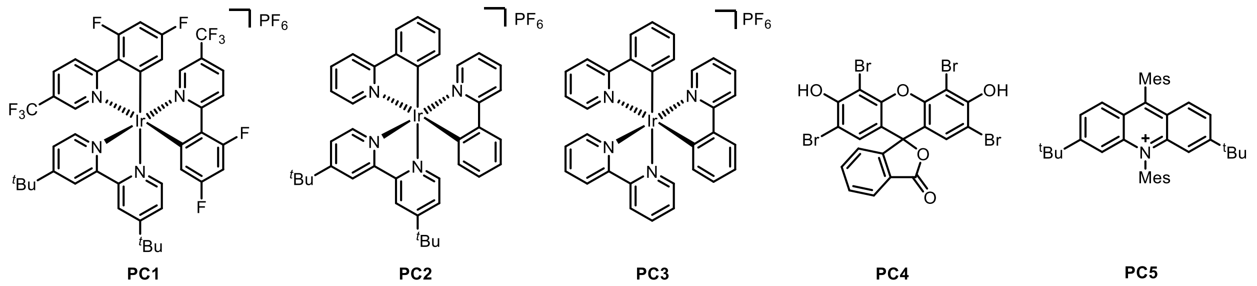

Investigation of commonly used HAT catalysts for reduction of the alkene **1a**:

To an oven dried Schlenk tube with a magnetic stirring bar, **P1** (0.5 mmol, 2.5 equiv.), **PC1** (0.004 mmol, 2 mol%), deionized water (0.15 mL) and acetonitrile (4.0 mL) were added under argon atmosphere using standard Schlenk techniques at ambient temperature. Then, the alkene **1a** (0.2 mmol, 1.0 equiv.) and the HAT catalyst (0.04 mmol, 0.2 equiv.) were added under argon atmosphere. The tube was sealed and placed in the photoreactor, and then irradiated with a 5 W 455 nm LED at 20 °C using the standard set-up. After 72 hours, the irradiation was stopped, then the crude yield of **2a** was determined by GC with tetradecane as the internal standard.

Table S3. Investigation of commonly used HAT catalysts for reduction of the alkene **1a**.

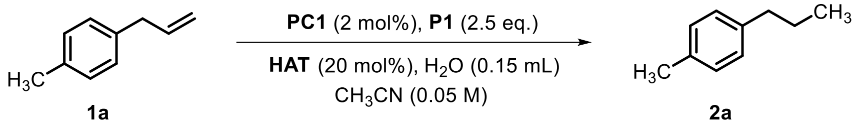


| Photocatalyst | Yield (**2a**) |
|---|---|
| **HAT1** | 92% |
| **HAT2** | 0 |
| **HAT3** | 0 |
| **HAT4** | 0 |

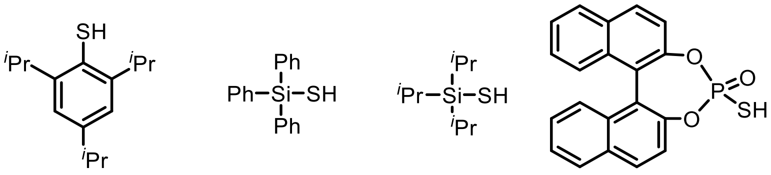


**HAT1** **HAT2** **HAT3** **HAT4**

Investigation of commonly used solvents for reduction of the alkene **1a**:

To an oven dried Schlenk tube with a magnetic stirring bar, **P1** (0.5 mmol, 2.5 equiv.), **PC1** (0.004 mmol, 2 mol%), deionized water (0.15 mL) and the solvent (4.0 mL) were added under argon atmosphere using standard Schlenk techniques at ambient temperature. Then, the alkene **1a** (0.2 mmol, 1.0 equiv.) and the HAT catalyst **HAT1** (0.04 mmol, 0.2 equiv.) were added under argon atmosphere. The tube was sealed and placed in the photoreactor, and then irradiated with a 5 W 455 nm LED at 20 ºC using the standard set-up. After 72 hours, the irradiation was stopped, then the crude yield of **2a** was determined by GC with tetradecane as the internal standard.

Table S4. Investigation of commonly used solvents for reduction of the alkene **1a**.

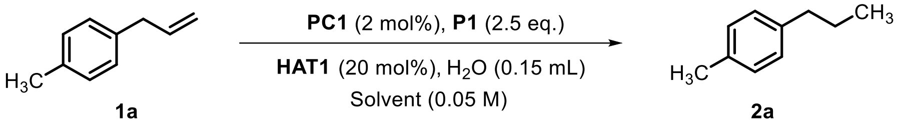


| Photocatalyst | Yield (**2a**) |
|---|---|
| **$CH_3CN$** | 92% |
| **DMF** | 62% |
| **DMSO** | 60% |
| **THF** | 30% |
| **Toluene** | 0 |
| **EtOAc** | 0 |

Investigation of the water amount for reduction of the alkene **1a**:

To an oven dried Schlenk tube with a magnetic stirring bar, **P1** (0.5 mmol, 2.5 equiv.), **PC1** (0.004 mmol, 2 mol%), deionized water (X mL) and acetonitrile (4.0 mL) were added under argon atmosphere using standard Schlenk techniques at ambient temperature. Then, the alkene **1a** (0.2 mmol, 1.0 equiv.) and the HAT catalyst **HAT1** (0.04 mmol, 0.2 equiv.) were added under argon atmosphere. The tube was sealed and placed in the photoreactor, and then irradiated with a 5 W 455 nm LED at 20 ºC using the standard set-up. After 72 hours, the irradiation was stopped, then the crude yield of **2a** was determined by GC with tetradecane as the internal standard.

Table S5. Investigation of the water amount for reduction of the alkene **1a**.

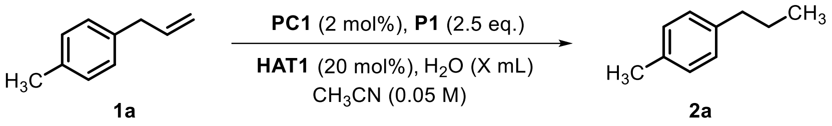


| Water | Yield (**2a**) |
|---|---|
| **0.4 mL** | 42% |
| **0.3 mL** | 62% |
| **0.2 mL** | 80% |
| **0.15 mL** | 92% |
| **0.1 mL** | 78% |

## 3. The synthesis of starting materials

Most substrates used in this work are commercially available unless otherwise specified. Substrates **5q**,[1] **5s**[2] and **5u**[3] were synthesized through the reported methods. Substrates **1g**, **1h**, **1i**, **1l**, **1n**, **1q**, **1t**, **1w**, **1x**, **1y**, **1z**, **3j**, **5h**, **5r**, **5t** and **15** were synthesized using the following procedures:

The synthesis of **1g**:

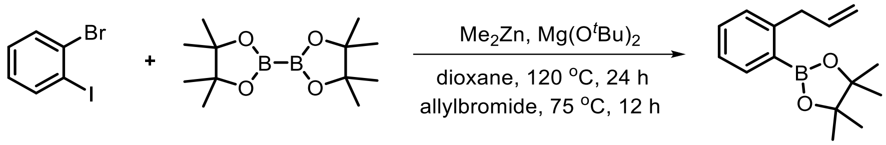


Bis(pinacolato)diboron (254 mg, 1.00 mmol) and magnesium *tert*-butoxide (171 mg, 1.00 mmol) were charged in a dried Schlenk tube under argon atmosphere, and dissolved in dry 1,4-dioxane (2 mL). To the mixture dimethylzinc (1.5 mL, 1.50 mmol; 1.0 M hexane solution) was added at 0 ºC, and the solution was stirred for 30 min at room temperature. To the mixture the 2-bromoiodobenzene (142 mg, 0.50 mmol) was added at room temperature. The mixture was stirred for 24 h at 120 ºC. After cooling to room temperature, to the mixture was added allyl bromide (3.0 equiv.) at the room temperature, and the solution was allowed to warm to 75 ºC, and stirred for 12 h. After cooling again to room temperature, the reaction was quenched by aqueous $NH_4Cl$ (10 mL), and extracted with EtOAc (3 × 15 mL). The combined EtOAc layers were dried over $MgSO_4$, and the solvent was removed under reduced pressure. Purification of the resulting crude residue via silica gel flash chromatography (pentane/EtOAc 10/1).

**$^1$H NMR** (300 MHz, $CDCl_3$) δ 7.90 – 7.79 (m, 1H), 7.48 – 7.35 (m, 1H), 7.31 – 7.13 (m, 2H), 6.20 – 5.87 (m, 1H), 5.15 – 4.92 (m, 2H), 3.76 (d, *J* = 6.6 Hz, 2H), 1.43 – 1.34 (m, 12H).

**$^{13}$C NMR** (76 MHz, $CDCl_3$) δ 146.8, 139.2, 136.1, 131.1, 129.2, 125.4, 114.8, 83.5, 39.8, 24.9.

The NMR data were in agreement with those reported in the literature.[4]

The synthesis of **1h**:

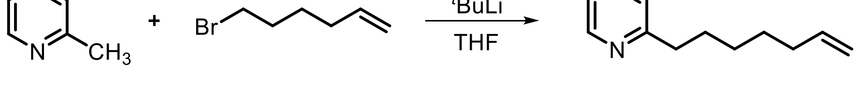


A round bottom flask equipped with a magnetic stir bar was flame dried and back filled with argon. THF (25 mL) and 2-methylpyridine (0.49 mL, 5.0 mmol, 1.0 equiv.) were added. The reaction mixture was cooled to -78 °C and *tert*-butyllithium (3.2 mL, 1.7 M in pentane, 5.5 mmol, 1.1 equiv.) was added dropwise. After completing addition, the reaction mixture was left to stir for 20 minutes, then 6-bromo-1-hexene (0.67 mL, 5.0 mmol, 1.0 equiv.) was added dropwise. The reaction mixture was allowed to warm to room temperature then

left to stir for 1 h at room temperature. The reaction mixture was cooled to 0 °C and quenched with aqueous $NH_4Cl$ (10 mL). Brine was added, the layers were separated, and the aqueous layer was extracted with EtOAc (3 × 20 mL). The combined organics were dried over $MgSO_4$, filtered, and concentrated under reduced pressure to afford the crude residue. Purification of the resulting crude residue via silica gel flash chromatography (pentane/EtOAc 10/1).

**$^1$H NMR** (300 MHz, $CDCl_3$) δ 8.51 (dt, *J* = 4.9, 1.4 Hz, 1H), 7.56 (tt, *J* = 7.7, 1.6 Hz, 1H), 7.21 – 6.98 (m, 2H), 5.79 (ddt, *J* = 16.9, 10.2, 6.7 Hz, 1H), 5.22 – 4.65 (m, 2H), 2.93 – 2.60 (m, 2H), 2.04 (qd, *J* = 7.5, 3.3 Hz, 2H), 1.87 – 1.60 (m, 2H), 1.56 – 1.30 (m, 4H).

**$^{13}$C NMR** (76 MHz, $CDCl_3$) δ 162.4, 149.2, 139.0, 136.2, 122.7, 120.9, 114.3, 38.4, 33.7, 29.8, 28.9, 28.8.

The NMR data were in agreement with those reported in the literature.[5]

The synthesis of **1i**:

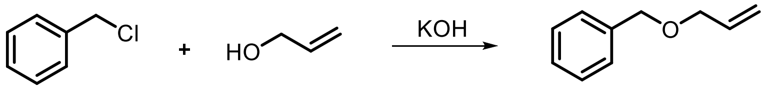


KOH (850 mg, 12.8 mmol) was suspended in allyl alcohol (2.2 mL, 32.4 mmol) and the mixture was stirred until all KOH had dissolved. The mixture was cooled with a water bath around 10 °C and benzyl chloride (0.74 mL, 12.8 mmol) was added slowly. The water bath was removed and the reaction mixture heated to 60 °C for 3 h. The reaction mixture was filtered and purification of the resulting crude residue via silica gel flash chromatography (pentane).

**$^1$H NMR** (300 MHz, $CDCl_3$) δ 7.48 – 7.25 (m, 5H), 6.12 – 5.86 (m, 1H), 5.43 – 5.29 (m, 1H), 5.25 (dd, *J* = 10.4, 1.5 Hz, 1H), 4.57 (d, *J* = 1.7 Hz, 2H), 4.17 – 3.97 (m, 2H).

**$^{13}$C NMR** (76 MHz, $CDCl_3$) δ 138.3, 134.8, 128.4, 127.8, 127.6, 117.2, 72.1, 71.2.

The NMR data were in agreement with those reported in the literature.[6]

The synthesis of **1l**:

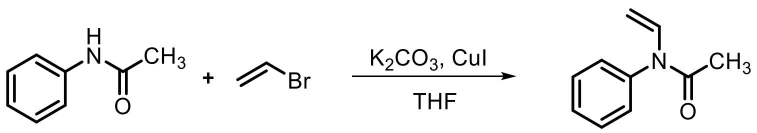


To an oven-dried screw-cap vial fitted with a magnetic stirring bar amide (1.0 equiv.), vinyl bromide (1.0 M solution in THF, 2.0 equiv.), CuI (0.050 equiv.), *N,N'*-dimethylethylenediamine (0.10 equiv.), and $K_2CO_3$ (2.0 equiv.) were added. The vial was tightly sealed using Teflon tape and heated to 80 °C while stirring overnight. Upon completion of the reaction, the mixture was filtered through a pad of celite, rinsing with EtOAc, and

solvent was removed in vacuo to afford the crude product. Purification of the resulting crude residue via silica gel flash chromatography (pentane/EtOAc 3/1).

**$^{1}$H NMR** (300 MHz, $CDCl_3$) δ 7.71 (dd, *J* = 15.9, 9.2 Hz, 1H), 7.49 (ddt, *J* = 12.9, 8.3, 4.3 Hz, 3H), 7.19 (dd, *J* = 7.1, 1.8 Hz, 2H), 4.38 (d, *J* = 9.0 Hz, 1H), 3.86 (d, *J* = 15.9 Hz, 1H), 1.87 (s, 3H).

**$^{13}$C NMR** (76 MHz, $CDCl_3$) δ 168.8, 139.2, 133.7, 130.0, 128.9, 128.7, 96.3, 23.3.

The NMR data were in agreement with those reported in the literature.[7]

The synthesis of **1n**:

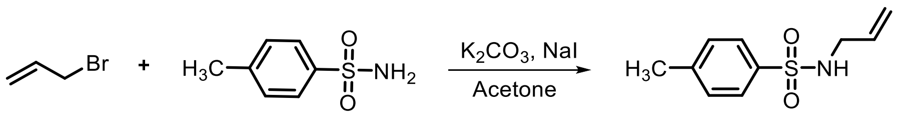


p-Toluenesulfonamide (1.5 equiv.), $K_2CO_3$ (2.0 equiv.) and NaI (10 mol%) were dissolved in reagent grade acetone (1.0 M). The allyl bromide (1.0 equiv.) was added and the reaction mixture was heated to reflux for 12 h. The reaction mixture was cooled, then poured onto $H_2O$ and extracted with EtOAc (3 × 20 mL). The combined organic phases were dried over anhydrous $MgSO_4$, filtered and concentrated in vacuo. Purification of the resulting crude residue via silica gel flash chromatography (pentane/EtOAc 2/1).

**$^{1}$H NMR** (300 MHz, $CDCl_3$) δ 7.78 (d, *J* = 8.1 Hz, 2H), 7.32 (d, *J* = 7.9 Hz, 2H), 5.85 – 5.57 (m, 1H), 5.23 – 5.05 (m, 2H), 4.93 (t, *J* = 6.3 Hz, 1H), 3.58 (tt, *J* = 6.2, 1.5 Hz, 2H), 2.44 (s, 3H).

**$^{13}$C NMR** (76 MHz, $CDCl_3$) δ 143.5, 136.9, 133.0, 129.7, 127.2, 117.6, 45.8, 21.6.

The NMR data were in agreement with those reported in the literature.[8]

The synthesis of **1q**:

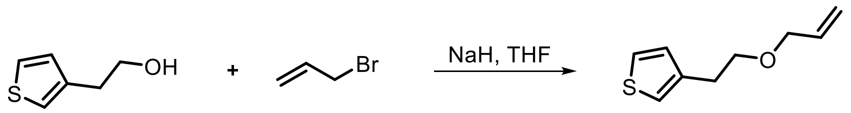


A round-bottomed flask (250 mL) was equipped with a magnetic stirring bar, and flame dried under argon. The 2-(thiophen-2-yl)ethanol (846 mg, 6.6 mmol) was weighed in the flask and dissolved in dry THF (70 mL). Sodium hydride (60%) (13.2 mmol, 2 equiv.) was added to the flask. The resulting dispersion was heated to 50 °C using a silicone oil bath for one hour. The mixture was cooled to 0 °C (ice/water bath) and allyl bromide (13.2 mmol, 2 equiv.) was added through a syringe needle. The new mixture was stirred at 0 °C for five minutes, then warmed to room temperature and stirred overnight. The reaction was quenched by careful addition of a saturated ammonium chloride solution (50 mL) and transferred to a separatory funnel (250 mL) with EtOAc (50 mL). The biphasic mixture was shaken, separated and the aqueous layer extracted twice with EtOAc (2 × 50 mL). The combined organic layers were washed with water (50 mL) and brine (50 mL), dried over $MgSO_4$ and

the solvent removed in vacuo, to give a dry residue. Purification was achieved employing flash column chromatography on silica gel (eluted with pentane/EtOAc 9:1) to afford the title product as yellow oil.

**$^{1}$H NMR** (300 MHz, $CDCl_3$) δ 7.33 – 7.22 (m, 1H), 7.09 – 7.04 (m, 1H), 7.02 (dd, *J* = 4.9, 1.4 Hz, 1H), 6.08 – 5.84 (m, 1H), 5.30 (ddd, *J* = 17.2, 2.4, 1.2 Hz, 1H), 5.21 (dt, *J* = 10.9, 1.7 Hz, 1H), 4.04 (dd, *J* = 5.6, 1.4 Hz, 2H), 3.69 (t, *J* = 7.1 Hz, 2H), 2.97 (t, *J* = 7.1 Hz, 2H).

**$^{13}$C NMR** (76 MHz, $CDCl_3$) δ 139.2, 134.8, 128.5, 125.3, 121.2, 117.0, 71.9, 70.5, 30.8.

The NMR data were in agreement with those reported in the literature.[9]

The synthesis of **1t**:

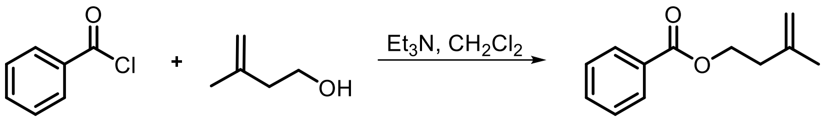


Into a 100-mL round-bottomed flask, equipped with a dropping funnel and charged with argon, 20 mL of dichloromethane, 3-buten-1-ol (2.0 g, 28 mmol) and triethylamine (5.7 g, 56 mmol) were added. To this solution cooled in an ice-water bath, benzoyl chloride (3.6 g, 26 mmol) was added dropwise over 10 minutes. Reaction mixture was stirred for an additional 50 minutes at 0 °C and at room temperature for 3 h. The mixture was poured into 1 M HCl (50 mL), and extracted with diethyl ether (3 × 30 mL). The separated organic layer was washed with aqueous $NaHCO_3$ (50 mL), and dried over anhydrous $MgSO_4$. After evaporation of the solvent under reduced pressure, components of the residue were separated by column chromatography on silica gel (pentane/EtOAc 20/1).

**$^{1}$H NMR** (300 MHz, $CDCl_3$) δ 7.50 – 7.24 (m, 5H), 5.06 (dd, *J* = 2.2, 1.1 Hz, 1H), 5.01 – 4.89 (m, 1H), 4.54 (s, 2H), 3.98 (s, 2H), 1.82 (d, *J* = 1.3 Hz, 3H).

**$^{13}$C NMR** (76 MHz, $CDCl_3$) δ 142.2, 138.5, 128.4, 127.7, 127.6, 112.4, 74.2, 71.8, 19.6.

The NMR data were in agreement with those reported in the literature.[10]

The synthesis of **1w**:

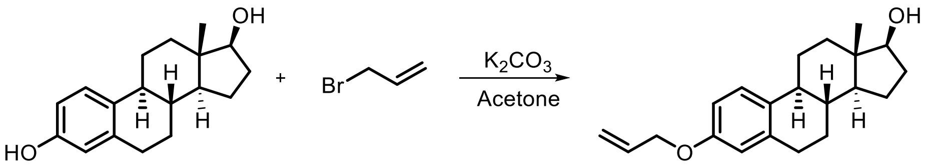


To a flame dried Schlenk flask, *β*-estradiol (136.2 mg, 0.5 mmol, 1.0 equiv.), 3-bromopropene (86 µL, 1.0 mmol, 2.0 equiv.), $K_2CO_3$ (207 mg, 1.5 mmol, 3.0 equiv.) and acetone (7.5 mL) were added. The resulting mixture was stirred at 50 ºC for 40 h. After complete consumption of the starting phenol, the reaction mixture was concentrated and the resulting residue was extracted with $CH_2Cl_2$ for three times. The combined organic layer

was dried over $MgSO_4$, concentrated under vacuum. The residue was separated by column chromatography on silica gel (pentane/EtOAc 6/1).

**$^1$H NMR** (300 MHz, $CDCl_3$) δ 7.31 – 7.18 (m, 1H), 6.75 (dd, *J* = 8.6, 2.8 Hz, 1H), 6.68 (d, *J* = 2.7 Hz, 1H), 6.08 (ddt, *J* = 17.4, 10.5, 5.3 Hz, 1H), 5.50 – 5.37 (m, 1H), 5.30 (dq, *J* = 10.5, 1.5 Hz, 1H), 4.65 – 4.42 (m, 2H), 3.74 (d, *J* = 11.0 Hz, 1H), 2.97 – 2.74 (m, 2H), 2.33 (dt, *J* = 12.5, 3.7 Hz, 1H), 2.29 – 2.05 (m, 2H), 2.04 – 1.82 (m, 2H), 1.72 (tdd, *J* = 10.0, 7.3, 4.0 Hz, 1H), 1.61 – 1.13 (m, 9H), 0.81 (s, 3H).

**$^{13}$C NMR** (76 MHz, $CDCl_3$) δ 156.5, 138.0, 133.6, 132.8, 126.3, 117.5, 114.8, 112.2, 81.9, 68.8, 50.1, 44.0, 43.3, 38.8, 36.7, 30.6, 29.8, 27.3, 26.3, 23.2, 11.1.

The NMR data were in agreement with those reported in the literature.[11]

The synthesis of **1x**:

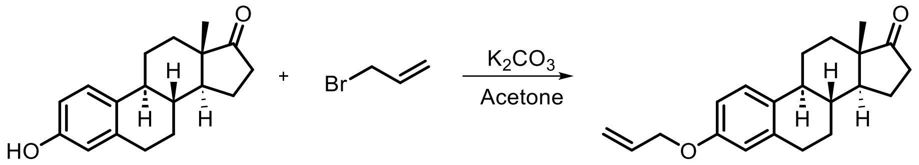


To a flame dried Schlenk flask, estrone (135.2 mg, 0.5 mmol, 1.0 equiv.), 3-bromopropene (86 μL, 1.0 mmol, 2.0 equiv.), $K_2CO_3$ (207 mg, 1.5 mmol, 3.0 equiv.) and acetone (7.5 mL) were added. The resulting mixture was stirred at 50 °C for 40 h. After complete consumption of the starting phenol, the reaction mixture was concentrated and the resulting residue was extracted with $CH_2Cl_2$ for three times. The combined organic layer was dried over $MgSO_4$, concentrated under vacuum. The residue was separated by column chromatography on silica gel (pentane/EtOAc 5/1).

**$^1$H NMR** (300 MHz, $CDCl_3$) δ 7.20 (dd, *J* = 8.7, 1.1 Hz, 1H), 6.74 (dd, *J* = 8.6, 2.8 Hz, 1H), 6.67 (s, 1H), 6.06 (ddt, *J* = 17.2, 10.5, 5.3 Hz, 1H), 5.41 (dq, *J* = 17.2, 1.6 Hz, 1H), 5.28 (dq, *J* = 10.4, 1.4 Hz, 1H), 4.51 (dt, *J* = 5.1, 1.4 Hz, 2H), 2.90 (dt, *J* = 8.5, 3.8 Hz, 2H), 2.57 – 2.45 (m, 1H), 2.40 (ddt, *J* = 12.0, 4.2, 2.1 Hz, 1H), 2.32 – 2.20 (m, 1H), 2.18 – 1.89 (m, 4H), 1.69 – 1.35 (m, 6H), 0.91 (s, 3H).

**$^{13}$C NMR** (76 MHz, $CDCl_3$) δ 221.0, 156.6, 137.8, 133.5, 132.2, 126.3, 117.5, 114.8, 112.3, 68.8, 50.4, 48.0, 44.0, 38.4, 35.9, 31.6, 29.7, 26.6, 25.9, 21.6, 13.9.

The NMR data were in agreement with those reported in the literature.[12]

The synthesis of **1y**:

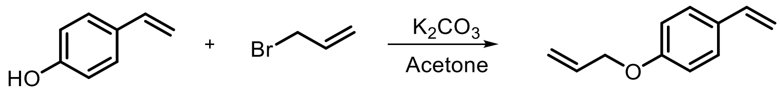


To a flame dried Schlenk flask, 4-vinylphenol (600 mg, 5.0 mmol, 1.0 equiv.), 3-bromopropene (860 μL, 10.0 mmol, 2.0 equiv.), $K_2CO_3$ (15.0 mmol, 3.0 equiv.) and acetone (50 mL) were added. The resulting mixture was

stirred at 50 ºC for 40 h. After complete consumption of the starting phenol, the reaction mixture was concentrated and the resulting residue was extracted with $CH_2Cl_2$ for three times. The combined organic layer was dried over $MgSO_4$, concentrated under vacuum. The residue was separated by column chromatography on silica gel (pentane/EtOAc 20/1).

**$^1$H NMR** (300 MHz, $CDCl_3$) δ 7.26 (d, *J* = 8.8 Hz, 2H), 6.79 (d, *J* = 8.7 Hz, 2H), 6.58 (dd, *J* = 17.6, 10.9 Hz, 1H), 5.98 (ddt, *J* = 17.2, 10.5, 5.3 Hz, 1H), 5.53 (dd, *J* = 17.6, 1.0 Hz, 1H), 5.33 (dd, *J* = 17.3, 1.6 Hz, 1H), 5.21 (dd, *J* = 10.5, 1.4 Hz, 1H), 5.05 (dd, *J* = 10.9, 1.0 Hz, 1H), 4.46 (dt, *J* = 5.3, 1.5 Hz, 2H).

The NMR data were in agreement with those reported in the literature.[13]

The synthesis of **1z**:

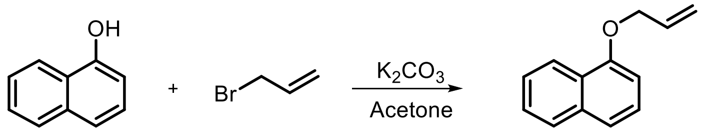


To a flame dried Schlenk flask, naphthalen-1-ol (720 mg, 5.0 mmol, 1.0 equiv.), 3-bromopropene (860 µL, 10.0 mmol, 2.0 equiv.), $K_2CO_3$ (15.0 mmol, 3.0 equiv.) and acetone (50 mL) were added. The resulting mixture was stirred at 50 ºC for 40 h. After complete consumption of the starting phenol, the reaction mixture was concentrated and the resulting residue was extracted with $CH_2Cl_2$ for three times. The combined organic layer was dried over $MgSO_4$, concentrated under vacuum. The residue was separated by column chromatography on silica gel (pentane/EtOAc 20/1).

**$^1$H NMR** (300 MHz, $CDCl_3$) δ 8.45 – 8.32 (m, 1H), 7.93 – 7.78 (m, 1H), 7.60 – 7.35 (m, 4H), 6.86 (dd, *J* = 7.5, 1.2 Hz, 1H), 6.23 (ddt, *J* = 17.3, 10.4, 5.1 Hz, 1H), 5.58 (dd, *J* = 17.3, 1.6 Hz, 1H), 5.39 (dd, *J* = 10.5, 1.5 Hz, 1H), 4.77 (dt, *J* = 5.1, 1.6 Hz, 2H).

The NMR data were in agreement with those reported in the literature.[14]

The synthesis of **3l**:

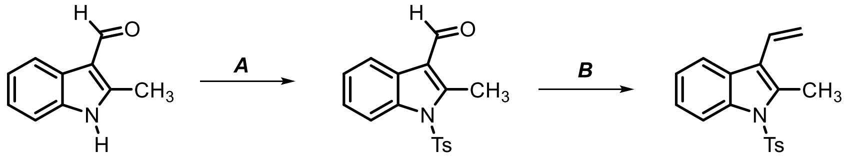


***Procedure A:*** Diisopropylethylamine (3.93 mL, 22.6 mmol) was added to a solution of 2-methyl 1H-indole-3-carbaldehyde (3.0 g, 18.8 mmol) and *p*-toluenesulfonyl chloride (3.94 g, 20.7 mmol) in acetonitrile (50 mL). After 18 h, the reaction mixture was poured into aqueous hydrochloric acid (1 M, 500 mL). The aqueous layer was extracted with ethyl acetate (3 × 150 mL). The combined organic layers were washed with brine (25 mL),

dried over $MgSO_4$, filtered, and concentrated in vacuo. The crude product was purified using silica gel column chromatography to afford 2-methyl-1-tosyl-1H-indole-3-carbaldehyde as a yellow solid.

***Procedure B:*** n-Butyllithium (2.5 M solution in hexanes, 1.0 mL, 2.5 mmol) was added over 5 minutes to a suspension of the methyltriphenylphosphonium bromide (1.1 g, 3.0 mmol) in anhydrous tetrahydrofuran (25 mL) maintained at 0 °C under an atmosphere of dry nitrogen. After complete addition, the reaction mixture was allowed to warm to room temperature and stirred for 1 h. The resulting orange colored solution, containing a small amount of unreacted solid phosphonium salt, was cooled to -15 °C. 2-Methyl-1-tosyl-1H-indole-3-carbaldehyde (0.63 g, 2.0 mmol) was added and the reaction mixture was allowed to warm to -5 °C over a period of 1 h. The reaction was quenched with water (25 mL) and extracted with ethyl acetate (3 × 10 mL). The organic extract was washed with brine (10 mL), dried over anhydrous magnesium sulfate, filtered, and concentrated in vacuo. The crude product was purified using silica gel column chromatography (pentane/EtOAc 5/1).

**$^1$H NMR** (300 MHz, $CDCl_3$) δ 8.15 (d, *J* = 7.0 Hz, 1H), 7.59 (dd, *J* = 13.9, 8.4 Hz, 3H), 7.26 – 7.14 (m, 2H), 7.10 (d, *J* = 8.0 Hz, 2H), 6.65 (dd, *J* = 17.8, 11.6 Hz, 1H), 5.61 (d, *J* = 17.8 Hz, 1H), 5.35 (d, *J* = 11.5 Hz, 1H), 2.54 (s, 3H), 2.24 (s, 3H).

**$^{13}$C NMR** (76 MHz, $CDCl_3$) δ 144.8, 136.5, 136.3, 134.6, 129.9, 128.6, 127.7, 126.4, 124.2, 123.7, 119.5, 118.5, 116.8, 114.6, 21.6, 13.1.

The NMR data were in agreement with those reported in the literature.[15]

The synthesis of **5h**:

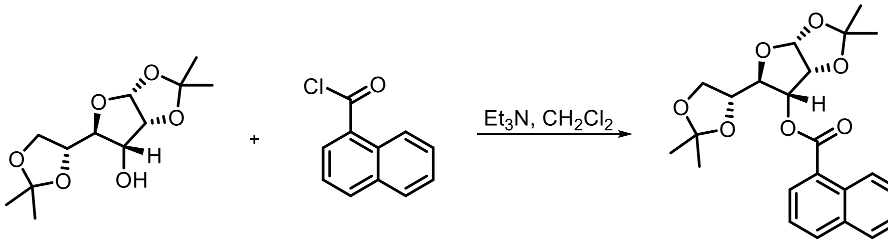


Into a 100-mL round-bottomed flask, equipped with a dropping funnel and charged with argon, 20 mL of dichloromethane, 1,2:5,6-di-O-isopropyliden-α-D-allofuranos (1.0 g, 3.8 mmol) and triethylamine (1.0 g, 10 mmol) were added. To this solution cooled in an ice-water bath, 1-naphthoyl chloride (722 mg, 3.8 mmol) was added dropwise over 10 min. Reaction mixture was stirred for additional 50 min at 0 °C and at room temperature for 3 h. The mixture was poured into 1 M HCl (50 mL), and extracted with diethyl ether (3 × 30 mL). The separated organic layer was washed with aqueous $NaHCO_3$ (50 mL), and dried over anhydrous $MgSO_4$. After evaporation of the solvent under reduced pressure, components of the residue were separated by column chromatography on silica gel (pentane/EtOAc 5/1).

**$^1$H NMR** (300 MHz, $CDCl_3$) δ 8.96 (d, *J* = 8.6 Hz, 1H), 8.29 – 8.13 (m, 1H), 8.11 – 7.98 (m, 1H), 7.89 (dd, *J* = 8.2, 1.6 Hz, 1H), 7.67 – 7.46 (m, 3H), 5.94 (d, *J* = 3.7 Hz, 1H), 5.27 – 5.14 (m, 1H), 5.07 (dd, *J* = 5.1, 3.8 Hz,

1H), 4.49 – 4.34 (m, 2H), 4.12 (ddd, $J$ = 8.0, 4.5, 1.5 Hz, 1H), 4.05 – 3.92 (m, 1H), 1.59 (s, 3H), 1.43 (s, 3H), 1.35 (d, $J$ = 3.3 Hz, 6H).

**$^{13}$C NMR** (76 MHz, $CDCl_3$) δ 166.6, 133.8, 133.7, 131.4, 130.5, 128.6, 127.8, 126.5, 126.3, 125.9, 124.5, 113.2, 110.1, 104.3, 77.9, 77.8, 75.3, 73.5, 65.9, 26.8, 26.7, 26.4, 25.1.

**HRMS** (ESI) m/z: $[M + Na]^+$ Calcd for $C_{23}H_{26}O_7Na$ 437.1571; found 437.1571.

The synthesis of **5r**:

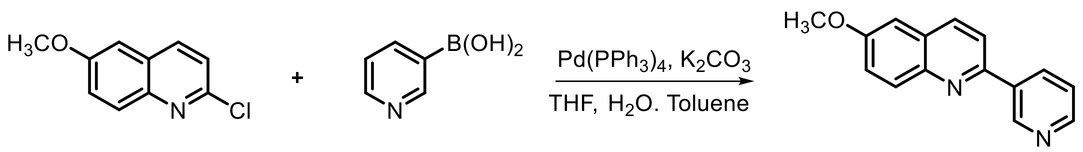


Under nitrogen atmosphere a two neck round bottom flask was charged with boronic acid (1.0 mmol), 2-chloro-6-methoxyquinoline (1 mmol), $Pd(PPh_3)_4$ (5 mol%) and potassium carbonate (3.0 mmol). The flask was protected against light. Degassed water (2.5 ml), degassed THF (2.5 ml), toluene (5 ml) were added via syringe. The mixture was heated at 80 $^{o}$C for 40 h and then diluted with $CH_2Cl_2$. The aqueous phase was extracted twice with $CH_2Cl_2$. The combined organic layers were washed with water and dried over $MgSO_4$. After removal of solvent the crude product was purified by column chromatography on silica gel (pentane/EtOAc 8/1).

**$^{1}$H NMR** (300 MHz, $CDCl_3$) δ 9.30 (d, $J$ = 1.5 Hz, 1H), 8.64 (dd, $J$ = 4.8, 1.7 Hz, 1H), 8.42 (ddd, $J$ = 8.0, 2.3, 1.6 Hz, 1H), 8.08 (d, $J$ = 8.3 Hz, 1H), 8.02 (d, $J$ = 9.2 Hz, 1H), 7.77 (d, $J$ = 8.6 Hz, 1H), 7.43 – 7.33 (m, 2H), 7.04 (d, $J$ = 2.8 Hz, 1H), 3.90 (s, 3H).

**$^{13}$C NMR** (76 MHz, $CDCl_3$) δ 158.0, 152.1, 149.9, 148.6, 144.5, 135.8, 135.2, 134.6, 131.2, 128.4, 123.6, 122.8, 118.7, 104.9, 55.6.

**HRMS** (ESI) m/z: $[M + H]^+$ Calcd for $C_{15}H_{13}N_2O$ 237.1022; found 237.1022.

The synthesis of **5t**:

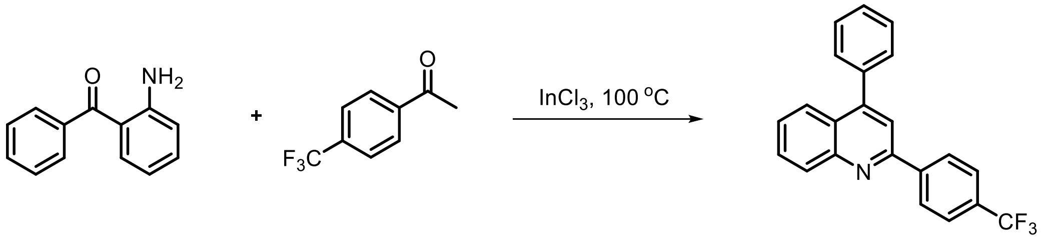


A mixture of 2-aminobenzophenone (10.0 mmol), acetophenone (12.0 mmol), and $InCl_3$ (2.0 mmol) under solvent-free conditions was heated at 100 °C in a 25 mL round-bottomed flask. After completion of the reaction (monitored by TLC), water (15 mL) was added and the reaction mixture was extracted with EtOAc (3 × 10 mL). The combined organic extract was washed with brine and dried over anhydrous $MgSO_4$. The solvent was

evaporated under reduced pressure and the residue was purified by column chromatography on silica gel (pentane/ EtOAc 20/1).

**$^1$H NMR** (300 MHz, $CDCl_3$) δ 8.32 (d, *J* = 8.1 Hz, 2H), 8.26 (d, *J* = 8.1 Hz, 1H), 7.98 – 7.89 (m, 1H), 7.86 – 7.74 (m, 4H), 7.62 – 7.46 (m, 6H).

**$^{19}$F NMR** (282 MHz, $CDCl_3$) δ -62.50.

The NMR data were in agreement with those reported in the literature.[16]

The synthesis of **15**:

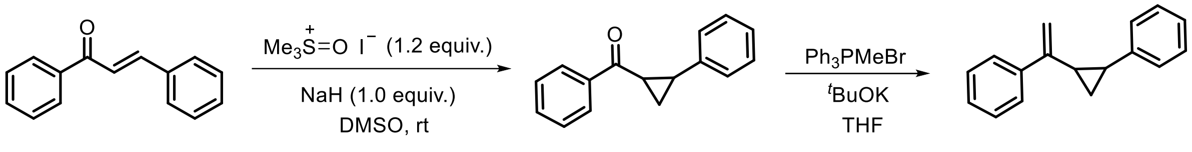


***Procedure A:*** To a two-necked round flask, sodium hydride (60% in mineral oil, 1.0 equiv.) was added. Then, dry DMSO and trimethylsulfoxonium iodide (1.2 equiv.) were added to the flask at room temperature under an Ar atmosphere. After hydrogen evolution ceased, the reaction mixture was stirred for additional 15 minutes, during which time the solution became clear. Chalcone (1.0 equiv.) was then added in one portion to the clear solution. The reaction solution was stirred at room temperature for 5 h. After completion of the reaction as detected by TLC, the reaction was quenched with ice cold water and extracted with diethyl ether (3 × 30 mL). The combined organic layer was washed with brine, dried over $Na_2SO_4$. The product was purified by flash silica gel column chromatography using (petroleum ether/ethyl acetate) as eluent to afford the cyclopropyl ketone product.

***Procedure B:*** Then, to an oven dried flask, methyl triphenylphosphonium bromide (12 mmol, 1.2 equiv.) and THF (30 mL) were added. The suspension was cooled to 0 $^o$C, *t*-BuOK (12 mmol, 1.2 equiv.) was added and the resulting yellow suspension was stirred at 0 $^o$C for 1 hour. To this suspension, a solution of the cyclopropyl ketone (10 mmol, 1.0 equiv.) in THF (10 mL) was added slowly and the resulting mixture was warmed gradually to room temperature and stirred at room temperature for 18 hours. The reaction mixture was concentrated under reduced pressure and filtered over celite. The filtrate was concentrated under reduced pressure and the crude product was purified using silica gel column chromatography (pentane/EtOAc 5/1).

**$^1$H NMR** (300 MHz, $CDCl_3$) δ 7.58 – 7.41 (m, 2H), 7.38 – 7.02 (m, 8H), 5.36 (s, 1H), 5.03 (s, 1H), 2.08 – 1.89 (m, 2H), 1.43 – 1.36 (m, 1H), 1.30 – 1.27 (m, 1H).

The NMR data were in agreement with those reported in the literature.[17]

## 4. Synthesis and characterization of products

### 4.1 Synthesis and characterization of products 2

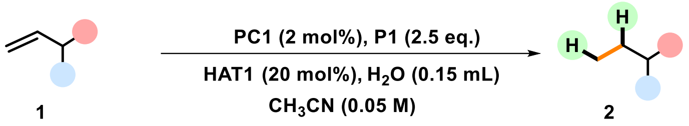


**General procedure (GP1) for the reduction of unactivated alkenes:** To an oven dried Schlenk tube with a magnetic stirring bar, **P1** (0.5 mmol, 2.5 equiv.), **PC1** (0.004 mmol, 2 mol%), deionized water (0.15 mL) and acetonitrile (4.0 mL) were added under argon atmosphere using standard Schlenk techniques at ambient temperature. Then, the alkene **1** (0.2 mmol, 1.0 equiv.) and the HAT catalyst **HAT1** (0.04 mmol, 0.2 equiv.) were added under argon atmosphere. The tube was sealed and placed in the photoreactor, and then irradiated with a 5 W 455 nm LED at 20 ºC using the standard set-up. After 72 hours, the irradiation was stopped. The volatiles were removed in vacuo, then the crude residue was purified by flash column chromatography on silica (pentane, pentane/EtOAc or pentane/$Et_2O$ mixtures, as detailed in the individual entries), to afford the corresponding product **2**.

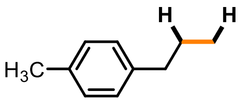


**2a**, 81%

1-Methyl-4-propylbenzene **2a**. According to the **GP1**, using the alkene **1a** (0.2 mmol, 1.0 equiv.) and stirring at room temperature for 72 h under 5 W blue LED irradiation. The crude residue was purified by chromatography (pentane) to give **2a** (21.7 mg, 0.16 mmol, 81%) as a colorless oil.

**$^1$H NMR** (300 MHz, $CDCl_3$) δ 7.17 – 6.99 (m, 4H), 2.68 – 2.51 (m, 2H), 2.36 (s, 3H), 1.67 (h, *J* = 7.4 Hz, 2H), 0.98 (t, *J* = 7.3 Hz, 3H).

**$^{13}$C NMR** (76 MHz, $CDCl_3$) δ 139.7, 135.0, 128.9, 128.4, 37.7, 24.8, 21.0, 13.9.

The NMR data were in agreement with those reported in the literature.[18]

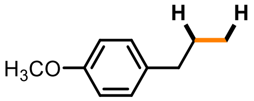


**2b**, 89%

1-Methoxy-4-propylbenzene **2b**. According to the **GP1**, using the alkene **1b** (0.2 mmol, 1.0 equiv.) and stirring at room temperature for 72 h under 5 W blue LED irradiation. The crude residue was purified by chromatography (pentane) to give **2b** (26.7 mg, 0.18 mmol, 89%) as a colorless oil.

$^{1}$**H NMR** (300 MHz, $CDCl_3$) δ 7.12 – 7.05 (m, 2H), 6.83 (d, *J* = 8.7 Hz, 2H), 3.79 (s, 3H), 2.53 (dd, *J* = 8.5, 6.8 Hz, 2H), 1.67 – 1.55 (m, 2H), 0.93 (t, *J* = 7.3 Hz, 3H).

$^{13}$**C NMR** (76 MHz, $CDCl_3$) δ 157.7, 134.8, 129.3, 113.6, 55.3, 37.2, 24.8, 13.8.

The NMR data were in agreement with those reported in the literature.[19]

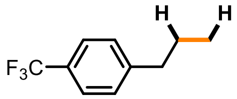


**2c**, 76%

1-Propyl-4-(trifluoromethyl)benzene **2c**. According to the **GP1**, using the alkene **1c** (0.2 mmol, 1.0 equiv.) and stirring at room temperature for 72 h under 5 W blue LED irradiation. The crude residue was purified by chromatography (pentane) to give **2c** (28.58 mg, 0.152 mmol, 76%) as a colorless liquid along with the unreacted alkene **1c** and 2,4,6-triisopropylbenzenethiol.

$^{1}$**H NMR** (300 MHz, $CDCl_3$) δ 7.53 (d, *J* = 7.7 Hz, 2H), 7.28 (d, *J* = 8.2 Hz, 2H), 2.64 (t, *J* = 7.6 Hz, 2H), 1.74 – 1.57 (m, 2H), 0.95 (t, *J* = 7.3 Hz, 3H).

The NMR data were in agreement with those reported in the literature.[20]

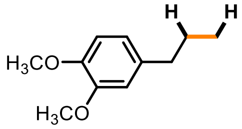


**2d**, 91%

1,2-Dimethoxy-4-propylbenzene **2d**. According to the **GP1**, using the alkene **1d** (0.2 mmol, 1.0 equiv.) and stirring at room temperature for 72 h under 5 W blue LED irradiation. The crude residue was purified by chromatography (pentane/EtOAc 10/1) to give **2d** (32.8 mg, 0.18 mmol, 91%) as a colorless oil.

$^{1}$**H NMR** (400 MHz, $CDCl_3$) δ 6.79 (d, *J* = 8.6 Hz, 1H), 6.72 (dd, *J* = 6.0, 2.1 Hz, 2H), 3.87 (s, 3H), 3.85 (s, 3H), 2.73 – 2.38 (m, 2H), 1.72 – 1.49 (m, 2H), 0.94 (t, *J* = 7.3 Hz, 3H).

$^{13}$**C NMR** (101 MHz, $CDCl_3$) δ 148.8, 147.1, 135.4, 120.2, 111.9, 111.2, 55.9, 55.8, 37.7, 24.8, 13.8.

The NMR data were in agreement with those reported in the literature.[21]

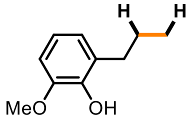


**2e**, 90%

2-Methoxy-6-propylphenol **2e**. According to the **GP1**, using the alkene **1e** (0.2 mmol, 1.0 equiv.) and stirring at room temperature for 72 h under 5 W blue LED irradiation. The crude residue was purified by chromatography (pentane/EtOAc 5/1 to 3/1) to give **2e** (29.9 mg, 0.18 mmol, 90%) as a colorless solid.

**$^1$H NMR** (300 MHz, $CDCl_3$) δ 6.85 – 6.64 (m, 3H), 5.68 (s, 1H), 3.89 (s, 3H), 2.72 – 2.54 (m, 2H), 1.75 – 1.56 (m, 2H), 0.98 (t, *J* = 7.4 Hz, 3H).

**$^{13}$C NMR** (76 MHz, $CDCl_3$) δ 146.3, 143.5, 128.5, 122.4, 119.1, 108.2, 56.0, 31.8, 22.9, 14.1.

The NMR data were in agreement with those reported in the literature.[21]

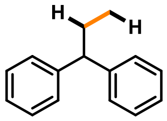


**2f**, 68%

Propane-1,1-diyldibenzene **2f**. According to the **GP1**, using the alkene **1f** (0.2 mmol, 1.0 equiv.) and stirring at room temperature for 72 h under 5 W blue LED irradiation. The crude residue was purified by chromatography (pentane) to give **2f** ($26.6_5$ mg, 0.14 mmol, 68%) as a colorless liquid.

**$^1$H NMR** (300 MHz, $CDCl_3$) δ 7.40 – 7.29 (m, 8H), 7.25 (tdd, *J* = 7.1, 3.3, 1.7 Hz, 2H), 3.88 (t, *J* = 7.8 Hz, 1H), 2.16 (p, *J* = 7.4 Hz, 2H), 1.07 – 0.87 (m, 3H).

**$^{13}$C NMR** (76 MHz, $CDCl_3$) δ 145.2, 128.4, 128.0, 126.1, 53.3, 28.7, 12.9.

The NMR data were in agreement with those reported in the literature.[22]

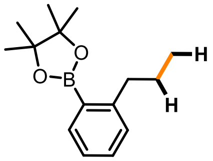


**2g**, 82%

4,4,5,5-Tetramethyl-2-(2-propylphenyl)-1,3,2-dioxaborolane **2g**. According to the **GP1**, using the alkene **1g** (0.2 mmol, 1.0 equiv.) and stirring at room temperature for 72 h under 5 W blue LED irradiation. The crude residue was purified by chromatography (pentane) to give **2g** (40.3 mg, 0.16 mmol, 82%) as a colorless liquid.

**$^1$H NMR** (300 MHz, $CDCl_3$) δ 7.68 (dd, *J* = 7.7, 1.6 Hz, 1H), 7.30 – 7.21 (m, 1H), 7.09 (ddd, *J* = 8.2, 5.3, 1.6 Hz, 2H), 2.83 – 2.71 (m, 2H), 1.49 (t, *J* = 7.6 Hz, 2H), 1.26 (s, 12H), 0.87 (t, *J* = 7.3 Hz, 3H).

**$^{13}$C NMR** (76 MHz, $CDCl_3$) δ 149.8, 136.0, 130.7, 129.2, 124.9, 83.4, 37.9, 26.6, 24.9, 14.1.

The NMR data were in agreement with those reported in the literature.[23]

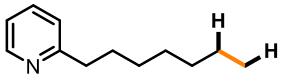


**2h**, 58%

2-Heptylpyridine **2h**. According to the **GP1**, using the alkene **1h** (0.2 mmol, 1.0 equiv.) and stirring at room temperature for 72 h under 5 W blue LED irradiation. The crude residue was purified by chromatography (pentane) to give **2h** (20.5 mg, 0.12 mmol, 58%) as a colorless liquid.

**$^{1}$H NMR** (300 MHz, $CDCl_3$) δ 8.45 (ddd, *J* = 4.9, 1.9, 0.9 Hz, 1H), 7.50 (td, *J* = 7.7, 1.9 Hz, 1H), 7.06 (d, *J* = 7.8 Hz, 1H), 7.04 – 6.98 (m, 1H), 2.81 – 2.54 (m, 2H), 1.65 (pd, *J* = 7.3, 3.6 Hz, 2H), 1.40 – 1.09 (m, 8H), 0.95 – 0.64 (m, 3H).

**$^{13}$C NMR** (76 MHz, $CDCl_3$) δ 162.6, 149.2, 136.2, 122.7, 120.8, 38.5, 31.8, 29.9, 29.4, 29.2, 22.6, 14.1.

The NMR data were in agreement with those reported in the literature.[24]

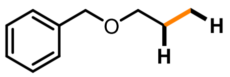

**2i**, 80%

(Propoxymethyl)benzene **2i**. According to the **GP1**, using the alkene **1i** (0.2 mmol, 1.0 equiv.) and stirring at room temperature for 120 h under 5 W blue LED irradiation. The crude residue was purified by chromatography (pentane) to give **2i** (24.0 mg, 0.16 mmol, 80%) as a colorless liquid.

**$^{1}$H NMR** (300 MHz, $CDCl_3$) $^{1}$H NMR (300 MHz, $CDCl_3$) δ 7.50 – 7.24 (m, 5H), 4.55 (s, 2H), 3.48 (t, *J* = 6.7 Hz, 2H), 1.69 (h, *J* = 7.2 Hz, 2H), 0.99 (t, *J* = 7.4 Hz, 3H).

**$^{13}$C NMR** (76 MHz, $CDCl_3$) δ 138.7, 128.4, 127.7, 127.5, 72.9, 72.2, 23.0, 10.7.

The NMR data were in agreement with those reported in the literature.[25]

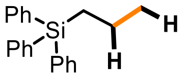

**2j**, 87%

Triphenyl(propyl)silane **2j**. According to the **GP1**, using the alkene **1j** (0.2 mmol, 1.0 equiv.) and stirring at room temperature for 72 h under 5 W blue LED irradiation. The crude residue was purified by chromatography (pentane) to give **2j** (52.5 mg, 0.17 mmol, 87%) as a colorless liquid.

**$^{1}$H NMR** (400 MHz, $CDCl_3$) δ 7.48 – 7.42 (m, 6H), 7.31 – 7.24 (m, 9H), 1.48 – 1.37 (m, 2H), 1.32 – 1.25 (m, 2H), 0.92 (t, *J* = 7.1 Hz, 3H).

**$^{13}$C NMR** (101 MHz, $CDCl_3$) δ 135.7, 135.5, 129.4, 127.9, 18.6, 17.7, 16.0.

The NMR data were in agreement with those reported in the literature.[26]

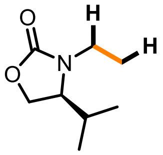

**2k**, 89%

(S)-3-Ethyl-4-isopropyloxazolidin-2-one **2k**. According to the **GP1**, using the alkene **1k** (0.2 mmol, 1.0 equiv.) and stirring at room temperature for 72 h under 5 W blue LED irradiation. The crude residue was purified by chromatography (pentane/EtOAc 5/1) to give **2k** (27.9 mg, 0.18 mmol, 89%) as a colorless liquid.

**$^1$H NMR** (400 MHz, $CDCl_3$) δ 4.19 (t, *J* = 9.0 Hz, 1H), 4.04 (dd, *J* = 8.9, 5.7 Hz, 1H), 3.76 (ddd, *J* = 9.2, 5.7, 3.6 Hz, 1H), 3.56 (dq, *J* = 14.7, 7.4 Hz, 1H), 3.03 (dq, *J* = 14.2, 7.0 Hz, 1H), 2.04 (ddq, *J* = 10.5, 6.9, 3.5 Hz, 1H), 1.15 (t, *J* = 7.2 Hz, 3H), 0.90 (d, *J* = 7.0 Hz, 3H), 0.87 (d, *J* = 6.9 Hz, 3H).
**$^{13}$C NMR** (101 MHz, $CDCl_3$) δ 158.3, 62.7, 58.7, 36.7, 27.6, 17.7, 14.3, 12.4.
**HRMS** (ESI) m/z: $[M + Na]^+$ Calcd for $C_8H_{15}NO_2Na$ 180.0995; found 180.0995.

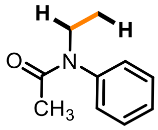

**2l**, 88%

N-Ethyl-N-phenylacetamide **2l**. According to the **GP1**, using the alkene **1l** (0.2 mmol, 1.0 equiv.) and stirring at room temperature for 72 h under 5 W blue LED irradiation. The crude residue was purified by chromatography (pentane/EtOAc 5/1) to give **2l** (28.7 mg, 0.18 mmol, 88%) as a colorless solid.
**$^1$H NMR** (300 MHz, $CDCl_3$) δ 7.41 – 7.22 (m, 3H), 7.15 – 7.01 (m, 2H), 3.68 (q, *J* = 7.2 Hz, 2H), 1.76 (s, 3H), 1.04 (t, *J* = 7.2 Hz, 3H).
**$^{13}$C NMR** (76 MHz, $CDCl_3$) δ 170.3, 142.8, 129.7, 128.2, 127.9, 43.9, 22.8, 13.0.
The NMR data were in agreement with those reported in the literature.[27]

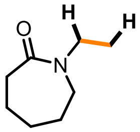

**2m**, 90%

1-Ethylazepan-2-one **2m**. According to the **GP1**, using the alkene **1m** (0.2 mmol, 1.0 equiv.) and stirring at room temperature for 72 h under 5 W blue LED irradiation. The crude residue was purified by chromatography (pentane/EtOAc 5/1) to give **2m** (25.4 mg, 0.18 mmol, 90%) as a colorless solid.
**$^1$H NMR** (300 MHz, $CDCl_3$) δ 3.37 (q, *J* = 6.5 Hz, 2H), 3.34 – 3.25 (m, 2H), 2.54 – 2.40 (m, 2H), 1.73 – 1.52 (m, 6H), 1.06 (t, *J* = 7.2 Hz, 3H).
**$^{13}$C NMR** (76 MHz, $CDCl_3$) δ 175.8, 49.2, 43.0, 37.1, 30.0, 28.7, 23.3, 13.2.
The NMR data were in agreement with those reported in the literature.[28]

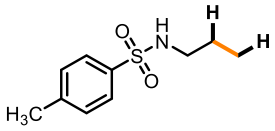

**2n**, 30%

4-Methyl-N-propylbenzenesulfonamide **2n**. According to the **GP1**, using the alkene **1n** (0.2 mmol, 1.0 equiv.) and stirring at room temperature for 120 h under 5 W blue LED irradiation. The crude residue was purified by chromatography (pentane) to give **2n** (12.8 mg, 0.06 mmol, 30%) as a colorless liquid along with the inseparably unreacted alkene **1n**.

**$^{1}$H NMR** (300 MHz, $CDCl_3$) δ 7.74 (s, 3H), 7.26 (d, *J* = 1.0 Hz, 2H), 4.60 (s, 1H), 2.89 (q, *J* = 6.8 Hz, 2H), 1.47 (h, *J* = 7.3 Hz, 2H), 0.86 (t, *J* = 7.4 Hz, 3H).

The NMR data were in agreement with those reported in the literature.[29]

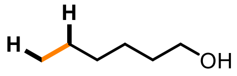

**2o**, 78%

Hexan-1-ol **2o**. According to the **GP1**, using the alkene **1o** (0.2 mmol, 1.0 equiv.) and stirring at room temperature for 72 h under 5 W blue LED irradiation. The crude residue was purified by chromatography (pentane/EtOAc 20/1) to give **2o** (15.9 mg, 0.16 mmol, 78%) as a colorless liquid.

**$^{1}$H NMR** (300 MHz, $CDCl_3$) δ 3.73 – 3.55 (m, 2H), 1.59 (td, *J* = 15.2, 8.2 Hz, 3H), 1.45 – 1.19 (m, 6H), 1.07 – 0.77 (m, 3H).

**$^{13}$C NMR** (76 MHz, $CDCl_3$) δ 63.0, 63.0, 32.8, 31.6, 25.4, 22.6, 14.0.

The NMR data were in agreement with those reported in the literature.[30]

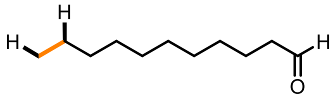

**2p**, 71%

Undecanal **2p**. According to the **GP1**, using the alkene **1p** (0.2 mmol, 1.0 equiv.) and stirring at room temperature for 72 h under 5 W blue LED irradiation. The yield was determined by the GC with tetradecane as the internal standard (71%).

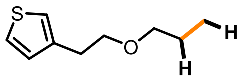

**2q**, 73%

3-(2-Propoxyethyl)thiophene **2q**. According to the **GP1**, using the alkene **1q** (0.2 mmol, 1.0 equiv.) and stirring at room temperature for 72 h under 5 W blue LED irradiation. The crude residue was purified by chromatography (pentane/EtOAc 10/1) to give **2q** (24.8 mg, 0.15 mmol, 73%) as a yellow liquid.

**$^1$H NMR** (300 MHz, $CDCl_3$) δ 7.26 – 7.22 (m, 1H), 7.07 – 6.92 (m, 2H), 3.64 (t, *J* = 7.1 Hz, 2H), 3.41 (t, *J* = 6.7 Hz, 2H), 2.92 (t, *J* = 7.1 Hz, 2H), 1.68 – 1.56 (m, 2H), 0.92 (t, *J* = 7.4 Hz, 3H).

**$^{13}$C NMR** (76 MHz, $CDCl_3$) δ 139.4, 128.6, 125.2, 121.1, 72.7, 71.0, 30.8, 22.9, 10.6.

The NMR data were in agreement with those reported in the literature.[31]

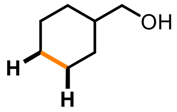

**2r**, 65%

Cyclohexylmethanol **2r**. According to the **GP1**, using the alkene **1r** (0.2 mmol, 1.0 equiv.) and stirring at room temperature for 72 h under 5 W blue LED irradiation. The crude residue was purified by chromatography (pentane/EtOAc 20/1) to give **2r** (14.8 mg, 0.13 mmol, 65%) as a colorless liquid.

**$^1$H NMR** (300 MHz, $CDCl_3$) δ 3.43 (d, *J* = 6.3 Hz, 2H), 1.86 – 1.62 (m, 6H), 1.59 – 1.38 (m, 1H), 1.37 – 1.03 (m, 3H), 0.93 (qd, *J* = 12.4, 3.6 Hz, 2H).

**$^{13}$C NMR** (76 MHz, $CDCl_3$) δ 68.7, 40.5, 29.6, 26.6, 25.8.

The NMR data were in agreement with those reported in the literature.[32]

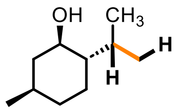

**2s**, 42%

(1R,2S,5R)-2-Isopropyl-5-methylcyclohexan-1-ol **2s**. According to the **GP1**, using the alkene **1s** (0.2 mmol, 1.0 equiv.) and stirring at room temperature for 120 h under 5 W blue LED irradiation. The yield was determined by the GC with tetradecane as the internal standard (42%).

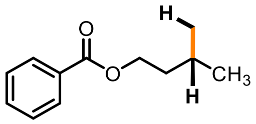

**2t**, 41%

Isopentyl benzoate **2t**. According to the **GP1**, using the alkene **1t** (0.2 mmol, 1.0 equiv.) and stirring at room temperature for 120 h under 5 W blue LED irradiation. The yield was determined by the GC with tetradecane as the internal standard (41%).

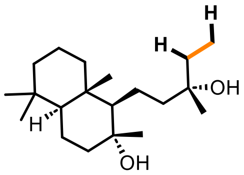

**2u**, 81%

(1R,2R,4aS,8aS)-1-((S)-3-Hydroxy-3-methylpentyl)-2,5,5,8a-tetramethyldecahydronaphthalen-2-ol **2u**. According to the **GP1**, using the alkene **1u** (0.2 mmol, 1.0 equiv.) and stirring at room temperature for 120 h under 5 W blue LED irradiation. The crude residue was purified by chromatography (pentane/EtOAc 10/1) to give **2u** (50.2 mg, 0.16 mmol, 81%) as a colorless liquid.

**$^1$H NMR** (300 MHz, $CDCl_3$) δ 2.34 (s, 2H), 1.83 (dt, *J* = 11.9, 3.1 Hz, 1H), 1.60 (tdd, *J* = 13.9, 7.3, 4.3 Hz, 4H), 1.52 – 1.35 (m, 6H), 1.35 – 1.20 (m, 3H), 1.14 (d, *J* = 7.6 Hz, 8H), 0.94 (t, *J* = 2.9 Hz, 1H), 0.89 (d, *J* = 7.4 Hz, 3H), 0.85 (d, *J* = 2.3 Hz, 4H), 0.78 (s, 3H), 0.77 (s, 3H).

**$^{13}$C NMR** (76 MHz, $CDCl_3$) δ 74.7, 73.4, 62.1, 56.1, 44.2, 44.1, 42.0, 39.7, 39.2, 35.8, 33.4, 33.2, 25.4, 24.3, 21.5, 20.5, 18.9, 18.5, 15.4, 8.3.

The NMR data were in agreement with those reported in the literature.[33]

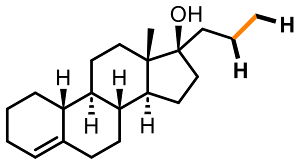

**2v**, 56%

(8R,9S,10R,13S,14S,17S)-13-Methyl-17-propyl-2,3,6,7,8,9,10,11,12,13,14,15,16,17-tetradecahydro-1H-cyclopenta[a]phenanthren-17-ol **2v.** According to the **GP1**, using the alkene **1v** (0.2 mmol, 1.0 equiv.) and stirring at room temperature for 120 h under 5 W blue LED irradiation. The crude residue was purified by chromatography (pentane/EtOAc 10/1) to give **2v** (33.8 mg, 0.11 mmol, 56%) as a colorless liquid.

**$^1$H NMR** (300 MHz, $CDCl_3$) δ 5.38 (s, 1H), 2.26 – 2.13 (m, 1H), 2.08 – 1.87 (m, 5H), 1.79 (td, *J* = 8.7, 2.2 Hz, 2H), 1.74 – 1.61 (m, 3H), 1.60 – 1.41 (m, 5H), 1.41 – 1.30 (m, 4H), 1.28 – 1.17 (m, 2H), 1.16 (d, *J* = 3.7 Hz, 1H), 1.13 – 1.00 (m, 3H), 0.99 – 0.87 (m, 7H).

**$^{13}$C NMR** (76 MHz, $CDCl_3$) δ 140.7, 119.9, 86.6, 47.8, 46.9, 42.4, 42.3, 39.8, 37.1, 36.0, 34.8, 32.6, 30.3, 28.4, 26.6, 25.5, 22.0, 21.3, 17.2, 16.3, 15.0.

**HRMS** (ESI) m/z: $[M + Na]^+$ Calcd for $C_{21}H_{34}ONa$ 325.2502; found 325.2502.

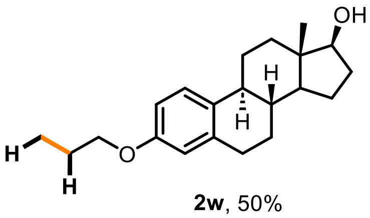


**2w**, 50%

(8R,9S,13S,17S)-13-Methyl-3-propoxy-7,8,9,11,12,13,14,15,16,17-decahydro-6H-cyclopenta[a]phenanthre17 ol **2w**. According to the **GP1**, using the alkene **1w** (0.2 mmol, 1.0 equiv.) and stirring at room temperature for 120 h under 5 W blue LED irradiation. The yield was determined by the $^1$H NMR spectrum (50%, $^1$H NMR yield) along with a small amount of inseparable alkene **1w**.

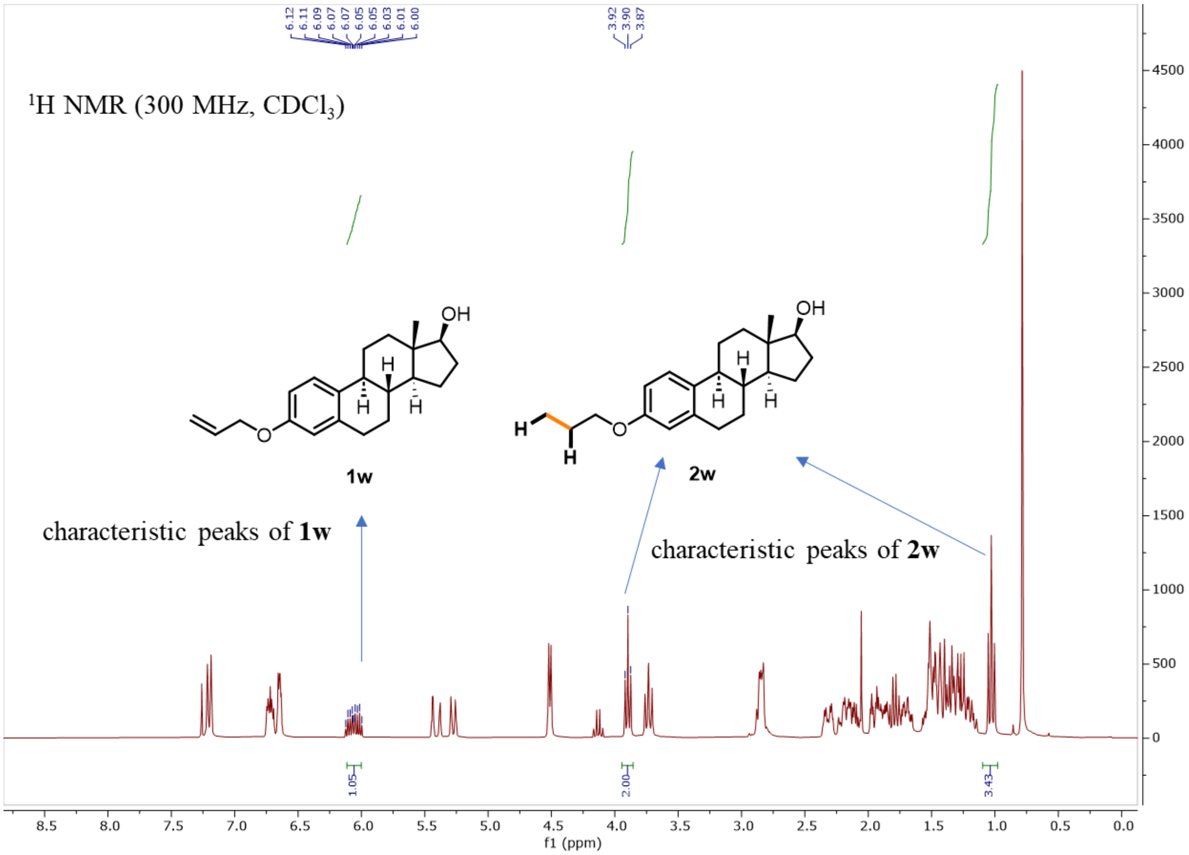

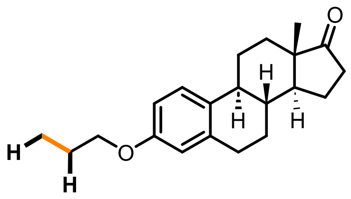


**2x**, 76%

(8R,9S,13S,14S)-13-Methyl-3-propoxy-6,7,8,9,11,12,13,14,15,16-decahydro-17H-cyclopenta[a]phenanthren-17-one **2x**. According to the **GP1**, using the alkene **1x** (0.2 mmol, 1.0 equiv.) and stirring at room temperature for 120 h under 5 W blue LED irradiation. The crude residue was purified by chromatography (pentane/EtOAc 5/1) to give **2x** (47.2 mg, 0.15 mmol, 76%) as a colorless liquid.

**$^1$H NMR** (300 MHz, $CDCl_3$) δ 7.20 (dd, $J$ = 8.7, 1.1 Hz, 1H), 6.72 (dd, $J$ = 8.6, 2.8 Hz, 1H), 6.66 (d, $J$ = 2.7 Hz, 1H), 3.90 (t, $J$ = 6.6 Hz, 2H), 2.90 (dt, $J$ = 8.4, 4.3 Hz, 2H), 2.58 – 2.46 (m, 1H), 2.40 (dp, $J$ = 9.3, 2.6 Hz, 1H), 2.25 (t, $J$ = 11.2 Hz, 1H), 2.18 – 1.92 (m, 4H), 1.87 – 1.73 (m, 2H), 1.69 – 1.36 (m, 6H), 1.04 (t, $J$ = 7.4 Hz, 3H), 0.92 (s, 3H).

**$^{13}$C NMR** (76 MHz, $CDCl_3$) δ 221.0, 157.2, 137.7, 131.8, 126.3, 114.6, 112.1, 69.4, 50.4, 48.0, 44.0, 38.4, 35.9, 31.6, 29.7, 26.6, 26.0, 22.7, 21.6, 13.9, 10.6.

The NMR data were in agreement with those reported in the literature.[11]

## 4.2 Synthesis and characterization of products 4

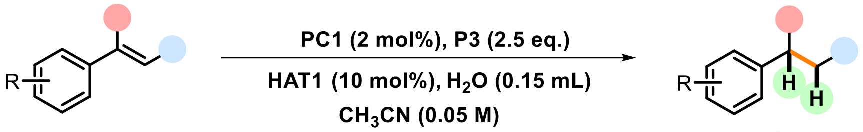


**General procedure (GP2) for the reduction of styrene derivates:** To an oven dried Schlenk tube with a magnetic stirring bar, **P3** (0.5 mmol, 2.5 equiv.), **PC1** (0.004 mmol, 2 mol%), deionized water (0.15 mL) and acetonitrile (4.0 mL) were added under argon atmosphere using standard Schlenk techniques at ambient temperature. Then, the alkene **3** (0.2 mmol, 1.0 equiv.) and the HAT catalyst **HAT1** (0.03 mmol, 0.15 equiv.) were added under argon atmosphere. The tube was sealed and placed in the photoreactor, and then irradiated with a 5 W 455 nm LED at 20 ºC using the standard set-up. After 48-72 hours, the irradiation was stopped. The volatiles were removed in vacuo, then the crude residue was purified by flash column chromatography on silica (pentane, pentane/EtOAc or pentane/$Et_2O$ mixtures, as detailed in the individual entries), to afford the corresponding product **4**.

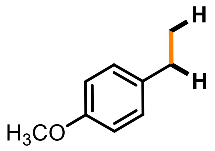

**4a**, 72%

1-Ethyl-4-methoxybenzene **4a**. According to the **GP2**, using the alkene **3a** (0.2 mmol, 1.0 equiv.) and stirring at room temperature for 48 h under 5 W blue LED irradiation. The crude residue was purified by chromatography (pentane) to give **4a** (19.6 mg, 0.14 mmol, 72%) as a colorless liquid.

**$^1$H NMR** (300 MHz, $CDCl_3$) δ 7.15 (d, *J* = 8.8 Hz, 2H), 6.86 (d, *J* = 8.7 Hz, 2H), 3.81 (d, *J* = 1.1 Hz, 3H), 2.62 (q, *J* = 7.7 Hz, 2H), 1.24 (t, *J* = 7.6 Hz, 3H).

**$^{13}$C NMR** (76 MHz, $CDCl_3$) δ 157.7, 136.4, 128.7, 113.8, 55.3, 28.0, 16.0.

The NMR data were in agreement with those reported in the literature.[11]

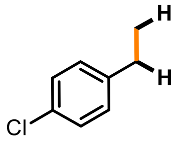

**4b**, 72%

1-Chloro-4-ethylbenzene **4b**. According to the **GP2**, using the alkene **3b** (0.2 mmol, 1.0 equiv.) and stirring at room temperature for 72 h under 5 W blue LED irradiation. The yield was determined by the GC with tetradecane as the internal standard (72%).

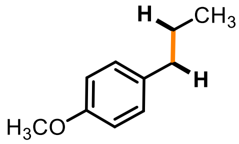

**4c**, 86%

1-Methoxy-4-propylbenzene **4c**. According to the **GP2**, using the alkene **3c** (0.2 mmol, 1.0 equiv.) and stirring at room temperature for 48 h under 5 W blue LED irradiation. The crude residue was purified by chromatography (pentane) to give **4c** (25.8 mg, 0.17 mmol, 86%) as a colorless liquid.

**$^1$H NMR** (300 MHz, $CDCl_3$) δ 7.12 – 7.05 (m, 2H), 6.83 (d, *J* = 8.7 Hz, 2H), 3.79 (s, 3H), 2.53 (dd, *J* = 8.5, 6.8 Hz, 2H), 1.67 – 1.55 (m, 2H), 0.93 (t, *J* = 7.3 Hz, 3H).

**$^{13}$C NMR** (76 MHz, $CDCl_3$) δ 157.7, 134.8, 129.3, 113.6, 55.3, 37.2, 24.8, 13.8.

The NMR data were in agreement with those reported in the literature.[11]

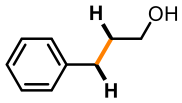

**4d**, 85%

3-Phenylpropan-1-ol **4d**. According to the **GP2**, using the alkene **3d** (0.2 mmol, 1.0 equiv.) and stirring at room temperature for 48 h under 5 W blue LED irradiation. The crude residue was purified by chromatography (pentane/EtOAc 10/1) to give **4d** (23.1 mg, 0.17 mmol, 85%) as a colorless liquid.

**$^1$H NMR** (400 MHz, $CDCl_3$) δ 7.25 – 7.17 (m, 2H), 7.16 – 7.08 (m, 3H), 3.61 (t, *J* = 6.4 Hz, 2H), 2.71 – 2.55 (m, 2H), 1.91 – 1.74 (m, 2H).

**$^{13}$C NMR** (101 MHz, $CDCl_3$) δ 141.8, 128.4, 128.4, 125.9, 62.3, 34.2, 32.1.

The NMR data were in agreement with those reported in the literature.[34]

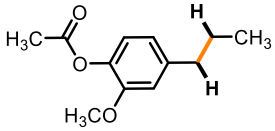

**4e**, 82%

2-Methoxy-4-propylphenyl acetate **4e**. According to the **GP2**, using the alkene **3e** (0.2 mmol, 1.0 equiv.) and stirring at room temperature for 72 h under 5 W blue LED irradiation. The crude residue was purified by chromatography (pentane/EtOAc 10/1) to give **4e** (34.1 mg, 0.16 mmol, 82%) as a colorless liquid.

**$^1$H NMR** (300 MHz, $CDCl_3$) δ 6.85 (d, *J* = 8.0 Hz, 1H), 6.74 – 6.63 (m, 2H), 3.74 (s, 3H), 2.56 – 2.41 (m, 2H), 2.23 (s, 3H), 1.63 – 1.48 (m, 2H), 0.88 (t, *J* = 7.3 Hz, 3H).

**$^{13}$C NMR** (76 MHz, $CDCl_3$) δ 169.3, 150.7, 141.7, 137.6, 122.3, 120.5, 112.6, 55.8, 38.0, 24.6, 20.7, 13.9.

The NMR data were in agreement with those reported in the literature.[35]

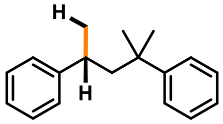

**4f**, 47%

(2-Methylpentane-2,4-diyl)dibenzene **4f**. According to the **GP2**, using the alkene **3f** (0.2 mmol, 1.0 equiv.) and stirring at room temperature for 72 h under 5 W blue LED irradiation. The crude residue was purified by chromatography (pentane) to give **4f** (22.37 mg, 0.094 mmol, 47%) as a colorless liquid along with the inseparable alkene **3f**.

**$^1$H NMR** (300 MHz, $CDCl_3$) δ 7.31 – 7.26 (m, 5H), 7.15 – 7.07 (m, 3H), 7.03 (dd, *J* = 8.2, 1.5 Hz, 2H), 2.55 (td, *J* = 7.1, 5.2 Hz, 1H), 2.07 (dd, *J* = 14.1, 7.0 Hz, 1H), 2.00 – 1.91 (m, 1H), 1.26 (s, 3H), 1.16 (s, 3H), 1.04 (d, *J* = 7.1 Hz, 3H).

The NMR data were in agreement with those reported in the literature.[36]

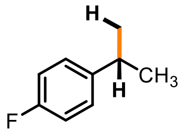

**4g**, 77%

1-Fluoro-4-isopropylbenzene **4g**. According to the **GP2**, using the alkene **3g** (0.2 mmol, 1.0 equiv.) and stirring at room temperature for 72 h under 5 W blue LED irradiation. The yield was determined by the GC with tetradecane as the internal standard (77%).

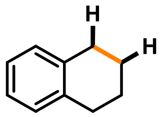

**4h**, 52%

1,2,3,4-Tetrahydronaphthalene **4h**. According to the **GP2**, using the alkene **3h** (0.2 mmol, 1.0 equiv.) and stirring at room temperature for 48 h under 5 W blue LED irradiation. The crude residue was purified by chromatography (pentane) to give **4h** (13.7 mg, 0.10 mmol, 52%) as a colorless liquid.

**$^1$H NMR** (300 MHz, $CDCl_3$) δ 7.15 (d, *J* = 2.0 Hz, 4H), 2.96 – 2.70 (m, 4H), 1.88 (dd, *J* = 4.9, 2.3 Hz, 4H).

**$^{13}$C NMR** (76 MHz, $CDCl_3$) δ 137.2, 129.2, 125.5, 29.4, 23.3.

The NMR data were in agreement with those reported in the literature.[37]

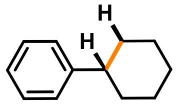

**4i**, 88%

Cyclohexylbenzene **4i**. According to the **GP2**, using the alkene **3i** (0.2 mmol, 1.0 equiv.) and stirring at room temperature for 48 h under 5 W blue LED irradiation. The crude residue was purified by chromatography (pentane) to give **4i** (28.2 mg, 0.18 mmol, 88%) as a colorless liquid.

**$^1$H NMR** (300 MHz, $CDCl_3$) δ 7.35 (t, *J* = 7.5 Hz, 2H), 7.31 – 7.18 (m, 3H), 2.55 (td, *J* = 8.0, 3.9 Hz, 1H), 1.92 (qd, *J* = 9.8, 6.3 Hz, 4H), 1.85 – 1.75 (m, 1H), 1.47 (dq, *J* = 16.0, 11.9 Hz, 4H), 1.32 (td, *J* = 9.5, 4.8 Hz, 1H).

**$^{13}$C NMR** (76 MHz, $CDCl_3$) δ 148.1, 128.3, 126.9, 125.8, 44.7, 34.5, 27.0, 26.2.

The NMR data were in agreement with those reported in the literature.[38]

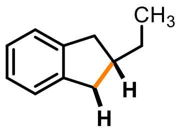

**4j**, 88%

2-Ethyl-2,3-dihydro-1H-indene **4j**. According to the **GP2**, using the alkene **3j** (0.2 mmol, 1.0 equiv.) and stirring at room temperature for 48 h under 5 W blue LED irradiation. The crude residue was purified by chromatography (pentane) to give **4j** (25.7 mg, 0.176 mmol, 88%) as a colorless liquid along with the inseparable 2,4,6-triisopropylbenzenethiol.

**$^1$H NMR** (300 MHz, $CDCl_3$) δ 7.23 – 7.16 (m, 2H), 7.13 (dt, *J* = 5.0, 3.6 Hz, 2H), 3.05 (dd, *J* = 15.3, 7.9 Hz, 2H), 2.59 (dd, *J* = 15.5, 8.1 Hz, 2H), 2.37 (ddd, *J* = 15.4, 8.3, 7.3 Hz, 1H), 1.59 – 1.47 (m, 2H), 0.98 (d, *J* = 7.4 Hz, 4H).

The NMR data were in agreement with those reported in the literature.[39]

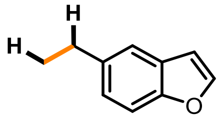

**4k**, 78%

5-Ethylbenzofuran **4k**. According to the **GP1**, using the alkene **3k** (0.2 mmol, 1.0 equiv.) and stirring at room temperature for 72 h under 5 W blue LED irradiation. The yield was determined by the GC with tetradecane as the internal standard (41%).

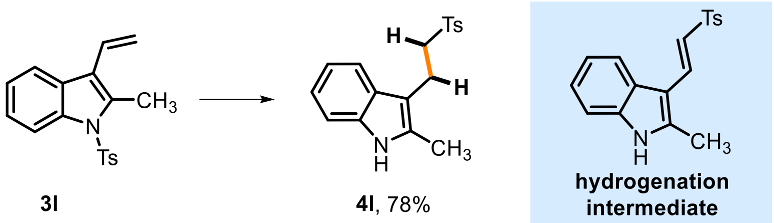


**3l** **4l**, 78%

2-Methyl-3-(2-tosylethyl)-1H-indole **4l**. According to the **GP1**, using the alkene **3l** (0.2 mmol, 1.0 equiv.) and stirring at room temperature for 72 h under 5 W blue LED irradiation. The crude residue was purified by chromatography (pentane/EtOAc 10/1 to 1:1) to give **4l** (48.8 mg, 0.156 mmol, 78%) as a colorless liquid.

**$^1$H NMR** (300 MHz, $CDCl_3$) δ 7.89 – 7.68 (m, 3H), 7.35 (d, *J* = 7.8 Hz, 2H), 7.26 – 7.20 (m, 2H), 7.13 – 6.99 (m, 2H), 3.39 – 3.23 (m, 2H), 3.23 – 3.04 (m, 2H), 2.45 (s, 3H), 2.32 (d, *J* = 1.5 Hz, 3H).

**$^{13}$C NMR** (76 MHz, $CDCl_3$) δ 144.6, 136.2, 135.2, 131.6, 129.9, 128.0, 127.7, 121.4, 119.5, 117.3, 110.4, 107.3, 56.3, 21.7, 17.7, 11.5.

**HRMS** (ESI) m/z: $[M + Na]^+$ Calcd for $C_{18}H_{19}NO_2SNa$ 336.1029; found 336.1029.

Comment: The product was obtained through the hydrogenation of the photoisomerized intermediate of **3l**.[40,41]

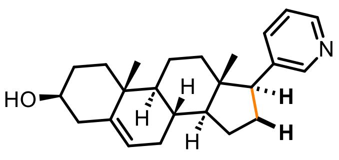

**4m**, 93%, dr > 20 : 1

(3S,8S,9S,10R,13S,14S,17S)-10,13-Dimethyl-17-(pyridin-3-yl)-2,3,4,7,8,9,10,11,12,13,14,15,16,17-tetradecahydro-1H-cyclopenta[a]phenanthren-3-ol **4m**. According to the **GP2**, using the alkene **3m** (0.2 mmol, 1.0 equiv.) and stirring at room temperature for 72 h under 5 W blue LED irradiation. The crude residue was purified by chromatography (pentane/EtOAc 10/1) to give **4m** (65.3 mg, 0.19 mmol, 93%) as a colorless liquid.

**$^1$H NMR** (300 MHz, $CDCl_3$) δ 8.47 – 8.32 (m, 2H), 7.51 (dt, *J* = 7.9, 2.0 Hz, 1H), 7.19 (dd, *J* = 7.9, 4.8 Hz, 1H), 5.45 – 5.22 (m, 1H), 3.67 – 3.39 (m, 1H), 2.65 (t, *J* = 9.8 Hz, 1H), 2.54 (s, 1H), 2.38 – 2.14 (m, 2H), 2.13 – 1.90 (m, 3H), 1.92 – 1.73 (m, 3H), 1.70 – 1.43 (m, 5H), 1.42 – 1.15 (m, 4H), 1.15 – 0.99 (m, 2H), 0.98 (s, 3H), 0.47 (s, 3H).

**$^{13}$C NMR** (76 MHz, $CDCl_3$) δ 150.2, 147.3, 141.1, 136.4, 135.7, 122.8, 121.3, 71.5, 56.5, 54.4, 50.3, 44.3, 42.3, 37.4, 37.3, 36.6, 32.3, 31.9, 31.6, 25.9, 24.5, 20.7, 19.4, 12.6.

The NMR data were in agreement with those reported in the literature.[42]

## 4.3 Synthesis and characterization of products 6

## Synthesis and characterization of products 6a-p

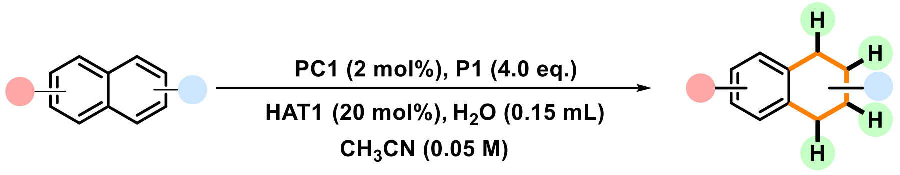


**5** **6**

**General procedure (GP3) for the reduction of naphthalene derivates:** To an oven dried Schlenk tube with a magnetic stirring bar, **P1** (0.8 mmol, 4.0 equiv.), **PC1** (0.004 mmol, 2 mol%), deionized water (0.15 mL) and acetonitrile (4.0 mL) were added under argon atmosphere using standard Schlenk techniques at ambient temperature. Then, the naphthalene **5** (0.2 mmol, 1.0 equiv.) and the HAT catalyst **HAT1** (0.04 mmol, 0.2 equiv.) were added under argon atmosphere. The tube was sealed and placed in the photoreactor, and then irradiated with a 5 W 455 nm LED at 20 °C using the standard set-up. After 72 hours, the irradiation was stopped. The volatiles were removed in vacuo, then the crude residue was purified by flash column chromatography on silica (pentane, pentane/EtOAc or pentane/$Et_2O$ mixtures, as detailed in the individual entries), to afford the corresponding product **6**.

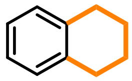

**6a**, 89%

1,2,3,4-Tetrahydronaphthalene **6a**. According to the **GP3**, using the alkene **5a** (0.2 mmol, 1.0 equiv.) and stirring at room temperature for 72 h under 5 W blue LED irradiation. The crude residue was purified by chromatography (pentane) to give **6a** (23.5 mg, 0.18 mmol, 89%) as a colorless liquid.

**$^{1}$H NMR** (300 MHz, $CDCl_3$) δ 7.15 (d, *J* = 2.0 Hz, 4H), 2.96 – 2.70 (m, 4H), 1.88 (dd, *J* = 4.9, 2.3 Hz, 4H).

**$^{13}$C NMR** (76 MHz, $CDCl_3$) δ 137.2, 129.2, 125.5, 29.4, 23.3.

The NMR data were in agreement with those reported in the literature.[37]

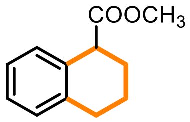


**6b**, 82%

Methyl 1,2,3,4-tetrahydronaphthalene-1-carboxylate **6b**. According to the **GP3**, using the alkene **5b** (0.2 mmol, 1.0 equiv.) and stirring at room temperature for 72 h under 5 W blue LED irradiation. The crude residue was purified by chromatography (pentane/EtOAc 20/1) to give **6b** (31.2 mg, 0.16 mmol, 82%) as a colorless liquid.

**$^{1}$H NMR** (400 MHz, $CDCl_3$) δ 7.23 – 7.06 (m, 4H), 3.89 – 3.82 (m, 1H), 3.72 (s, 3H), 2.92 – 2.71 (m, 2H), 2.21 – 2.09 (m, 1H), 2.06 – 1.94 (m, 2H), 1.84 – 1.70 (m, 1H).

**$^{13}$C NMR** (101 MHz, $CDCl_3$) δ 175.4, 137.2, 133.3, 129.4, 129.3, 126.9, 125.8, 52.0, 44.8, 29.1, 26.7, 20.6.

The NMR data were in agreement with those reported in the literature.[43]

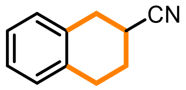


**6c**, 86%

1,2,3,4-Tetrahydronaphthalene-2-carbonitrile **6c**. According to the **GP3**, using the alkene **5c** (0.2 mmol, 1.0 equiv.) and stirring at room temperature for 72 h under 5 W blue LED irradiation. The crude residue was purified by chromatography (pentane/EtOAc 20/1) to give **6c** (27.0 mg, 0.17 mmol, 86%) as a colorless liquid.

**$^{1}$H NMR** (400 MHz, $CDCl_3$) δ 7.13 – 6.94 (m, 4H), 3.04 (td, *J* = 16.7, 6.8 Hz, 2H), 2.96 – 2.86 (m, 2H), 2.77 (ddd, *J* = 17.4, 9.0, 6.1 Hz, 1H), 2.12 (dtdd, *J* = 11.9, 6.0, 3.2, 1.1 Hz, 1H), 2.08 – 1.88 (m, 1H).

**$^{13}$C NMR** (101 MHz, $CDCl_3$) δ 134.5, 132.3, 129.1, 129.0, 126.7, 126.3, 122.1, 32.4, 27.1, 26.3, 25.6.

The NMR data were in agreement with those reported in the literature.[44]

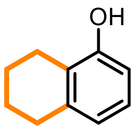

**6d**, 78%

5,6,7,8-Tetrahydronaphthalen-1-ol **6d**. According to the **GP3**, using the alkene **5d** (0.2 mmol, 1.0 equiv.) and stirring at room temperature for 72 h under 5 W blue LED irradiation. The crude residue was purified by chromatography (pentane/EtOAc 5/1) to give **6d** (23.1 mg, 0.156 mmol, 78%) as a colorless liquid.

**$^1$H NMR** (300 MHz, $CDCl_3$) δ 7.10 – 6.95 (m, 1H), 6.74 (t, $J$ = 6.2 Hz, 1H), 6.65 (dd, $J$ = 8.0, 4.8 Hz, 1H), 4.76 (s, 1H), 2.80 (t, $J$ = 5.8 Hz, 2H), 2.67 (t, $J$ = 5.9 Hz, 2H), 1.86 (tt, $J$ = 10.6, 4.6 Hz, 4H).

**$^{13}$C NMR** (76 MHz, $CDCl_3$) δ 153.4, 139.0, 126.0, 123.3, 121.6, 111.8, 29.6, 22.8, 22.8, 22.7.

The NMR data were in agreement with those reported in the literature.[45]

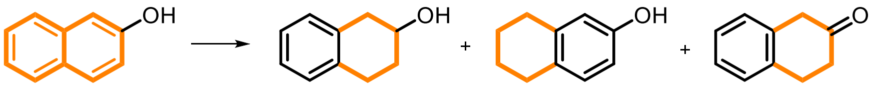

**6e**, 45% **6e'**, 20% **6e''**, 11%

1,2,3,4-Tetrahydronaphthalen-2-ol **6e**; 5,6,7,8-tetrahydronaphthalen-2-ol **6e'**; 3,4-dihydronaphthalen-2(1H)-one **6e''**. According to the **GP3**, using the alkene **5e** (0.2 mmol, 1.0 equiv.) and stirring at room temperature for 120 h under 5 W blue LED irradiation. The crude residue was purified by chromatography (pentane) to give **6e** (13.3 mg, 0.09 mmol, 45%) as a colorless liquid; **6e'** (5.9 mg, 0.040 mmol, 20%) as a colorless liquid; **6e''** (3.2 mg, 0.022 mmol, 11%) as a colorless liquid.

**$^1$H NMR** (300 MHz, $CDCl_3$, **6e**) δ 7.11 (q, $J$ = 3.1 Hz, 4H), 4.16 (dd, $J$ = 9.4, 5.5 Hz, 1H), 3.17 – 3.05 (m, 1H), 3.03 – 2.84 (m, 2H), 2.78 (dd, $J$ = 16.3, 7.7 Hz, 1H), 2.13 – 1.99 (m, 1H), 1.92 – 1.75 (m, 1H).

**$^{13}$C NMR** (101 MHz, $CDCl_3$, **6e**) δ 135.7, 134.2, 129.5, 128.6, 126.0, 125.9, 67.3, 38.4, 31.5, 27.0.

The NMR data were in agreement with those reported in the literature.[46]

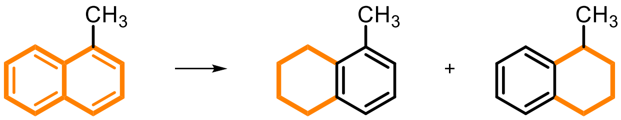

**6f**, 47% **6f'**, 23%

5-Methyl-1,2,3,4-tetrahydronaphthalene **6f**; 1-methyl-1,2,3,4-tetrahydronaphthalene **6f'**. According to the **GP3**, using the alkene **5f** (0.2 mmol, 1.0 equiv.) and stirring at room temperature for 120 h under 5 W blue LED irradiation. The crude residue was purified by chromatography (pentane) to give **6f** (13.7 mg, 0.094 mmol, 47%) as a colorless liquid; **6f'** (6.7 mg, 0.046 mmol, 23%) as a colorless liquid.

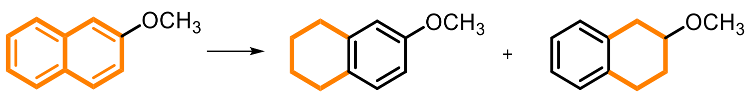


**6g**, 38%  **6g'**, 24%

6-Methoxy-1,2,3,4-tetrahydronaphthalene **6g**; 2-methoxy-1,2,3,4-tetrahydronaphthalene **6g'**; According to the **GP3**, using the alkene **5g** (0.2 mmol, 1.0 equiv.) and stirring at room temperature for 120 h under 5 W blue LED irradiation. The crude residue was purified by chromatography (pentane) to give **6g** (12.3 mg, 0.076 mmol, 38%) as a colorless liquid; **6g'** (7.8 mg, 0.048 mmol, 24%) as a colorless liquid.

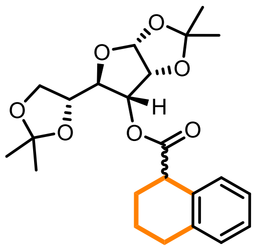

**6h**, 92%, dr 1:1

(3aR,5R,6R,6aR)-5-((R)-2,2-Dimethyl-1,3-dioxolan-4-yl)-2,2-dimethyltetrahydrofuro[2,3-d][1,3]dioxol-6-yl 1,2,3,4-tetrahydronaphthalene-1-carboxylate **6h**. According to the **GP3**, using the alkene **5h** (0.2 mmol, 1.0 equiv.) and stirring at room temperature for 72 h under 5 W blue LED irradiation. The crude residue was purified by chromatography (pentane/EtOAc 5/1) to give **6h** (76.9 mg, 0.18 mmol, 92%) as a colorless liquid.

**$^{1}$H NMR** (300 MHz, $CDCl_3$, both diastereoisomers) δ 7.25 – 7.18 (m, 1H), 7.18 – 7.07 (m, 3H), 5.83 (dd, *J* = 3.8, 1.8 Hz, 1H), 4.88 (ddd, *J* = 5.9, 3.0, 1.3 Hz, 1H), 4.84 – 4.78 (m, 1H), 4.29 (td, *J* = 6.4, 4.0 Hz, 1H), 4.11 (dt, *J* = 8.5, 3.9 Hz, 1H), 4.03 – 3.95 (m, 1H), 3.89 (q, *J* = 5.6 Hz, 1H), 3.79 – 3.65 (m, 1H), 2.91 – 2.69 (m, 2H), 2.27 – 2.12 (m, 1H), 2.11 – 1.91 (m, 2H), 1.85 – 1.68 (m, 1H), 1.51 (d, *J* = 7.1 Hz, 3H), 1.38 (d, *J* = 7.3 Hz, 3H), 1.34 (d, *J* = 5.2 Hz, 6H).

**$^{13}$C NMR** (76 MHz, $CDCl_3$, both diastereoisomers) δ 174.1, 174.0, 137.2, 137.2, 132.9, 132.9, 130.0, 129.5, 129.4, 129.3, 126.9, 126.9, 125.8, 125.6, 112.9, 112.9, 110.0, 110.0, 104.3, 104.2, 77.7, 77.6, 77.5, 75.0, 74.8, 73.0, 72.8, 65.6, 65.4, 44.8, 44.7, 29.1, 29.1, 26.8, 26.8, 26.7, 26.6, 26.6, 26.2, 26.2, 25.1, 25.0, 20.6, 20.5.

**HRMS** (ESI) m/z: $[M + Na]^+$ Calcd for $C_{23}H_{30}O_7Na$ 441.1884; found 441.1884.

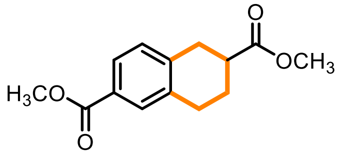


**6i**, 66%

Dimethyl 1,2,3,4-tetrahydronaphthalene-2,6-dicarboxylate **6i**. According to the **GP3**, using the alkene **5i** (0.2 mmol, 1.0 equiv.) and stirring at room temperature for 72 h under 5 W blue LED irradiation. The crude residue

was purified by chromatography (pentane/EtOAc 10/1) to give **6i** (32.7 mg, 0.13 mmol, 66%) as a colorless liquid.

**$^1$H NMR** (300 MHz, $CDCl_3$) δ 7.76 (d, *J* = 6.7 Hz, 2H), 7.16 (d, *J* = 8.3 Hz, 1H), 3.89 (s, 3H), 3.73 (s, 3H), 3.05 (d, *J* = 7.9 Hz, 2H), 2.89 (dt, *J* = 11.0, 5.7 Hz, 2H), 2.83 – 2.69 (m, 1H), 2.28 – 2.15 (m, 1H), 1.88 (qd, *J* = 10.7, 6.2 Hz, 1H).

The NMR data were in agreement with those reported in the literature.[47]

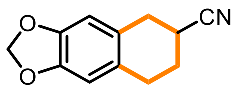


**6j**, 84%

5,6,7,8-Tetrahydronaphtho[2,3-d][1,3]dioxole-6-carbonitrile **6j**. According to the **GP3**, using the alkene **5j** (0.2 mmol, 1.0 equiv.) and stirring at room temperature for 72 h under 5 W blue LED irradiation. The crude residue was purified by chromatography (pentane/EtOAc 10/1) to give **6j** (33.8 mg, 0.17 mmol, 84%) as a colorless liquid.

**$^1$H NMR** (300 MHz, $CDCl_3$) δ 6.55 (s, 1H), 6.52 (s, 1H), 5.89 (s, 2H), 3.05 – 2.81 (m, 4H), 2.81 – 2.66 (m, 1H), 2.13 (dtd, *J* = 11.8, 6.1, 2.9 Hz, 1H), 2.07 – 1.95 (m, 1H).

**$^{13}$C NMR** (76 MHz, $CDCl_3$) δ 146.5, 146.2, 127.5, 125.0, 122.0, 108.7, 108.5, 100.9, 32.4, 27.1, 26.2, 25.6.

**HRMS** (ESI) m/z: $[M + Na]^+$ Calcd for $C_{12}H_{11}NO_2Na$ 224.0682; found 224.0682.

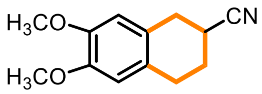


**6k**, 89%

6,7-Dimethoxy-1,2,3,4-tetrahydronaphthalene-2-carbonitrile **6k**. According to the **GP3**, using the alkene **5k** (0.2 mmol, 1.0 equiv.) and stirring at room temperature for 72 h under 5 W blue LED irradiation. The crude residue was purified by chromatography (pentane/EtOAc 10/1) to give **6k** (38.6 mg, 0.18 mmol, 89%) as a colorless liquid.

**$^1$H NMR** (300 MHz, $CDCl_3$) δ 6.57 (s, 1H), 6.54 (s, 1H), 3.84 (s, 6H), 3.06 – 2.84 (m, 4H), 2.85 – 2.71 (m, 1H), 2.21 – 2.09 (m, 1H), 2.10 – 1.94 (m, 1H).

**$^{13}$C NMR** (76 MHz, $CDCl_3$) δ 147.9, 147.6, 126.4, 124.0, 122.1, 111.6, 111.5, 56.0, 55.9, 32.0, 26.6, 26.3, 25.6.

**HRMS** (ESI) m/z: $[M + Na]^+$ Calcd for $C_{13}H_{15}NO_2Na$ 240.0995; found 240.0995.

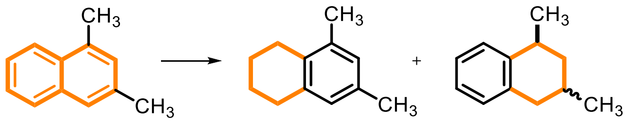


**6l**, 56% **6l'**, 26%, cis:trans 2:1

5,7-Dimethyl-1,2,3,4-tetrahydronaphthalene **6l**; 1,3-dimethyl-1,2,3,4-tetrahydronaphthalene **6l'**. According to the **GP3**, using the alkene **5l** (0.2 mmol, 1.0 equiv.) and stirring at room temperature for 120 h under 5 W blue LED irradiation. The crude residue was purified by chromatography (pentane/EtOAc 10/1) to give **6l** (17.9 mg, 0.11 mmol, 56%) as a colorless liquid; give **6l'** (8.3 mg, 0.052 mmol, 26%) as a colorless liquid along with a small amount of inseparably unreacted **5l** and 2,4,6-triisopropylbenzenethiol.

**$^1$H NMR** (300 MHz, $CDCl_3$, **6l**) δ 6.84 (s, 1H), 6.79 (s, 1H), 2.76 (t, *J* = 6.1 Hz, 2H), 2.61 (t, *J* = 6.3 Hz, 2H), 2.28 (s, 3H), 2.21 (s, 3H), 1.82 (ddddd, *J* = 13.9, 12.3, 7.9, 4.8, 2.8 Hz, 4H).

The NMR data were in agreement with those reported in the literature.[48]

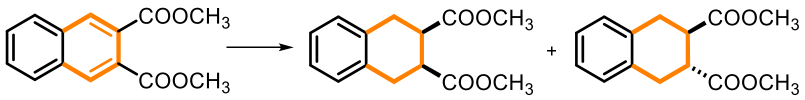


**6m**, 85% cis:trans 10:1

2-(λ$^3$-Methyl) 3-methyl 1,2,3,4-tetrahydronaphthalene-2,3-dicarboxylate ***cis*-6m**; dimethyl 1,2,3,4-tetrahydronaphthalene-2,3-dicarboxylate ***trans*-6m**. According to the **GP3**, using the alkene **5m** (0.2 mmol, 1.0 equiv.) and stirring at room temperature for 72 h under 5 W blue LED irradiation. The crude residue was purified by chromatography (pentane/EtOAc 10/1) to give **6m** (42.2 mg, 0.17 mmol, 85%) as a colorless solid with inseparable 10:1 cis: trans mixture.

**$^1$H NMR** (300 MHz, $CDCl_3$, ***cis*-6m**) δ 7.12 (s, 4H), 3.69 (s, 6H), 3.36 – 3.19 (m, 4H), 3.18 – 3.02 (m, 2H).

**$^{13}$C NMR** (76 MHz, $CDCl_3$, ***cis*-6m**) δ 173.4, 133.8, 129.0, 126.1, 52.0, 40.5, 29.5.

The NMR data were in agreement with those reported in the literature.[49]

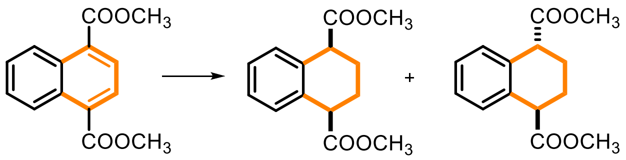


**6n**, 91% cis:trans 2:1

Dimethyl 1,2,3,4-tetrahydronaphthalene-1,4-dicarboxylate ***cis*-6n**; dimethyl (1R)-1,2,3,4-tetrahydronaphthalene-1,4-dicarboxylate ***trans*-6n**. According to the **GP3**, using the alkene **5n** (0.2 mmol, 1.0 equiv.) and stirring at room temperature for 72 h under 5 W blue LED irradiation. The crude residue was purified by chromatography (pentane/EtOAc 10/1) to give **6n** (45.1 mg, 0.17 mmol, 91%) as a colorless solid with inseparable 2:1 cis: trans mixture.

**$^{1}$H NMR** (300 MHz, $CDCl_3$, ***cis*-6n** and ***trans*-6n**) δ 7.19 – 7.10 (m, 4H), 3.83 – 3.78 (m, 1H), 3.75 (t, *J* = 6.1 Hz, 1H), 3.64 (s, 6H), 2.33 – 2.19 (m, 1H), 2.19 – 2.09 (m, 1H), 2.05 (ddq, *J* = 9.1, 7.3, 2.3 Hz, 1H), 1.96 – 1.84 (m, 1H).

**$^{13}$C NMR** (76 MHz, $CDCl_3$, ***cis*-6n** and ***trans*-6n**) δ 174.9, 174.6, 133.4, 133.4, 129.8, 129.7, 127.2, 127.2, 52.2, 52.1, 44.7, 43.9, 24.1, 23.4.

**HRMS** (ESI) m/z: $[M + Na]^+$ Calcd for $C_{14}H_{16}O_4Na$ 271.0941; found 271.0941.

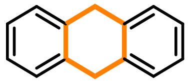

**6o**, 70%

9,10-Dihydroanthracene **6o**. According to the **GP3**, using the alkene **5o** (0.2 mmol, 1.0 equiv.) and stirring at room temperature for 72 h under 5 W blue LED irradiation. The crude residue was purified by chromatography (pentane) to give **6o** (25.2 mg, 0.14 mmol, 70%) as a colorless solid.

**$^{1}$H NMR** (300 MHz, $CDCl_3$) δ 7.43 – 7.31 (m, 4H), 7.32 – 7.15 (m, 4H), 4.00 (s, 4H).

**$^{13}$C NMR** (76 MHz, $CDCl_3$) δ 136.7, 127.4, 126.1, 36.2.

The NMR data were in agreement with those reported in the literature.[50]

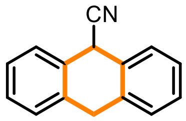


**6p**, 69%

9,10-Dihydroanthracene-9-carbonitrile **6p**. According to the **GP3**, using the alkene **5p** (0.2 mmol, 1.0 equiv.) and stirring at room temperature for 72 h under 5 W blue LED irradiation. The crude residue was purified by chromatography (pentane) to give **6p** (28.3 mg, 0.14 mmol, 69%) as a colorless solid.

**$^{1}$H NMR** (400 MHz, $CDCl_3$) δ 7.63 (ddd, *J* = 4.5, 3.7, 1.6 Hz, 2H), 7.40 – 7.30 (m, 6H), 5.04 (dd, *J* = 2.4, 1.1 Hz, 1H), 4.10 (d, *J* = 17.9 Hz, 1H), 3.93 (dd, *J* = 17.9, 2.4 Hz, 1H).

**$^{13}$C NMR** (101 MHz, $CDCl_3$) δ 136.1, 130.8, 128.3, 128.0, 127.1, 126.6, 118.3, 37.1, 35.6.

The NMR data were in agreement with those reported in the literature.[51]

## Synthesis and characterization of products 6q-u

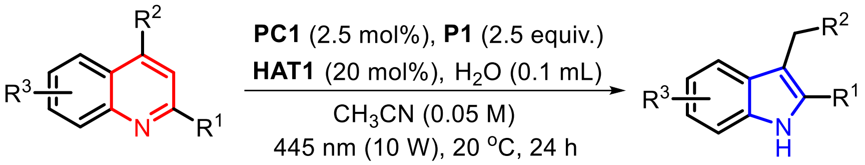


5q-u 6q-u

**General procedure (GP4) for the skeletal editing of quinoline derivates:** To an oven dried Schlenk tube with a magnetic stirring bar, **P1** (0.5 mmol, 2.5 equiv.), **PC1** (0.005 mmol, 2.5 mol%), deionized water (0.10 mL) and acetonitrile (4.0 mL) were added under argon atmosphere using standard Schlenk techniques at ambient temperature. Then, the quinoline **5q-u** (0.2 mmol, 1.0 equiv.) and the HAT catalyst **HAT1** (0.04 mmol, 0.2 equiv.) were added under argon atmosphere. The tube was sealed and placed in the photoreactor, and then irradiated with a 10 W 445 nm LED at 20 °C using the standard set-up. After 24 hours, the irradiation was stopped. The volatiles were removed in vacuo, then the crude residue was purified by flash column chromatography on silica (pentane, pentane/EtOAc or pentane/$Et_2O$ mixtures, as detailed in the individual entries), to afford the corresponding product **6q-u**.

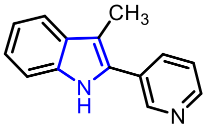


**6q**, 61%

3-Methyl-2-(pyridin-3-yl)-1*H*-indole **6q**. According to the **GP4**, using the alkene **5q** (0.2 mmol, 1.0 equiv.) and stirring at room temperature for 24 h under 10 W blue LED irradiation. The crude residue was purified by chromatography (pentane/EtOAc 1/1 to EtOAc) to give **6q** (25.4 mg, 0.12 mmol, 61%) as a yellow solid.

**$^1$H NMR** (300 MHz, $CDCl_3$) δ 8.82 (d, *J* = 2.3 Hz, 1H), 8.58 – 8.45 (m, 2H), 7.84 (dt, *J* = 7.9, 2.0 Hz, 1H), 7.57 (dd, *J* = 7.5, 1.4 Hz, 1H), 7.38 – 7.30 (m, 2H), 7.23 – 7.18 (m, 1H), 7.17 – 7.08 (m, 1H), 2.42 (s, 3H).

**$^{13}$C NMR** (76 MHz, $CDCl_3$) δ 148.6, 148.0, 136.3, 134.9, 130.6, 129.7, 129.5, 123.7, 122.9, 119.8, 119.2, 111.0, 110.3, 9.6.

The NMR data were in agreement with those reported in the literature.[52]

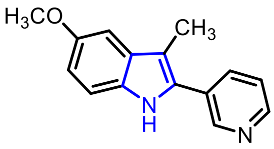


**6r**, 46%

5-Methoxy-3-methyl-2-(pyridin-3-yl)-1*H*-indole **6r**. According to the **GP4**, using the alkene **5r** (0.2 mmol, 1.0 equiv.) and stirring at room temperature for 72 h under 10 W blue LED irradiation. The crude residue was

purified by chromatography (pentane/EtOAc 1/1 to EtOAc) to give **6r** (21.9 mg, 0.09 mmol, 46%) as a white solid along with a small amount of inseparable unidentified byproduct.

**$^1$H NMR** (300 MHz, $CDCl_3$) δ 8.85 (d, *J* = 2.3 Hz, 1H), 8.58 (dd, *J* = 4.9, 1.6 Hz, 1H), 8.20 (s, 1H), 7.87 (dt, *J* = 7.9, 2.0 Hz, 1H), 7.40 (dd, *J* = 7.9, 4.8 Hz, 1H), 7.29 (d, *J* = 8.8 Hz, 1H), 7.04 (d, *J* = 2.4 Hz, 1H), 6.90 (dt, *J* = 8.9, 2.5 Hz, 1H), 3.90 (s, 3H), 2.44 (s, 3H).

**$^{13}$C NMR** (76 MHz, $CDCl_3$) δ 154.3, 148.6, 148.1, 146.3, 134.8, 131.5, 130.2, 129.5, 123.6, 113.2, 111.7, 110.1, 100.8, 55.9, 9.7.

**HRMS** (ESI) m/z: $[M + H]^+$ Calcd for $C_{15}H_{15}N_2O$ 239.1179; found 239.1179.

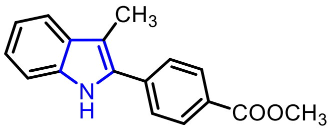


**6s**

Methyl 4-(3-methyl-1*H*-indol-2-yl)benzoate **6s**. According to the **GP4**, using the alkene **5s** (0.2 mmol, 1.0 equiv.) and stirring at room temperature for 24 h under 10 W blue LED irradiation. The crude residue was purified by chromatography (pentane/EtOAc 10/1) to give **6s** (27.0 mg, 0.10 mmol, 51%) as a white solid.

**$^1$H NMR** (300 MHz, $CDCl_3$) δ 8.19 – 8.08 (m, 3H), 7.69 – 7.60 (m, 3H), 7.39 (dt, *J* = 8.0, 1.0 Hz, 1H), 7.29 – 7.22 (m, 1H), 7.20 – 7.12 (m, 1H), 3.96 (s, 3H), 2.51 (s, 3H).

**$^{13}$C NMR** (76 MHz, $CDCl_3$) δ 166.9, 137.8, 136.2, 132.8, 130.1, 130.0, 128.5, 127.2, 123.1, 119.8, 119.3, 110.9, 110.6, 52.2, 9.9.

The NMR data were in agreement with those reported in the literature.[53]

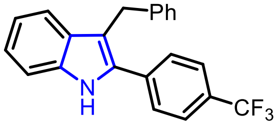


**6t**, 52%

3-Benzyl-2-(4-(trifluoromethyl)phenyl)-1*H*-indole **6t**. According to the **GP4**, using the alkene **5t** (0.2 mmol, 1.0 equiv.) and stirring at room temperature for 72 h under 5 W blue LED (455 nm) irradiation. The crude residue was purified by chromatography (pentane/EtOAc 10/1) to give **6t** (36.5 mg, 0.10 mmol, 52%) as a yellow solid.

**$^1$H NMR** (400 MHz, $CDCl_3$) δ 8.09 (s, 1H), 7.58 (d, *J* = 8.2 Hz, 2H), 7.52 (d, *J* = 8.2 Hz, 2H), 7.39 (d, *J* = 7.9 Hz, 1H), 7.34 (d, *J* = 8.1 Hz, 1H), 7.22 – 7.08 (m, 6H), 7.02 (ddd, *J* = 8.0, 7.0, 1.0 Hz, 1H), 4.20 (s, 2H).

**$^{19}$F NMR** (376 MHz, $CDCl_3$) δ -62.49.

**$^{13}$C NMR** (101 MHz, $CDCl_3$) δ 141.0, 136.4, 136.4, 136.3, 133.7, 129.5, 128.6, 128.1, 127.9, 126.0, 125.9 (q, *J* = 3.8 Hz), 124.1 (q, *J* = 272.1 Hz), 123.2, 120.2, 119.9, 112.7, 111.0, 30.4.

**HRMS** (ESI) m/z: $[M - H]^-$ Calcd for $C_{22}H_{15}F_3N$ 350.1162; found 350.1162.

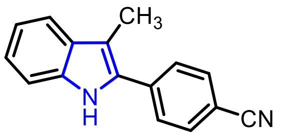


**6u**, 36%

4-(3-Methyl-1*H*-indol-2-yl)benzonitrile **6u**. According to the **GP4**, using the alkene **5u** (0.2 mmol, 1.0 equiv.) and stirring at room temperature for 24 h under 10 W blue LED irradiation. The crude residue was purified by chromatography (pentane/EtOAc 10/1) to give **6u** (16.7 mg, 0.07 mmol, 36%) as a white solid.

**$^1$H NMR** (300 MHz, $CDCl_3$) δ 8.07 (s, 1H), 7.76 (d, *J* = 8.7 Hz, 2H), 7.68 (d, *J* = 8.6 Hz, 2H), 7.63 (dd, *J* = 7.8, 1.2 Hz, 1H), 7.40 (dt, *J* = 8.0, 1.0 Hz, 1H), 7.30 – 7.24 (m, 1H), 7.18 (ddd, *J* = 8.0, 7.0, 1.1 Hz, 1H), 2.50 (s, 3H).

**$^{13}$C NMR** (76 MHz, $CDCl_3$) δ 137.8, 136.4, 132.6, 131.8, 129.9, 127.8, 123.6, 120.1, 119.5, 118.9, 111.5, 111.0, 110.4, 10.0.

The NMR data were in agreement with those reported in the literature.[54]

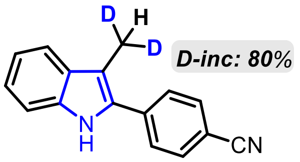


**6u-D$_2$**, 20%

4-(3-(Methyl-*d*$_2$)-1*H*-indol-2-yl)benzonitrile **6u-D$_2$**. According to the **GP4**, using the alkene **5u** (0.2 mmol, 1.0 equiv.) and stirring at room temperature for 24 h under 10 W blue LED irradiation. The crude residue was purified by chromatography (pentane/EtOAc 10/1) to give **6u-D$_2$** (9.4 mg, 0.04 mmol, 20%) as a white solid.

**$^1$H NMR** (300 MHz, $CDCl_3$) δ 8.14 (s, 1H), 7.75 (d, *J* = 8.5 Hz, 2H), 7.68 (d, *J* = 8.7 Hz, 2H), 7.65 – 7.61 (m, 1H), 7.39 (dt, *J* = 8.1, 0.9 Hz, 1H), 7.30 – 7.23 (m, 1H), 7.17 (ddd, *J* = 8.0, 7.0, 1.1 Hz, 1H), 2.47 (tt, *J* = 4.4, 2.4 Hz, 1H, 2D).

**HRMS** (ESI) m/z: $[M + Na]^+$ Calcd for $C_{16}H_{10}D_2N_2Na$ 257.1018; found 257.1018.

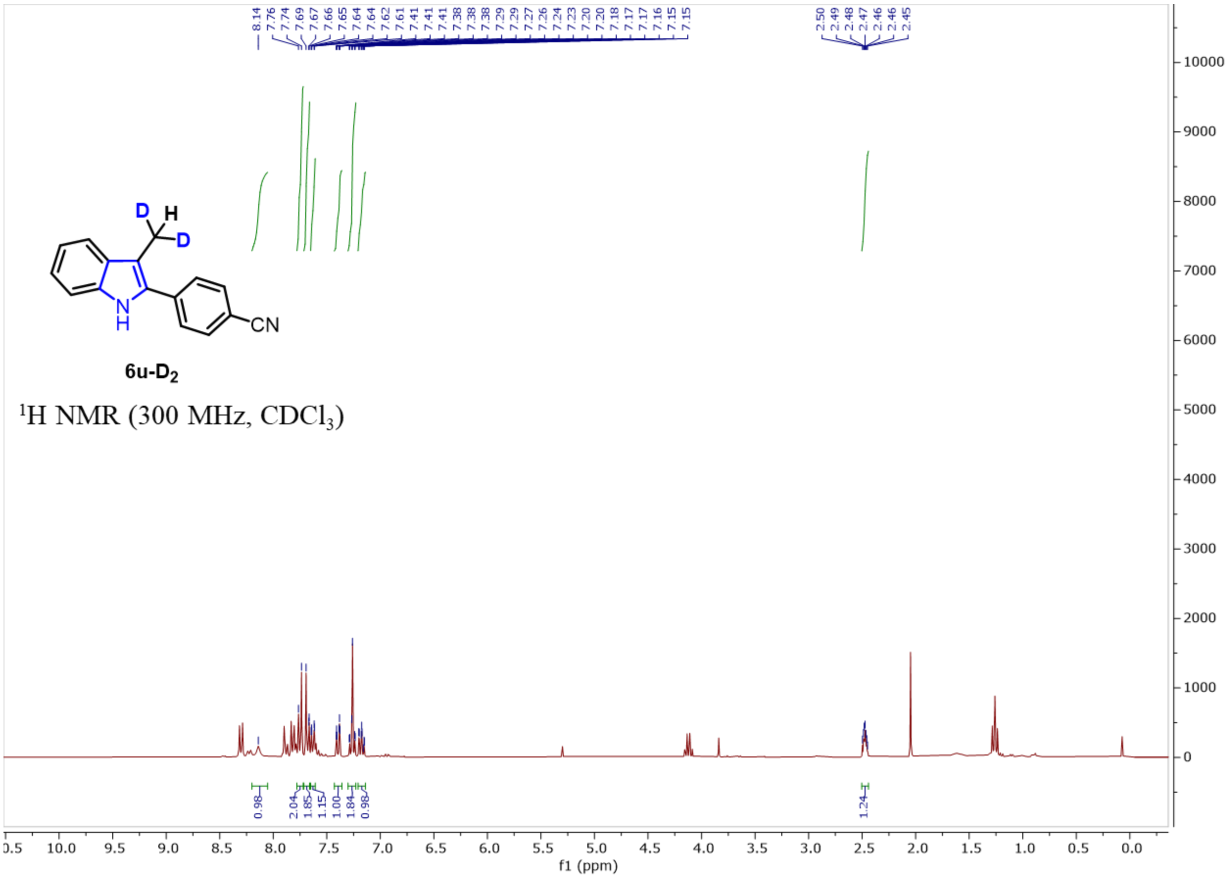


The amount of D-incorporation was determined by HR-MS (ESI) analysis:

WWU MÜNSTER

Organisch-Chemisches Institut
Abteilung Massenspektrometrie
Corrensstraße 40
48149 Münster

0251-83-33299

# LabelChecker Results

Formula: C16 H12 N2 Na
Mass (monoisotopic): 255,09

Difference Value: 0,000340
Error Sum: 0,018
Error (%): 0,201

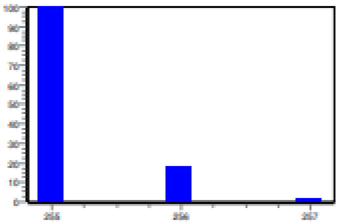

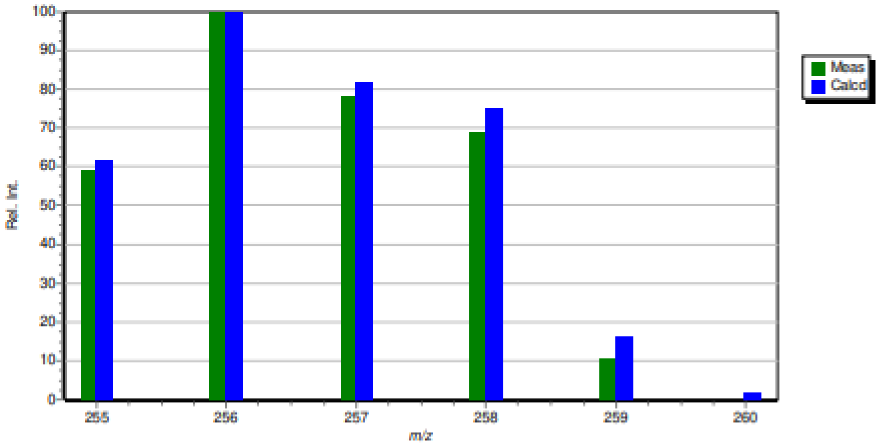


Deuterium: 0-fold [%]: 69,58 21,93
Deuterium: 1-fold [%]: 100,00 31,52
Deuterium: 2-fold [%]: 72,93 22,99
Deuterium: 3-fold [%]: 69,80 22,00
Deuterium: 4-fold [%]: 4,89 1,54
Deuterium: 5-fold [%]: 0,00 0,00
Deuterium: 6-fold [%]: 0,07 0,02
Label Atom Sum: 1,50 (12,48%)

Isotope list used for fitting data:

m/z intensity
255,08 2757614
255,12 12342
255,16 98728
255,19 15286
256,09 4669050
257,07 38059
257,10 3662020
257,14 120862
258,11 290223
258,28 3217597
259,08 239612
259,11 55004
259,28 498750

*Citation of UMC software in scientific publications: The computer software UMC (Universal Mass Calculator) was used to (e.g. simulate isotope patterns, evaluate the degree of deuteration/labelling, evaluate elemental compositions). UMC Version [illegible], Dr. Matthias C. Letzel, WWU Münster, Org.-Chem. Institut, Germany. https://www.uni-muenster.de/Chemie.oc/ms/downloads.html*

## 5. Radical cyclization and ring-opening experiments

### 5.1 Radical cyclization experiments

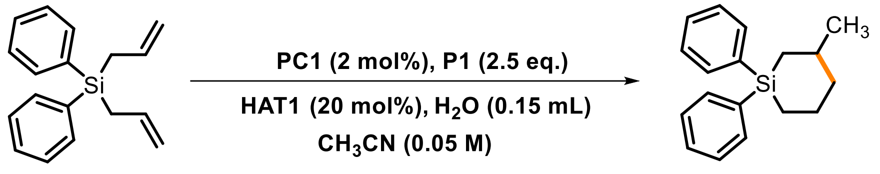


**7** **8**, 91%

To an oven dried Schlenk tube with a magnetic stirring bar, **P1** (0.5 mmol, 2.5 equiv.), **PC1** (0.004 mmol, 2 mol%), deionized water (0.15 mL) and acetonitrile (4.0 mL) were added under argon atmosphere using standard Schlenk techniques at ambient temperature. Then, the substrate **7** (0.2 mmol, 1.0 equiv.) and the HAT catalyst **HAT1** (0.04 mmol, 0.2 equiv.) were added under argon atmosphere. The tube was sealed and placed in the photoreactor, and then irradiated with a 5 W 455 nm LED at 20 $^{o}$C using the standard set-up. After 72 hours, the irradiation was stopped. The volatiles were removed in vacuo, then the crude residue was purified by flash column chromatography on silica (pentane) to afford the product **8** (48.4 mg, 0.18 mmol, 91%) as a colorless liquid.

**$^{1}$H NMR** (400 MHz, $CDCl_3$) δ 7.67 – 7.60 (m, 2H), 7.48 – 7.43 (m, 2H), 7.43 – 7.37 (m, 3H), 7.35 – 7.29 (m, 3H), 2.15 – 2.03 (m, 1H), 1.71 (tdt, *J* = 13.5, 6.2, 2.9 Hz, 2H), 1.58 – 1.48 (m, 1H), 1.48 – 1.35 (m, 2H), 1.05 (d, *J* = 6.4 Hz, 4H), 0.90 (td, *J* = 14.4, 5.3 Hz, 1H), 0.74 (dd, *J* = 14.5, 12.5 Hz, 1H).

**$^{13}$C NMR** (101 MHz, $CDCl_3$) δ 138.0, 135.8, 134.8, 134.3, 129.2, 129.1, 128.0, 127.8, 38.4, 31.5, 27.7, 23.7, 20.9, 10.7.

The NMR data were in agreement with those reported in the literature.[55]

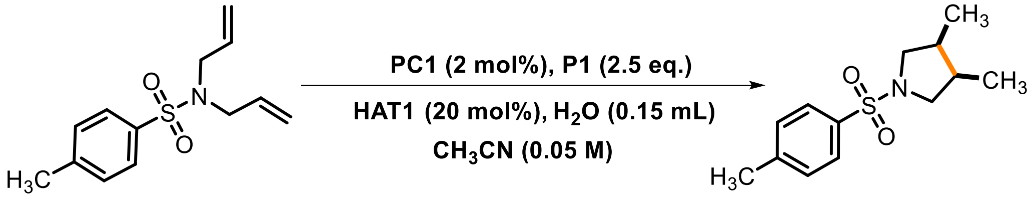


**9** **10**, 62%, cis:trans 12:1

To an oven dried Schlenk tube with a magnetic stirring bar, **P1** (0.5 mmol, 2.5 equiv.), **PC1** (0.004 mmol, 2 mol%), deionized water (0.15 mL) and acetonitrile (4.0 mL) were added under argon atmosphere using standard Schlenk techniques at ambient temperature. Then, the substrate **9** (0.2 mmol, 1.0 equiv.) and the HAT catalyst **HAT1** (0.04 mmol, 0.2 equiv.) were added under argon atmosphere. The tube was sealed and placed in the photoreactor, and then irradiated with a 5 W 455 nm LED at 20 $^{o}$C using the standard set-up. After 72 hours, the irradiation was stopped. The volatiles were removed in vacuo, then the crude residue was purified by flash

column chromatography on silica (pentane/EtOAc 5/1) to afford the product **10** (31.4 mg, 0.12 mmol, 62%) as a colorless solid with inseparable 12:1 cis: trans mixture.

**$^{1}$H NMR** (400 MHz, $CDCl_3$, ***cis*-10**) δ 7.71 (d, *J* = 8.3 Hz, 2H), 7.31 (d, *J* = 8.0 Hz, 2H), 3.37 (dd, *J* = 9.7, 6.7 Hz, 2H), 2.95 (dd, *J* = 9.7, 5.7 Hz, 2H), 2.43 (s, 3H), 2.19 – 2.05 (m, 2H), 0.76 (d, *J* = 6.7 Hz, 6H).

**$^{13}$C NMR** (101 MHz, $CDCl_3$, ***cis*-10**) δ 143.2, 134.3, 129.5, 127.4, 53.9, 36.2, 21.5, 12.9.

The NMR data were in agreement with those reported in the literature.[56]

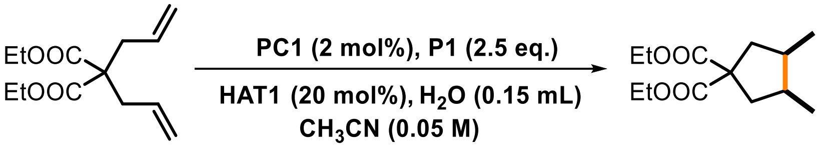


**11** **12**, 87%, cis:trans 9:1

To an oven dried Schlenk tube with a magnetic stirring bar, **P1** (0.5 mmol, 2.5 equiv.), **PC1** (0.004 mmol, 2 mol%), deionized water (0.15 mL) and acetonitrile (4.0 mL) were added under argon atmosphere using standard Schlenk techniques at ambient temperature. Then, the substrate **11** (0.2 mmol, 1.0 equiv.) and the HAT catalyst **HAT1** (0.04 mmol, 0.2 equiv.) were added under argon atmosphere. The tube was sealed and placed in the photoreactor, and then irradiated with a 5 W 455 nm LED at 20 $^{o}$C using the standard set-up. After 72 hours, the irradiation was stopped. The volatiles were removed in vacuo, then the crude residue was purified by flash column chromatography on silica (pentane/EtOAc 50/1) to afford the product **12** (42.1 mg, 0.17 mmol, 87%) as a colorless liquid with inseparable 9:1 cis: trans mixture.

**$^{1}$H NMR** (300 MHz, $CDCl_3$, ***cis*-12**) δ 4.15 (qd, *J* = 7.1, 1.3 Hz, 4H), 2.37 (dd, *J* = 13.3, 6.8 Hz, 2H), 2.14 – 2.00 (m, 2H), 1.93 (dd, *J* = 13.5, 6.9 Hz, 2H), 1.22 (t, *J* = 7.2 Hz, 6H), 0.85 (d, *J* = 6.7 Hz, 6H).

**$^{13}$C NMR** (76 MHz, $CDCl_3$, ***cis*-12**) δ 173.1, 172.9, 61.2, 61.2, 59.2, 41.0, 36.8, 14.9, 14.0.

The NMR spectra data were in agreement with those reported in the literature.[57]

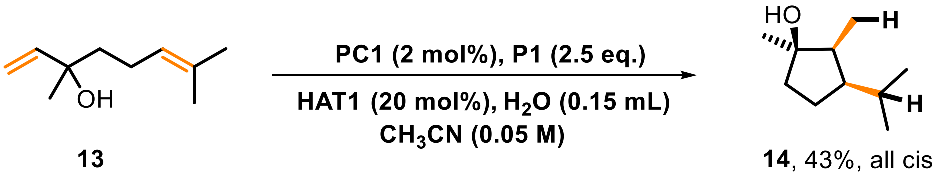


**13** **14**, 43%, all cis

To an oven dried Schlenk tube with a magnetic stirring bar, **P1** (0.5 mmol, 2.5 equiv.), **PC1** (0.004 mmol, 2 mol%), deionized water (0.15 mL) and acetonitrile (4.0 mL) were added under argon atmosphere using standard Schlenk techniques at ambient temperature. Then, the substrate **13** (0.2 mmol, 1.0 equiv.) and the HAT catalyst **HAT1** (0.04 mmol, 0.2 equiv.) were added under argon atmosphere. The tube was sealed and placed in the photoreactor, and then irradiated with a 5 W 455 nm LED at 20 $^{o}$C using the standard set-up. After 120 hours, the irradiation was stopped. The volatiles were removed in vacuo, then the crude residue was purified by flash

column chromatography on silica (pentane/EtOAc 50/1) to afford the product **14** (13.4 mg, 0.086 mmol, 43%) as a colorless liquid.

**$^1$H NMR** (400 MHz, $CDCl_3$) δ 1.81 – 1.70 (m, 2H), 1.70 – 1.64 (m, 2H), 1.62 – 1.52 (m, 2H), 1.51 – 1.34 (m, 2H), 1.32 (s, 3H), 0.88 (d, *J* = 6.4 Hz, 3H), 0.85 (dd, *J* = 6.8, 1.8 Hz, 6H).

**$^{13}$C NMR** (126 MHz, $CDCl_3$) δ 80.4, 49.2, 45.2, 37.8, 30.0, 29.8, 25.6, 21.9, 21.4, 8.5.

**HRMS** (ESI) m/z: $[M + Na]^+$ Calcd for $C_{10}H_{20}ONa$ 179.1406; found 179.1406.

## 5.2 Cycle-opening experiment

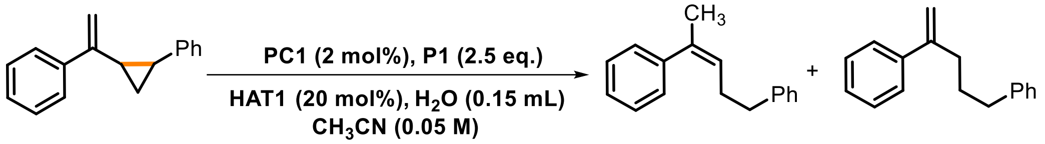


**15** **16**, 64% *Z/E* 17/1 **17**, 13%

To an oven dried Schlenk tube with a magnetic stirring bar, **P3** (0.5 mmol, 2.5 equiv.), **PC1** (0.004 mmol, 2 mol%), deionized water (0.15 mL) and acetonitrile (4.0 mL) were added under argon atmosphere using standard Schlenk techniques at ambient temperature. Then, the substrate **15** (0.2 mmol, 1.0 equiv.) and the HAT catalyst **HAT1** (0.02 mmol, 0.1 equiv.) were added under argon atmosphere. The tube was sealed and placed in the photoreactor, and then irradiated with a 5 W 455 nm LED at 20 $^o$C using the standard set-up. After 72 hours, the irradiation was stopped. The volatiles were removed in vacuo, then the crude residue was purified by flash column chromatography on silica (pentane to pentane/EtOAc 50/1) to afford the product **16** (28.4 mg, 0.13 mmol, 64%) as a colorless liquid along with a small amount of **17**[58] (13% yield, inseparable).

**$^1$H NMR** (300 MHz, $CDCl_3$, ***Z*-16**) δ 7.24 – 7.12 (m, 6H), 7.02 (d, *J* = 7.6 Hz, 4H), 5.42 (t, *J* = 7.4 Hz, 1H), 2.56 (t, *J* = 7.8 Hz, 2H), 2.21 (q, *J* = 7.7 Hz, 2H), 1.94 (s, 3H).

The NMR spectra data were in agreement with those reported in the literature.[59]

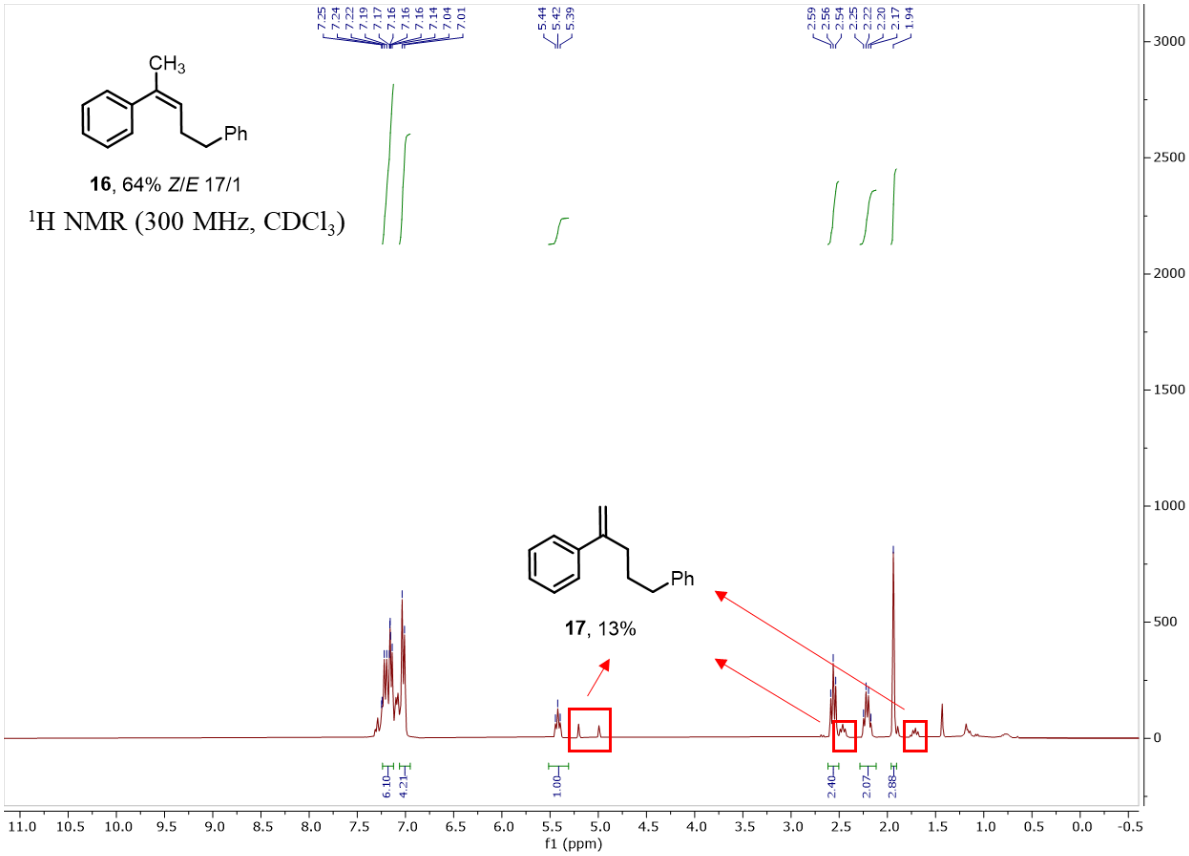

16, 64% Z/E 17/1
¹H NMR (300 MHz, CDCl3)
17, 13%
f1 (ppm)

## 6. Deuteration experiments with $CD_3CN$ and $D_2O$ as hydrogen source

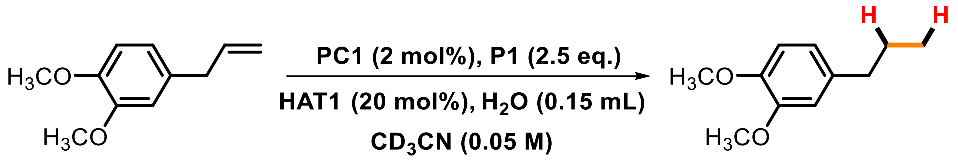


**1d** **2d**, 90%

To an oven dried Schlenk tube with a magnetic stirring bar, **P1** (0.5 mmol, 2.5 equiv.), **PC1** (0.004 mmol, 2 mol%), deionized water (0.15 mL) and $CD_3CN$ (4.0 mL) were added under argon atmosphere using standard Schlenk techniques at ambient temperature. Then, the substrate **1d** (0.2 mmol, 1.0 equiv.) and the HAT catalyst **HAT1** (0.04 mmol, 0.2 equiv.) were added under argon atmosphere. The tube was sealed and placed in the photoreactor, and then irradiated with a 5 W 455 nm LED at 20 °C using the standard set-up. After 72 hours, the irradiation was stopped. The volatiles were removed in vacuo, then the crude residue was purified by flash column chromatography on silica (pentane to pentane/EtOAc 10/1) to afford the product **2d** (32.4 mg, 0.18 mmol, 90%) as a colorless liquid.

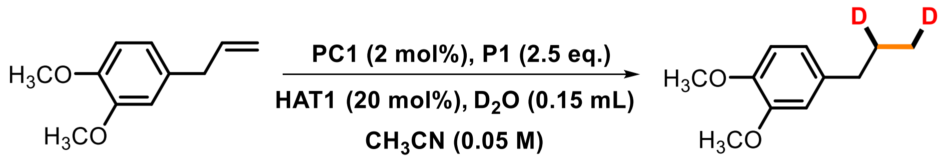


**1d** **2d-D²**, 86%

To an oven dried Schlenk tube with a magnetic stirring bar, **P1** (0.5 mmol, 2.5 equiv.), **PC1** (0.004 mmol, 2 mol%), $D_2O$ (0.15 mL) and acetonitrile (4.0 mL) were added under argon atmosphere using standard Schlenk techniques at ambient temperature. Then, the substrate **1d** (0.2 mmol, 1.0 equiv.) and the HAT catalyst **HAT1** (0.04 mmol, 0.2 equiv.) were added under argon atmosphere. The tube was sealed and placed in the photoreactor, and then irradiated with a 5 W 455 nm LED at 20 °C using the standard set-up. After 120 hours, the irradiation was stopped. The volatiles were removed in vacuo, then the crude residue was purified by flash column chromatography on silica (pentane to pentane/EtOAc 10/1) to afford the product **2d-D²** (31.3 mg, 0.17 mmol, 86%) as a colorless liquid.

**$^1$H NMR** (300 MHz, $CDCl_3$) δ 6.72 (d, $J$ = 8.6 Hz, 1H), 6.64 (dq, $J$ = 3.1, 1.9 Hz, 2H), 3.80 (s, 3H), 3.78 (s, 3H), 2.44 (s, 2H), 1.58 – 1.49 (m, 1H, 1D), 0.89 – 0.77 (m, 2H, 1D).

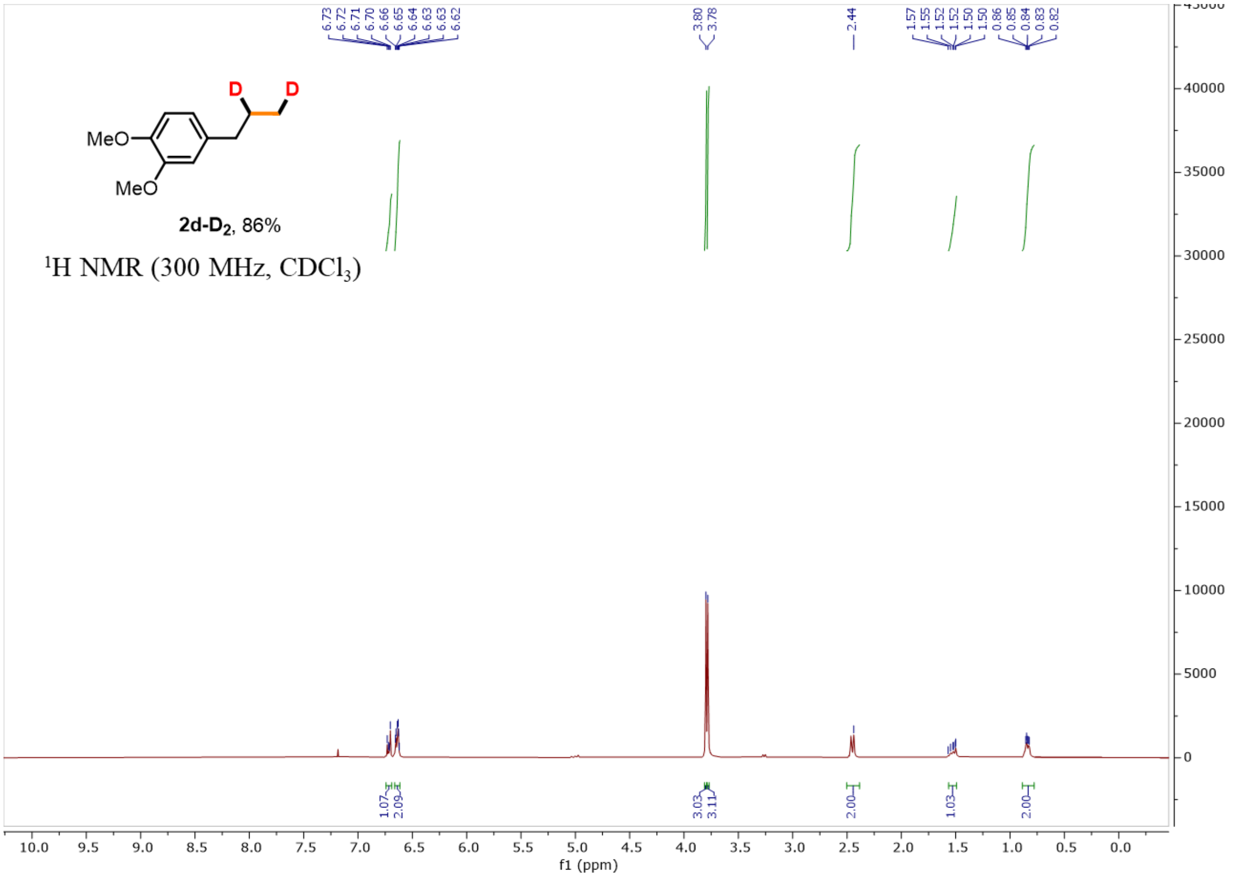


The amount of D-incorporation was determined by HR-MS (ESI) analysis:

Organisch-Chemisches Institut
Abteilung Massenspektrometrie
Corrensstraße 40
48149 Münster

0251-83-33 299

# LabelChecker Results

Formula: C11 H16 O2 Na
Mass (monoisotopic): 203,10

Difference Value: 0,000115
Error Sum: 0,011
Error (%): 0,329

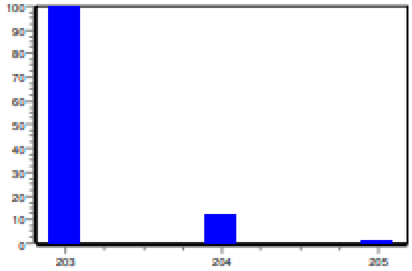


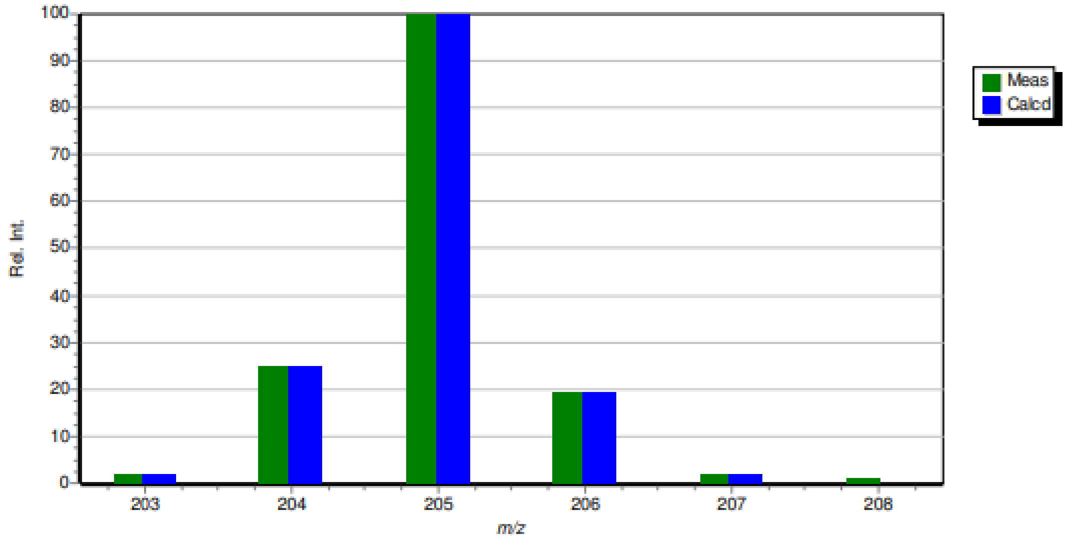


Deuterium: 0-fold (%): 1,77 1,32
Deuterium: 1-fold (%): 25,15 18,72
Deuterium: 2-fold (%): 100,00 74,42
Deuterium: 3-fold (%): 7,45 5,54
Label Atom Sum: 1,84 (11,51%)

Isotope List used for fitting data:

m/z intensity
203,10 12737867
203,22 665125
204,11 192425008
205,12 781893591
205,19 61842
206,12 150806224
207,13 13015988
208,13 9254309

*Citation of UMC software in scientific publications: The computer software UMC (Universal Mass Calculator) was used to (e.g. simulate isotope patterns, evaluate the degree of deuteration/labelling, evaluate elemental compositions). UMC Version 3.11.0.71, Dr. Matthias C. Letzel, WWU Münster, Org.-Chem. Institut, Germany. https://www.uni-muenster.de/Chemie.oc/ms/downloads.html*

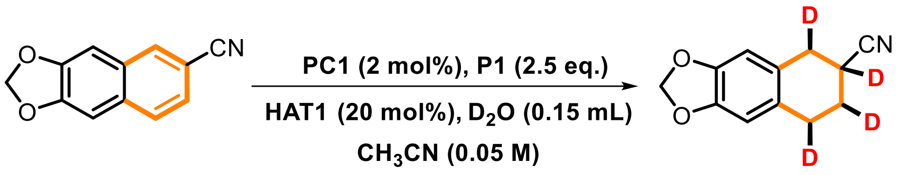


**5j** **6j-D$_4$**, 85%

To an oven dried Schlenk tube with a magnetic stirring bar, **P1** (0.5 mmol, 2.5 equiv.), **PC1** (0.004 mmol, 2 mol%), $D_2O$ (0.15 mL) and acetonitrile (4.0 mL) were added under argon atmosphere using standard Schlenk techniques at ambient temperature. Then, the substrate **5j** (0.2 mmol, 1.0 equiv.) and the HAT catalyst **HAT1** (0.04 mmol, 0.2 equiv.) were added under argon atmosphere. The tube was sealed and placed in the photoreactor, and then irradiated with a 5 W 455 nm LED at 20 ºC using the standard set-up. After 72 hours, the irradiation was stopped. The volatiles were removed in vacuo, then the crude residue was purified by flash column chromatography on silica (pentane to pentane/EtOAc 10/1) to afford the product **6j-D$_4$** (34.9 mg, 0.17 mmol, 85%) as a colorless liquid.

**$^1$H NMR** (400 MHz, $CDCl_3$) δ 6.48 (s, 1H), 6.46 (s, 1H), 5.82 (s, 2H), 2.97 – 2.87 (m, 1H, 1D), 2.86 – 2.77 (m, 1H, 1D), 2.06 – 1.92 (m, 1H, 1D).

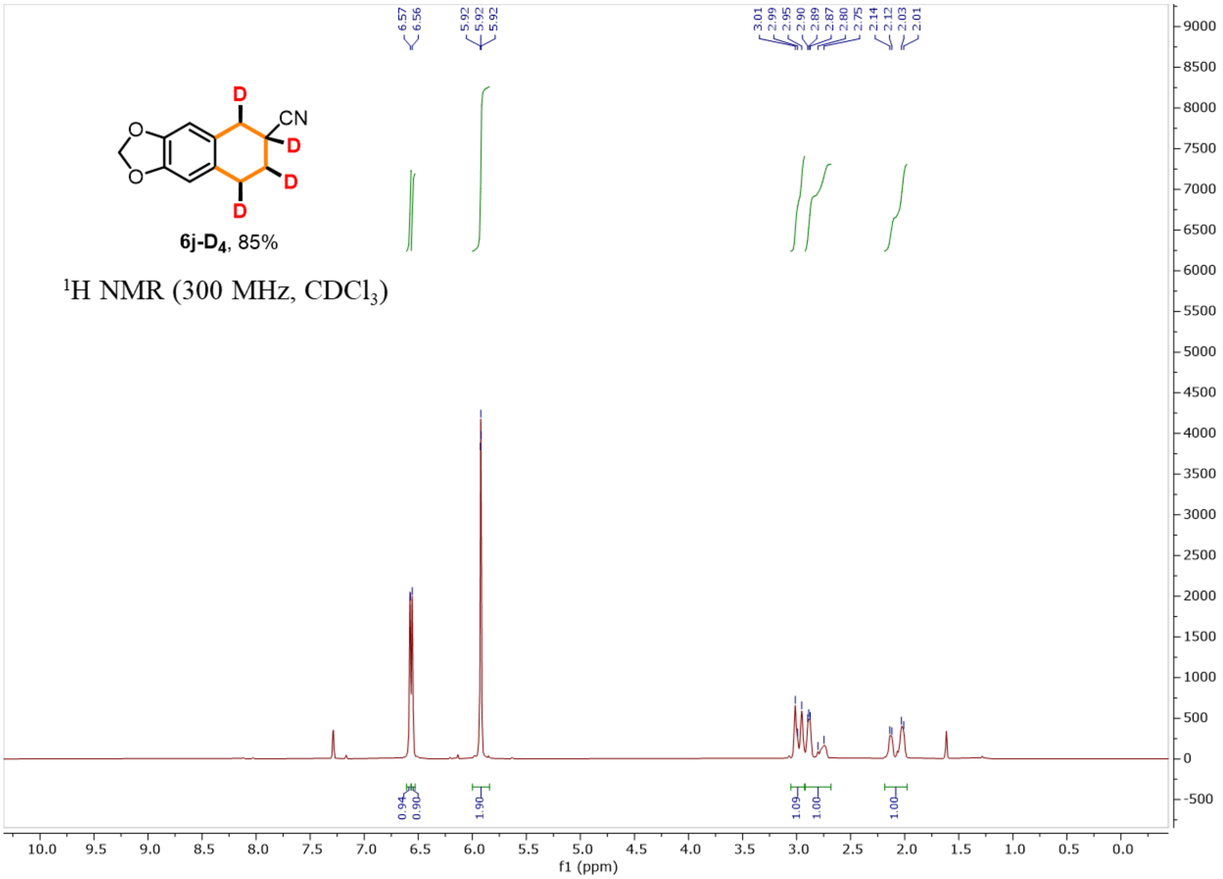

The amount of D-incorporation was determined by HR-MS (ESI) analysis:

Organisch-Chemisches Institut
Abteilung Massenspektrometrie
Corrensstraße 40
48149 Münster

0251-83-33 299

# LabelChecker Results

Formula: C12 H11 N O2 Na

Mass (monoisotopic): 224,07

Difference Value: 0,012910

Error Sum: 0,114

Error (%): 1,326

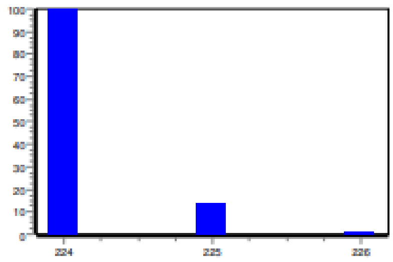


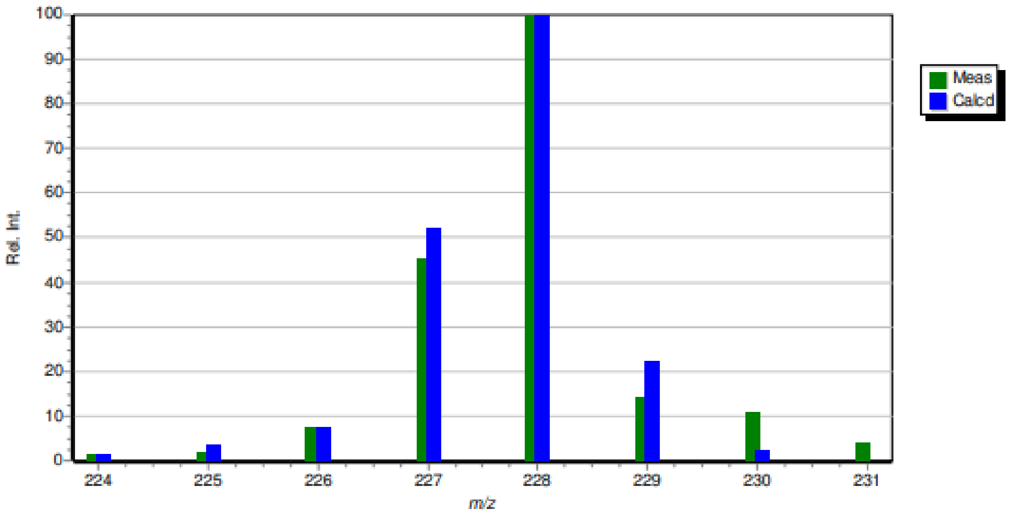


No Convergence! - Max Iterations (3000) reached!
Deuterium: 0-fold (%): 1,61 0,91
Deuterium: 1-fold (%): 3,32 1,87
Deuterium: 2-fold (%): 7,32 4,14
Deuterium: 3-fold (%): 55,07 31,12
Deuterium: 4-fold (%): 100,00 56,51
Deuterium: 5-fold (%): 9,66 5,46
Label Atom Sum: 3,57 (32,44%)

Isotope List used for fitting data:

| m/z | intensity |
|---|---|
| 224,16 | 886673 |
| 225,07 | 392530 |
| 225,11 | 1229216 |
| 225,15 | 328854 |
| 225,46 | 360504 |
| 226,08 | 4300440 |
| 227,09 | 26991076 |
| 227,12 | 3990383 |
| 228,09 | 59394864 |
| 228,13 | 362303 |
| 228,96 | 674042 |
| 229,06 | 1762049 |
| 229,10 | 8426047 |
| 229,14 | 1480510 |
| 230,10 | 776133 |
| 230,25 | 6546198 |

*Citation of UMC software in scientific publications: The computer software UMC (Universal Mass Calculator) was used to (e.g. simulate isotope patterns, evaluate the degree of deuteration/labelling, evaluate elemental compositions). UMC Version 3.11.0.71, Dr. Matthias C. Letzel, WWU Münster, Org.-Chem. Institut, Germany. https://www.uni-muenster.de/Chemie.oc/ms/downloads.html*

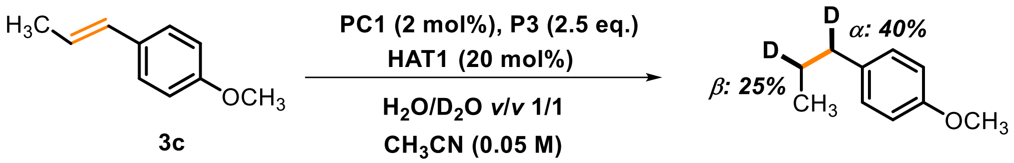


To an oven dried Schlenk tube with a magnetic stirring bar, **P3** (0.5 mmol, 2.5 equiv.), **PC1** (0.004 mmol, 2 mol%), $D_2O$ (0.10 mL), $H_2O$ (0.10 mL) and acetonitrile (4.0 mL) were added under argon atmosphere using standard Schlenk techniques at ambient temperature. Then, the substrate **3c** (0.2 mmol, 1.0 equiv.) and the HAT catalyst **HAT1** (0.04 mmol, 0.2 equiv.) were added under argon atmosphere. The tube was sealed and placed in the photoreactor, and then irradiated with a 5 W 455 nm LED at 20 °C using the standard set-up. After 72 hours, the irradiation was stopped. The volatiles were removed in vacuo, then the crude residue was purified by flash column chromatography on silica (pentane to pentane/EtOAc 10/1) to afford the product **3c-D₂** (26.7 mg, 0.18 mmol, 89%) as a colorless liquid. The deuterium ratio of *α*- and *β*-positions of the product **3c-D₂** was analyzed by $^1$H NMR spectrum.

**$^1$H NMR** (300 MHz, $CDCl_3$) δ 7.11 (d, *J* = 8.1 Hz, 2H), 6.84 (d, *J* = 8.1 Hz, 2H), 3.80 (s, 3H), 2.54 (t, *J* = 7.5 Hz, 2H), 1.63 (q, *J* = 7.5 Hz, 2H), 0.95 (t, *J* = 7.3 Hz, 3H).

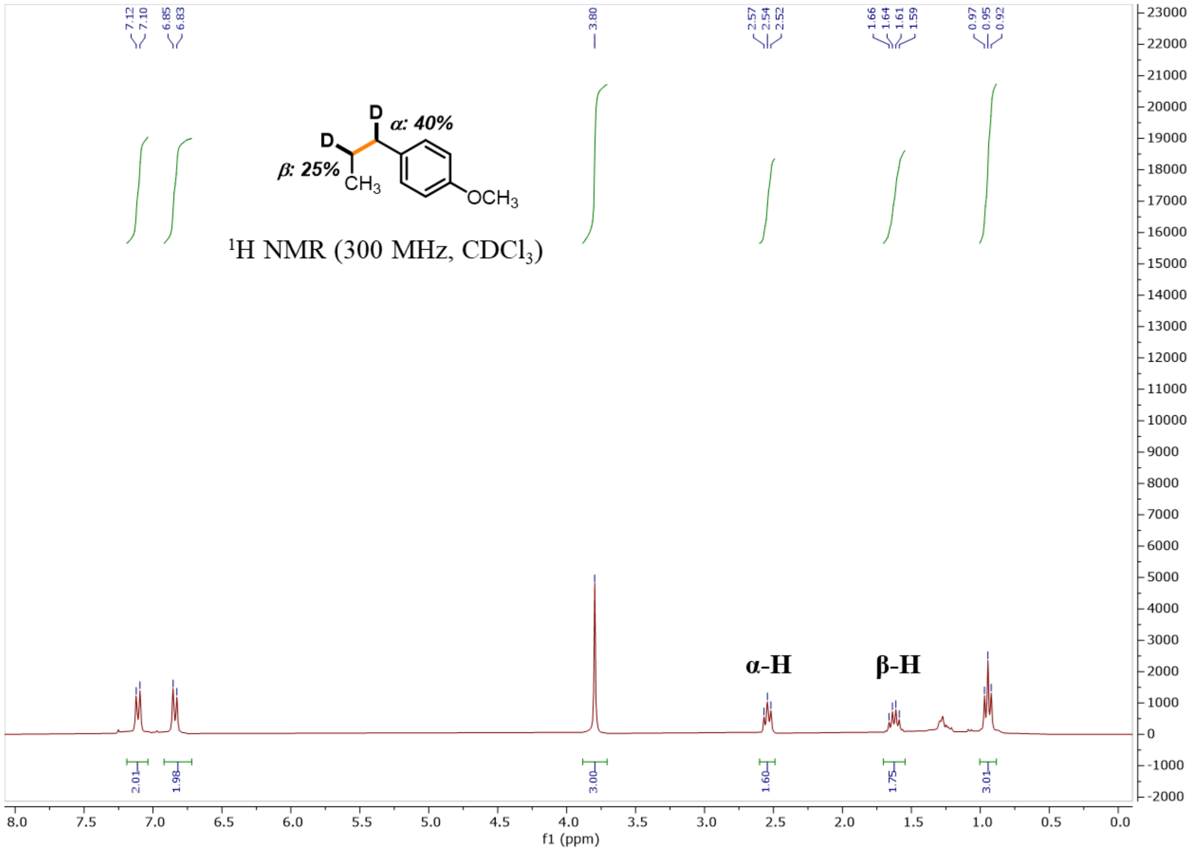

## 7. Detection of hydrogen gas $H_2$

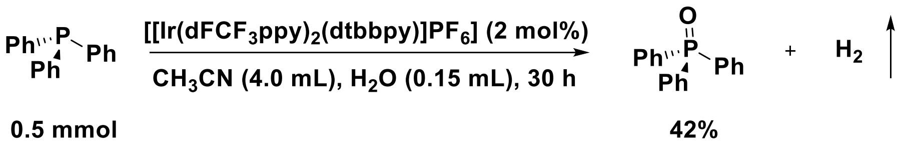

To an oven dried Schlenk tube with a magnetic stirring bar, triphenyl phosphine $PPh_3$ (0.5 mmol), **PC1** (0.01 mmol, 2 mol%), deionized water (0.15 mL) and acetonitrile (4.0 mL) were added under argon atmosphere using standard Schlenk techniques at ambient temperature. The tube was sealed and placed in the photoreactor, and then irradiated with a 5 W 455 nm LED at 20 $^{o}$C using the standard set-up. After 30 hours, the irradiation was stopped. The mixture was analyzed by GC (0.014 ± 0.00045 mmol of hydrogen gas in the gas phase). Then, 10 mL of $CDCl_3$ was added and the mixture was continued to be analyzed by $^{1}$H NMR and $^{31}$P NMR (0.21 mmol).

Comments: No hydrogen gas was detected without either $PPh_3$ or $H_2O$, indicating that both components were involved in generation of hydrogen gas.

***Detected by GC:***

***1) Method***

Table S6. The method for detection of hydrogen gas $H_2$.

| **Injektor** | | **Column** | | | **BID** | |
|---|---|---|---|---|---|---|
| Temp. | 150 °C | Total programm time | 25.50 min | | Temp | 300.0 °C |
| Injekction mode | split | Rate | Temp. | Hold Time | Sampling rate | 40 msec |
| split ratio | 50 | - | 30.0 | 4.00 | Stop Time | 25.50 min |
| carrier gas | He | 40.00 | 290.0 | 15.00 | Delay Time | 0.00 min |
| Flow control Mode | Linear Velocity | | | | | |
| Pressure | 76.1 kPa | max Temp. | 300 °C | | Flow Programm | Discharge Gas |
| Total Flow | 53.8 mL/min | Length | 30.0 m | | | Flow: 50.0 |
| Column Flow | 1.00 mL/min | ID | 0.32 mm | | | |
| Linear Velocity | 21.7 cm/sec | Film Thickness | 30 um | | | |
| Purge Flow | 3.0 mL/min | | | | | |
| | | | | | | |
| | | | | | | |
| INJ: Program | Purge Flow | | | | | |
| | Flow: 3.0 | | | | | |

***2) GC spectrum***

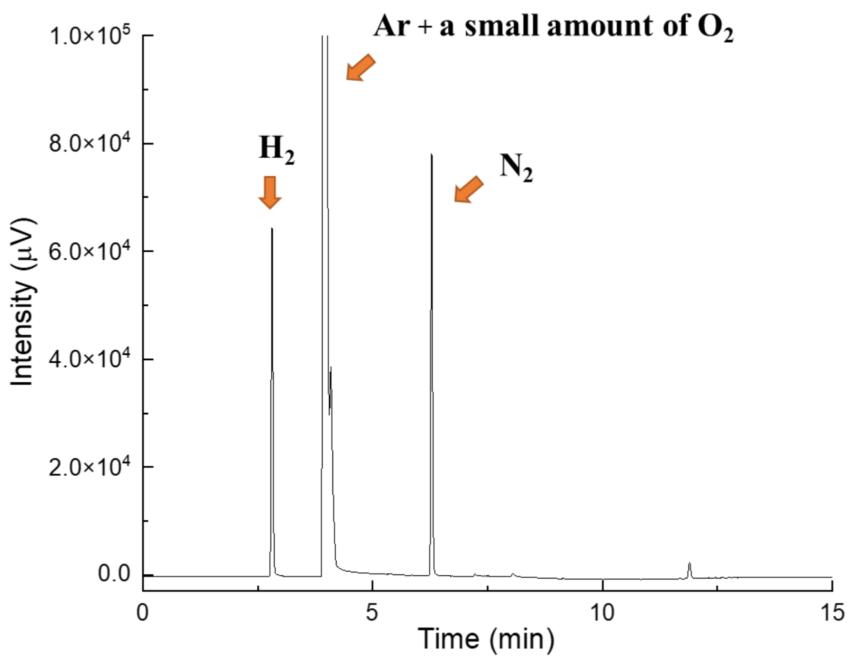


Figure S2. The GC sprctrum for detection of hydrogen gas $H_2$.

***Detected by NMR:***

***1) ¹H NMR***

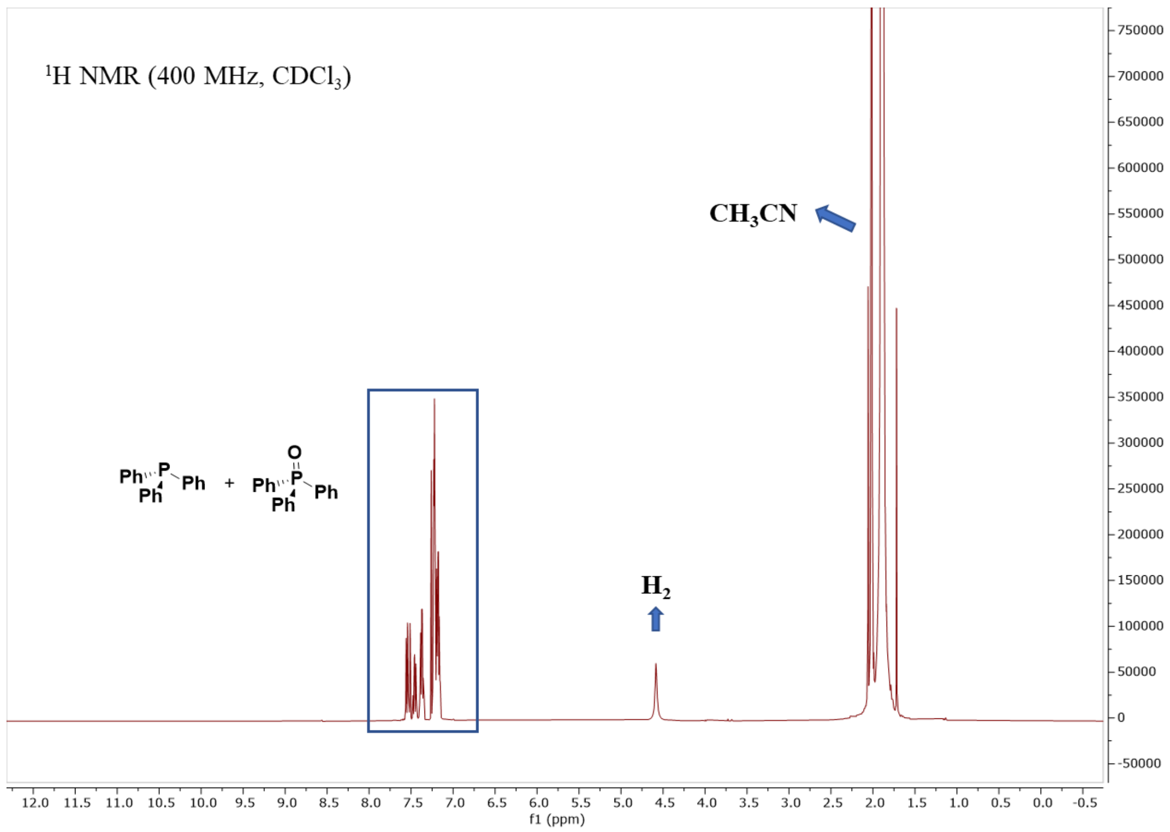

*2) $^{31}$P NMR*

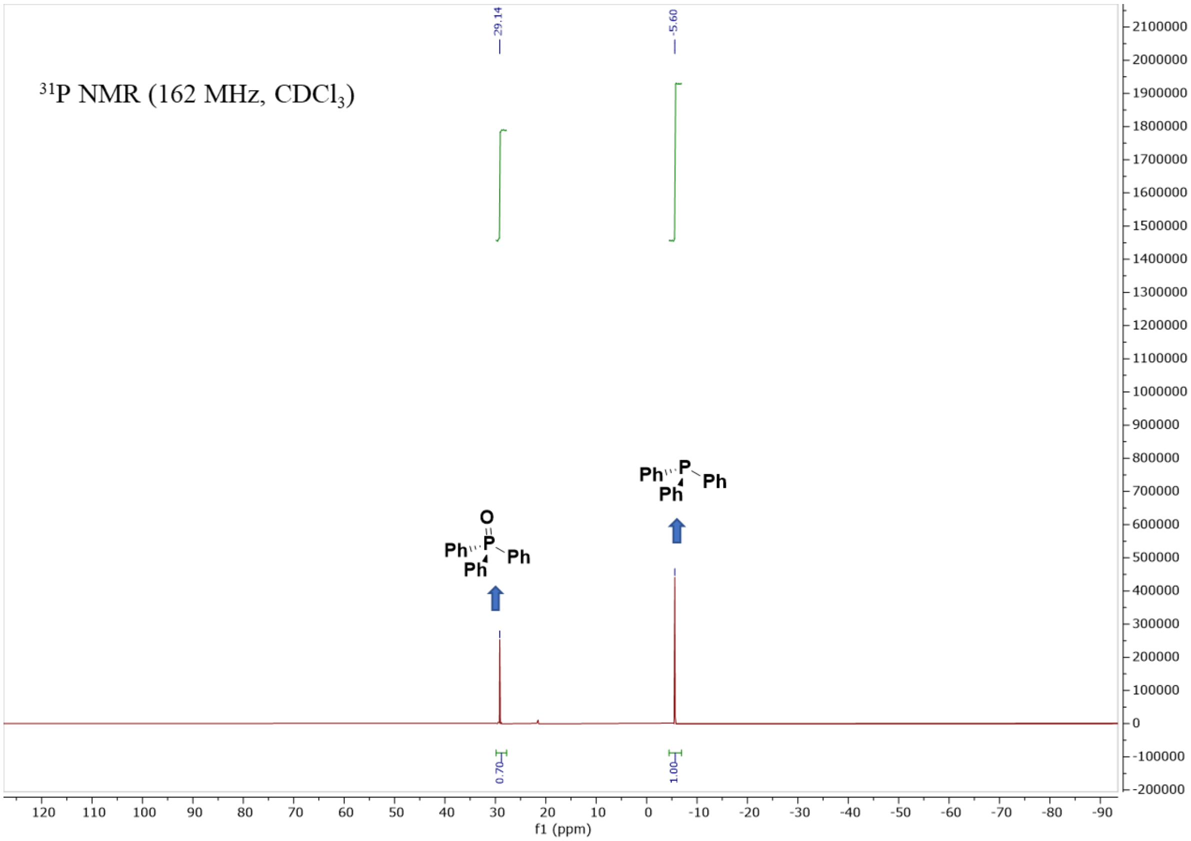

31P NMR (162 MHz, CDCl3)
29.14
-5.60
0.70
1.00
O
Ph
P
Ph
Ph
Ph
P
Ph
Ph
f1 (ppm)

## 8. Gram-scale experiments and recycle of phosphine oxides

### 2b:

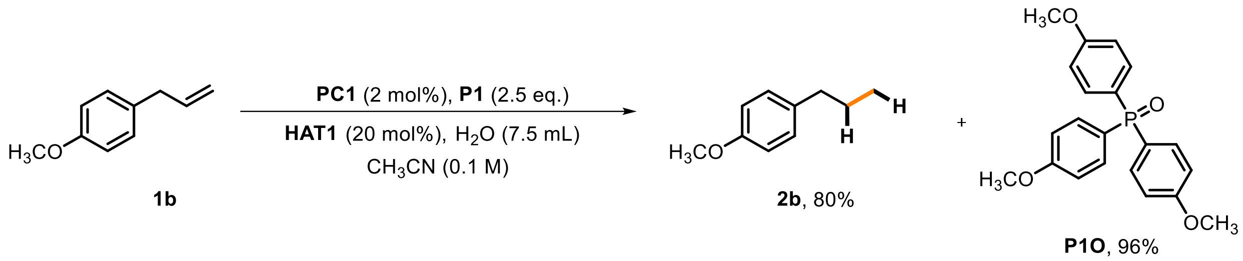


To an oven dried Schlenk tube with a magnetic stirring bar, **P1** (2.5 equiv.), **PC1** (2 mol%), deionized water (7.5 mL) and acetonitrile (100.0 mL) were added under argon atmosphere using standard Schlenk techniques at ambient temperature. Then, the substrate **1b** (10.0 mmol, 1.0 equiv.) and the HAT catalyst **HAT1** (2.0 mmol, 0.2 equiv.) were added under argon atmosphere. The tube was sealed and placed in the photoreactor, and then irradiated with two Kessil PR160L (40 W, 456 nm) lights at 20 °C using the standard set-up. After 72 hours, the irradiation was stopped. Hydrogen peroxide was added to oxide the remaining **P1** to the corresponding phosphine oxide. Then poured onto $H_2O$ and extracted with EtOAc (3 × 100 mL). The combined organic phases were dried over anhydrous $MgSO_4$, filtered and concentrated in vacuo. Purification of the resulting crude residue via silica gel flash chromatography (pentane) to afford the product **2b** (1.20 g, 80%) and phosphine oxide (**P1O**, 8.81 g, 96%).

### 4d:

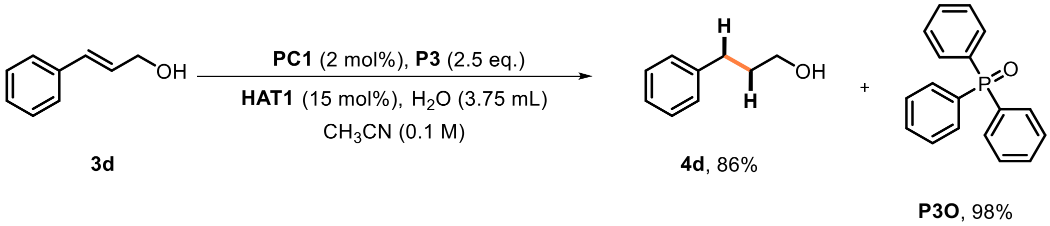


To an oven dried Schlenk tube with a magnetic stirring bar, **P3** (2.5 equiv.), **PC1** (2 mol%), deionized water (3.75 mL) and acetonitrile (50.0 mL) were added under argon atmosphere using standard Schlenk techniques at ambient temperature. Then, the substrate **3d** (5.0 mmol, 1.0 equiv.) and the HAT catalyst **HAT1** (0.75 mmol, 0.15 equiv.) were added under argon atmosphere. The tube was sealed and placed in the photoreactor, and then irradiated with two Kessil PR160L (40 W, 456 nm) lights at 20 °C using the standard set-up. After 72 hours, the irradiation was stopped. Hydrogen peroxide was added to oxide the remaining **P1** to the corresponding phosphine oxide. Then poured onto $H_2O$ and extracted with EtOAc (3 × 100 mL). The combined organic phases were dried over anhydrous $MgSO_4$, filtered and concentrated in vacuo. Purification of the resulting crude residue via silica gel flash chromatography (pentane/EtOAc 10/1) to afford the product **4d** (0.58 g, 86%) and phosphine oxide (**P3O**, 3.27 g, 98%).

**6c:**

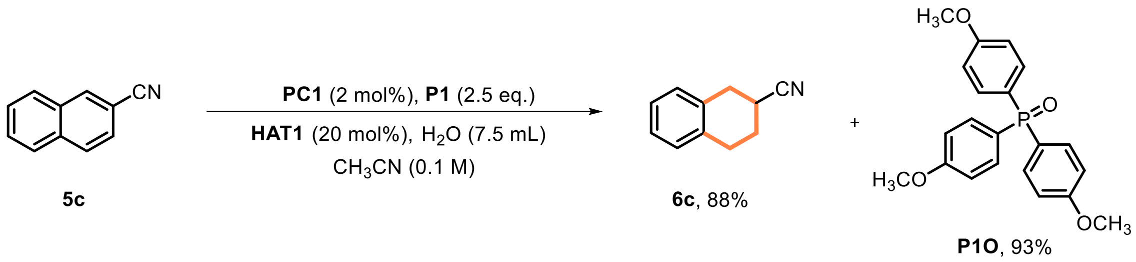


To an oven dried Schlenk tube with a magnetic stirring bar, **P1** (2.5 equiv.), **PC1** (2 mol%), deionized water (7.5 mL) and acetonitrile (100.0 mL) were added under argon atmosphere using standard Schlenk techniques at ambient temperature. Then, the substrate **5c** (10.0 mmol, 1.0 equiv.) and the HAT catalyst **HAT1** (2.0 mmol, 0.2 equiv.) were added under argon atmosphere. The tube was sealed and placed in the photoreactor, and then irradiated with two Kessil PR160L (40 W, 456 nm) lights at 20 °C using the standard set-up. After 72 hours, the irradiation was stopped. Hydrogen peroxide was added to oxide the remaining **P1** to the corresponding phosphine oxide. Then poured onto $H_2O$ and extracted with EtOAc (3 × 100 mL). The combined organic phases were dried over anhydrous $MgSO_4$, filtered and concentrated in vacuo. Purification of the resulting crude residue via silica gel flash chromatography (pentane) to afford the product **6c** (1.38 g, 88%) and phosphine oxide (**P1O**, 8.53 g, 93%).

## 9. Stern-Volmer quenching experiment and quantum yield measurment

### Stern-Volmer quenching experiment

Stern-Volmer luminescence quenching analysis was conducted using a Jasco FP8300 spectrofluorometer at 25 °C. The following parameters were employed: Excitation bandwidth = 5 nm, data interval = 0.2 nm, scan speed = 500 nm/min, response time = 0.2 sec. The samples were measured in Hellma fluorescence QS quartz cuvettes (chamber volume = 1.4 mL, H × W × D = 46 mm × 12.5 mm, 12.5 mm) fitted with a PTFE stopper. Samples were prepared in 1.4 mL quartz cuvettes equipped with PTFE stoppers inside an argon filled glove bag. The $CH_3CN$ solution of Ir[dF($CF_3$)ppy]$_2$(dtbbpy)$PF_6$ ($2.5 \times 10^{-5}$ M) were excited at $\lambda_{ex}$ = 380 nm and the emission was recorded at 475 nm. For each quenching experiment, 10 μl of the quenching reagent were titrated to a solution (1.4 mL) of Ir[dF($CF_3$)ppy]$_2$(dtbbpy)$PF_6$ in a screw-top 10.0 mm quartz cuvette $I_0$ is the luminescence intensity without the quencher, $I$ is the intensity in the presence of the quencher. The results are listed below:

| Entry | [$PPh_3$] / M | $I_o/I$ | [**1a**] / M | $I_o/I$ |
|---|---|---|---|---|
| 1 | 0 | 1 | 0 | 1 |
| 2 | 0.000521 | 1.04138 | 0.00102 | 0.9996 |
| 3 | 0.00104 | 1.08782 | 0.00204 | 0.9994 |
| 4 | 0.00156 | 1.11944 | 0.00408 | 0.9990 |
| 5 | 0.00188 | 1.13866 | 0.00816 | 0.9990 |
| 6 | 0.00229 | 1.16079 | 0.0163 | 0.9989 |

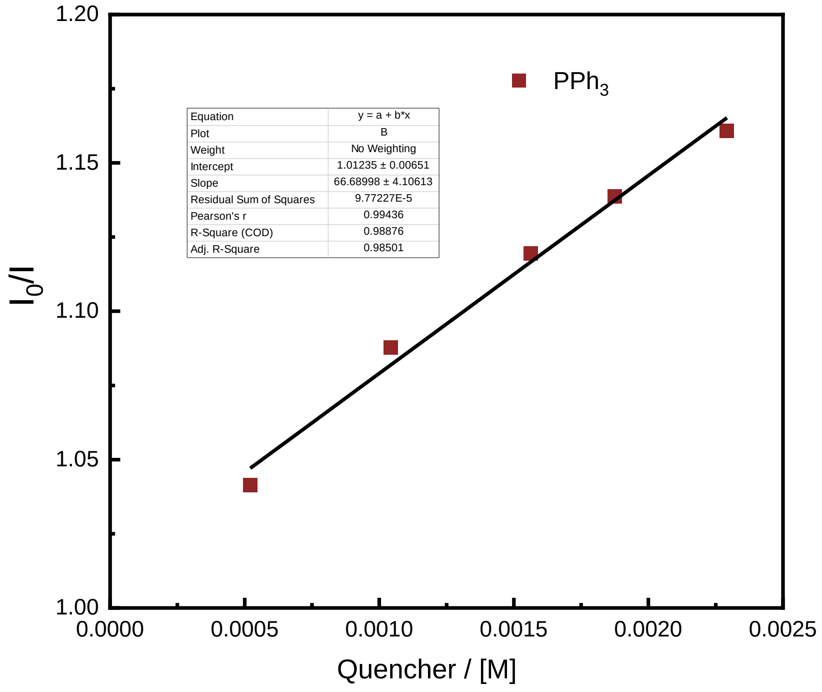


Figure S3. Stern-Volmer quenching experiment.

**Determination of the light intensity of 5 W-455 nm LEDs**

Standard ferrioxalate actinometry[60,61] was used to determine the photon flux of 5 W-455 nm LEDs. A solution of ferrioxalate (0.15 M) was prepared by dissolving potassium ferrioxalate trihydrate (737 mg, 1.50 mmol) in 10.0 mL of 0.20 M aqueous $H_2SO_4$. A buffered solution of 1,10-phenanthroline (0.15 M) was prepared by dissolving NaOAc (1.23 g, 15.0 mmol) and 1,10-phenanthroline (541 mg, 3.00 mmol) in 20 mL of 0.20 M aqueous $H_2SO_4$. To a 10-mL borosilicate vial equipped with a stir bar was added 1.0 mL of the ferrioxalate solution. The vial was sealed with a Teflon cap and placed in the 5 W-455 nm LEDs. After being irradiated for 20 seconds, 0.5 mL of the reaction mixture was taken out immediately and injected into a 4-mL borosilicate vial containing 1.5 mL of the aqueous sulfuric acid and 2.0 mL of the buffered solution. The resulting mixture was then allowed to rest for 1 hour to allow the formed ferrous ions to react completely with 1,10-phenanthroline. An aliquot (25 µL) of the resulting solution was diluted with 3.0 mL of 0.20 M aqueous sulfuric acid, and the absorbance in a cuvette ($l$ = 1.0 cm) at 510 nm was measured by UV-Vis spectrometer. The above procedure was repeated four more times, and the average absorption was used for the calculation of photon flux. A nonirradiated sample was also prepared and the absorbance at 510 nm was measured. The photon flux was calculated as follows:

$$\textbf{mol Fe}^{2+} = \frac{V \text{ x } \Delta A \text{ (510 nm)}}{l \text{ x } \varepsilon}$$

where $V$ is the total volume (0.96 L) of the solution that was analyzed, $\Delta A$ (0.23) is the difference between the average absorption of irradiated and non-irradiated solutions at 510 nm, $l$ is the path length (1.00 cm), and $\varepsilon$ is the molar absorptivity of the ferrioxalate actinometer at 510 nm (11100 L $mol^{-1}$ $cm^{-1}$).

The photon flux was calculated as follows:

$$\textbf{photon flux} = \frac{\text{mol Fe}^{2+}}{\Phi \text{ x } t \text{ x } f}$$

where $\Phi$ is the quantum yield for the ferrioxalate actinometer (approximated as 0.9),[62,63] $t$ is the irradiation time (20 s), and $f$ is the fraction of light absorbed at $\lambda$ = 455 nm by the ferrioxalate actinometer. This value is calculated using the following equation where $A$(455 nm) is the absorption of the ferrioxalate solution at 455 nm. An absorption spectrum gave an $A$(455) value of 1.84, indicating that the fraction of absorbed light ($f$) is 0.986.

$$f = 1 - 10^{-A(455\ nm)}$$

The average photon flux was thus calculated to be $1.12 \times 10^{-6}$ einsteins $s^{-1}$.

**Determination of quantum yield**

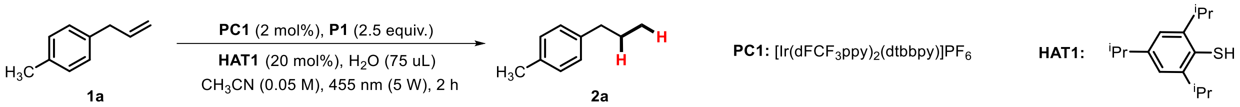


To an oven dried Schlenk tube with a magnetic stirring bar, **P1** (0.25 mmol, 2.5 equiv.), **PC1** (0.002 mmol, 2 mol%) and $CH_3CN$ (2.0 mL) were added under argon atmosphere using standard Schlenk techniques at ambient temperature. Then, alkene **1a** (0.1 mmol, 1.0 equiv.) and **HAT1** (0.02 mmol, 0.2 equiv.) were added under argon. The tube was sealed and placed in the photoreactor, and then irradiated with a 5 W 455 nm LED at 20 $^{o}$C using the standard set-up. After one hour, the irradiation was stopped. The yield of product **2a** (6% yield) was determined by gas chromatography with tetradecane as internal standard. The quantum yield was calculated as follows:

$$\boldsymbol{\Phi} = \frac{\textbf{mol product}}{\textbf{photo flux x } \boldsymbol{t} \textbf{ x } \boldsymbol{f}}$$

where mol product is the amount of product **2a** ($1.2 \times 10^{-5}$ mol), photon flux is determined by above ferrioxalate actinometry ($1.12 \times 10^{-6}$ einsteins $s^{-1}$), $t$ is the reaction time (7200 s), and $f$ is the fraction of light absorbed by the reaction mixture at 455 nm. This value is calculated using the following equation where $A$(455 nm) is the absorption of the non-irradiated reaction mixture (3.22) at 455 nm. (Note: the reaction mixture was pre-stirred for 30 min without irradiation, and the resulting solution was filtered through a syringe filter before measuring the absorption spectrum). The fraction of light absorbed at 455 nm was calculated:

$$\boldsymbol{f} = \mathbf{1 - 10^{-A(455\ nm)}}$$

Thus, the quantum yield was calculated as $\Phi = 0.0019$.

Comment: The relatively low quantum yield indicates that there are several possible pathways for energy besides the pathway generating the corresponding product and also shows that a chain reaction is not operative. Hence, the Ir-catalyst is a true catalyst and not an initiator in this transformation.

## 10. Chemoselective hydrogenation experiments

### Competition experiments

To an oven dried Schlenk tube with a magnetic stirring bar, **P** (**P1** or **P3**, 4.0 equiv.), **PC1** (2 mol%), deionized water (0.15 mL) and acetonitrile (4.0 mL) were added under argon atmosphere using standard Schlenk techniques at ambient temperature. Then, **substrate-1** (0.2 mmol, 1.0 equiv.), **substrate-2** (0.2 mmol, 1.0 equiv.) and the HAT catalyst **HAT1** (0.04 mmol, 0.2 equiv.) were added under argon atmosphere. The tube was sealed and placed in the photoreactor, and then irradiated with a 5 W 455 nm LED at 20 $^{o}$C using the standard set-up. After 16 hours, the irradiation was stopped. The yield ratio was determined by the GC with tetradecane as the internal standard.

Table S7. Competition experiments for chemoselective hydrogenation.

**substrate-1** (0.2 mmol) + **substrate-2** (0.2 mmol) → **product-1** + **product-2**

Conditions: **PC1 (2 mol%), P (4.0 eq.)**; **HAT1 (20 mol%), $H_2O$ (0.15 mL)**; **$CH_3CN$ (0.05 M)**

| Entry | substrate-1 | substrate-2 | P | Time | Yield (product-1/product-2) |
|---|---|---|---|---|---|
| 1 | 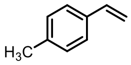 | 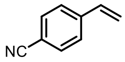 | **P3** | 16 h | 15% / **21%** |
| 2 | 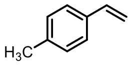 | 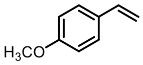 | **P3** | 16 h | 10% / **20%** |
| 3 | 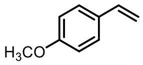 | 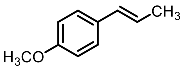 | **P3** | 16 h | **16%** / 7% |
| 4 | 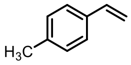 | 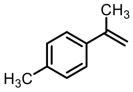 | **P3** | 16 h | **19%** / 9% |
| 5 | 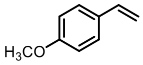 | 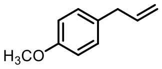 | **P3** | 16 h | **14%** / 0% |
| 6 | 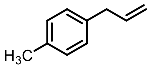 | 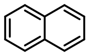 | **P1** | 16 h | **32%** / 0% |
| 7 | 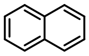 | 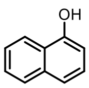 | **P1** | 16 h | 14% / **25%** |
| 8 | 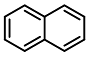 | 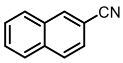 | **P1** | 16 h | 0 / **41%** |
| 9 | 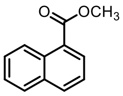 | 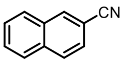 | **P1** | 16 h | 5% / **18%** |

Comment: Considering styrene pairs that slightly differ in their electronics by variation of their para-substituents, low selectivity was noted using the milder $PPh_3$-derived H-donor (Me vs. CN = 1:1.4; Me vs. MeO = 1:2). Steric effects play a role as *α*-methyl (trans) and also *β*-methyl-substituted styrenes are less reactive than their corresponding unsubstituted styrenes (around 2:1). Comparing an activated alkene with an unactivated alkene, we found excellent chemoselectivity for the hydrogenation with the milder **P3** as mediator. The same outcome was noted for the hydrogenation pair of unactivated alkene and naphthalene with more reactive **P1** as mediator. Considering HAT to naphthalenes, we ran a series of competition experiments. Only in the case of 2-cyanonaphthalene versus naphthalene, complete chemoselectivity for the hydrogenation of cyanonaphthalene was obtained. 1-Naphthol was around 1.8 times more reactive than naphthalene. 2-Cyanonaphthalene was 3.6 times more reactive than 1-methoxycarbonylnapthalene.

**Chemoselective hydrogenation**

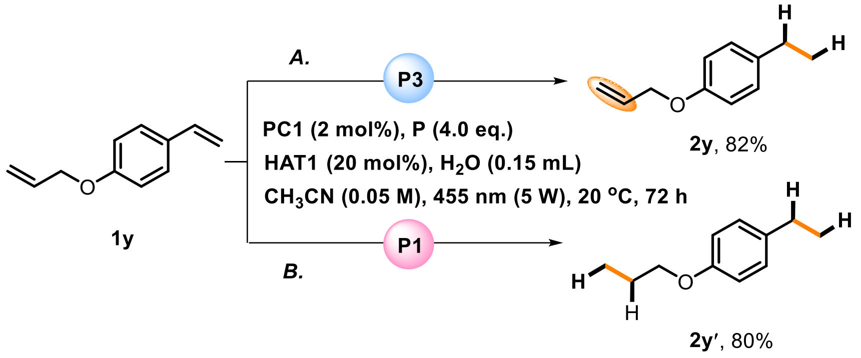


**Process A:** To an oven dried Schlenk tube with a magnetic stirring bar, **P3** (4.0 equiv.), **PC1** (2 mol%), deionized water (0.15 mL) and acetonitrile (4.0 mL) were added under argon atmosphere using standard Schlenk techniques at ambient temperature. Then, **1y** (0.2 mmol, 1.0 equiv.) and the HAT catalyst **HAT1** (0.04 mmol, 0.2 equiv.) were added under argon atmosphere. The tube was sealed and placed in the photoreactor, and then irradiated with a 5 W 455 nm LED at 20 °C using the standard set-up. After 72 hours, the irradiation was stopped. The yield was determined by the GC with tetradecane as the internal standard. The product analysis showed that only activated alkene of **1y** was reduced to give **2y** with 82% yield.

**Process B:** To an oven dried Schlenk tube with a magnetic stirring bar, **P1** (4.0 equiv.), **PC1** (2 mol%), deionized water (0.15 mL) and acetonitrile (4.0 mL) were added under argon atmosphere using standard Schlenk techniques at ambient temperature. Then, **1y** (0.2 mmol, 1.0 equiv.) and the HAT catalyst **HAT1** (0.04 mmol, 0.2 equiv.) were added under argon atmosphere. The tube was sealed and placed in the photoreactor, and then irradiated with a 5 W 455 nm LED at 20 °C using the standard set-up. After 72 hours, the irradiation was stopped. The yield was determined by the GC with tetradecane as the internal standard. The product analysis showed that **1y** was fully reduced to give **2y'** with 80% yield.

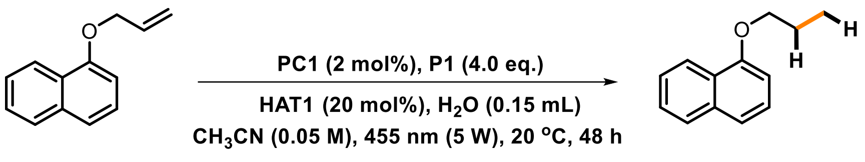


**1z** **2z**, 42%

To an oven dried Schlenk tube with a magnetic stirring bar, **P1** (4.0 equiv.), **PC1** (2 mol%), deionized water (0.15 mL) and acetonitrile (4.0 mL) were added under argon atmosphere using standard Schlenk techniques at ambient temperature. Then, **1z** (0.2 mmol, 1.0 equiv.) and the HAT catalyst **HAT1** (0.04 mmol, 0.2 equiv.) were added under argon atmosphere. The tube was sealed and placed in the photoreactor, and then irradiated with a 5 W 455 nm LED at 20 °C using the standard set-up. After 48 hours, the irradiation was stopped. The yield was determined by the GC with tetradecane as the internal standard. The product analysis showed that only alkene of **1z** was reduced to give **2z** with 42% yield.

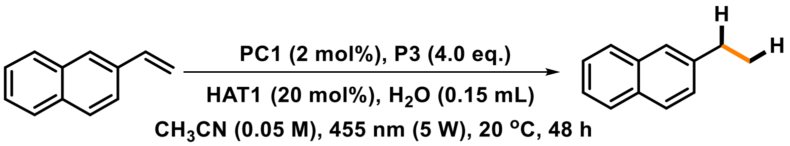


**3n** **4n**, 80% (87% with **P1**, 24 h)

To an oven dried Schlenk tube with a magnetic stirring bar, **P3** (4.0 equiv.), **PC1** (2 mol%), deionized water (0.15 mL) and acetonitrile (4.0 mL) were added under argon atmosphere using standard Schlenk techniques at ambient temperature. Then, **3n** (0.2 mmol, 1.0 equiv.) and the HAT catalyst **HAT1** (0.04 mmol, 0.2 equiv.) were added under argon atmosphere. The tube was sealed and placed in the photoreactor, and then irradiated with a 5 W 455 nm LED at 20 °C using the standard set-up. After 48 hours, the irradiation was stopped. The yield was determined by the GC with tetradecane as the internal standard. The product analysis showed that only alkene of **3n** was reduced to give **4n** with 80% yield.

The $^1H$ NMR spectrum of the reaction mixture:

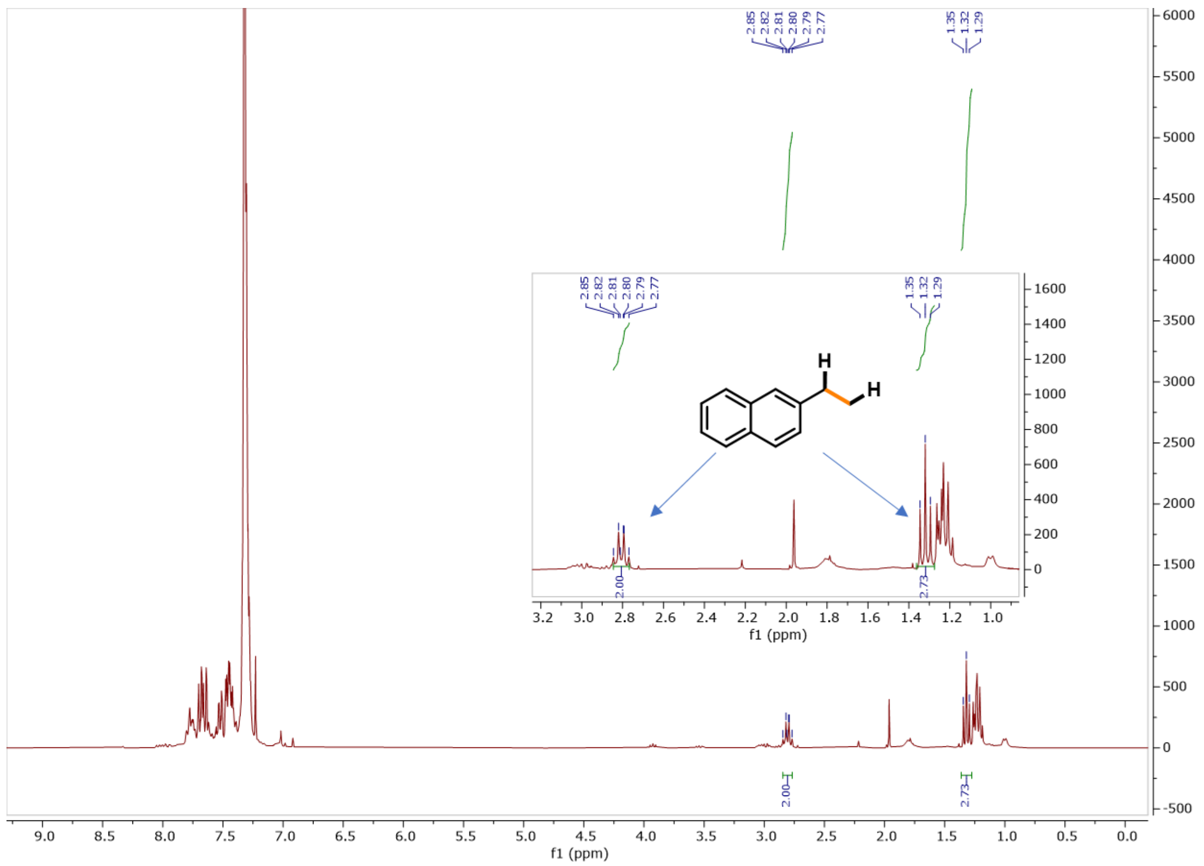

2.85
2.82
2.81
2.80
2.79
2.77
1.35
1.32
1.29
2.00
2.73
f1 (ppm)
H
H

## 11. Kinetic experiments

### 11.1 The reaction order of alkenes in our conditions

To an oven dried Schlenk tube with a magnetic stirring bar, **P1** (0.5 mmol), **PC1** (0.01 mmol), deionized water (0.15 mL) and acetonitrile (4.0 mL) were added under argon atmosphere using standard Schlenk techniques at ambient temperature. Then, the substrate **1a** (0.2 mmol, 0.4 mmol and 0.6 mmol for three different Schlenk tubes, respectively), the HAT catalyst **HAT1** (0.04 mmol) and tetradecane (internal standard, 0.1 mmol) were added under argon atmosphere.  The tube was sealed and placed in the photoreactor, and then irradiated with a 5 W 455 nm LED at 20 °C using the standard set-up. At each indicated time an aliquot (60 μL) of the reaction mixture was taken under positive argon pressure, which was diluted and analyzed by GC.

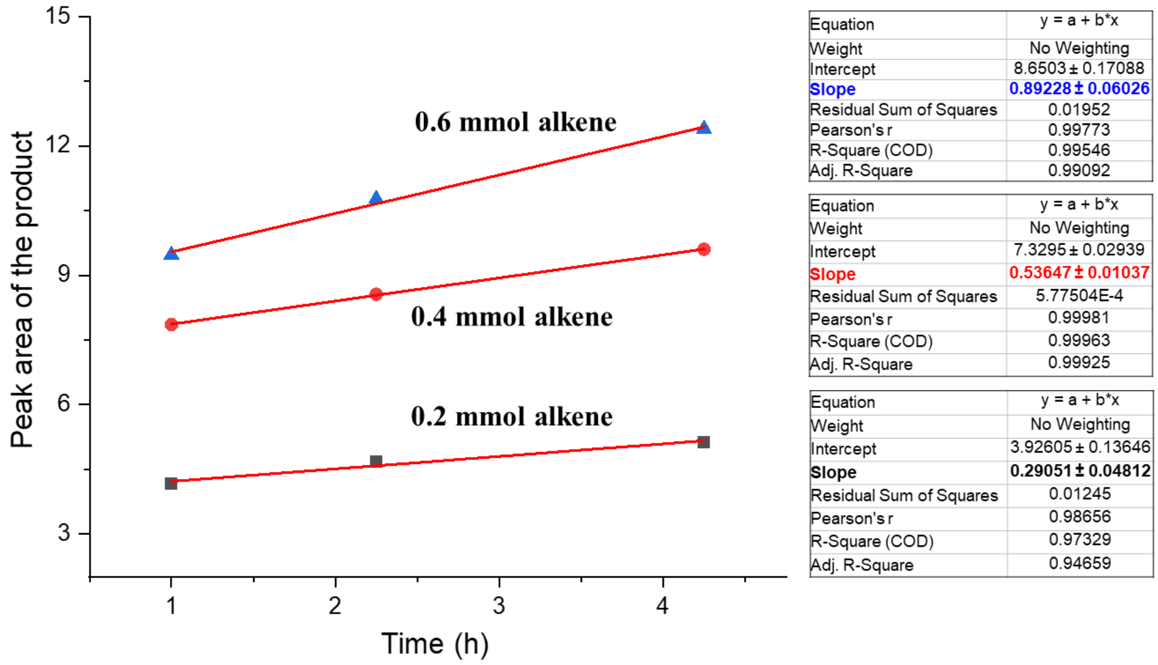


Figure S4. The measutrement of reaction order of alkenes in our conditions.

Comment: The reaction is first order in the alkene, indicating that the reaction step where the alkene is involved is involved in the rate-determining step.

## 11.2 The reaction order of the HAT catalyst in our conditions

To an oven dried Schlenk tube with a magnetic stirring bar, **P1** (0.5 mmol), **PC1** (0.01 mmol), deionized water (0.15 mL) and acetonitrile (4.0 mL) were added under argon atmosphere using standard Schlenk techniques at ambient temperature. Then, the substrate **1j** (0.2 mmol), the HAT catalyst **HAT1** (0.02 mmol, 0.04 mmol and 0.06 mmol for three different Schlenk tubes, respectively) and tetradecane (internal standard, 0.2 mmol) were added under argon atmosphere. The tube was sealed and placed in the photoreactor, and then irradiated with a 5 W 455 nm LED at 20 ºC using the standard set-up. At each indicated time an aliquot (60 µL) of the reaction mixture was taken under positive argon pressure, which was diluted and analyzed by GC.

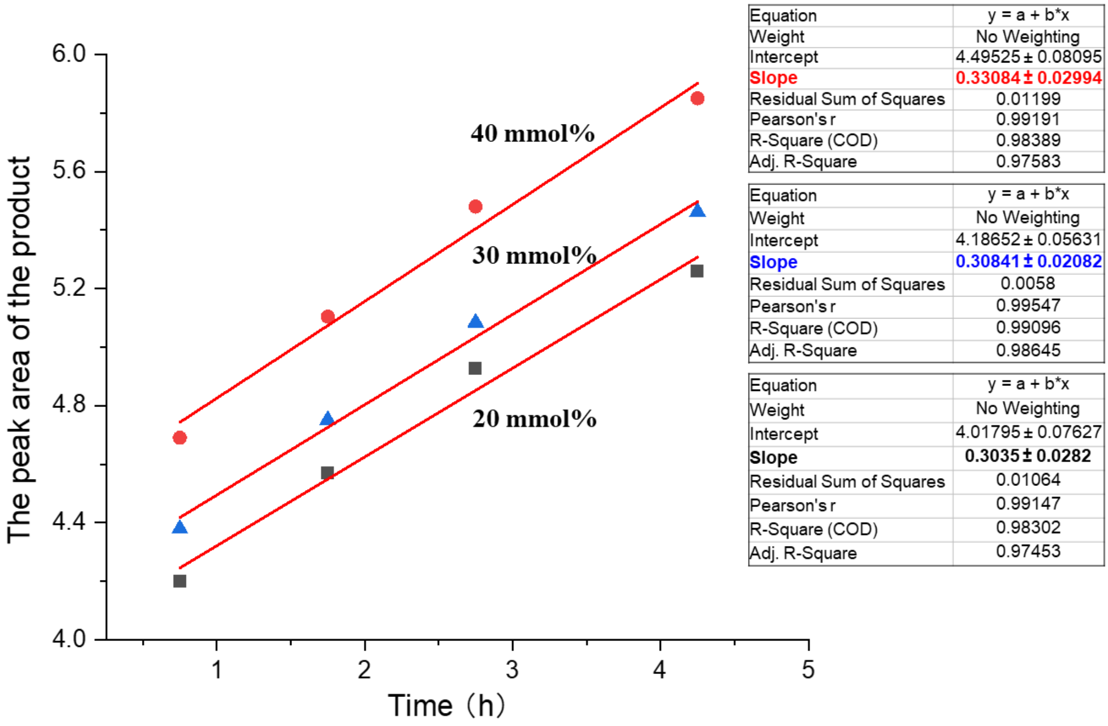


| | |
|---|---|
| Equation | y = a + b*x |
| Weight | No Weighting |
| Intercept | 4.49525 ± 0.08095 |
| Slope | 0.33084 ± 0.02994 |
| Residual Sum of Squares | 0.01199 |
| Pearson's r | 0.99191 |
| R-Square (COD) | 0.98389 |
| Adj. R-Square | 0.97583 |

| | |
|---|---|
| Equation | y = a + b*x |
| Weight | No Weighting |
| Intercept | 4.18652 ± 0.05631 |
| Slope | 0.30841 ± 0.02082 |
| Residual Sum of Squares | 0.0058 |
| Pearson's r | 0.99547 |
| R-Square (COD) | 0.99096 |
| Adj. R-Square | 0.98645 |

| | |
|---|---|
| Equation | y = a + b*x |
| Weight | No Weighting |
| Intercept | 4.01795 ± 0.07627 |
| Slope | 0.3035 ± 0.0282 |
| Residual Sum of Squares | 0.01064 |
| Pearson's r | 0.99147 |
| R-Square (COD) | 0.98302 |
| Adj. R-Square | 0.97453 |

Figure S5. The measurement of reaction order of the HAT catalyst in our conditions.

Comment: The reaction is zero order in the HAT catalyst, indicating the reaction step where the thiol is involved is not the rate-determining step.

## 11.3 The KIE measurement

To an oven dried Schlenk tube with a magnetic stirring bar, **P3** (0.5 mmol), **PC1** (0.01 mmol), $H_2O$ or $D_2O$ (0.15 mL for two different Schlenk tubes, respectively) and acetonitrile (4.0 mL) were added under argon atmosphere using standard Schlenk techniques at ambient temperature. Then, the substrate **3a** (0.2 mmol), the HAT catalyst **HAT1** (0.03 mmol) and tetradecane (internal standard, 0.2 mmol) were added under argon atmosphere. The tube was sealed and placed in the photoreactor, and then irradiated with a 5 W 455 nm LED at 20 $^{o}$C using the standard set-up. At each indicated time an aliquot (60 μL) of the reaction mixture was taken under positive argon pressure, which was diluted and analyzed by GC.

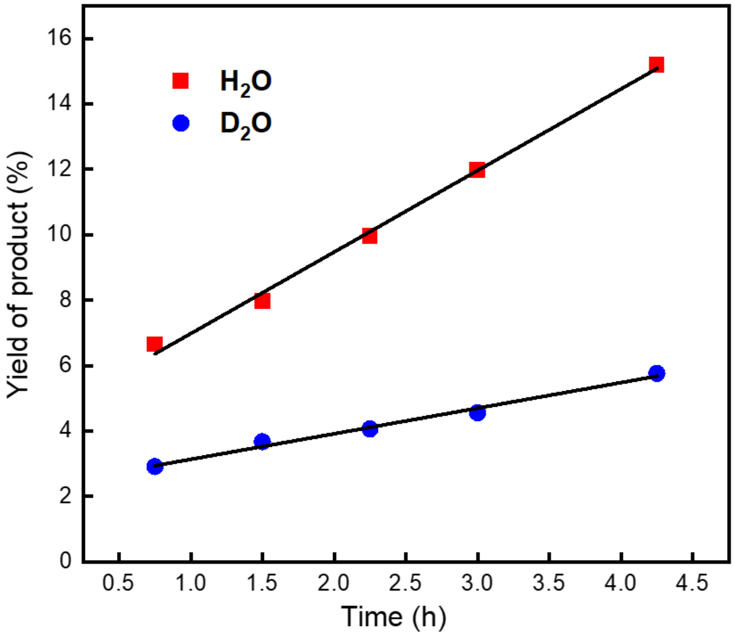


| | |
|---|---|
| Equation | y = a + b*x |
| Weight | No Weighting |
| Intercept | 4.488 ± 0.24727 |
| **Slope** | **2.49276 ± 0.09354** |
| Residual Sum of Squares | 0.19229 |
| Pearson's r | 0.99789 |
| R-Square (COD) | 0.99579 |
| Adj. R-Square | 0.99439 |

| | |
|---|---|
| Equation | y = a + b*x |
| Weight | No Weighting |
| Intercept | 2.35483 ± 0.1269 |
| **Slope** | **0.78177 ± 0.04801** |
| Residual Sum of Squares | 0.05065 |
| Pearson's r | 0.99439 |
| R-Square (COD) | 0.98881 |
| Adj. R-Square | 0.98508 |

Figure S6. The rate constants for the reactions using $H_2O$ or $D_2O$ as hydrogen source.

Comment: The KIE value of the reaction is 3.2, indicating that the cleavage of the O-H bond is involved in the rate-determining step.

## 12. DFT calculations

### 12.1 Methods

All structures were optimized without geometry constraints using the PBE0 hybrid functional[64,65] and an atom-pairwise dispersion correction (D3).[66,67] A flexible triple zeta basis set (def2-TZVP)[68] was used in all calculations. The nature of the optimized stationary points was proven by the presence of either 0 (minimum) or 1 (transition structure) imaginary vibrational frequency. For the determination of the enthalpy and free enthalpy contributions at 298 K ($H^{\mathrm{RRHO}}$, $G^{\mathrm{RRHO}}$), the rigid-rotor-harmonic-oscillator model and a rotor approximation for vibrational modes with wave numbers below 100 $cm^{-1}$ was applied.[69] Electronic energies of the structures optimized with PBE0-D3 were recalculated with the double hybrid functional PWPB95(-D3)[70] using the def2-TZVP basis set and the frozen core option for the perturbation theory part of the correlation energy. Solvation free energies ($G_{\mathrm{solv}}$) for $T$ = 298.15 K in acetonitrile were obtained with COSMO-RS.[71,72] Relative values of free enthalpy in solution ($\Delta G^{\mathrm{s}}_{298}$), *i.e.* the activation free energies of HAT for **P3**-OH were obtained using the sum of the differences in PWPB95-D3 electronic energies, $G^{\mathrm{RRHO}}$(298K), and $G_{\mathrm{solv}}$ as

$$\Delta G^{\mathrm{s}}_{298} = \Delta E(\mathrm{PWPB95\text{-}D3}) + \Delta G^{\mathrm{RRHO}}(298\mathrm{K}) + \Delta G_{\mathrm{solv}} \qquad (1)$$

Bond dissociation energies for the H-atom donors (Table S9) were calculated as enthalpies for the reaction R-H → R + H$^{\bullet}$:

$$\mathrm{BDE} = \Delta H^{\mathrm{o}}(298\mathrm{K}) = \Delta E(\mathrm{PWPB95\text{-}D3}) + \Delta H^{\mathrm{RRHO}}(298\mathrm{K}) \qquad (2)$$

Spin density isosurfaces of **P3**-OH and the transition structures of H-atom transfer were calculated with PBE0/def2-TZVP (Figure S7).

All PBE0 calculations were performed with the TURBOMOLE program.[73,74] For the PWPB95-D3 double hybrid energy calculations, ORCA was used.

## 12.2 DFT calculated energies

Table S8. DFT-calculates energies and thermostatistical contributions of all molecular species.

| Structure | $E$(PBE0-D3) [$E_h$] | $G^{RRHO}_{298\ K}$ [kcal/mol] | $H^{RRHO}_{298\ K}$ [kcal/mol] | $E$(PWPB95-D3)[a] [$E_h$] | $G_{solv}(CH_3CN)$ COSMO-RS [kcal/mol] |
|---|---|---|---|---|---|
| **P1-OH** | -1454.733077 | 209.257 | 258.268 | -1455.231938 | -17.823 |
| **P1=O** | -1454.211067 | 204.716 | 252.999 | -1454.719451 | -18.334 |
| **P2-OH** | -1229.245314 | 200.478 | 246.869 | -1229.635654 | -13.629 |
| **P2=O** | -1228.717969 | 195.187 | 241.470 | -1229.117739 | -14.150 |
| **P3-OH** | -1111.390124 | 153.047 | 191.906 | -1111.748784 | -13.046 |
| **P3=O** | -1110.861463 | 148.256 | 186.528 | -1111.229267 | -13.071 |
| **P3-Hortho** | -1111.412034 | 153.466 | 192.331 | -1111.771294 | -12.751 |
| **P3-TS-STYR** | -1420.791573 | 229.998 | 278.875 | -1421.258076 | -14.574 |
| **P3-TS-PROP** | -1229.184121 | 197.381 | 242.603 | -1229.573328 | -11.071 |
| **P4-OH** | -1408.968422 | 135.829 | 178.183 | -1409.475076 | -13.024 |
| **P4=O** | -1408.442921 | 131.196 | 172.833 | -1408.959484 | -12.743 |
| **P5-OH** | -2122.041959 | 153.338 | 208.033 | -2122.885518 | -15.234 |
| **P5=O** | -2121.506149 | 147.796 | 202.543 | -2122.359449 | -13.177 |
| **CH4** | -40.475114 | 16.387 | 30.463 | -40.485495 | 4.050 |
| **CH3** | -39.799762 | 7.348 | 21.206 | -39.808846 | 3.689 |
| **PROPENE** | -117.800541 | 34.347 | 53.182 | -117.840296 | 2.593 |
| **STYRENE** | -309.390352 | 64.434 | 88.691 | -309.506185 | -1.553 |
| **H.** | -0.501037 | -6.271 | 1.481 | -0.498413 | |

[a] calculated using the structures optimized with PBE0-D3

Table S9. DFT-calculated BDE(O-H) for $PR_3O$-H radicals from values in Table S8 using Eqn. (2).

| Radical | Ar | BDE = Δ$H^o$(298K) |
|---|---|---|
| **P1-OH** | (p-OMe)Ph | 5.04 |
| **P2-OH** | (p-Me)Ph | 8.32 |
| **P3-OH** | Ph | 9.35 |
| **P4-OH** | (p-F)Ph | 6.91 |
| **P5-OH** | (p-$CF_3$)Ph | 13.35 |

Table S10. DFT-calculates energies and thermostatistical contributions of HAT from **P1**-OH to naphthalenes **5b**, **5c** and **5f**.

| Structure | $E$(PBE0-D3) [$E_h$] | $G^{RRHO}_{298}$ [kcal/mol] | $E$(PWPB95-D3)[a] [$E_h$] | $G_{solv}$ ($CH_3CN$) COSMO-RS [kcal/mol] |
|---|---|---|---|---|
| **5f** | -424.863736 | 89.528 | -425.019969 | -3.233 |
| **5f-TS2** | -1879.606497 | 311.722 | -1880.257285 | -20.940 |
| **5f-TS3** | -1879.608540 | 312.471 | -1880.260030 | -21.115 |
| **5f-TS4** | -1879.613187 | 312.273 | -1880.263211 | -21.117 |
| **5f-TS5** | -1879.611789 | 311.882 | -1880.262396 | -21.080 |
| **5f-TS6** | -1879.607436 | 312.232 | -1880.259216 | -21.084 |
| **5f-TS7** | -1879.607591 | 312.218 | -1880.258516 | -20.987 |
| **5f-TS8** | -1879.612416 | 312.012 | -1880.262561 | -20.669 |
| **5f-H2** | -425.414915 | 94.206 | -425.562460 | -3.234 |
| **5f-H3** | -425.414505 | 94.781 | -425.562268 | -3.116 |
| **5f-H4** | -425.422786 | 94.648 | -425.570332 | -3.034 |
| **5f-H5** | -425.421808 | 94.576 | -425.569316 | -3.077 |
| **5f-H6** | -425.413159 | 94.428 | -425.560763 | -3.188 |
| **5f-H7** | -425.414626 | 94.588 | -425.562282 | -3.192 |
| **5f-H8** | -425.422555 | 94.531 | -425.570002 | -3.156 |
| **5b** | -613.306849 | 96.800 | -613.552730 | -5.942 |
| **5b-H2** | -613.864470 | 102.295 | -614.102063 | -5.999 |
| **5b-H3** | -613.856106 | 101.877 | -614.093572 | -6.318 |
| **5b-H4** | -613.869797 | 102.144 | -614.107187 | -6.114 |
| **5b-H5** | -613.865539 | 101.855 | -614.102569 | -5.963 |
| **5b-H6** | -613.856921 | 101.829 | -614.094027 | -5.983 |
| **5b-H7** | -613.859557 | 101.868 | -614.096676 | -5.820 |
| **5b-H8** | -613.866840 | 101.901 | -614.104200 | -5.413 |
| **5c** | -477.753151 | 70.916 | -477.949053 | -6.627 |
| **5c-H1** | -478.318361 | 76.300 | -478.504863 | -6.791 |
| **5c-H3** | -478.306941 | 76.039 | -478.493554 | -6.677 |
| **5c-H4** | -478.311148 | 75.968 | -478.498033 | -6.683 |
| **5c-H5** | -478.311995 | 75.996 | -478.499030 | -6.615 |
| **5c-H6** | -478.306605 | 76.080 | -478.493584 | -7.084 |
| **5c-H7** | -478.304331 | 75.942 | -478.491302 | -6.697 |
| **5c-H8** | -478.313416 | 76.068 | -478.500225 | -6.786 |

[a] calculated using the structures optimized with PBE0-D3

## 12.3 Optimized molecular structures

Figure S7. Molecular structures of all intermediates and transition structures optimized with PBE0-D3/def2-TZVP. Distances are given in Å. Element colors are gray (C), white (H), red (O), orange (P), green (F).

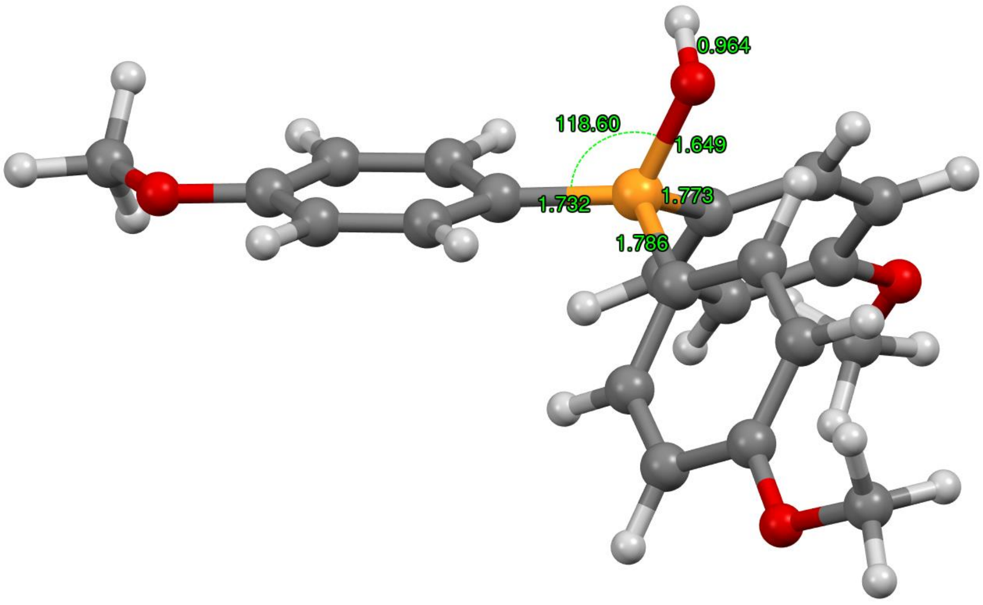


**P1-OH**

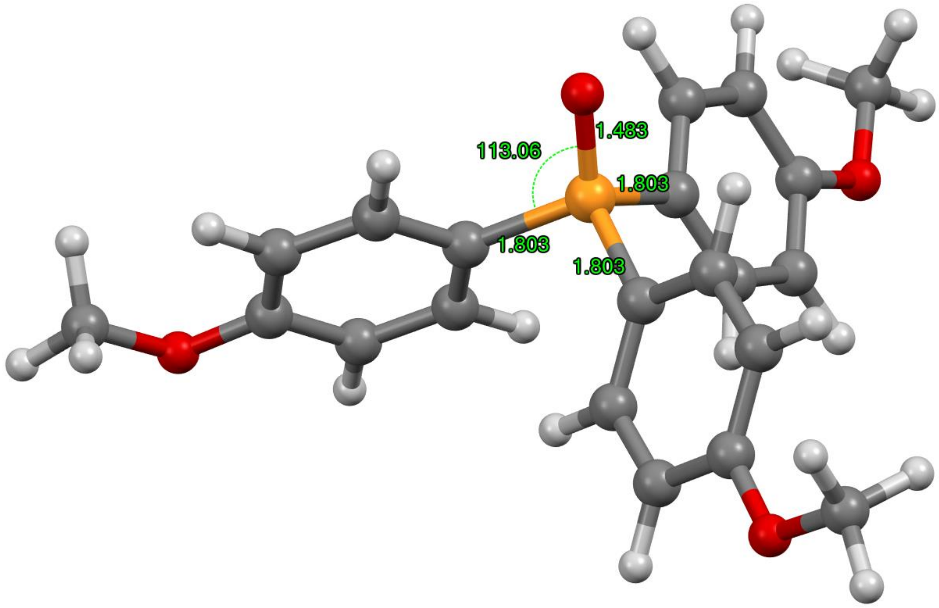


**P1=O**

Figure S7 (continued). Molecular structures of all intermediates and transition structures optimized with PBE0-D3/def2-TZVP. Distances are given in Å. Element colors are gray (C), white (H), red (O), orange (P), green (F).

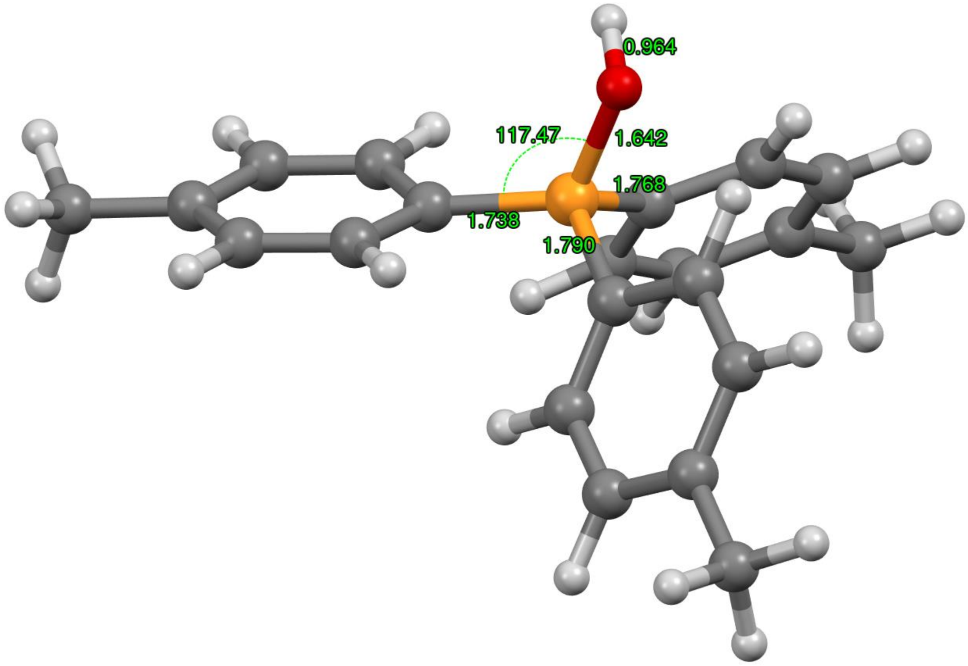


**P2-OH**

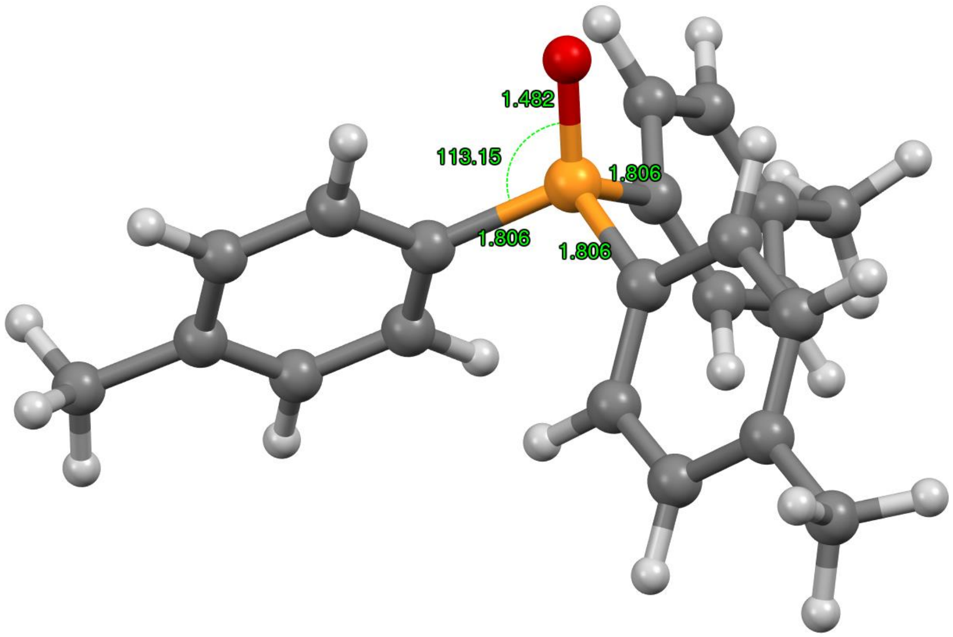


**P2=O**

Figure S7 (continued). Molecular structures of all intermediates and transition structures optimized with PBE0-D3/def2-TZVP. Distances are given in Å. Element colors are gray (C), white (H), red (O), orange (P), green (F).

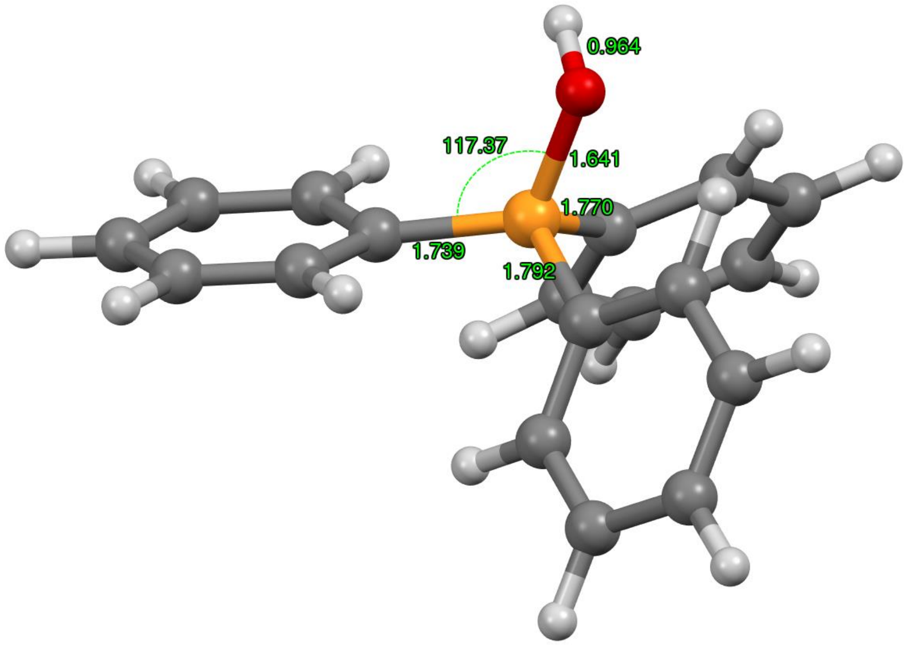


**P3-OH**

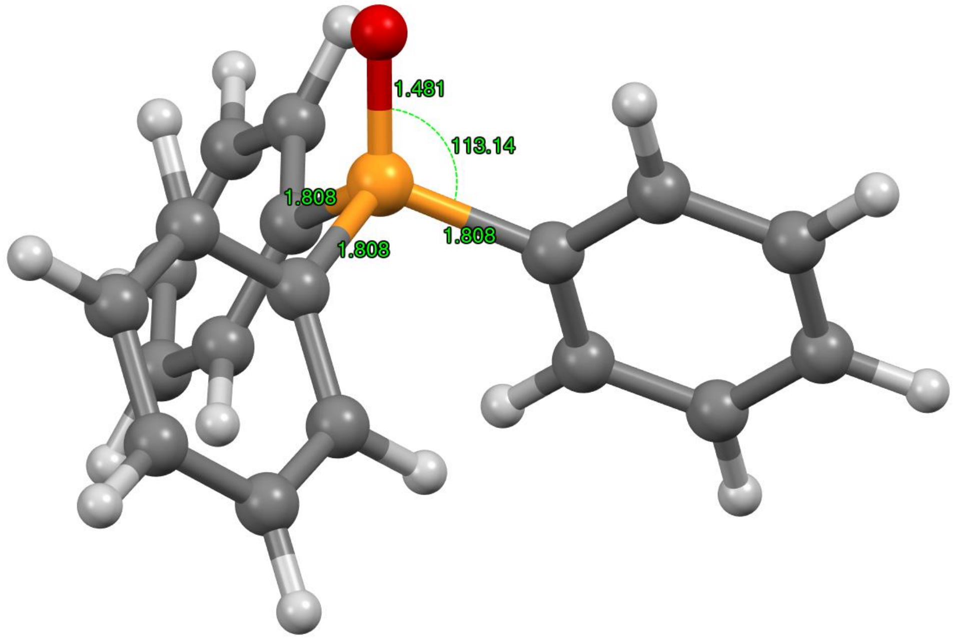


**P3=O**

Figure S7 (continued). Molecular structures of all intermediates and transition structures optimized with PBE0-D3/def2-TZVP. Distances are given in Å. Element colors are gray (C), white (H), red (O), orange (P), green (F).

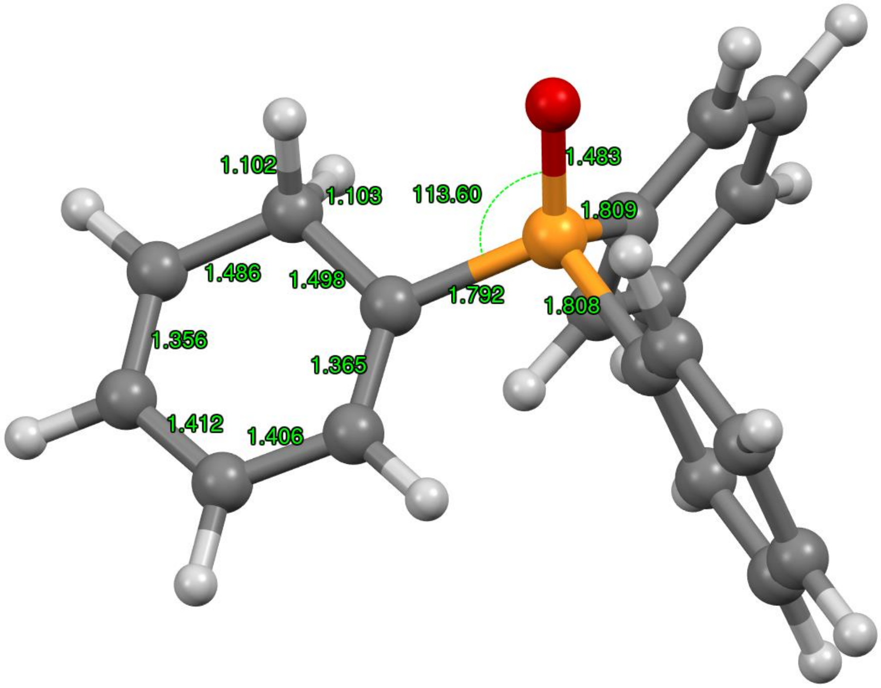


**P3-H$_{ortho}$**

Figure S7 (continued). Molecular structures of all intermediates and transition structures optimized with PBE0-D3/def2-TZVP. Distances are given in Å. Element colors are gray (C), white (H), red (O), orange (P), green (F).

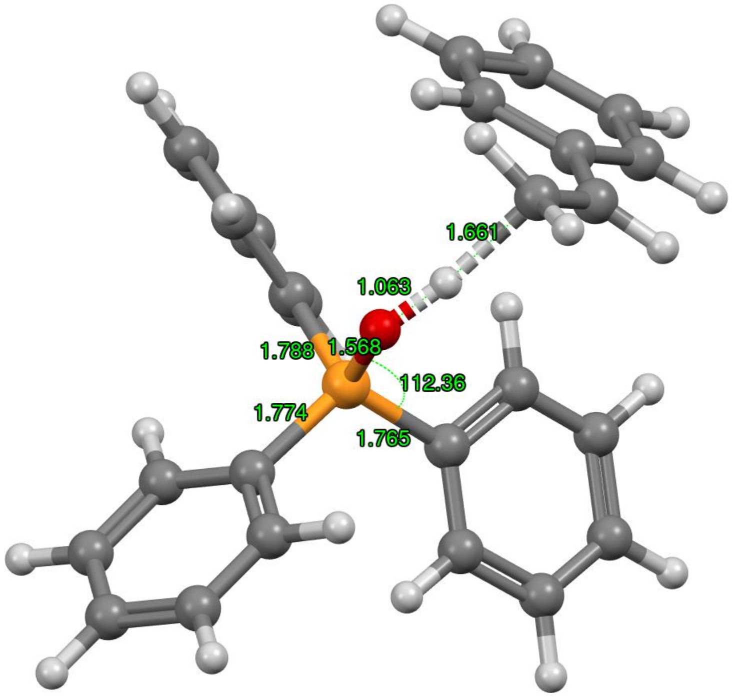


**P3-TS-STYR**

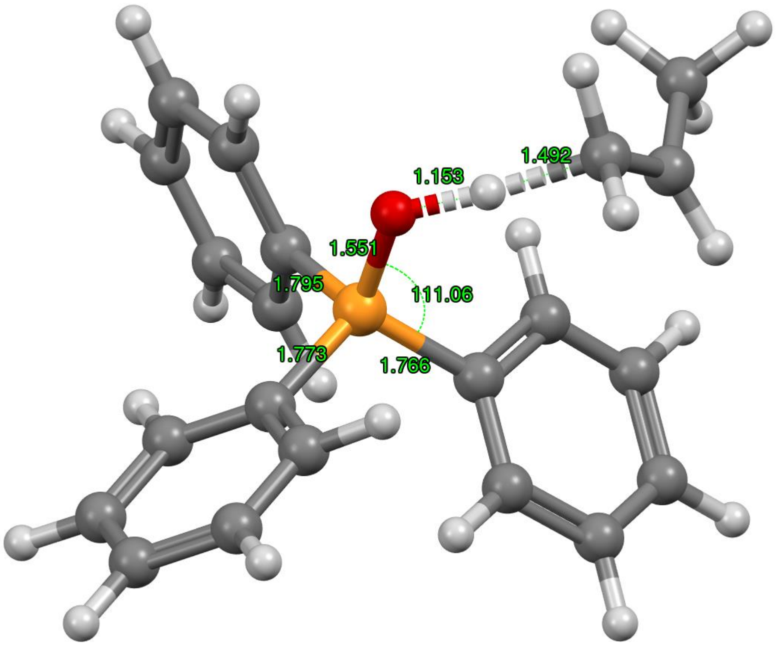


**P3-TS-PROP**

Figure S7 (continued). Molecular structures of all intermediates and transition structures optimized with PBE0-D3/def2-TZVP. Distances are given in Å. Element colors are gray (C), white (H), red (O), orange (P), green (F).

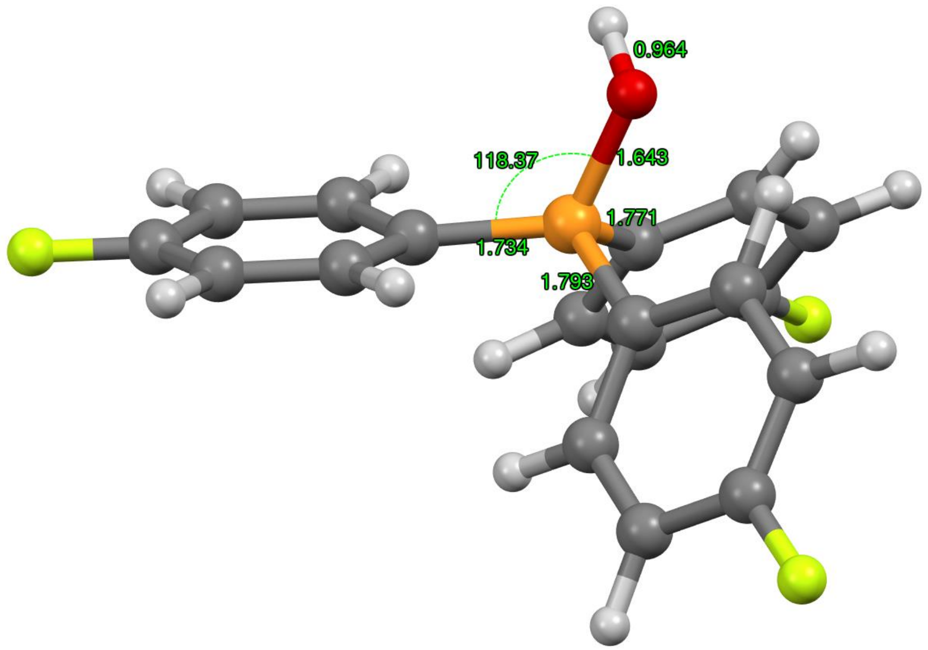


**P4-OH**

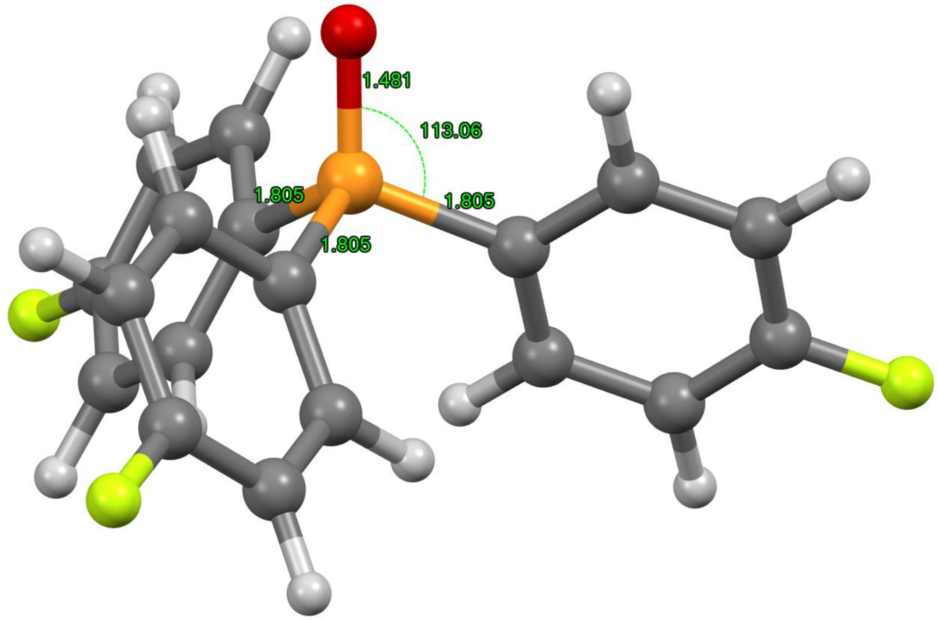


**P4=O**

Figure S7 (continued). Molecular structures of all intermediates and transition structures optimized with PBE0-D3/def2-TZVP. Distances are given in Å. Element colors are gray (C), white (H), red (O), orange (P), green (F).

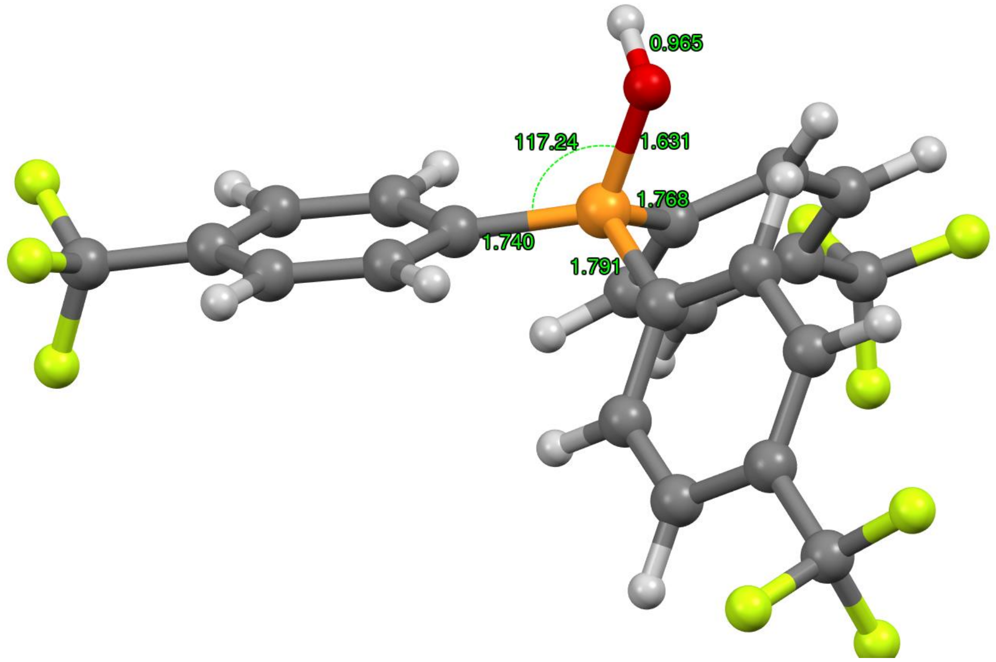


**P5-OH**

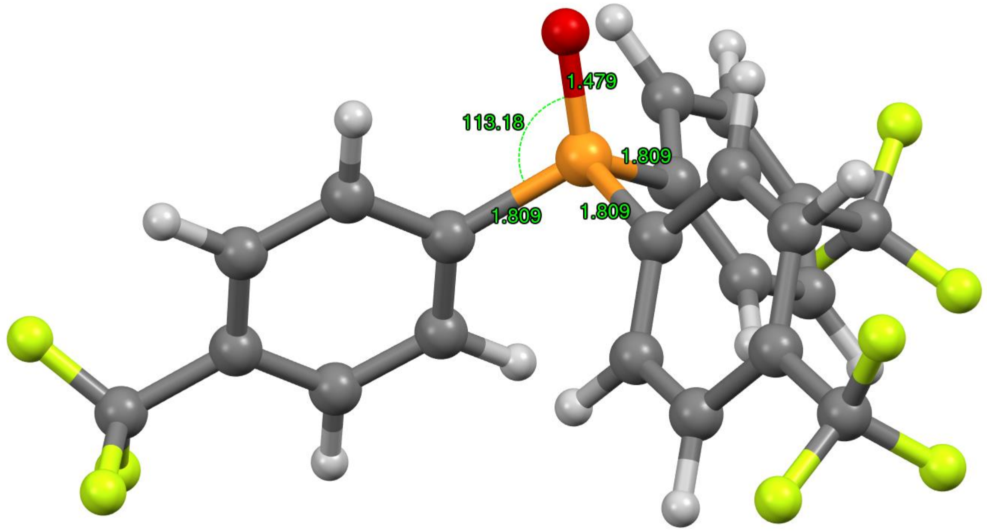


**P5=O**

Figure S7 (continued). Molecular structures of all intermediates and transition structures optimized with PBE0-D3/def2-TZVP. Distances are given in Å. Element colors are gray (C), white (H), red (O), orange (P), green (F).

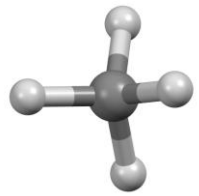

$CH_4$

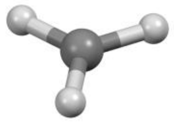

$CH_3^{\cdot}$

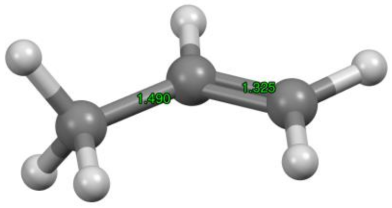


**PROPENE**

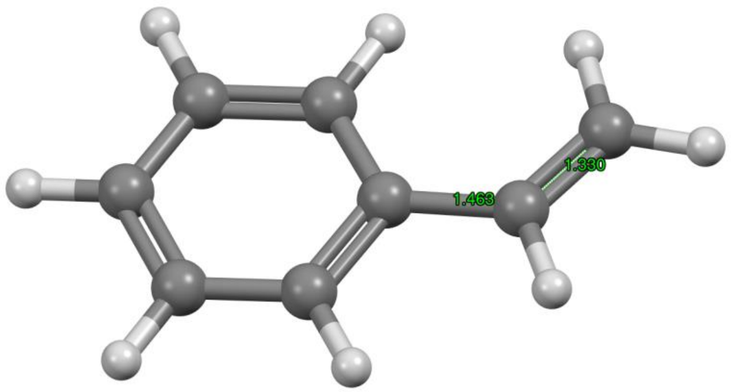


**STYRENE**

Figure S8. Spin density isosurfaces ($\rho_{SD} = \rho_{\alpha}-\rho_{\beta}$, $\rho_{SD}^{iso}$ = 0.005 a.u., PBE0-D3/def2-TZVP).

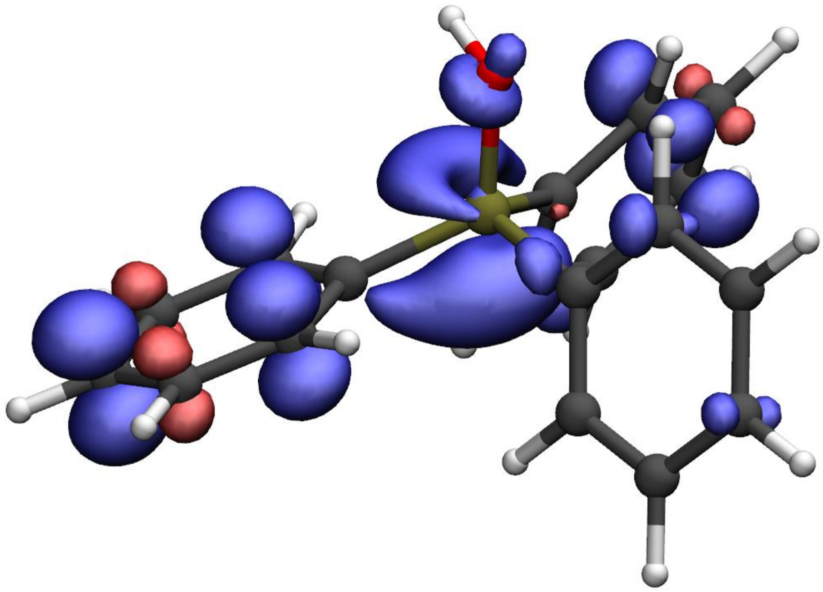

**P3-OH**

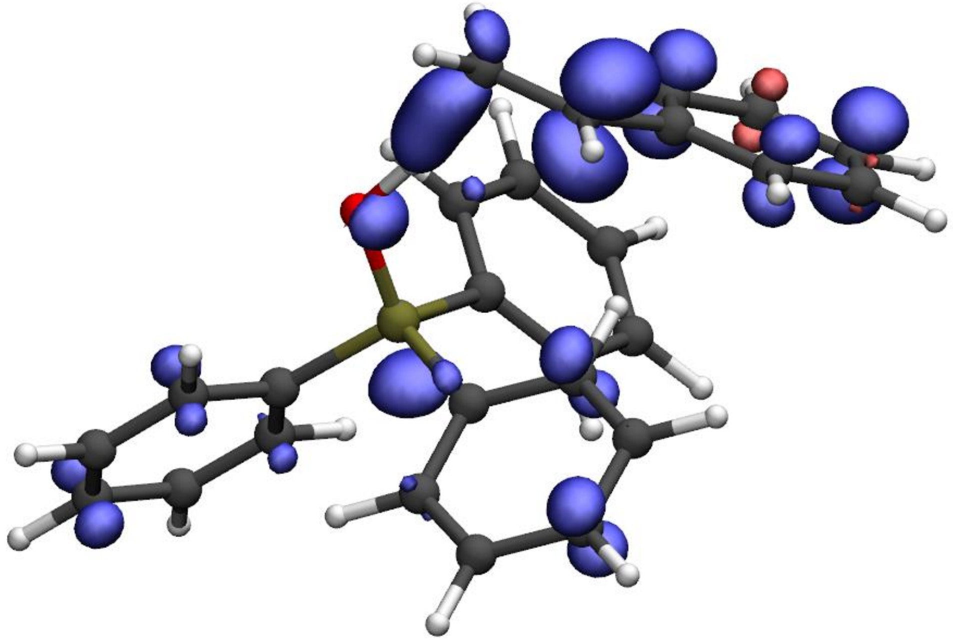

**P3-TS-STYR**

Figure S8 (continued). Spin density isosurfaces ($\rho_{SD} = \rho_{\alpha}-\rho_{\beta}$, $\rho_{SD}^{iso}$ = 0.005 a.u., PBE0-D3/def2-TZVP).

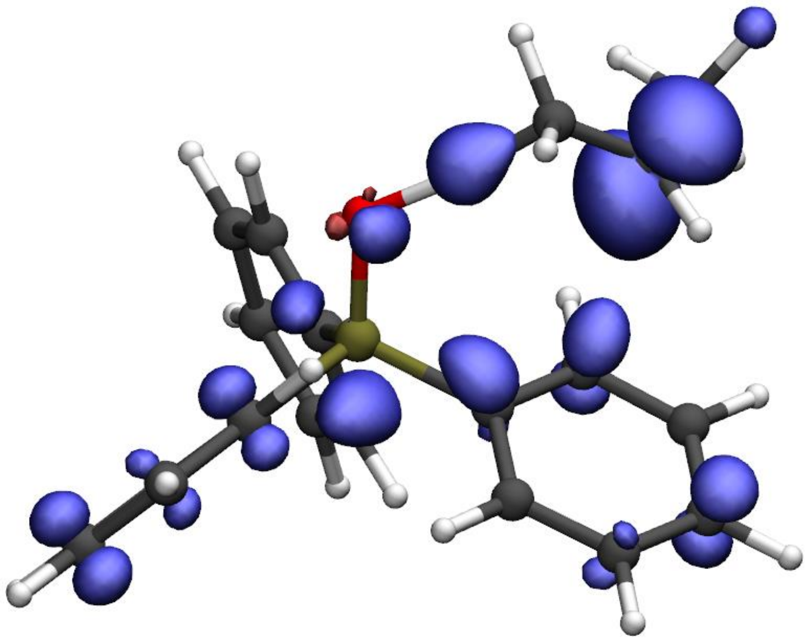

**P3-TS-PROP**

Figure S9. Gibbs free energies of the HAT from radical **P1**-OH to positions 2-8 of 1-methyl-naphthalene (**5f**). Transition state free energies (**5f-TS*n***) correspond to free energy barriers with respect to the separated **P1**-OH radical and **5f**.

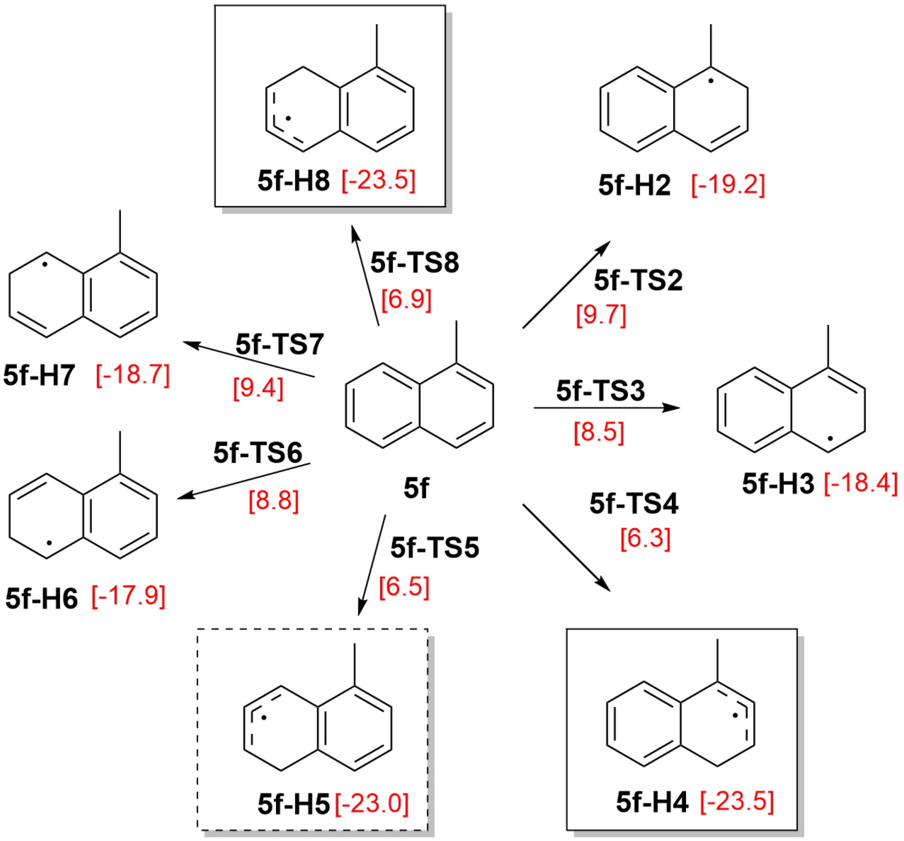


[$\Delta G^{s}_{298}$ / kcal mol$^{-1}$]
PWPB95-D3/def2-TZVP//PBE0-D3/def2-TZVP + COSMO-RS($CH_3CN$)

Figure S10. Gibbs free energies of the HAT from radical **P1**-OH to positions 2-8 of naphthalene-1-methylester (**5b**).

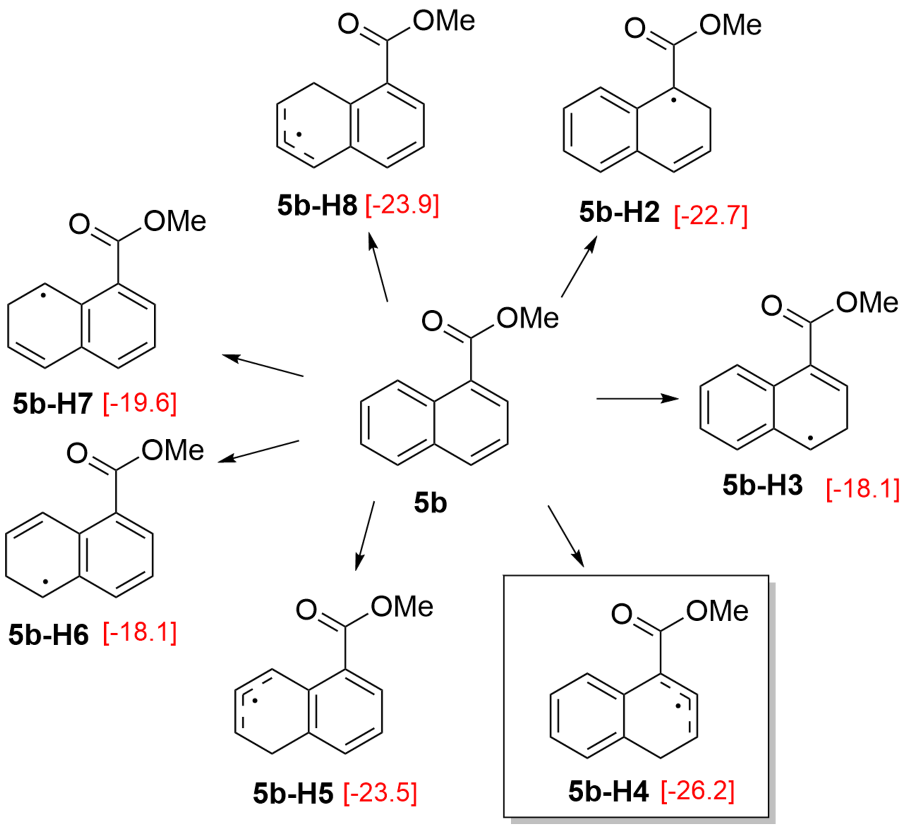


[$\Delta G^{s}_{298}$ / kcal mol$^{-1}$]
PWPB95-D3/def2-TZVP//PBE0-D3/def2-TZVP + COSMO-RS($CH_3CN$)

Figure S11. Gibbs free energies of the HAT from radical **P1**-OH to positions 1 and 3-8 of 2-cyano-naphthalene (**5c**).

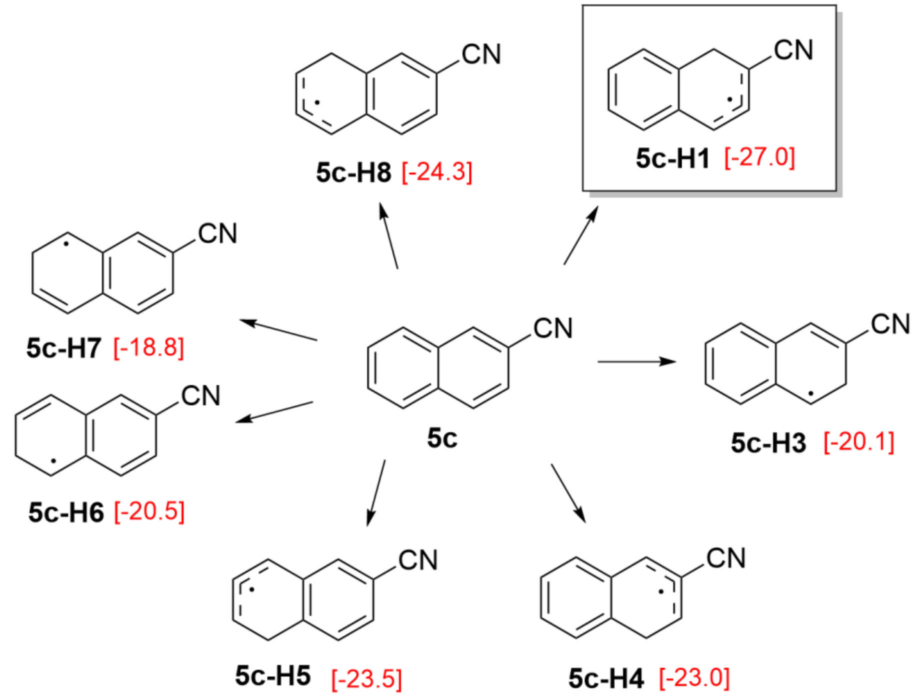


[$\Delta G^{s}_{298}$ / kcal mol$^{-1}$]

PWPB95-D3/def2-TZVP//PBE0-D3/def2-TZVP + COSMO-RS($CH_3CN$)

Figure S12. Molecular structures of the two most favorable transition structures of HAT from **P1-**OH to **5f**, optimized with PBE0-D3/def2-TZVP. Distances are given in Å. Element colors are gray (C), white (H), red (O), orange (P).

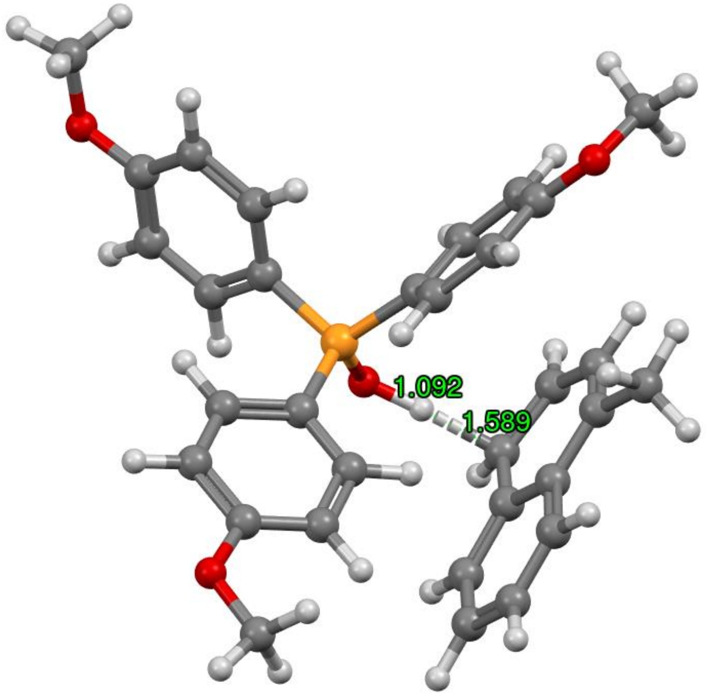


**5f-TS4**

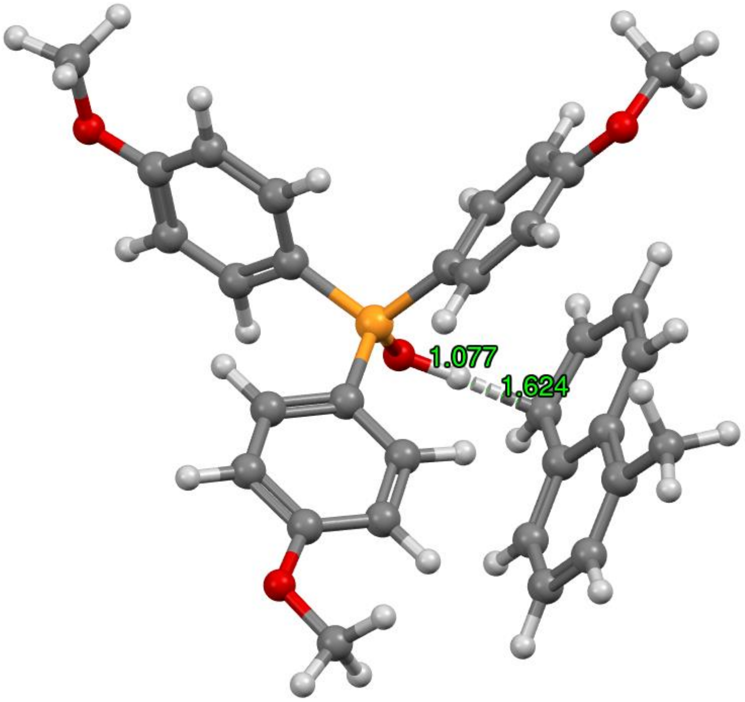


**5f-TS5**

## 12.4 DFT optimized (PBE0-D3/def2-TZVP) cartesian coordinates

**P1-OH**

E(PBE0-D3/def2-TZVP) = -1454.733077432 (conv)

Lowest Freq. = 19.71 cm^-1

48

P1-OH (053c1/opt)

| | | | |
|---|---|---|---|
| P | 1.1233471 | -0.1298920 | -0.0521003 |
| C | 0.6645890 | 1.3349228 | 0.8354575 |
| C | 1.3680507 | 2.5357125 | 0.6193588 |
| H | 2.2672142 | 2.5279084 | 0.0154303 |
| C | 0.9282979 | 3.7256067 | 1.1604557 |
| H | 1.4713309 | 4.6480365 | 0.9922369 |
| C | -0.2209362 | 3.7634128 | 1.9449895 |
| C | -0.9422345 | 2.5846293 | 2.1583676 |
| H | -1.8502661 | 2.5869638 | 2.7462089 |
| C | -0.5121527 | 1.4011672 | 1.5932485 |
| H | -1.1071920 | 0.5066239 | 1.7316081 |
| C | 0.5718853 | -1.5558852 | 0.7619883 |
| C | 0.4049334 | -2.7750481 | 0.0459028 |
| H | 0.5771062 | -2.7957180 | -1.0231922 |
| C | 0.0228477 | -3.9267345 | 0.6829465 |
| H | -0.1164494 | -4.8453277 | 0.1248758 |
| C | -0.1818657 | -3.9503146 | 2.0680686 |
| C | 0.0407002 | -2.7847556 | 2.8040330 |
| H | -0.0691305 | -2.7796313 | 3.8807199 |
| C | 0.4268799 | -1.6211256 | 2.1713895 |
| H | 0.6247854 | -0.7432045 | 2.7739963 |
| C | 0.6402549 | -0.1646770 | -1.7714144 |
| C | -0.6633938 | -0.5674793 | -2.0932578 |
| H | -1.3053701 | -0.9604109 | -1.3127018 |
| C | -1.1385332 | -0.4715990 | -3.3788508 |
| H | -2.1404889 | -0.7948638 | -3.6332245 |
| C | -0.3325499 | 0.0695961 | -4.3867834 |
| C | 0.9536935 | 0.4959517 | -4.0819752 |
| H | 1.5953745 | 0.9184926 | -4.8433115 |

| | | | |
|---|---|---|---|
| C | 1.4346527 | 0.3696027 | -2.7844289 |
| H | 2.4454275 | 0.6833817 | -2.5609553 |
| O | 2.7429152 | 0.0632263 | -0.2969020 |
| H | 3.1995150 | 0.1333396 | 0.5492185 |
| O | -0.5668503 | 4.9708008 | 2.4465449 |
| O | -0.5579554 | -5.1422260 | 2.5991094 |
| O | -0.8915142 | 0.1390161 | -5.6132032 |
| C | -1.7296198 | 5.0475789 | 3.2330362 |
| H | -1.8242091 | 6.0889160 | 3.5356441 |
| H | -1.6530076 | 4.4187376 | 4.1268423 |
| H | -2.6195170 | 4.7560825 | 2.6646436 |
| C | -0.7471349 | -5.2027754 | 3.9878812 |
| H | 0.1770736 | -4.9755179 | 4.5319180 |
| H | -1.0508546 | -6.2240457 | 4.2124840 |
| H | -1.5327293 | -4.5134254 | 4.3188849 |
| C | -0.1215705 | 0.6839402 | -6.6569453 |
| H | 0.1567431 | 1.7225609 | -6.4488104 |
| H | -0.7482646 | 0.6513761 | -7.5462124 |
| H | 0.7861726 | 0.0970736 | -6.8332200 |

**P1=O**

E(PBE0-D3/def2-TZVP) = -1454.211067460 (conv)

Lowest Freq. = 18.41 cm^-1

47

P1=O (061c1/opt)

| | | | |
|---|---|---|---|
| P | 1.2429435 | -0.0611013 | -0.0226846 |
| C | 0.6222379 | 1.3537838 | 0.9058321 |
| C | -0.5721451 | 2.0115511 | 0.6098263 |
| H | -1.1642811 | 1.6997901 | -0.2434777 |
| C | -1.0034651 | 3.0710057 | 1.3806161 |
| H | -1.9264430 | 3.5919004 | 1.1565128 |
| C | -0.2405927 | 3.5019003 | 2.4659699 |
| C | 0.9606423 | 2.8633114 | 2.7638224 |
| H | 1.5747943 | 3.1896393 | 3.5920523 |
| C | 1.3801113 | 1.7976013 | 1.9827392 |
| H | 2.3225748 | 1.3069344 | 2.1994461 |

| | | | |
|---|---|---|---|
| C | 0.4767874 | -1.5094781 | 0.7284925 |
| C | 1.1366254 | -2.7254800 | 0.5983432 |
| H | 2.0933926 | -2.7517289 | 0.0886332 |
| C | 0.6021337 | -3.8911045 | 1.1252790 |
| H | 1.1417064 | -4.8216416 | 1.0130839 |
| C | -0.6168879 | -3.8450306 | 1.7970510 |
| C | -1.2820071 | -2.6275451 | 1.9425414 |
| H | -2.2208666 | -2.6111620 | 2.4826651 |
| C | -0.7366448 | -1.4749929 | 1.4165278 |
| H | -1.2534332 | -0.5317378 | 1.5549249 |
| C | 0.5152823 | 0.0776541 | -1.6660274 |
| C | -0.7394147 | -0.4310118 | -2.0034763 |
| H | -1.3179231 | -0.9770215 | -1.2665169 |
| C | -1.2493078 | -0.2644865 | -3.2742345 |
| H | -2.2200274 | -0.6596434 | -3.5481370 |
| C | -0.5067858 | 0.4118434 | -4.2422189 |
| C | 0.7530018 | 0.9121578 | -3.9227250 |
| H | 1.3516243 | 1.4276435 | -4.6613417 |
| C | 1.2513672 | 0.7407949 | -2.6404654 |
| H | 2.2385376 | 1.1133006 | -2.3901253 |
| O | 2.7239488 | -0.1350566 | -0.0476119 |
| O | -0.7388376 | 4.5452466 | 3.1608142 |
| O | -1.2231638 | -4.9181153 | 2.3452568 |
| O | -1.0832732 | 0.5244790 | -5.4565439 |
| C | 0.0009387 | 5.0205098 | 4.2601834 |
| H | -0.5699090 | 5.8495618 | 4.6739519 |
| H | 0.9885093 | 5.3800005 | 3.9528621 |
| H | 0.1213991 | 4.2472395 | 5.0260953 |
| C | -0.5842351 | -6.1674162 | 2.2320729 |
| H | -0.4611761 | -6.4607088 | 1.1842739 |
| H | -1.2316579 | -6.8852924 | 2.7319909 |
| H | 0.3950006 | -6.1620624 | 2.7219948 |
| C | -0.3666651 | 1.1903869 | -6.4688727 |
| H | -0.1606250 | 2.2305247 | -6.1956221 |
| H | -1.0014245 | 1.1705071 | -7.3526759 |
| H | 0.5776334 | 0.6825498 | -6.6910980 |

**P2-OH**

E(PBE0-D3/def2-TZVP) = -1229.245313825 (conv)

Lowest Freq. = 25.83 cm^-1

45

P2-OH (018c1/opt)

| | | | |
|---|---|---|---|
| P | 1.0308729 | -0.0979339 | 0.1756812 |
| C | 0.4967698 | 1.3576553 | 1.0258113 |
| C | 1.2304321 | 2.5480227 | 0.8806777 |
| H | 2.1819358 | 2.5333285 | 0.3627454 |
| C | 0.7471258 | 3.7413716 | 1.3907760 |
| H | 1.3341450 | 4.6456294 | 1.2646694 |
| C | -0.4651820 | 3.8067301 | 2.0667794 |
| C | -1.2037438 | 2.6214268 | 2.1924334 |
| H | -2.1653697 | 2.6456825 | 2.6954674 |
| C | -0.7511835 | 1.4317131 | 1.6711835 |
| H | -1.3688599 | 0.5452017 | 1.7449707 |
| C | 0.3637336 | -1.5359280 | 0.8871070 |
| C | 0.2825687 | -2.7346610 | 0.1346358 |
| H | 0.5809620 | -2.7364832 | -0.9066045 |
| C | -0.1727475 | -3.8994487 | 0.7120058 |
| H | -0.2341223 | -4.7964300 | 0.1032570 |
| C | -0.5424157 | -3.9636150 | 2.0578702 |
| C | -0.4026946 | -2.7939317 | 2.8178883 |
| H | -0.6428852 | -2.8185927 | 3.8764965 |
| C | 0.0509472 | -1.6191530 | 2.2677345 |
| H | 0.1788841 | -0.7507571 | 2.9024226 |
| C | 0.7475843 | -0.0847943 | -1.5913540 |
| C | -0.5124489 | -0.4598445 | -2.0688206 |
| H | -1.2470084 | -0.8575056 | -1.3772746 |
| C | -0.8258955 | -0.3253764 | -3.4048875 |
| H | -1.8047590 | -0.6346215 | -3.7567264 |
| C | 0.0891633 | 0.2196917 | -4.3104390 |
| C | 1.3295595 | 0.6142588 | -3.8260336 |
| H | 2.0547927 | 1.0439062 | -4.5094978 |
| C | 1.6658757 | 0.4613054 | -2.4890081 |

| | | | |
|---|---|---|---|
| H | 2.6461494 | 0.7570391 | -2.1399249 |
| O | 2.6670954 | 0.0353908 | 0.1291109 |
| H | 3.0216340 | 0.1078296 | 1.0228362 |
| C | -0.9778101 | 5.0898318 | 2.6390686 |
| H | -1.9874046 | 5.3099300 | 2.2804875 |
| H | -0.3346137 | 5.9290848 | 2.3706672 |
| H | -1.0298042 | 5.0442917 | 3.7314947 |
| C | -1.0654114 | -5.2246890 | 2.6669340 |
| H | -0.7463512 | -6.1020156 | 2.1007250 |
| H | -2.1612564 | -5.2339009 | 2.6912403 |
| H | -0.7216826 | -5.3437232 | 3.6974968 |
| C | -0.2624275 | 0.3583927 | -5.7583585 |
| H | -0.2804537 | -0.6184237 | -6.2515521 |
| H | 0.4597640 | 0.9833685 | -6.2851890 |
| H | -1.2534637 | 0.8007462 | -5.8850040 |

**P2=O**

E(PBE0-D3/def2-TZVP) = -1228.717968701 (conv)

Lowest Freq. = 16.83 cm^-1

44

P2=O (058c1/opt)

| | | | |
|---|---|---|---|
| P | 1.4220512 | -0.0316862 | 0.1241210 |
| C | 0.6695207 | 1.3901371 | 0.9443001 |
| C | -0.4958001 | 2.0052577 | 0.5005559 |
| H | -0.9831246 | 1.6555755 | -0.4028805 |
| C | -1.0315814 | 3.0751118 | 1.2006552 |
| H | -1.9411998 | 3.5469002 | 0.8435776 |
| C | -0.4165504 | 3.5604441 | 2.3502584 |
| C | 0.7560357 | 2.9428858 | 2.7822863 |
| H | 1.2537806 | 3.3103964 | 3.6739185 |
| C | 1.2962150 | 1.8736816 | 2.0910468 |
| H | 2.2176738 | 1.4090732 | 2.4238989 |
| C | 0.6155099 | -1.4830309 | 0.8337408 |
| C | 1.3075249 | -2.6914695 | 0.7786006 |
| H | 2.3038538 | -2.7092633 | 0.3511201 |
| C | 0.7346705 | -3.8460554 | 1.2794691 |

H 1.2841252 -4.7807622 1.2311990

C -0.5370848 -3.8291246 1.8505257

C -1.2171560 -2.6171669 1.9077962

H -2.2046481 -2.5813323 2.3561024

C -0.6493314 -1.4543387 1.4098544

H -1.1911797 -0.5177510 1.4818226

C 0.8596453 0.0448135 -1.5900329

C -0.3309694 -0.5191478 -2.0340116

H -0.9604643 -1.0699672 -1.3438518

C -0.7116400 -0.3934218 -3.3612927

H -1.6428508 -0.8389401 -3.6956823

C 0.0860728 0.2874883 -4.2749864

C 1.2826272 0.8415400 -3.8223297

H 1.9219896 1.3712866 -4.5212404

C 1.6688294 0.7227504 -2.4998476

H 2.6089293 1.1418327 -2.1587409

O 2.8980947 -0.0643171 0.2520786

C -0.9776932 4.7359105 3.0886694

H -0.8965076 4.6012222 4.1691554

H -2.0275384 4.8981279 2.8406220

H -0.4323903 5.6497754 2.8339874

C -1.1376306 -5.0802869 2.4125689

H -0.6741068 -5.3374615 3.3696647

H -0.9854013 -5.9286229 1.7418230

H -2.2089261 -4.9662022 2.5831641

C -0.3101678 0.3993757 -5.7145682

H 0.2708585 -0.2923108 -6.3318738

H -0.1284440 1.4054941 -6.0984594

H -1.3656214 0.1635789 -5.8567852

**P3-OH**

E(PBE0-D3/def2-TZVP) = -1111.390124009 (conv)

Lowest Freq. = 28.03 cm^-1

36

P3-OH (011c1/opt)

P 0.7925257 -0.0821743 0.1355014

| | | | |
|---|---|---|---|
| C | 0.2564541 | 1.3804597 | 0.9758534 |
| C | 1.0034611 | 2.5639750 | 0.8272342 |
| H | 1.9569806 | 2.5355975 | 0.3137643 |
| C | 0.5292254 | 3.7622365 | 1.3318500 |
| H | 1.1202611 | 4.6626418 | 1.2075859 |
| C | -0.6862331 | 3.8162863 | 1.9987199 |
| H | -1.0502263 | 4.7546960 | 2.3988817 |
| C | -1.4429444 | 2.6521331 | 2.1350366 |
| H | -2.4029718 | 2.6857978 | 2.6373362 |
| C | -0.9933108 | 1.4569443 | 1.6160496 |
| H | -1.6129871 | 0.5719446 | 1.6886548 |
| C | 0.1195730 | -1.5189909 | 0.8472190 |
| C | 0.0454172 | -2.7159789 | 0.0894753 |
| H | 0.3438671 | -2.7111702 | -0.9517093 |
| C | -0.4074960 | -3.8835327 | 0.6636354 |
| H | -0.4683227 | -4.7802965 | 0.0569247 |
| C | -0.7747535 | -3.9282726 | 2.0075096 |
| H | -1.1233222 | -4.8516681 | 2.4525390 |
| C | -0.6520349 | -2.7708743 | 2.7795400 |
| H | -0.9029426 | -2.7958810 | 3.8342027 |
| C | -0.1994740 | -1.5948820 | 2.2271080 |
| H | -0.0801052 | -0.7214912 | 2.8563495 |
| C | 0.5086026 | -0.0620174 | -1.6335111 |
| C | -0.7528390 | -0.4343170 | -2.1078041 |
| H | -1.4854622 | -0.8338357 | -1.4154378 |
| C | -1.0668055 | -0.2951085 | -3.4459567 |
| H | -2.0428327 | -0.6016845 | -3.8033958 |
| C | -0.1396577 | 0.2519718 | -4.3278275 |
| H | -0.3917638 | 0.3735703 | -5.3747321 |
| C | 1.1048469 | 0.6444187 | -3.8615191 |
| H | 1.8305871 | 1.0708723 | -4.5445230 |
| C | 1.4358673 | 0.4848330 | -2.5241021 |
| H | 2.4160418 | 0.7716597 | -2.1677371 |
| O | 2.4277328 | 0.0429806 | 0.0916878 |
| H | 2.7850414 | 0.0991568 | 0.9855967 |

**P3=O**

E(PBE0-D3/def2-TZVP) = -1110.861463254 (conv)

Lowest Freq. = 24.75 cm^-1

35

P3=O (029c1/opt)

P 1.1406598 0.1260966 0.2030661

C 0.1402176 1.4425207 0.9340121

C 0.7288316 2.7011937 1.0341135

H 1.7552481 2.8298593 0.7093991

C 0.0123442 3.7626857 1.5624274

H 0.4748272 4.7396232 1.6420395

C -1.2929407 3.5733214 1.9953259

H -1.8515365 4.4040249 2.4111490

C -1.8794982 2.3192868 1.9077396

H -2.8936823 2.1674238 2.2585693

C -1.1642878 1.2542210 1.3810233

H -1.6173676 0.2703924 1.3319265

C 0.4678034 -1.4174401 0.8611687

C -0.5995616 -2.0958127 0.2801165

H -1.0474865 -1.7206907 -0.6332585

C -1.0788989 -3.2624595 0.8567485

H -1.9075467 -3.7899773 0.3987203

C -0.4906769 -3.7583519 2.0110846

H -0.8635256 -4.6725875 2.4584859

C 0.5823539 -3.0918487 2.5867850

H 1.0496622 -3.4857240 3.4818368

C 1.0624085 -1.9252404 2.0141435

H 1.9114773 -1.4041871 2.4420123

C 0.7286652 0.1231681 -1.5572301

C -0.4784558 0.5982086 -2.0614313

H -1.2137690 1.0291552 -1.3911464

C -0.7347728 0.5391795 -3.4231000

H -1.6743847 0.9139870 -3.8121442

C 0.2149265 0.0114800 -4.2856385

H 0.0150289 -0.0304205 -5.3502025

C 1.4254108 -0.4517481 -3.7884683

| | | | |
|---|---|---|---|
| H | 2.1722337 | -0.8529667 | -4.4636863 |
| C | 1.6838033 | -0.3957036 | -2.4285699 |
| H | 2.6319669 | -0.7364734 | -2.0280831 |
| O | 2.5905226 | 0.2858047 | 0.4610656 |

**P3-Hortho**

E(PBE0-D3/def2-TZVP) = -1111.412034368 (conv)

Lowest Freq. = 24.50 cm^-1

36

P3-Hortho (017c1/opt)

| | | | |
|---|---|---|---|
| P | 1.0922943 | 0.2354984 | -0.0190488 |
| C | 0.0929899 | 1.5068525 | 0.7927115 |
| C | 0.6442240 | 2.7785585 | 0.9218837 |
| H | 1.6525164 | 2.9515004 | 0.5627374 |
| C | -0.0860721 | 3.7940427 | 1.5187534 |
| H | 0.3460099 | 4.7829710 | 1.6191169 |
| C | -1.3658337 | 3.5433023 | 1.9940682 |
| H | -1.9342877 | 4.3377662 | 2.4638715 |
| C | -1.9147502 | 2.2739388 | 1.8796036 |
| H | -2.9088258 | 2.0745234 | 2.2628363 |
| C | -1.1864981 | 1.2567928 | 1.2818410 |
| H | -1.6067066 | 0.2588518 | 1.2074753 |
| C | 0.5956798 | -1.2972720 | 0.7645161 |
| C | -0.1013080 | -2.2824310 | 0.1257835 |
| H | -0.4019396 | -2.1380478 | -0.9064185 |
| C | -0.4406074 | -3.4840439 | 0.7720465 |
| H | -0.9954463 | -4.2453532 | 0.2388746 |
| C | -0.0553429 | -3.6929613 | 2.1143258 |
| H | -0.3267092 | -4.6216202 | 2.6044207 |
| C | 0.6493349 | -2.7557608 | 2.7959280 |
| H | 0.9414655 | -2.9339104 | 3.8250751 |
| C | 1.0564069 | -1.4660277 | 2.1794693 |
| H | 0.6983002 | -0.6239029 | 2.7957482 |
| C | 0.5114057 | 0.1547513 | -1.7287637 |
| C | -0.8166809 | 0.3485293 | -2.0989183 |
| H | -1.5608367 | 0.5883512 | -1.3479502 |

C -1.1883616 0.2508874 -3.4303437
H -2.2225277 0.4060053 -3.7151685
C -0.2350093 -0.0349873 -4.3981071
H -0.5268167 -0.1074166 -5.4395772
C 1.0914495 -0.2176156 -4.0351405
H 1.8376506 -0.4309950 -4.7916678
C 1.4659178 -0.1220019 -2.7036368
H 2.4997111 -0.2459830 -2.4016448
O 2.5471806 0.5080691 0.0781905
H 2.1520237 -1.3508616 2.2171087

**P3-TS-Styr**

E(PBE0-D3/def2-TZVP) = -1420.791573061 (conv)

Lowest Freq. = -902.17 cm^-1

52

P3-TS-Styr (023TSc3/opt)

P -1.0124655 0.6443415 0.5182938
C -2.7358228 1.0341306 0.6760940
C -3.2192813 1.5082601 1.9027880
H -2.5302648 1.6824239 2.7209593
C -4.5677036 1.7757293 2.0585280
H -4.9343343 2.1413042 3.0106782
C -5.4458161 1.5942059 0.9978293
H -6.4997043 1.8128923 1.1216510
C -4.9668412 1.1407575 -0.2266585
H -5.6471357 1.0033603 -1.0589308
C -3.6234133 0.8572713 -0.3891120
H -3.2557401 0.4981643 -1.3430251
C -0.6363582 -0.8732254 1.3372150
C 0.5911762 -1.5015593 1.0689066
H 1.2793231 -1.0805307 0.3488306
C 0.9203789 -2.6792046 1.7198255
H 1.8688976 -3.1547436 1.4982151
C 0.0509966 -3.2404274 2.6417251
H 0.3151895 -4.1602627 3.1498678
C -1.1712346 -2.6206066 2.9097666

| | | | |
|---|---|---|---|
| H | -1.8588551 | -3.0611992 | 3.6221109 |
| C | -1.5150292 | -1.4505327 | 2.2664496 |
| H | -2.4718575 | -0.9843917 | 2.4681654 |
| C | -0.6057046 | 0.5106015 | -1.2175985 |
| C | -0.5488703 | -0.7180767 | -1.8725934 |
| H | -0.6980313 | -1.6338210 | -1.3137763 |
| C | -0.2891951 | -0.7657390 | -3.2312839 |
| H | -0.2312582 | -1.7234416 | -3.7340975 |
| C | -0.0975534 | 0.4105697 | -3.9448199 |
| H | 0.1083612 | 0.3702042 | -5.0081453 |
| C | -0.1648263 | 1.6367323 | -3.2989826 |
| H | -0.0132741 | 2.5539877 | -3.8557662 |
| C | -0.4152994 | 1.6910173 | -1.9379429 |
| H | -0.4495803 | 2.6439033 | -1.4228661 |
| O | -0.2433114 | 1.8623335 | 1.1366165 |
| H | 0.8003625 | 1.7917334 | 1.3269920 |
| H | 4.1496624 | -1.7766574 | -3.1299196 |
| C | 3.9271628 | -1.2526262 | -2.2082793 |
| C | 4.2632119 | -1.8135423 | -0.9766207 |
| C | 3.3032739 | -0.0106214 | -2.2348242 |
| C | 3.0141291 | 0.6616836 | -1.0631154 |
| C | 3.9754142 | -1.1475853 | 0.1963245 |
| C | 3.3388097 | 0.1093966 | 0.1928942 |
| H | 3.0280432 | 0.4357900 | -3.1840141 |
| H | 4.7551603 | -2.7793673 | -0.9380471 |
| H | 2.5099459 | 1.6188643 | -1.1126424 |
| H | 4.2361620 | -1.5950035 | 1.1505817 |
| C | 2.9964073 | 0.7482728 | 1.4289517 |
| C | 2.4294915 | 1.9949219 | 1.5769618 |
| H | 3.1714557 | 0.1560688 | 2.3237366 |
| H | 2.4626870 | 2.7121917 | 0.7610296 |
| H | 2.3530595 | 2.4220519 | 2.5710734 |

**P3-TS-Prop**

E(PBE0-D3/def2-TZVP) = -1229.184121491 (conv)

Lowest Freq. = -1787.47 cm^-1

45

P3-TS-Prop (033TSc1/opt)

| | | | |
|---|---|---|---|
| P | -0.3739772 | 0.5967447 | -0.0544471 |
| C | -2.0761663 | 0.9578774 | 0.2841329 |
| C | -2.4072955 | 1.4533766 | 1.5540774 |
| H | -1.6243958 | 1.6292009 | 2.2833551 |
| C | -3.7241547 | 1.7373885 | 1.8683273 |
| H | -3.9693965 | 2.1164764 | 2.8537819 |
| C | -4.7261537 | 1.5542818 | 0.9240517 |
| H | -5.7554238 | 1.7853890 | 1.1709380 |
| C | -4.4001201 | 1.0844334 | -0.3446229 |
| H | -5.1763768 | 0.9482056 | -1.0886567 |
| C | -3.0893237 | 0.7854980 | -0.6649195 |
| H | -2.8446436 | 0.4181068 | -1.6542779 |
| C | 0.1299938 | -0.8633361 | 0.8009514 |
| C | 1.3507582 | -1.4609842 | 0.4258322 |
| H | 1.9483467 | -1.0258242 | -0.3656964 |
| C | 1.7408416 | -2.6603574 | 1.0100796 |
| H | 2.6650148 | -3.1315564 | 0.6937676 |
| C | 0.9735237 | -3.2416176 | 2.0014997 |
| H | 1.2920042 | -4.1690796 | 2.4624345 |
| C | -0.2243104 | -2.6340109 | 2.4023931 |
| H | -0.8321627 | -3.0891719 | 3.1752888 |
| C | -0.6457491 | -1.4685429 | 1.8005724 |
| H | -1.5913725 | -1.0242571 | 2.0880240 |
| C | -0.1945530 | 0.2921908 | -1.8146382 |
| C | -0.5130507 | -0.9488966 | -2.3625828 |
| H | -0.8241310 | -1.7582199 | -1.7116063 |
| C | -0.4219637 | -1.1457090 | -3.7310896 |
| H | -0.6718304 | -2.1109159 | -4.1555993 |
| C | -0.0027049 | -0.1091897 | -4.5542871 |
| H | 0.0712498 | -0.2657849 | -5.6243576 |
| C | 0.3299762 | 1.1230849 | -4.0108267 |
| H | 0.6645479 | 1.9286257 | -4.6538281 |
| C | 0.2350987 | 1.3258572 | -2.6422208 |
| H | 0.4975567 | 2.2807097 | -2.2023570 |

O 0.5172421 1.8056283 0.3333669
H 1.4348335 1.6013267 1.0006765
C 3.2487127 0.3178187 1.7385061
C 2.6203290 1.5398729 1.9041567
H 2.9957491 -0.5037008 2.3989584
H 3.0846635 2.4196368 1.4574919
H 2.0899204 1.7256581 2.8353446
C 4.3243302 0.0870747 0.7395476
H 4.3487670 -0.9468937 0.3867547
H 5.3144024 0.2977176 1.1685140
H 4.2113937 0.7458678 -0.1268107

**P4-OH**

E(PBE0-D3/def2-TZVP) = -1408.968422356 (conv)

Lowest Freq. = 25.46 cm^-1

36

P4-OH (037c1/opt)

P 0.8127821 -0.0700909 0.1371956
C 0.2680092 1.3853068 0.9858968
C 1.0085673 2.5741680 0.8555332
H 1.9675279 2.5574758 0.3528417
C 0.5273579 3.7710428 1.3600131
H 1.0920390 4.6900587 1.2634263
C -0.6911229 3.7823260 2.0062895
C -1.4588882 2.6321085 2.1406826
H -2.4206614 2.6850191 2.6352782
C -0.9880867 1.4498500 1.6142420
H -1.6035210 0.5610481 1.6745379
C 0.1485480 -1.5067407 0.8452952
C 0.0661241 -2.7037400 0.0829439
H 0.3719374 -2.7024649 -0.9556451
C -0.4051547 -3.8708867 0.6429254
H -0.4818770 -4.7788122 0.0571232
C -0.7735457 -3.8860553 1.9790137
C -0.6465283 -2.7557686 2.7780443
H -0.9043872 -2.8125154 3.8285372

C -0.1735643 -1.5883234 2.2269620
H -0.0417754 -0.7229675 2.8642819
C 0.5235982 -0.0583644 -1.6320890
C -0.7502377 -0.4051666 -2.0916291
H -1.4882572 -0.7734692 -1.3878884
C -1.0802699 -0.2857647 -3.4267309
H -2.0585641 -0.5636083 -3.7976773
C -0.1307496 0.2197771 -4.3006652
C 1.1288505 0.5925782 -3.8798669
H 1.8400096 0.9832283 -4.5968781
C 1.4576245 0.4441759 -2.5407953
H 2.4486177 0.7099999 -2.1993995
O 2.4474008 0.0852118 0.0737342
H 2.8208748 0.1025630 0.9624777
F -1.1572817 4.9320237 2.5078912
F -1.2245955 -5.0259244 2.5252143
F -0.4508006 0.3527017 -5.5911167

**P4=O**

E(PBE0-D3/def2-TZVP) = -1408.442921322 (conv)

Lowest Freq. = 24.36 cm^-1

35

P4=O (059c1/opt)

P 1.1583542 -0.0715982 0.1278489
C 0.4497878 1.4046684 0.8879480
C 1.2436304 2.5502181 0.9090242
H 2.2496198 2.5025405 0.5078646
C 0.7647881 3.7286972 1.4540330
H 1.3664195 4.6283590 1.4847768
C -0.5151569 3.7421075 1.9771919
C -1.3232377 2.6212688 1.9822841
H -2.3129934 2.6749827 2.4178376
C -0.8292635 1.4478847 1.4351581
H -1.4441056 0.5551672 1.4502143
C 0.2805423 -1.4667935 0.8638550
C -0.9205805 -1.9661417 0.3685112

H -1.3596820 -1.5374900 -0.5251743
C -1.5572539 -3.0243632 0.9970908
H -2.4892217 -3.4311963 0.6254508
C -0.9689932 -3.5742462 2.1201057
C 0.2302918 -3.1106973 2.6290049
H 0.6601135 -3.5824889 3.5036197
C 0.8532435 -2.0516936 1.9920328
H 1.8034575 -1.6773188 2.3554945
C 0.6398754 -0.0250746 -1.6007182
C 1.4315294 -0.7132142 -2.5186518
H 2.3290672 -1.2123537 -2.1714858
C 1.0912449 -0.7417721 -3.8597345
H 1.6947725 -1.2658986 -4.5900204
C -0.0480820 -0.0718798 -4.2662945
C -0.8482436 0.6284886 -3.3838722
H -1.7223567 1.1514416 -3.7508135
C -0.4935517 0.6500436 -2.0444855
H -1.0999176 1.2125281 -1.3436495
O 2.6276790 -0.1599519 0.2901042
F -0.9839723 4.8756815 2.5052514
F -1.5759024 -4.5960174 2.7294630
F -0.3819019 -0.0938876 -5.5592655

**P5-OH**

E(PBE0-D3/def2-TZVP) = -2122.041959222 (conv)

Lowest Freq. = 7.66 cm^-1

45

P5-OH (048c1/opt)

P 1.0525254 -0.0982433 0.1885241
C 0.4943039 1.3589905 1.0195604
C 1.2313075 2.5472659 0.8709735
H 2.1873978 2.5297848 0.3625459
C 0.7477064 3.7425648 1.3695396
H 1.3262707 4.6497486 1.2514392
C -0.4732328 3.7818071 2.0260645
C -1.2262200 2.6120645 2.1620026

| | | | |
|---|---|---|---|
| H | -2.1908739 | 2.6461800 | 2.6541643 |
| C | -0.7614789 | 1.4235559 | 1.6509311 |
| H | -1.3766000 | 0.5357215 | 1.7218190 |
| C | 0.3784178 | -1.5384863 | 0.8948926 |
| C | 0.3184040 | -2.7328597 | 0.1350636 |
| H | 0.6259560 | -2.7288356 | -0.9031163 |
| C | -0.1310071 | -3.9034697 | 0.7004228 |
| H | -0.1818466 | -4.8031504 | 0.0999735 |
| C | -0.5038585 | -3.9458065 | 2.0433326 |
| C | -0.3971939 | -2.7888087 | 2.8223107 |
| H | -0.6520977 | -2.8242918 | 3.8745184 |
| C | 0.0510435 | -1.6140675 | 2.2716536 |
| H | 0.1609095 | -0.7419971 | 2.9039086 |
| C | 0.7628374 | -0.0777272 | -1.5793198 |
| C | -0.5018389 | -0.4486596 | -2.0484639 |
| H | -1.2374759 | -0.8420234 | -1.3564133 |
| C | -0.8167228 | -0.3172429 | -3.3835277 |
| H | -1.7911346 | -0.6225657 | -3.7452645 |
| C | 0.1177470 | 0.2202054 | -4.2668720 |
| C | 1.3639996 | 0.6125663 | -3.8097280 |
| H | 2.0872207 | 1.0267133 | -4.5001579 |
| C | 1.6910946 | 0.4602617 | -2.4709336 |
| H | 2.6736471 | 0.7451354 | -2.1203858 |
| O | 2.6782365 | 0.0240177 | 0.1506249 |
| H | 3.0494876 | 0.0639703 | 1.0398616 |
| C | -1.0037334 | 5.0549075 | 2.6052080 |
| C | -1.0390493 | -5.2000786 | 2.6479204 |
| C | -0.2634045 | 0.3723771 | -5.7098462 |
| F | -2.3867039 | -5.2139197 | 2.6712849 |
| F | -0.6405928 | -5.3559936 | 3.9199463 |
| F | -0.6582553 | -6.2924826 | 1.9730449 |
| F | -1.0361781 | 5.0119935 | 3.9488529 |
| F | -2.2617476 | 5.2966777 | 2.2045351 |
| F | -0.2665225 | 6.1169217 | 2.2646424 |
| F | -0.6556172 | -0.7964877 | -6.2384921 |
| F | -1.2891596 | 1.2242800 | -5.8586752 |

F 0.7440330 0.8294862 -6.4583657

**P5=O**

E(PBE0-D3/def2-TZVP) = -2121.506149093 (conv)

Lowest Freq. = 2.37 cm^-1

44

P5=O (060c1/opt)

| | | | |
|---|---|---|---|
| P | 1.4215354 | -0.1328199 | 0.0707467 |
| C | 0.7939180 | 1.3548329 | 0.8866367 |
| C | 1.5200639 | 1.8257715 | 1.9779619 |
| H | 2.4313060 | 1.3156010 | 2.2680923 |
| C | 1.0911365 | 2.9470160 | 2.6652187 |
| H | 1.6577985 | 3.3199836 | 3.5093478 |
| C | -0.0635028 | 3.6036468 | 2.2599874 |
| C | -0.7856334 | 3.1486624 | 1.1670659 |
| H | -1.6738500 | 3.6779122 | 0.8464107 |
| C | -0.3537306 | 2.0264366 | 0.4790436 |
| H | -0.9064776 | 1.6867401 | -0.3891687 |
| C | 0.5421773 | -1.5137244 | 0.8406888 |
| C | 1.1796071 | -2.7511307 | 0.8227453 |
| H | 2.1685125 | -2.8314544 | 0.3864254 |
| C | 0.5636291 | -3.8587953 | 1.3798367 |
| H | 1.0603405 | -4.8206379 | 1.3736092 |
| C | -0.6895487 | -3.7289003 | 1.9613913 |
| C | -1.3270684 | -2.4964382 | 1.9938324 |
| H | -2.2961928 | -2.4003722 | 2.4673922 |
| C | -0.7094678 | -1.3898553 | 1.4365238 |
| H | -1.1984867 | -0.4237995 | 1.4848406 |
| C | 0.8049610 | -0.0476954 | -1.6277487 |
| C | -0.4463045 | -0.5160413 | -2.0145780 |
| H | -1.0976767 | -0.9951276 | -1.2927614 |
| C | -0.8595008 | -0.3930515 | -3.3311594 |
| H | -1.8295502 | -0.7643404 | -3.6363220 |
| C | -0.0160935 | 0.1926369 | -4.2635143 |
| C | 1.2404723 | 0.6496241 | -3.8886746 |
| H | 1.8989259 | 1.0894271 | -4.6273481 |

C 1.6508128 0.5273697 -2.5731430
H 2.6369748 0.8591727 -2.2695473
O 2.8925580 -0.2637124 0.1500997
C -0.5489620 4.7928826 3.0396849
C -1.3859557 -4.9327313 2.5307401
C -0.4759798 0.3719650 -5.6828688
F -2.2297370 -5.4723308 1.6367728
F -2.1114489 -4.6224712 3.6115113
F -0.5254947 -5.8912750 2.8857190
F -1.2781907 4.4114986 4.1002977
F -1.3219853 5.5964221 2.3020759
F 0.4646660 5.5288559 3.5081348
F -1.4002131 -0.5300913 -6.0290004
F -1.0232499 1.5842143 -5.8651801
F 0.5349061 0.2661242 -6.5518185

**CH4**

E(PBE0-D3/def2-TZVP) = -40.47511350880 (conv)

Lowest Freq. = 1326.81 cm^-1

5

CH4 (CH4/opt)

H 0.0000000 0.0000000 -1.0887801
C -0.0000000 0.0000000 0.0007861
H -0.5136301 -0.8896334 0.3639731
H -0.5136301 0.8896334 0.3639731
H 1.0272601 0.0000000 0.3639731

**CH3**

E(PBE0-D3/def2-TZVP) = -39.79976187783 (conv)

Lowest Freq. = 526.63 cm^-1

4

CH3 (CH3/opt)

C 0.0000000 0.0000000 0.0076124
H -0.5400054 -0.9353167 0.0081699
H -0.5400054 0.9353167 0.0081699
H 1.0800107 0.0000000 0.0081699

**PROP**

E(PBE0-D3/def2-TZVP) = -117.8005413712 (conv)

Lowest Freq. = 208.44 cm^-1

9

PROP (PROP/opt)

| | | | |
|---|---|---|---|
| H | 0.4631268 | -0.9542626 | -1.2205238 |
| C | 0.4121970 | 0.1222078 | -1.0469924 |
| H | -0.2191363 | 0.5661093 | -1.8231063 |
| H | 1.4145821 | 0.5402320 | -1.1829146 |
| C | -0.1152576 | 0.4308312 | 0.3115215 |
| C | -0.4816472 | -0.4720072 | 1.2100400 |
| H | -0.1982450 | 1.4858321 | 0.5661920 |
| H | -0.8586980 | -0.1832182 | 2.1840626 |
| H | -0.4169217 | -1.5357243 | 1.0017211 |

**STYR**

E(PBE0-D3/def2-TZVP) = -309.3903523134 (conv)

Lowest Freq. = 33.82 cm^-1

16

STYR (STYR/opt)

| | | | |
|---|---|---|---|
| H | 1.3309472 | -3.1770125 | -0.2468929 |
| C | 0.9641187 | -2.1598721 | -0.1746390 |
| C | 1.8451441 | -1.0924654 | -0.2394718 |
| C | -0.3950291 | -1.9164617 | -0.0162321 |
| C | -0.8675150 | -0.6208111 | 0.0763594 |
| C | 1.3686427 | 0.2055883 | -0.1464407 |
| C | 0.0074105 | 0.4667099 | 0.0129691 |
| H | -1.0906268 | -2.7462781 | 0.0352256 |
| H | 2.9073302 | -1.2695702 | -0.3629131 |
| H | -1.9306523 | -0.4513214 | 0.1993354 |
| H | 2.0613162 | 1.0393606 | -0.1976199 |
| C | -0.4392666 | 1.8570800 | 0.1059296 |
| C | -1.6861334 | 2.2938742 | 0.2612986 |
| H | 0.3581260 | 2.5936456 | 0.0375760 |
| H | -1.8990889 | 3.3539212 | 0.3177317 |

H -2.5347238 1.6236127 0.3377841

**5f**

E(PBE0-D3/def2-TZVP) = -424.8637356965 (conv)

Lowest Freq. = 133.67 cm^-1

21

5f (094c1/opt)

C 2.5961481 1.2108281 -0.0729700

C 2.6031426 -0.1950986 -0.0828146

C 1.4262236 -0.8936268 -0.0507032

C 0.1811369 -0.2272194 -0.0071285

C 0.1778572 1.1958826 0.0024965

C 1.4082790 1.8870580 -0.0312489

C -1.0531621 1.8866181 0.0453429

C -2.2294778 1.1951248 0.0774079

C -2.2247116 -0.2120131 0.0681978

C -1.0568573 -0.9301205 0.0270289

H 3.5331319 1.7549127 -0.0985556

H 3.5468278 -0.7272817 -0.1160389

H 1.4482450 -1.9763437 -0.0588539

H 1.3931170 2.9718791 -0.0234278

H -1.0453022 2.9712124 0.0521809

H -3.1745868 1.7247762 0.1102356

H -3.1701822 -0.7435977 0.0943113

C -1.0847119 -2.4264163 0.0179736

H -0.5521849 -2.8434301 0.8776002

H -0.6114971 -2.8315472 -0.8811519

H -2.1114353 -2.7915971 0.0501178

**5f-TS2**

E(PBE0-D3/def2-TZVP) = -1879.606496561 (conv)

Lowest Freq. = -1104.64 cm^-1

69

5f-TS2 (105TSc1/opt)

P -0.1491301 -0.4502966 -1.1652548

C 0.0499028 1.1963437 -0.5348252

| | | | |
|---|---|---|---|
| C | 0.9274996 | 2.0646255 | -1.1956864 |
| H | 1.5025965 | 1.7103477 | -2.0423647 |
| C | 1.0819140 | 3.3596157 | -0.7570534 |
| H | 1.7720600 | 4.0379806 | -1.2430031 |
| C | 0.3839778 | 3.8086359 | 0.3638144 |
| C | -0.5125679 | 2.9620474 | 1.0134562 |
| H | -1.0594973 | 3.2890951 | 1.8857729 |
| C | -0.6702353 | 1.6656174 | 0.5598774 |
| H | -1.3576570 | 1.0081302 | 1.0764849 |
| C | -1.0232737 | -1.4171416 | 0.0373673 |
| C | -2.1327782 | -2.1965023 | -0.3067970 |
| H | -2.5119925 | -2.1775212 | -1.3218166 |
| C | -2.7526037 | -2.9858660 | 0.6344119 |
| H | -3.6132746 | -3.5923635 | 0.3812453 |
| C | -2.2749947 | -3.0158972 | 1.9492105 |
| C | -1.1784264 | -2.2370129 | 2.3058129 |
| H | -0.7997487 | -2.2311661 | 3.3188377 |
| C | -0.5572116 | -1.4450042 | 1.3530774 |
| H | 0.2979079 | -0.8462532 | 1.6447235 |
| C | -1.1258938 | -0.3831311 | -2.6563569 |
| C | -2.1145673 | 0.5892664 | -2.8280859 |
| H | -2.2655769 | 1.3438111 | -2.0643601 |
| C | -2.8914693 | 0.6038153 | -3.9641913 |
| H | -3.6586209 | 1.3538759 | -4.1118043 |
| C | -2.6955265 | -0.3568098 | -4.9598656 |
| C | -1.7078505 | -1.3258632 | -4.8026153 |
| H | -1.5319073 | -2.0713401 | -5.5658324 |
| C | -0.9298001 | -1.3307377 | -3.6549987 |
| H | -0.1504143 | -2.0743219 | -3.5375431 |
| O | 1.2057886 | -1.0797491 | -1.5668921 |
| H | 2.1033588 | -1.0959341 | -0.8984239 |
| O | 0.6428354 | 5.0699880 | 0.7513280 |
| O | -2.9426582 | -3.8194659 | 2.7975937 |
| O | -3.5006565 | -0.2620135 | -6.0334972 |
| C | 0.0399053 | 5.5332607 | 1.9388437 |
| H | 0.4301148 | 6.5354933 | 2.1042099 |

| | | | |
|---|---|---|---|
| H | 0.3012956 | 4.8939919 | 2.7872452 |
| H | -1.0495241 | 5.5822928 | 1.8407562 |
| C | -2.4899417 | -3.8959495 | 4.1300318 |
| H | -1.4600294 | -4.2634844 | 4.1800748 |
| H | -3.1491342 | -4.6002339 | 4.6334322 |
| H | -2.5507131 | -2.9232229 | 4.6285049 |
| C | -3.3379798 | -1.2013068 | -7.0714153 |
| H | -2.3365768 | -1.1419520 | -7.5095321 |
| H | -4.0770905 | -0.9463653 | -7.8280471 |
| H | -3.5191135 | -2.2215632 | -6.7182317 |
| C | 1.8183143 | 1.0690087 | 4.0218323 |
| C | 2.1685066 | 2.3044060 | 3.4862186 |
| C | 2.7817294 | 2.3527460 | 2.2372276 |
| C | 3.0298181 | 1.1981152 | 1.5005328 |
| C | 2.6559885 | -0.0837112 | 2.0344050 |
| C | 2.0639569 | -0.0996255 | 3.3260981 |
| C | 2.8893669 | -1.2527889 | 1.2825645 |
| C | 3.4036482 | -1.1435756 | -0.0473776 |
| C | 3.8871525 | 0.1166062 | -0.4960687 |
| C | 3.6829137 | 1.2487085 | 0.2257790 |
| H | 1.3591877 | 1.0174905 | 5.0038318 |
| H | 1.9937918 | 3.2147948 | 4.0482842 |
| H | 3.0827357 | 3.3072795 | 1.8167381 |
| H | 1.8092807 | -1.0503568 | 3.7799040 |
| H | 3.8043159 | -2.0420101 | -0.5126118 |
| H | 4.3836276 | 0.1719381 | -1.4602295 |
| H | 4.0153326 | 2.2115453 | -0.1462581 |
| C | 2.6655290 | -2.6121312 | 1.8635265 |
| H | 3.4144227 | -2.8427801 | 2.6326911 |
| H | 2.7436154 | -3.3778365 | 1.0894530 |
| H | 1.6860455 | -2.7275883 | 2.3358406 |

**5f-TS3**

E(PBE0-D3/def2-TZVP) = -1879.608539787 (conv)

Lowest Freq. = -1077.89 cm^-1

69

5f-TS3 (106TSc1/opt)

| | | | |
|---|---|---|---|
| P | -0.2443678 | -0.7764191 | -1.0225724 |
| C | -0.0269074 | 0.8640663 | -0.3816300 |
| C | 0.7901697 | 1.7485502 | -1.0964690 |
| H | 1.3185050 | 1.4049562 | -1.9779356 |
| C | 0.9327189 | 3.0512901 | -0.6783244 |
| H | 1.5557479 | 3.7511892 | -1.2202719 |
| C | 0.2923590 | 3.4894095 | 0.4824390 |
| C | -0.5320076 | 2.6222778 | 1.1937637 |
| H | -1.0295714 | 2.9396556 | 2.0984569 |
| C | -0.6890257 | 1.3193125 | 0.7533624 |
| H | -1.3316804 | 0.6502659 | 1.3106713 |
| C | -1.1136050 | -1.7656883 | 0.1663871 |
| C | -2.2165802 | -2.5458301 | -0.1996554 |
| H | -2.5925583 | -2.5060128 | -1.2152000 |
| C | -2.8336786 | -3.3624522 | 0.7190700 |
| H | -3.6889319 | -3.9686975 | 0.4477148 |
| C | -2.3601807 | -3.4218179 | 2.0348083 |
| C | -1.2682283 | -2.6475178 | 2.4131690 |
| H | -0.8913221 | -2.6688313 | 3.4267458 |
| C | -0.6501194 | -1.8258071 | 1.4829380 |
| H | 0.1959087 | -1.2242825 | 1.7898031 |
| C | -1.2421631 | -0.6775654 | -2.4996897 |
| C | -2.2821853 | 0.2498051 | -2.6034293 |
| H | -2.4634597 | 0.9485404 | -1.7943799 |
| C | -3.0717900 | 0.2913576 | -3.7298479 |
| H | -3.8792830 | 1.0067072 | -3.8254022 |
| C | -2.8355217 | -0.5947211 | -4.7843936 |
| C | -1.7960297 | -1.5167123 | -4.6949858 |
| H | -1.5891021 | -2.2036721 | -5.5039350 |
| C | -1.0070544 | -1.5506760 | -3.5548680 |
| H | -0.1895445 | -2.2585738 | -3.4870271 |
| O | 1.1050185 | -1.3965805 | -1.4526426 |
| H | 2.0046759 | -1.3651180 | -0.7794312 |
| O | 0.5322878 | 4.7626620 | 0.8385067 |
| O | -3.0283808 | -4.2481614 | 2.8611389 |

| | | | |
|---|---|---|---|
| O | -3.6562927 | -0.4777203 | -5.8438211 |
| C | -0.0291124 | 5.2283858 | 2.0455766 |
| H | 0.3353458 | 6.2455037 | 2.1749061 |
| H | 0.2930966 | 4.6128110 | 2.8901941 |
| H | -1.1229485 | 5.2402521 | 1.9971991 |
| C | -2.5857232 | -4.3501672 | 4.1949459 |
| H | -1.5554620 | -4.7165668 | 4.2461599 |
| H | -3.2470213 | -5.0657640 | 4.6793125 |
| H | -2.6527445 | -3.3876750 | 4.7123055 |
| C | -3.4554490 | -1.3416607 | -6.9389944 |
| H | -2.4635593 | -1.2018242 | -7.3803969 |
| H | -4.2154497 | -1.0809503 | -7.6726088 |
| H | -3.5781420 | -2.3898354 | -6.6479635 |
| C | 1.6739714 | 1.0908888 | 4.0293215 |
| C | 2.1854179 | 2.2723228 | 3.4989184 |
| C | 2.8664865 | 2.2408894 | 2.2838092 |
| C | 3.0394881 | 1.0563205 | 1.5709084 |
| C | 2.5137265 | -0.1669551 | 2.1123058 |
| C | 1.8359726 | -0.1028906 | 3.3584998 |
| C | 2.6797285 | -1.3666598 | 1.4111561 |
| C | 3.2604391 | -1.3816810 | 0.1134997 |
| C | 3.8579128 | -0.1755864 | -0.3505691 |
| C | 3.7512466 | 1.0099901 | 0.3144634 |
| H | 1.1542329 | 1.1055449 | 4.9818450 |
| H | 2.0790109 | 3.2057522 | 4.0398941 |
| H | 3.2684581 | 3.1628551 | 1.8810553 |
| H | 1.4627349 | -1.0255355 | 3.7932836 |
| H | 2.3280265 | -2.2914116 | 1.8563514 |
| H | 3.6256920 | -2.3238236 | -0.2895954 |
| H | 4.3993963 | -0.1971200 | -1.2928942 |
| C | 4.3909457 | 2.2501029 | -0.2181139 |
| H | 3.6617339 | 3.0536453 | -0.3681556 |
| H | 4.8795284 | 2.0534394 | -1.1739027 |
| H | 5.1452003 | 2.6402151 | 0.4742203 |

**5f-TS4**

E(PBE0-D3/def2-TZVP) = -1879.613187414 (conv)

Lowest Freq. = -884.80 cm^-1

69

5f-TS4 (107TSc1/opt)

P -0.3300766 1.1515157 0.2313778

C -1.6782670 2.1730545 0.7979270

C -1.5545595 3.5659335 0.7803509

H -0.6167413 4.0151653 0.4760163

C -2.6098307 4.3653947 1.1595599

H -2.5272252 5.4452792 1.1542638

C -3.8138789 3.7899882 1.5706676

C -3.9446273 2.4029616 1.6004350

H -4.8646819 1.9361738 1.9238836

C -2.8777291 1.6077839 1.2133577

H -2.9804666 0.5288731 1.2429444

C -0.4320652 -0.4300245 1.0156311

C -0.8286066 -1.5827974 0.3271595

H -1.0149918 -1.5345740 -0.7386257

C -0.9801049 -2.7762313 0.9904605

H -1.2738026 -3.6780902 0.4685442

C -0.7356383 -2.8455816 2.3687758

C -0.3600775 -1.7055118 3.0678051

H -0.1639163 -1.7433013 4.1304503

C -0.1993145 -0.5049491 2.3917737

H 0.1130803 0.3766734 2.9381707

C -0.4743281 0.9531245 -1.5254196

C -1.4944490 1.5678144 -2.2591663

H -2.2387137 2.1698944 -1.7517161

C -1.5634908 1.4124668 -3.6249866

H -2.3484658 1.8820450 -4.2048319

C -0.6083048 0.6392409 -4.2914004

C 0.4090070 0.0204785 -3.5707966

H 1.1673208 -0.5784324 -4.0547761

C 0.4733618 0.1812585 -2.1976103

H 1.2783564 -0.3047099 -1.6584686

| | | | |
|---|---|---|---|
| O | 0.9653321 | 1.9059066 | 0.6351542 |
| H | 1.9065346 | 1.3636460 | 0.7459247 |
| O | -4.7862210 | 4.6499054 | 1.9241040 |
| O | -0.8870504 | -4.0631211 | 2.9256434 |
| O | -0.7511023 | 0.5490773 | -5.6265413 |
| C | -6.0204461 | 4.1199973 | 2.3503181 |
| H | -6.6519888 | 4.9749337 | 2.5826836 |
| H | -5.9016243 | 3.5038544 | 3.2473025 |
| H | -6.4928646 | 3.5251816 | 1.5620052 |
| C | -0.5980003 | -4.2029137 | 4.2962088 |
| H | 0.4404556 | -3.9319845 | 4.5132631 |
| H | -0.7529643 | -5.2535555 | 4.5335333 |
| H | -1.2663943 | -3.5910307 | 4.9111154 |
| C | 0.1995611 | -0.2083197 | -6.3410175 |
| H | 1.2084732 | 0.1962942 | -6.2131897 |
| H | -0.0870023 | -0.1419804 | -7.3886334 |
| H | 0.1903176 | -1.2570206 | -6.0276234 |
| C | 3.9426977 | -0.0582428 | -2.6891174 |
| C | 3.6415280 | -1.4141409 | -2.7155583 |
| C | 3.2676892 | -2.0647261 | -1.5491496 |
| C | 3.1931673 | -1.3857544 | -0.3172542 |
| C | 3.4863722 | 0.0141819 | -0.2989599 |
| C | 3.8547641 | 0.6428630 | -1.4952592 |
| C | 3.3559798 | 0.7405177 | 0.9349382 |
| C | 3.1604245 | 0.0179454 | 2.1352394 |
| C | 2.8917027 | -1.3346426 | 2.0983958 |
| C | 2.8625603 | -2.0518173 | 0.9031761 |
| H | 4.2394083 | 0.4549559 | -3.5969676 |
| H | 3.7080879 | -1.9704238 | -3.6445918 |
| H | 3.0435969 | -3.1239252 | -1.5814428 |
| H | 4.0806571 | 1.7042392 | -1.4733747 |
| H | 3.8033189 | 1.7310584 | 0.9815870 |
| H | 3.1909107 | 0.5391155 | 3.0858061 |
| H | 2.6967876 | -1.8639246 | 3.0256524 |
| C | 2.5500405 | -3.5098195 | 0.8995637 |
| H | 1.6601838 | -3.7353596 | 0.2983278 |

H 2.3570290 -3.8659257 1.9131018
H 3.3713058 -4.1059607 0.4838795

**5f-TS5**

E(PBE0-D3/def2-TZVP) = -1879.611788731 (conv)

Lowest Freq. = -779.44 cm^-1

69

5f-TS5 (101TSc1/opt)

P -0.3383649 1.1695856 0.3160732
C -1.6885813 2.1765493 0.9006683
C -1.5737960 3.5705944 0.8995117
H -0.6399858 4.0297322 0.5977545
C -2.6329756 4.3584989 1.2915494
H -2.5572386 5.4388728 1.2988929
C -3.8319946 3.7705848 1.6997711
C -3.9538331 2.3824121 1.7131173
H -4.8700014 1.9058782 2.0333705
C -2.8834307 1.5988198 1.3127479
H -2.9799237 0.5190546 1.3285712
C -0.4307168 -0.4259085 1.0727891
C -0.8063045 -1.5725965 0.3607990
H -0.9935384 -1.5072710 -0.7037683
C -0.9302338 -2.7814812 0.9991395
H -1.2038920 -3.6785502 0.4580921
C -0.6788109 -2.8761776 2.3738328
C -0.3359113 -1.7401186 3.0982575
H -0.1401241 -1.7948809 4.1601541
C -0.2041548 -0.5241629 2.4479691
H 0.0907257 0.3519330 3.0129830
C -0.4782752 0.9998865 -1.4424160
C -1.4911262 1.6351849 -2.1695536
H -2.2323312 2.2348040 -1.6547465
C -1.5570866 1.5022492 -3.5375355
H -2.3367784 1.9869729 -4.1120219
C -0.6056492 0.7322393 -4.2136993
C 0.4042475 0.0937758 -3.5001312

| | | | |
|---|---|---|---|
| H | 1.1590854 | -0.5039483 | -3.9910795 |
| C | 0.4653395 | 0.2305182 | -2.1242077 |
| H | 1.2649181 | -0.2705204 | -1.5907214 |
| O | 0.9527508 | 1.9295261 | 0.7356465 |
| H | 1.8943345 | 1.4126516 | 0.8166086 |
| O | -4.8084476 | 4.6198959 | 2.0669596 |
| O | -0.8042173 | -4.1067793 | 2.9068800 |
| O | -0.7455441 | 0.6646810 | -5.5505814 |
| C | -6.0381582 | 4.0770925 | 2.4901023 |
| H | -6.6742020 | 4.9251589 | 2.7350008 |
| H | -5.9128545 | 3.4505538 | 3.3789562 |
| H | -6.5092578 | 3.4893148 | 1.6957567 |
| C | -0.5162642 | -4.2669064 | 4.2759388 |
| H | 0.5149985 | -3.9771118 | 4.5018375 |
| H | -0.6484708 | -5.3252479 | 4.4917384 |
| H | -1.2007830 | -3.6828145 | 4.8999785 |
| C | 0.2011712 | -0.0884940 | -6.2745750 |
| H | 1.2127266 | 0.3067068 | -6.1383764 |
| H | -0.0824676 | -0.0039352 | -7.3216765 |
| H | 0.1836585 | -1.1418146 | -5.9774192 |
| C | 3.8502150 | -0.2792640 | -2.5854120 |
| C | 3.4802847 | -1.6170655 | -2.4984084 |
| C | 3.1089386 | -2.1863572 | -1.2878054 |
| C | 3.1159257 | -1.3960658 | -0.1102945 |
| C | 3.4612056 | -0.0108761 | -0.2067513 |
| C | 3.8250634 | 0.5167135 | -1.4514132 |
| C | 3.3909114 | 0.8036850 | 0.9769421 |
| C | 3.2211330 | 0.1872841 | 2.2379330 |
| C | 2.9141756 | -1.1546576 | 2.3184014 |
| C | 2.8216757 | -1.9364243 | 1.1677147 |
| H | 4.1458516 | 0.1426715 | -3.5394386 |
| H | 3.4912486 | -2.2386791 | -3.3889631 |
| H | 4.0965465 | 1.5655396 | -1.5143038 |
| H | 3.8377110 | 1.7940933 | 0.9291106 |
| H | 3.2959008 | 0.7859739 | 3.1393004 |
| H | 2.7440495 | -1.6101210 | 3.2875945 |

H 2.5656207 -2.9840404 1.2557080
C 2.7270137 -3.6308826 -1.2168396
H 2.7364877 -4.0841447 -2.2093923
H 1.7265242 -3.7635024 -0.7915151
H 3.4152864 -4.1988883 -0.5811061

**5f-TS6**

E(PBE0-D3/def2-TZVP) = -1879.607435551 (conv)

Lowest Freq. = -1099.27 cm^-1

69

5f-TS6 (102TSc1/opt)

P -0.2179906 -0.8365319 -1.1135467
C -0.0089841 0.8053261 -0.4719914
C 0.8297719 1.6825726 -1.1690078
H 1.3757412 1.3333344 -2.0372419
C 0.9786792 2.9825139 -0.7466741
H 1.6293353 3.6712171 -1.2709179
C 0.3189652 3.4270846 0.3999879
C -0.5231403 2.5649402 1.0980815
H -1.0326048 2.8862760 1.9948110
C -0.6853104 1.2652794 0.6534383
H -1.3421755 0.6010153 1.1998758
C -1.0837607 -1.8301242 0.0724724
C -2.1631455 -2.6388593 -0.3027948
H -2.5259966 -2.6147420 -1.3236435
C -2.7732349 -3.4635015 0.6130448
H -3.6101788 -4.0916488 0.3345727
C -2.3159779 -3.5030065 1.9357417
C -1.2485358 -2.7001805 2.3235246
H -0.8855409 -2.7057509 3.3424031
C -0.6374050 -1.8695542 1.3961942
H 0.1887423 -1.2448572 1.7103710
C -1.2104877 -0.7439685 -2.5945443
C -2.2547670 0.1780626 -2.7027680
H -2.4426493 0.8765069 -1.8949721
C -3.0406047 0.2148525 -3.8319663

| | | | |
|---|---|---|---|
| H | -3.8513469 | 0.9260622 | -3.9308968 |
| C | -2.7963365 | -0.6709496 | -4.8849061 |
| C | -1.7526089 | -1.5877032 | -4.7910909 |
| H | -1.5394856 | -2.2743190 | -5.5987272 |
| C | -0.9672696 | -1.6166379 | -3.6483261 |
| H | -0.1463188 | -2.3202347 | -3.5772132 |
| O | 1.1385204 | -1.4461358 | -1.5390335 |
| H | 2.0325196 | -1.4111284 | -0.8617902 |
| O | 0.5584150 | 4.6995165 | 0.7582640 |
| O | -2.9757413 | -4.3398166 | 2.7584753 |
| O | -3.6140594 | -0.5589205 | -5.9472367 |
| C | 0.0040443 | 5.1614044 | 1.9699938 |
| H | 0.3796927 | 6.1734882 | 2.1066835 |
| H | 0.3218646 | 4.5352805 | 2.8085776 |
| H | -1.0896801 | 5.1858474 | 1.9237909 |
| C | -2.5481712 | -4.4236326 | 4.0983787 |
| H | -1.5101019 | -4.7646868 | 4.1649909 |
| H | -3.1982604 | -5.1522589 | 4.5784950 |
| H | -2.6450149 | -3.4601408 | 4.6091487 |
| C | -3.4053005 | -1.4229121 | -7.0408752 |
| H | -2.4126322 | -1.2786450 | -7.4791015 |
| H | -4.1641088 | -1.1666541 | -7.7772884 |
| H | -3.5238238 | -2.4713912 | -6.7492161 |
| C | 1.6986008 | 1.1120473 | 3.9321940 |
| C | 2.2641278 | 2.2704040 | 3.4057362 |
| C | 2.9647399 | 2.2394337 | 2.1991122 |
| C | 3.0808025 | 1.0325442 | 1.4950668 |
| C | 2.5185191 | -0.1725583 | 2.0373838 |
| C | 1.8274110 | -0.0902503 | 3.2728973 |
| C | 2.6744347 | -1.3836873 | 1.3478704 |
| C | 3.2755098 | -1.4335322 | 0.0565231 |
| C | 3.8943361 | -0.2450404 | -0.4191293 |
| C | 3.7832765 | 0.9379177 | 0.2460237 |
| H | 1.1675738 | 1.1551372 | 4.8775516 |
| H | 2.1887514 | 3.2051003 | 3.9519964 |
| H | 1.4221358 | -0.9992192 | 3.7073237 |

H 2.3028632 -2.2961287 1.8019512
H 3.6273182 -2.3900547 -0.3240517
H 4.4391778 -0.2788157 -1.3579881
H 4.2375520 1.8280264 -0.1706349
C 3.6217326 3.4873577 1.6925442
H 3.2965582 3.7452537 0.6812461
H 4.7109599 3.3797453 1.6609210
H 3.3900784 4.3346312 2.3399155

**5f-TS7**

E(PBE0-D3/def2-TZVP) = -1879.607591181 (conv)

Lowest Freq. = -1114.00 cm^-1

69

5f-TS7 (103TSc1/opt)

P -0.1119367 -0.5742905 -1.2271775
C 0.0819993 1.0725734 -0.5947377
C 0.8935889 1.9695081 -1.3006360
H 1.4319438 1.6355717 -2.1794919
C 1.0266234 3.2698655 -0.8719104
H 1.6643668 3.9709968 -1.3957034
C 0.3741503 3.6971948 0.2844003
C -0.4532211 2.8201134 0.9832583
H -0.9609029 3.1289427 1.8853881
C -0.5916675 1.5186864 0.5386162
H -1.2287821 0.8397337 1.0905584
C -1.0154109 -1.5407390 -0.0470852
C -2.1807485 -2.2313834 -0.4002572
H -2.5636255 -2.1603468 -1.4113862
C -2.8508212 -2.9967258 0.5251507
H -3.7538041 -3.5338328 0.2627273
C -2.3720049 -3.0890413 1.8365888
C -1.2139112 -2.4094962 2.2002366
H -0.8299869 -2.4640727 3.2094184
C -0.5380292 -1.6435155 1.2627305
H 0.3640664 -1.1175297 1.5556459
C -1.0668997 -0.4890202 -2.7325682

| | | | |
|---|---|---|---|
| C | -2.0727143 | 0.4672159 | -2.8947158 |
| H | -2.2494119 | 1.1997810 | -2.1152067 |
| C | -2.8348255 | 0.4932145 | -4.0406839 |
| H | -3.6155635 | 1.2305530 | -4.1809995 |
| C | -2.6047990 | -0.4382958 | -5.0564230 |
| C | -1.5989129 | -1.3900342 | -4.9090049 |
| H | -1.3971246 | -2.1128904 | -5.6874102 |
| C | -0.8376967 | -1.4077283 | -3.7501909 |
| H | -0.0461180 | -2.1391071 | -3.6378806 |
| O | 1.2451038 | -1.2109101 | -1.6081759 |
| H | 2.1621580 | -1.1450974 | -0.9669176 |
| O | 0.6054684 | 4.9692164 | 0.6539823 |
| O | -3.0983500 | -3.8526205 | 2.6750152 |
| O | -3.3972281 | -0.3342109 | -6.1384637 |
| C | 0.0388984 | 5.4188264 | 1.8645572 |
| H | 0.3979089 | 6.4362714 | 2.0060550 |
| H | 0.3616027 | 4.7963904 | 2.7040879 |
| H | -1.0548925 | 5.4260032 | 1.8144220 |
| C | -2.6500698 | -3.9848199 | 4.0042552 |
| H | -1.6541864 | -4.4373817 | 4.0462261 |
| H | -3.3641632 | -4.6387072 | 4.5007869 |
| H | -2.6281298 | -3.0173986 | 4.5163940 |
| C | -3.2004177 | -1.2438262 | -7.1968234 |
| H | -2.1947457 | -1.1523371 | -7.6192955 |
| H | -3.9346377 | -0.9858101 | -7.9571546 |
| H | -3.3643003 | -2.2761041 | -6.8713317 |
| C | 1.9654376 | 1.1283898 | 3.9244683 |
| C | 2.2736922 | 2.3656534 | 3.3610408 |
| C | 2.8563206 | 2.4150234 | 2.1021847 |
| C | 3.1028568 | 1.2504797 | 1.3802688 |
| C | 2.7628693 | -0.0268203 | 1.9430121 |
| C | 2.2133444 | -0.0593240 | 3.2620011 |
| C | 2.9941543 | -1.1859130 | 1.1896580 |
| C | 3.4742031 | -1.1173392 | -0.1510142 |
| C | 3.9282634 | 0.1457095 | -0.6264471 |
| C | 3.7218987 | 1.2836145 | 0.0870994 |

H 1.5409750 1.0887278 4.9236820
H 2.0900817 3.2764561 3.9197162
H 3.1278423 3.3688910 1.6610045
H 2.7734887 -2.1563205 1.6169037
H 3.8792023 -2.0183576 -0.6059566
H 4.4039552 0.1998040 -1.6010845
H 4.0294743 2.2453622 -0.3091209
C 1.9761715 -1.3706813 3.9384437
H 2.9203773 -1.8996911 4.1133156
H 1.3605232 -2.0485748 3.3381417
H 1.4870288 -1.2284749 4.9038129

**5f-TS8**

E(PBE0-D3/def2-TZVP) = -1879.612415674 (conv)

Lowest Freq. = -823.65 cm^-1

69

5f-TS8 (104TSc1/opt)

P -0.4096945 0.8711940 0.3487133
C -1.7600014 1.8877491 0.9169473
C -1.6383312 3.2811876 0.9134693
H -0.7000509 3.7349992 0.6175180
C -2.6962279 4.0753626 1.2963974
H -2.6150984 5.1553621 1.3016449
C -3.9005175 3.4944140 1.6988809
C -4.0290207 2.1068859 1.7153182
H -4.9492551 1.6356229 2.0317012
C -2.9602359 1.3170488 1.3231994
H -3.0621398 0.2377959 1.3415532
C -0.5084697 -0.7177461 1.1187641
C -0.9272536 -1.8623038 0.4285468
H -1.1381438 -1.8036485 -0.6320250
C -1.0667230 -3.0610665 1.0833427
H -1.3743921 -3.9566827 0.5586272
C -0.7894009 -3.1464203 2.4537114
C -0.3969727 -2.0129185 3.1560943
H -0.1773844 -2.0610858 4.2136609

| | | | |
|---|---|---|---|
| C | -0.2477374 | -0.8079078 | 2.4883401 |
| H | 0.0826646 | 0.0665256 | 3.0358278 |
| C | -0.5367188 | 0.6905244 | -1.4096273 |
| C | -1.5027672 | 1.3780399 | -2.1534458 |
| H | -2.2208184 | 2.0146467 | -1.6503727 |
| C | -1.5522639 | 1.2502493 | -3.5223846 |
| H | -2.2955302 | 1.7757198 | -4.1091732 |
| C | -0.6303619 | 0.4324756 | -4.1834825 |
| C | 0.3302776 | -0.2606348 | -3.4536903 |
| H | 1.0609842 | -0.8960964 | -3.9332340 |
| C | 0.3754960 | -0.1286171 | -2.0764628 |
| H | 1.1382516 | -0.6723074 | -1.5307396 |
| O | 0.8820000 | 1.6270610 | 0.7745736 |
| H | 1.8200724 | 1.0916456 | 0.8551913 |
| O | -4.8750389 | 4.3494542 | 2.0581579 |
| O | -0.9383765 | -4.3666423 | 3.0045082 |
| O | -0.7480592 | 0.3760536 | -5.5229684 |
| C | -6.1094612 | 3.8135471 | 2.4762394 |
| H | -6.7428089 | 4.6652086 | 2.7156070 |
| H | -5.9917602 | 3.1887498 | 3.3673674 |
| H | -6.5791885 | 3.2258663 | 1.6809980 |
| C | -0.6294936 | -4.5178893 | 4.3699002 |
| H | 0.4140118 | -4.2563211 | 4.5730579 |
| H | -0.7889188 | -5.5689786 | 4.6022120 |
| H | -1.2843563 | -3.9055786 | 4.9987166 |
| C | 0.1742456 | -0.4207388 | -6.2316943 |
| H | 1.2015254 | -0.0790064 | -6.0706758 |
| H | -0.0817303 | -0.3155955 | -7.2840461 |
| H | 0.0961668 | -1.4734665 | -5.9422883 |
| C | 3.7590842 | -0.4619147 | -2.6030641 |
| C | 3.4837957 | -1.8243238 | -2.5744911 |
| C | 3.1451466 | -2.4349959 | -1.3812359 |
| C | 3.0965111 | -1.7017054 | -0.1814098 |
| C | 3.3763738 | -0.3002214 | -0.2160109 |
| C | 3.6918620 | 0.3102737 | -1.4474661 |
| C | 3.2875397 | 0.4461869 | 1.0120013 |

| | | | |
|---|---|---|---|
| C | 3.1214861 | -0.2442063 | 2.2383418 |
| C | 2.8518772 | -1.5978190 | 2.2491920 |
| C | 2.8021533 | -2.3193444 | 1.0624204 |
| H | 4.0178200 | 0.0183379 | -3.5413911 |
| H | 3.5407818 | -2.4086427 | -3.4868564 |
| H | 2.9339877 | -3.4988792 | -1.3490344 |
| H | 3.7311694 | 1.4373984 | 1.0390014 |
| H | 3.1777241 | 0.3081342 | 3.1700697 |
| H | 2.6809542 | -2.1051003 | 3.1922159 |
| H | 2.5860552 | -3.3817989 | 1.0751868 |
| C | 3.9488179 | 1.7824834 | -1.5078562 |
| H | 4.1537836 | 2.0993718 | -2.5314295 |
| H | 4.8043855 | 2.0718077 | -0.8878449 |
| H | 3.0876985 | 2.3532210 | -1.1428163 |

**5f-H2**

E(PBE0-D3/def2-TZVP) = -425.4149146717 (conv)

Lowest Freq. = 61.46 cm^-1

22

5f-H2 (118c1/opt)

| | | | |
|---|---|---|---|
| C | 2.7290840 | 1.2223825 | -0.1021585 |
| C | 2.7402769 | -0.1728417 | -0.1085290 |
| C | 1.5609897 | -0.8821545 | -0.0652573 |
| C | 0.3072403 | -0.2246691 | -0.0133113 |
| C | 0.3112035 | 1.2056406 | -0.0078889 |
| C | 1.5158013 | 1.8934231 | -0.0519966 |
| C | -0.9493398 | 1.9091875 | 0.0439701 |
| C | -2.1197656 | 1.2684754 | 0.0875200 |
| C | -2.2115427 | -0.2156804 | 0.0864400 |
| C | -0.9050334 | -0.9285978 | 0.0317301 |
| H | 3.6590071 | 1.7768749 | -0.1362069 |
| H | 3.6843898 | -0.7044017 | -0.1478533 |
| H | 1.5917560 | -1.9646807 | -0.0708866 |
| H | 1.4964158 | 2.9785903 | -0.0467384 |
| H | -0.9197951 | 2.9944400 | 0.0464362 |
| H | -3.0482483 | 1.8286240 | 0.1256991 |

H -2.8465400 -0.5472786 -0.7541044
C -0.9468249 -2.4190453 0.0281678
H -0.4146727 -2.8450897 0.8861413
H -0.4817925 -2.8392014 -0.8706928
H -1.9754857 -2.7813580 0.0662878
H -2.7771236 -0.5526394 0.9732314

**5f-H3**

E(PBE0-D3/def2-TZVP) = -425.4145047024 (conv)

Lowest Freq. = 92.60 cm^-1

22

5f-H3 (119c1/opt)

C 2.7345206 1.1355311 -0.0958470
C 2.7311886 -0.2606303 -0.1155419
C 1.5261295 -0.9497443 -0.0764071
C 0.3084695 -0.2835963 -0.0171090
C 0.3040229 1.1483748 0.0042273
C 1.5479634 1.8256554 -0.0373573
C -0.8969628 1.8477545 0.0645866
C -2.2115310 1.1704893 0.1107762
C -2.1177480 -0.3134225 0.0827449
C -0.9643198 -0.9944649 0.0241414
H 3.6740873 1.6753255 -0.1265714
H 3.6654071 -0.8071844 -0.1613370
H 1.5404504 -2.0326415 -0.0925898
H 1.5450118 2.9104049 -0.0215662
H -0.8731088 2.9322770 0.0796828
H -2.8481628 1.5167599 -0.7213850
H -3.0533782 -0.8643967 0.1125854
C -0.9620013 -2.4909107 0.0000252
H -0.4124924 -2.9025701 0.8516973
H -0.4840044 -2.8742069 -0.9062498
H -1.9807159 -2.8774645 0.0353146
H -2.7728254 1.4886606 1.0061797

**5f-H4**

E(PBE0-D3/def2-TZVP) = -425.4227859471 (conv)

Lowest Freq. = 77.21 cm^-1

22

5f-H4 (120c1/opt)

| | | | |
|---|---|---|---|
| C | 2.6786404 | 1.0793747 | -0.0756857 |
| C | 2.6733225 | -0.3096350 | -0.0993038 |
| C | 1.4752435 | -0.9942775 | -0.0683907 |
| C | 0.2450574 | -0.3177234 | -0.0127333 |
| C | 0.2604393 | 1.0935698 | 0.0116203 |
| C | 1.4744054 | 1.7634508 | -0.0206907 |
| C | -1.0156556 | 1.8839855 | 0.0734269 |
| C | -2.2478277 | 1.0570889 | 0.0921123 |
| C | -2.2027612 | -0.3044552 | 0.0665187 |
| C | -1.0050115 | -1.0324523 | 0.0171808 |
| H | 3.6143667 | 1.6252752 | -0.1001402 |
| H | 3.6074467 | -0.8577995 | -0.1422608 |
| H | 1.4837707 | -2.0770186 | -0.0878598 |
| H | 1.4747433 | 2.8492389 | -0.0021555 |
| H | -1.0509914 | 2.5850695 | -0.7742237 |
| H | -3.2028377 | 1.5685808 | 0.1299868 |
| H | -3.1356892 | -0.8602239 | 0.0844934 |
| C | -1.0293125 | -2.5222964 | -0.0075965 |
| H | -0.4928653 | -2.9526309 | 0.8454560 |
| H | -0.5548074 | -2.9222980 | -0.9107188 |
| H | -2.0545501 | -2.8927313 | 0.0221191 |
| H | -0.9951263 | 2.5379081 | 0.9588453 |

**5f-H5**

E(PBE0-D3/def2-TZVP) = -425.4218075840 (conv)

Lowest Freq. = 78.80 cm^-1

22

5f-H5 (114c1/opt)

| | | | |
|---|---|---|---|
| C | 2.6027238 | 1.0561522 | -0.0535326 |
| C | 2.5585639 | -0.3050240 | -0.0808644 |
| C | 1.3465965 | -1.0047185 | -0.0561766 |

| | | | |
|---|---|---|---|
| C | 0.0952812 | -0.3182056 | -0.0073926 |
| C | 0.0833044 | 1.0931057 | 0.0205408 |
| C | 1.3668197 | 1.8769156 | 0.0077604 |
| C | -1.1257736 | 1.7703207 | 0.0623182 |
| C | -2.3246324 | 1.0791665 | 0.0787838 |
| C | -2.3180668 | -0.3107110 | 0.0539774 |
| C | -1.1330104 | -1.0227022 | 0.0111814 |
| H | 3.5548668 | 1.5742159 | -0.0732692 |
| H | 3.4857369 | -0.8667985 | -0.1233163 |
| H | 1.3590741 | -2.0858589 | -0.0782783 |
| H | 1.3510481 | 2.5841643 | -0.8355093 |
| H | -1.1254799 | 2.8557742 | 0.0824462 |
| H | -3.2646615 | 1.6173855 | 0.1111405 |
| H | -3.2580779 | -0.8522897 | 0.0677751 |
| C | -1.1588135 | -2.5195517 | -0.0155836 |
| H | -0.6308683 | -2.9469617 | 0.8418502 |
| H | -0.6808775 | -2.9148371 | -0.9166964 |
| H | -2.1853314 | -2.8863638 | 0.0068439 |
| H | 1.4015777 | 2.5268221 | 0.8960017 |

**5f-H6**

E(PBE0-D3/def2-TZVP) = -425.4131591360 (conv)

Lowest Freq. = 102.42 cm^-1

22

5f-H6 (115c1/opt)

| | | | |
|---|---|---|---|
| C | 2.5662686 | 1.1718256 | -0.0288847 |
| C | 2.4735922 | -0.3134049 | -0.0447038 |
| C | 1.3049764 | -0.9647990 | -0.0337035 |
| C | 0.0291535 | -0.2843534 | -0.0044661 |
| C | 0.0372696 | 1.1464173 | 0.0123516 |
| C | 1.2444028 | 1.8413074 | -0.0000059 |
| C | -1.2024027 | 1.8303507 | 0.0416634 |
| C | -2.3833264 | 1.1330415 | 0.0541558 |
| C | -2.3770678 | -0.2642083 | 0.0375249 |
| C | -1.1847378 | -0.9801783 | 0.0081290 |
| H | 3.1512166 | 1.5163085 | -0.8990520 |

| | | | |
|---|---|---|---|
| H | 3.4029575 | -0.8731464 | -0.0663387 |
| H | 1.3077789 | -2.0483712 | -0.0467334 |
| H | 1.2202751 | 2.9259727 | 0.0124228 |
| H | -1.1996581 | 2.9148980 | 0.0544591 |
| H | -3.3278931 | 1.6645840 | 0.0770856 |
| H | -3.3171100 | -0.8046934 | 0.0476255 |
| C | -1.2221769 | -2.4793907 | -0.0093314 |
| H | -0.7099381 | -2.9069304 | 0.8570151 |
| H | -0.7400308 | -2.8851335 | -0.9030227 |
| H | -2.2522540 | -2.8365193 | 0.0038835 |
| H | 3.1787046 | 1.4964232 | 0.8299260 |

**5f-H7**

E(PBE0-D3/def2-TZVP) = -425.4146258173 (conv)

Lowest Freq. = 102.48 cm^-1

22

5f-H7 (116c1/opt)

| | | | |
|---|---|---|---|
| C | 2.4680933 | 1.2815602 | -0.0424504 |
| C | 2.5734718 | -0.2027839 | -0.0392616 |
| C | 1.2618337 | -0.8967120 | -0.0185048 |
| C | 0.0426204 | -0.2262221 | -0.0002458 |
| C | 0.0296566 | 1.2058728 | -0.0027076 |
| C | 1.2899096 | 1.9117985 | -0.0250873 |
| C | -1.1768725 | 1.8901412 | 0.0155686 |
| C | -2.3792913 | 1.2025994 | 0.0361281 |
| C | -2.3780420 | -0.1941652 | 0.0382481 |
| C | -1.2063170 | -0.9206006 | 0.0205759 |
| H | 3.3908973 | 1.8519665 | -0.0592547 |
| H | 3.1629327 | -0.5347582 | -0.9108647 |
| H | 1.2757482 | -1.9796359 | -0.0172067 |
| H | 1.2541026 | 2.9969700 | -0.0279118 |
| H | -1.1668647 | 2.9751623 | 0.0134364 |
| H | -3.3183076 | 1.7426359 | 0.0504518 |
| H | -3.3237019 | -0.7262178 | 0.0544128 |
| C | -1.2412394 | -2.4148052 | 0.0236475 |
| H | -0.7242188 | -2.8270168 | 0.8958002 |

H -0.7521192 -2.8299683 -0.8630723
H -2.2695750 -2.7767807 0.0405686
H 3.1872832 -0.5290401 0.8177295

**5f-H8**

E(PBE0-D3/def2-TZVP) = -425.4225552921 (conv)

Lowest Freq. = 107.07 cm^-1

22

5f-H8 (117c1/opt)

C 2.5504677 1.2682227 -0.0727577
C 2.6038102 -0.0946066 -0.0672411
C 1.3767534 -0.9320487 -0.0282464
C 0.0834007 -0.1687476 -0.0005860
C 0.0920741 1.2418089 -0.0078682
C 1.3318733 1.9500790 -0.0432359
C -1.1283856 1.9340773 0.0192867
C -2.3229887 1.2493863 0.0524387
C -2.3229456 -0.1400941 0.0592696
C -1.1303962 -0.8575675 0.0330490
H 3.4734865 1.8375219 -0.1008406
H 3.5602035 -0.6045619 -0.0908496
H 1.3774591 -1.6232063 -0.8863989
H 1.3101110 3.0333593 -0.0479643
H -1.1152422 3.0187095 0.0135852
H -3.2617864 1.7907741 0.0731893
H -3.2632949 -0.6801577 0.0854625
C -1.1568563 -2.3549666 0.0408155
H -0.6228465 -2.7626244 0.9046232
H -0.6764890 -2.7705036 -0.8503653
H -2.1811183 -2.7268324 0.0736932
H 1.4227103 -1.6080216 0.8409412

**5b**

E(PBE0-D3/def2-TZVP) = -613.3068493583 (conv)

Lowest Freq. = 31.23 cm^-1

24

5b (092c1/opt)

C 2.9099920 1.9886057 -0.3314012

C 2.9898518 0.5924064 -0.1935903

C 1.8623563 -0.1691801 -0.0425398

C 0.5802515 0.4281776 -0.0232913

C 0.5034274 1.8445575 -0.1702892

C 1.6881777 2.5981411 -0.3200110

C -0.7529382 2.4854102 -0.1685536

C -1.9067490 1.7662903 -0.0389344

C -1.8457280 0.3730132 0.0951389

C -0.6433732 -0.2980779 0.1122828

H 3.8139908 2.5748634 -0.4476813

H 3.9598258 0.1086176 -0.2054215

H 1.9398151 -1.2390426 0.0747377

H 1.6048938 3.6741792 -0.4289027

H -0.7835338 3.5647877 -0.2742264

H -2.8693483 2.2631498 -0.0380213

H -2.7611152 -0.1933724 0.1965697

C -0.6607344 -1.7721939 0.3003264

O 0.2711102 -2.4595430 0.6366115

O -1.8753195 -2.2935335 0.0618382

C -1.9785011 -3.6963640 0.2656342

H -1.2956243 -4.2323541 -0.3948252

H -3.0101406 -3.9535658 0.0361039

H -1.7405869 -3.9549724 1.2984461

**5b-H2**

E(PBE0-D3/def2-TZVP) = -613.8644699240 (conv)

Lowest Freq. = 41.15 cm^-1

25

5b-H2 (110c1/opt)

C 3.0150532 1.9782061 -0.2861187

C 3.1051394 0.5913460 -0.1749699
C 1.9721962 -0.1818435 -0.0638081
C 0.6826814 0.4030785 -0.0598751
C 0.6052043 1.8266258 -0.1754275
C 1.7695240 2.5789930 -0.2848260
C -0.6788311 2.4858064 -0.1790541
C -1.8206242 1.8046703 -0.0799721
C -1.8506736 0.3292525 0.0414542
C -0.5178175 -0.3447822 0.0502102
H 3.9114871 2.5807175 -0.3728286
H 4.0769382 0.1113786 -0.1755349
H 2.0502448 -1.2544497 0.0226257
H 1.6843023 3.6571107 -0.3705637
H -0.6871543 3.5674225 -0.2668562
H -2.7735409 2.3229251 -0.0859132
H -2.4694403 -0.0945023 -0.7641970
C -0.5308830 -1.8026002 0.1776365
O 0.4189394 -2.5539516 0.2159151
O -1.7974725 -2.2704114 0.2559254
C -1.9090718 -3.6795540 0.3834921
H -1.4615640 -4.1829157 -0.4749959
H -2.9754171 -3.8898196 0.4338159
H -1.4088188 -4.0274262 1.2886380
H -2.4104012 0.0447233 0.9452280

**5b-H3**

E(PBE0-D3/def2-TZVP) = -613.8561060737 (conv)

Lowest Freq. = 41.76 cm^-1

25

5b-H3 (109c1/opt)

C 3.0205693 1.8734248 -0.3890334
C 3.0791110 0.4841634 -0.2670065
C 1.9170733 -0.2526181 -0.0854873
C 0.6731297 0.3651815 -0.0232742
C 0.6018725 1.7904397 -0.1573340
C 1.8074409 2.5131444 -0.3352456

C -0.6201041 2.4558923 -0.1210643
C -1.9098151 1.7507881 0.0409902
C -1.7666295 0.2799864 0.1600662
C -0.5854554 -0.3608647 0.1405032
H 3.9304520 2.4463167 -0.5267917
H 4.0337528 -0.0261220 -0.3093310
H 1.9777442 -1.3249498 0.0269955
H 1.7495916 3.5919364 -0.4326362
H -0.6314203 3.5357024 -0.2213971
H -2.5892801 1.9792644 -0.7981296
H -2.6736252 -0.2993938 0.2799008
C -0.5929918 -1.8363363 0.3483335
O 0.3039757 -2.4836720 0.8249640
O -1.7497221 -2.3950762 -0.0402532
C -1.8521185 -3.7929308 0.2004094
H -1.0709441 -4.3332147 -0.3357979
H -2.8348010 -4.0845559 -0.1633848
H -1.7585429 -4.0093841 1.2656042
H -2.4592630 2.1328779 0.9184000

**5b-H4**

E(PBE0-D3/def2-TZVP) = -613.8697969114 (conv)

Lowest Freq. = 23.83 cm^-1

25

5b-H4 (108c1/opt)

C 2.9770723 1.8579030 -0.2844908
C 3.0515110 0.4742642 -0.1808598
C 1.9034304 -0.2789992 -0.0583398
C 0.6319693 0.3254437 -0.0350862
C 0.5676239 1.7332699 -0.1441991
C 1.7376999 2.4704605 -0.2655792
C -0.7371476 2.4698373 -0.1326267
C -1.9303669 1.6037415 -0.0474077
C -1.8384052 0.2561062 0.0495112
C -0.6003244 -0.4276102 0.0759547
H 3.8778587 2.4527875 -0.3805948

| | | | |
|---|---|---|---|
| H | 4.0149623 | -0.0218547 | -0.1961267 |
| H | 1.9658660 | -1.3526641 | 0.0265797 |
| H | 1.6682216 | 3.5507616 | -0.3482022 |
| H | -0.8059544 | 3.1075640 | -1.0262515 |
| H | -2.9055682 | 2.0778563 | -0.0555887 |
| H | -2.7422972 | -0.3328722 | 0.1181019 |
| C | -0.6171429 | -1.8910566 | 0.2246989 |
| O | 0.3381456 | -2.6151461 | 0.3907580 |
| O | -1.8692194 | -2.3908592 | 0.1647180 |
| C | -1.9598159 | -3.7984952 | 0.3216083 |
| H | -1.3982167 | -4.3122702 | -0.4601195 |
| H | -3.0187252 | -4.0372690 | 0.2480188 |
| H | -1.5660368 | -4.1064111 | 1.2915308 |
| H | -0.7451404 | 3.1855122 | 0.7039922 |

**5b-H5**

E(PBE0-D3/def2-TZVP) = -613.8655389537 (conv)

Lowest Freq. = 28.24 cm^-1

25

5b-H5 (129c1/opt)

| | | | |
|---|---|---|---|
| C | 2.9159762 | 1.8287565 | -0.3084749 |
| C | 2.9449461 | 0.4749016 | -0.1843614 |
| C | 1.7817670 | -0.2901239 | -0.0268931 |
| C | 0.4913539 | 0.3197939 | -0.0051807 |
| C | 0.4034523 | 1.7270697 | -0.1426170 |
| C | 1.6366810 | 2.5770475 | -0.2803817 |
| C | -0.8312798 | 2.3513691 | -0.1482447 |
| C | -2.0060703 | 1.6265906 | -0.0273821 |
| C | -1.9427229 | 0.2507853 | 0.1009736 |
| C | -0.7215246 | -0.4133798 | 0.1211087 |
| H | 3.8338072 | 2.3934616 | -0.4258207 |
| H | 3.8989134 | -0.0411785 | -0.2052346 |
| H | 1.8517963 | -1.3599470 | 0.0830295 |
| H | 1.5490440 | 3.1955075 | -1.1860260 |
| H | -0.8726413 | 3.4316400 | -0.2508614 |
| H | -2.9650965 | 2.1299565 | -0.0327259 |

H -2.8518002 -0.3261590 0.1957088

C -0.7316248 -1.8873807 0.2971237

O 0.2050455 -2.5818670 0.6070701

O -1.9519992 -2.4085654 0.0795874

C -2.0478400 -3.8134545 0.2669568

H -1.3801364 -4.3399570 -0.4164198

H -3.0842854 -4.0698521 0.0586335

H -1.7849436 -4.0859424 1.2901548

H 1.6591821 3.3109275 0.5402774

**5b-H6**

E(PBE0-D3/def2-TZVP) = -613.8569212226 (conv)

Lowest Freq. = 35.25 cm^-1

25

5b-H6 (130c1/opt)

C 2.8744426 1.9298754 -0.2901132

C 2.8575864 0.4505813 -0.1585670

C 1.7353388 -0.2649548 -0.0211055

C 0.4232234 0.3480166 0.0023726

C 0.3569646 1.7732238 -0.1505067

C 1.5209083 2.5250587 -0.2895617

C -0.9094236 2.4046673 -0.1569539

C -2.0655244 1.6758989 -0.0312799

C -2.0046977 0.2926972 0.1070178

C -0.7752568 -0.3708336 0.1342084

H 3.4201872 2.2171256 -1.2047800

H 3.8140222 -0.0619926 -0.1732692

H 1.7922498 -1.3375651 0.0859848

H 1.4312278 3.6006491 -0.3994330

H -0.9474940 3.4833110 -0.2656718

H -3.0281222 2.1734394 -0.0382006

H -2.9134438 -0.2834404 0.2053117

C -0.7855418 -1.8451664 0.3325362

O 0.1267684 -2.5207061 0.7388116

O -1.9763696 -2.3796666 0.0165751

C -2.0809124 -3.7817539 0.2241055

H -1.3525827 -4.3167237 -0.3868260
H -3.0935037 -4.0497796 -0.0690951
H -1.9072937 -4.0306228 1.2720268
H 3.4872470 2.3686614 0.5164129

**5b-H7**

E(PBE0-D3/def2-TZVP) = -613.8595573476 (conv)

Lowest Freq. = 23.94 cm^-1

25

5b-H7 (131c1/opt)

C 2.7853460 2.0499506 -0.2466604
C 2.9707849 0.5839086 -0.1107367
C 1.7121576 -0.1906082 -0.0059156
C 0.4503165 0.3923170 -0.0334015
C 0.3580054 1.8208323 -0.1718806
C 1.5700496 2.6014240 -0.2727170
C -0.8754117 2.4463669 -0.2094667
C -2.0524108 1.7163856 -0.1179857
C -1.9925222 0.3363792 0.0135997
C -0.7822936 -0.3416825 0.0608151
H 3.6709030 2.6716217 -0.3256122
H 3.5684878 0.2017199 -0.9556532
H 1.7882804 -1.2619987 0.0994780
H 1.4591165 3.6765840 -0.3734884
H -0.9105884 3.5260148 -0.3129789
H -3.0125703 2.2162765 -0.1479119
H -2.9080041 -0.2336537 0.0857144
C -0.8044559 -1.8125607 0.2167006
O 0.1525539 -2.5359336 0.3618983
O -2.0558002 -2.3080881 0.1850086
C -2.1490052 -3.7155786 0.3454336
H -1.6076511 -4.2322491 -0.4484700
H -3.2103248 -3.9495059 0.2960013
H -1.7350908 -4.0239586 1.3066922
H 3.6101277 0.3660365 0.7615371

**5b-H8**

E(PBE0-D3/def2-TZVP) = -613.8668399424 (conv)

Lowest Freq. = 22.67 cm^-1

25

5b-H8 (132c1/opt)

C 2.8573846 2.0129772 -0.3147258

C 2.9797235 0.6637019 -0.1729488

C 1.8092996 -0.2303558 0.0102571

C 0.4669074 0.4460834 -0.0224168

C 0.4062564 1.8523916 -0.1712021

C 1.6017806 2.6214248 -0.3080320

C -0.8406679 2.4942485 -0.1906093

C -2.0134603 1.7806113 -0.0762109

C -1.9635576 0.4039828 0.0557443

C -0.7368397 -0.2654825 0.0854694

H 3.7440070 2.6258345 -0.4356163

H 3.9589676 0.1986764 -0.1790550

H 1.8340945 -1.0378271 -0.7329060

H 1.5108471 3.6953509 -0.4187810

H -0.8650641 3.5733490 -0.3002257

H -2.9693819 2.2903246 -0.0916663

H -2.8751805 -0.1696346 0.1412497

C -0.7422067 -1.7426489 0.2426565

O 0.2243107 -2.4383421 0.4353405

O -1.9783829 -2.2583089 0.1487721

C -2.0566377 -3.6682409 0.3071478

H -1.4664801 -4.1752855 -0.4574289

H -3.1098208 -3.9197319 0.2038227

H -1.6869785 -3.9673997 1.2889624

H 1.9110797 -0.7856991 0.9524026

**5c**

E(PBE0-D3/def2-TZVP) = -477.7531509349 (conv)

Lowest Freq. = 87.67 cm^-1

19

5c (093c1/opt)

C 2.6770596 0.8098079 0.0000000
C 2.6759297 -0.5985601 0.0000000
C 1.4952304 -1.2871457 0.0000000
C 0.2618764 -0.5985749 0.0000000
C 0.2627819 0.8236820 0.0000000
C 1.4985096 1.5040610 0.0000000
C -0.9728254 1.5094831 0.0000000
C -2.1553328 0.8306980 0.0000000
C -2.1541945 -0.5854391 0.0000000
C -0.9687103 -1.2820260 0.0000000
H 3.6202530 1.3433726 0.0000000
H 3.6174490 -1.1347737 0.0000000
H 1.4884136 -2.3715720 0.0000000
H 1.4980973 2.5885539 0.0000000
H -0.9687881 2.5937742 0.0000000
H -3.1010641 1.3574127 0.0000000
H -0.9780337 -2.3656861 0.0000000
C -3.3950981 -1.2883343 0.0000000
N -4.4015533 -1.8487334 0.0000000

**5c-H1**

E(PBE0-D3/def2-TZVP) = -478.3183610339 (conv)

Lowest Freq. = 49.16 cm^-1

20

5c-H1 (111c1/opt)

C 2.7544748 0.8997523 -0.0284483
C 2.7620065 -0.4909698 0.0011770
C 1.5647604 -1.1891673 0.0222513
C 0.3479636 -0.5239104 0.0141155
C 0.3348667 0.8830447 -0.0167988
C 1.5531680 1.5766111 -0.0373481
C -0.9131486 1.5839690 -0.0295679
C -2.1201083 0.9106881 -0.0171071
C -2.1725760 -0.4711082 0.0130944
C -0.9322245 -1.3069420 0.0399994
H 3.6882224 1.4490606 -0.0448882

H 3.7016670 -1.0303683 0.0076875
H 1.5757579 -2.2742622 0.0454377
H 1.5364955 2.6609455 -0.0611719
H -0.9002812 2.6670744 -0.0531232
H -3.0494388 1.4676713 -0.0322157
H -0.9515512 -2.0095118 -0.8042264
C -3.4123979 -1.1389165 0.0216966
N -4.4162188 -1.7138715 0.0283733
H -0.9514375 -1.9497888 0.9310628

**5c-H3**

E(PBE0-D3/def2-TZVP) = -478.3069408201 (conv)

Lowest Freq. = 76.04 cm^-1

20

5c-H3 (112c1/opt)

C 2.8256945 0.7463717 -0.0212861
C 2.8122396 -0.6539451 -0.0434049
C 1.6037599 -1.3292335 -0.0405250
C 0.3974375 -0.6371978 -0.0155351
C 0.3980552 0.7936431 0.0076159
C 1.6507107 1.4563856 0.0034880
C -0.8050792 1.4884943 0.0332720
C -2.1299862 0.8236668 0.0395110
C -2.0392675 -0.6732890 0.0128591
C -0.8603054 -1.3262180 -0.0123056
H 3.7727139 1.2735181 -0.0237558
H 3.7438005 -1.2057944 -0.0627961
H 1.5863011 -2.4138686 -0.0577994
H 1.6652863 2.5406232 0.0205953
H -0.7866741 2.5726845 0.0504107
H -2.7369174 1.1678191 -0.8127158
H -0.8524706 -2.4107947 -0.0307655
C -3.2602887 -1.3976932 0.0154878
N -4.2712786 -1.9534391 0.0186687
H -2.7137316 1.1382669 0.9189809

**5c-H4**

E(PBE0-D3/def2-TZVP) = -478.3111482965 (conv)

Lowest Freq. = 57.07 cm^-1

20

5c-H4 (113c1/opt)

C 2.7627174 0.6881564 0.0013631

C 2.7473203 -0.7029876 -0.0328926

C 1.5438334 -1.3741188 -0.0418694

C 0.3261058 -0.6746934 -0.0166574

C 0.3475619 0.7346291 0.0189608

C 1.5682394 1.3915801 0.0269855

C -0.9321085 1.5216794 0.0490607

C -2.1695977 0.7057309 0.0275419

C -2.1288115 -0.6650419 -0.0080199

C -0.9156132 -1.3721458 -0.0287401

H 3.7051731 1.2225753 0.0080323

H 3.6780892 -1.2572781 -0.0528300

H 1.5219881 -2.4581521 -0.0692340

H 1.5851454 2.4766615 0.0538711

H -0.9501799 2.2284036 -0.7942956

H -3.1256066 1.2140367 0.0423295

H -0.9237980 -2.4539579 -0.0567389

C -3.3557409 -1.3999171 -0.0253945

N -4.3408623 -1.9958690 -0.0395489

H -0.9438553 2.1707088 0.9380763

**5c-H5**

E(PBE0-D3/def2-TZVP) = -478.3119951054 (conv)

Lowest Freq. = 79.42 cm^-1

20

5c-H5 (133c1/opt)

C 2.6710945 0.6686445 0.0209545

C 2.6213256 -0.6945085 -0.0106234

C 1.4070118 -1.3848874 -0.0262230

C 0.1656399 -0.6785805 -0.0136664

C 0.1548726 0.7321008 0.0192665

| | | | |
|---|---|---|---|
| C | 1.4423634 | 1.5047197 | 0.0448064 |
| C | -1.0592786 | 1.4040243 | 0.0290839 |
| C | -2.2627752 | 0.7242627 | 0.0077450 |
| C | -2.2572102 | -0.6736018 | -0.0244854 |
| C | -1.0549966 | -1.3640084 | -0.0349795 |
| H | 3.6268327 | 1.1795776 | 0.0319067 |
| H | 3.5468397 | -1.2595726 | -0.0245969 |
| H | 1.3904383 | -2.4675644 | -0.0507318 |
| H | 1.4579182 | 2.2104437 | -0.7993973 |
| H | -1.0634795 | 2.4889013 | 0.0540548 |
| H | -3.2040365 | 1.2584305 | 0.0155349 |
| H | -1.0590410 | -2.4473714 | -0.0602368 |
| C | -3.4909334 | -1.3900319 | -0.0467620 |
| N | -4.4883912 | -1.9657861 | -0.0646591 |
| H | 1.4558055 | 2.1548081 | 0.9330088 |

**5c-H6**

E(PBE0-D3/def2-TZVP) = -478.3066046684 (conv)

Lowest Freq. = 78.17 cm^-1

20

5c-H6 (134c1/opt)

| | | | |
|---|---|---|---|
| C | 2.6241942 | 0.7842501 | 0.0439245 |
| C | 2.5264806 | -0.7008862 | 0.0275947 |
| C | 1.3555531 | -1.3445252 | 0.0051195 |
| C | 0.0927154 | -0.6425526 | -0.0054399 |
| C | 0.1043669 | 0.7906796 | 0.0082386 |
| C | 1.3116042 | 1.4712281 | 0.0319549 |
| C | -1.1419148 | 1.4710249 | -0.0028965 |
| C | -2.3255759 | 0.7862555 | -0.0254030 |
| C | -2.3248723 | -0.6222528 | -0.0378664 |
| C | -1.1126983 | -1.3175784 | -0.0279066 |
| H | 3.2362032 | 1.1252128 | -0.8078519 |
| H | 3.4542395 | -1.2628319 | 0.0348054 |
| H | 1.3287145 | -2.4292884 | -0.0058943 |
| H | 1.3021433 | 2.5560714 | 0.0425643 |
| H | -1.1426738 | 2.5552097 | 0.0069617 |

H -3.2706351 1.3146432 -0.0335210
H -1.1219069 -2.4014681 -0.0377568
C -3.5536127 -1.3326914 -0.0596298
N -4.5535273 -1.9081956 -0.0768149
H 3.2112021 1.1076952 0.9198176

**5c-H7**

E(PBE0-D3/def2-TZVP) = -478.3043308636 (conv)

Lowest Freq. = 87.02 cm^-1

20

5c-H7 (135c1/opt)

C 2.5300080 0.8989205 0.0182798
C 2.6262366 -0.5866695 0.0391003
C 1.3108633 -1.2695494 0.0310167
C 0.1020392 -0.5776332 0.0082782
C 0.0953561 0.8526636 -0.0093507
C 1.3608285 1.5472044 -0.0037020
C -1.1143586 1.5346872 -0.0315363
C -2.3226621 0.8612904 -0.0374609
C -2.3268576 -0.5434779 -0.0209556
C -1.1392182 -1.2509990 0.0015331
H 3.4590839 1.4590211 0.0216240
H 3.2376739 -0.9291890 -0.8125816
H 1.2946784 -2.3539130 0.0436162
H 1.3398336 2.6321737 -0.0179576
H -1.1058951 2.6191860 -0.0444189
H -3.2613019 1.3992052 -0.0546159
H -1.1580990 -2.3342863 0.0142795
C -3.5690578 -1.2449174 -0.0275684
N -4.5743473 -1.8075249 -0.0329308
H 3.2151961 -0.9061925 0.9153510

**5c-H8**

E(PBE0-D3/def2-TZVP) = -478.3134158042 (conv)

Lowest Freq. = 63.99 cm^-1

20

5c-H8 (136c1/opt)

| | | | |
|---|---|---|---|
| C | 2.6227845 | 0.8981801 | -0.0047861 |
| C | 2.6701897 | -0.4632262 | 0.0188705 |
| C | 1.4408055 | -1.2975828 | 0.0347141 |
| C | 0.1541735 | -0.5211706 | 0.0119617 |
| C | 0.1670351 | 0.8919826 | -0.0122580 |
| C | 1.4061263 | 1.5905289 | -0.0184214 |
| C | -1.0583107 | 1.5820061 | -0.0315503 |
| C | -2.2568050 | 0.9113532 | -0.0282364 |
| C | -2.2632673 | -0.4883346 | -0.0049424 |
| C | -1.0556188 | -1.1875943 | 0.0152859 |
| H | 3.5484931 | 1.4629744 | -0.0136607 |
| H | 3.6250411 | -0.9760110 | 0.0286568 |
| H | 1.4604213 | -1.9964864 | -0.8146413 |
| H | 1.3935699 | 2.6736172 | -0.0364455 |
| H | -1.0464468 | 2.6660690 | -0.0502404 |
| H | -3.1955329 | 1.4504783 | -0.0439299 |
| H | -1.0760060 | -2.2718345 | 0.0336232 |
| C | -3.4963267 | -1.1981659 | -0.0021523 |
| N | -4.4953969 | -1.7735495 | -0.0001743 |
| H | 1.4550711 | -1.9532340 | 0.9183268 |

# 13. NMR spectra

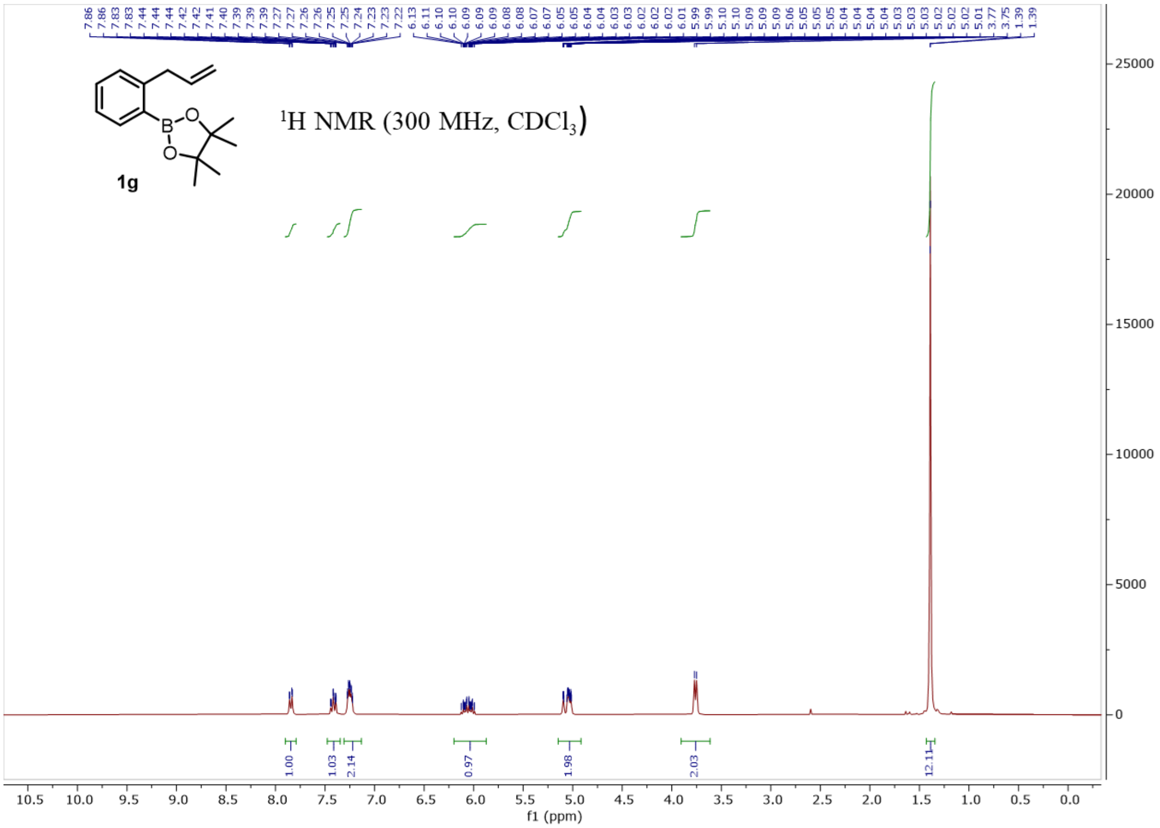


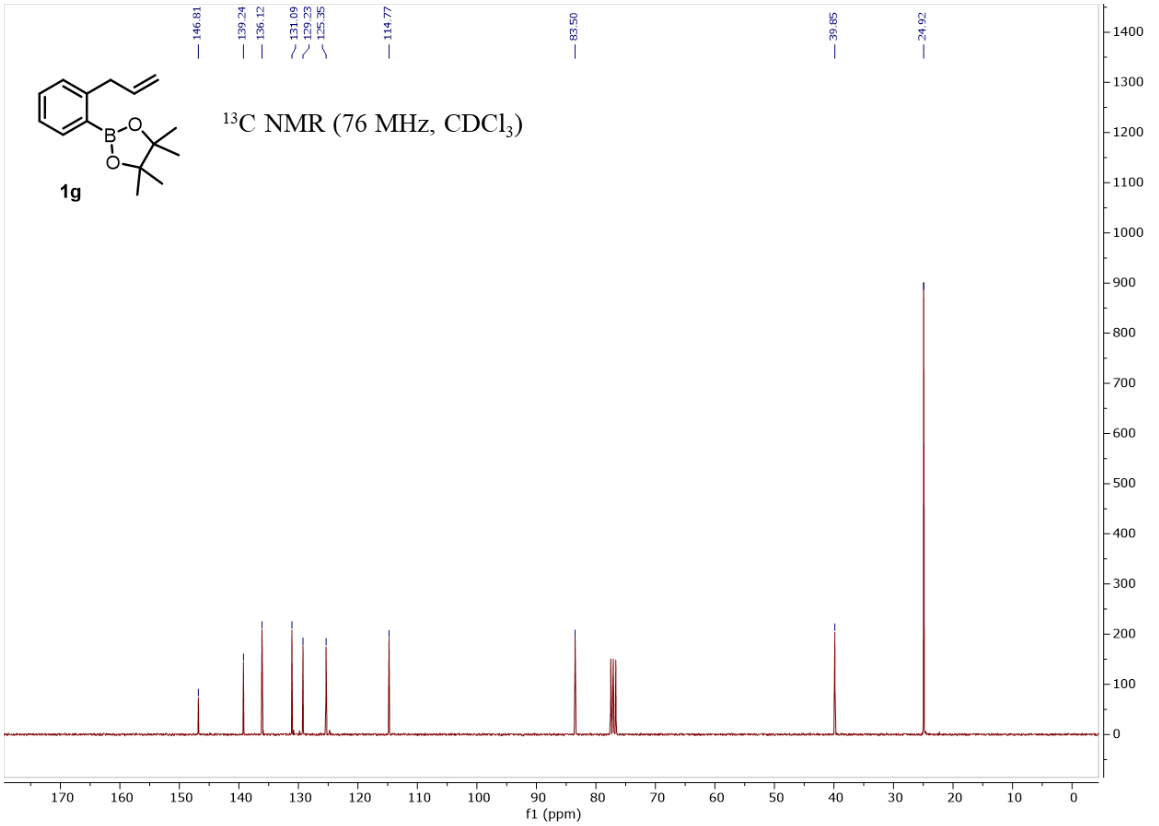

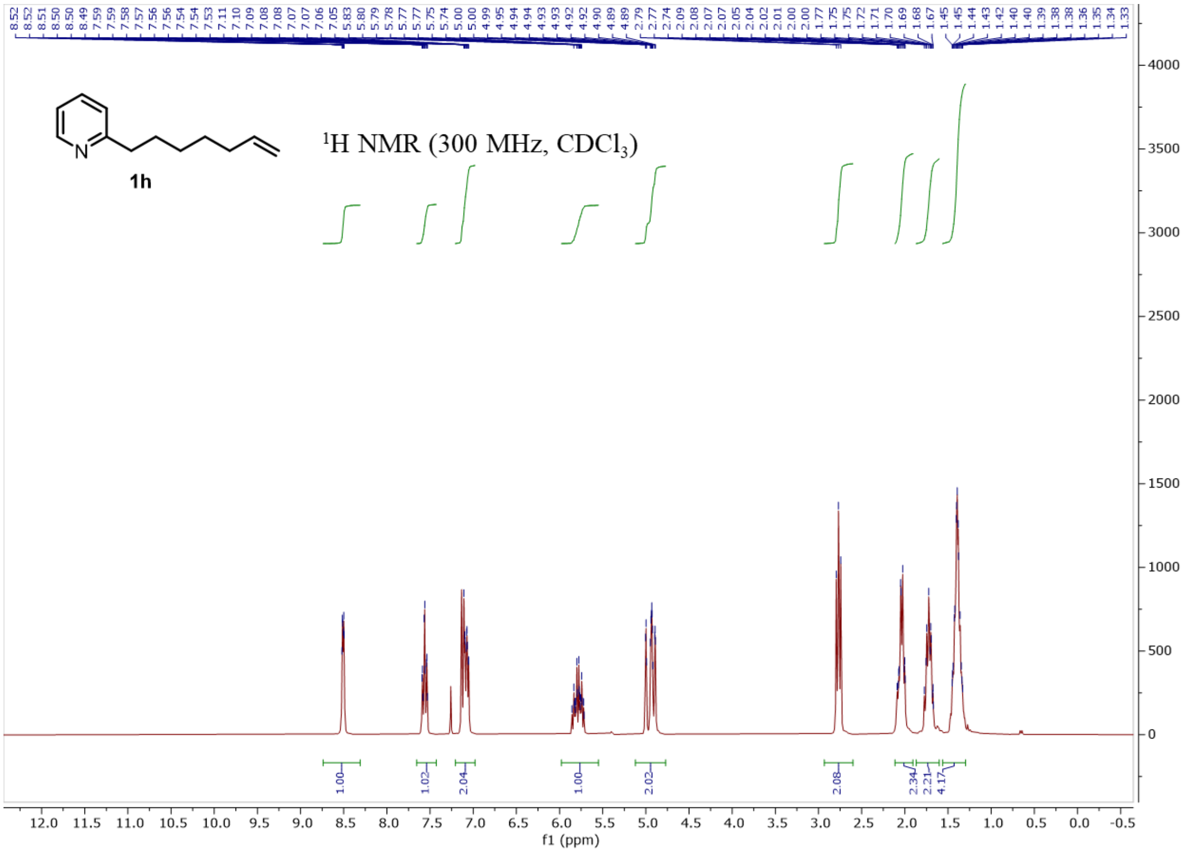
1h
1H NMR (300 MHz, CDCl3)
f1 (ppm)

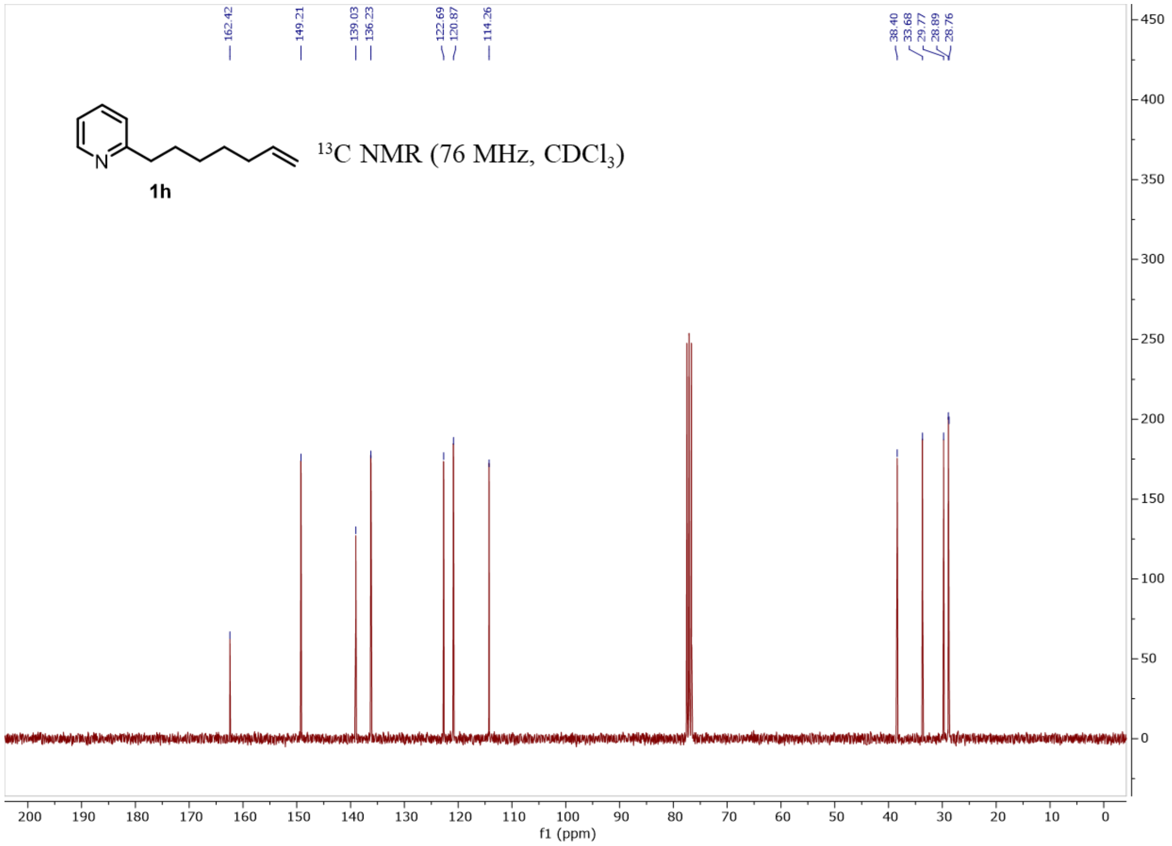
1h
13C NMR (76 MHz, CDCl3)
162.42
149.21
139.03
136.23
122.69
120.87
114.26
38.40
33.68
29.77
28.89
28.76
f1 (ppm)

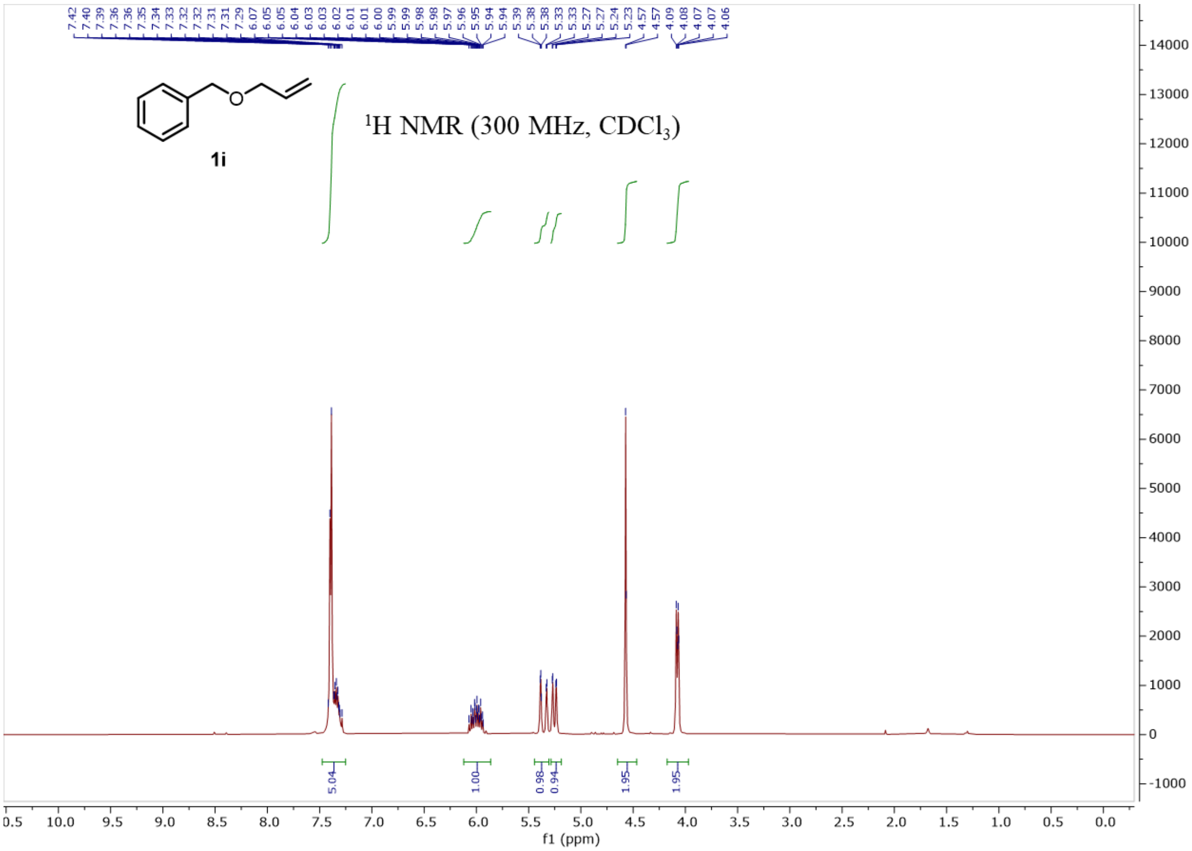
1i
$^1$H NMR (300 MHz, CDCl$_3$)
f1 (ppm)

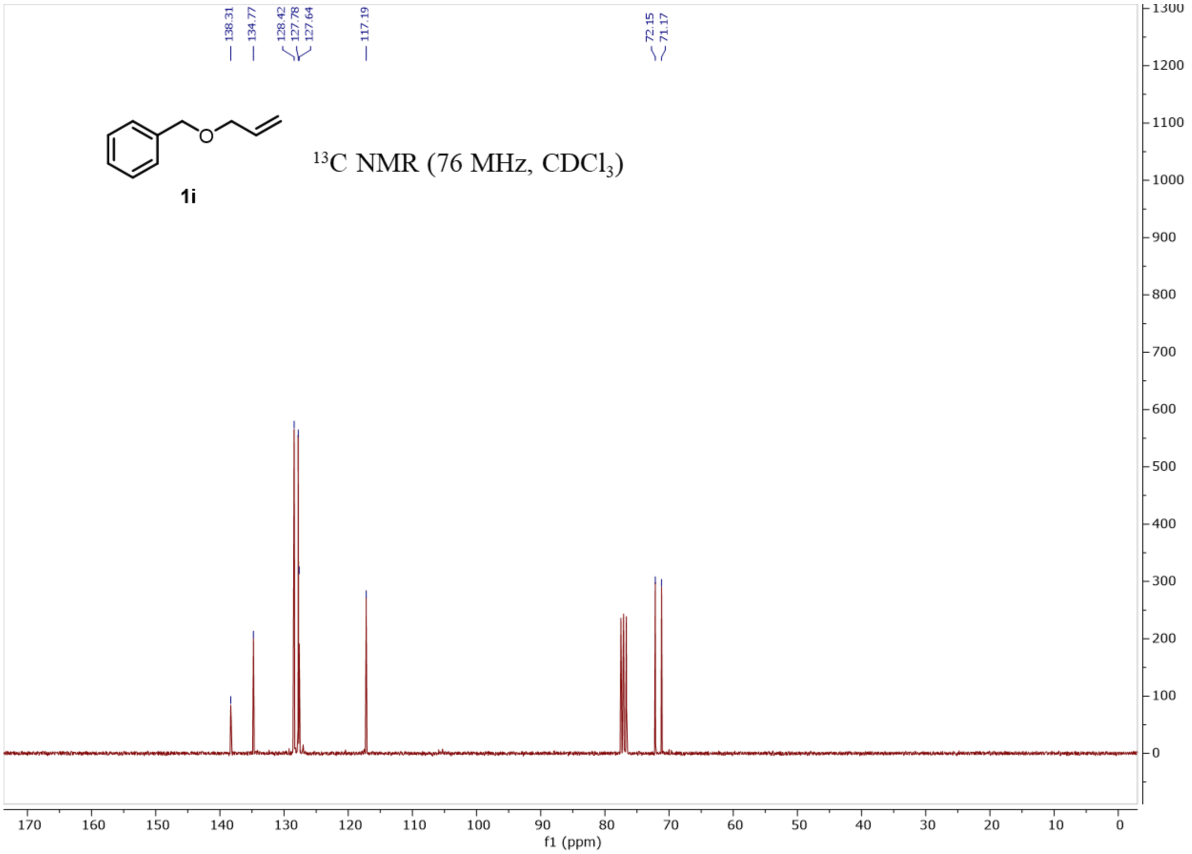
1i
$^{13}$C NMR (76 MHz, CDCl$_3$)
138.31
134.77
128.42
127.78
127.64
117.19
72.15
71.17
f1 (ppm)

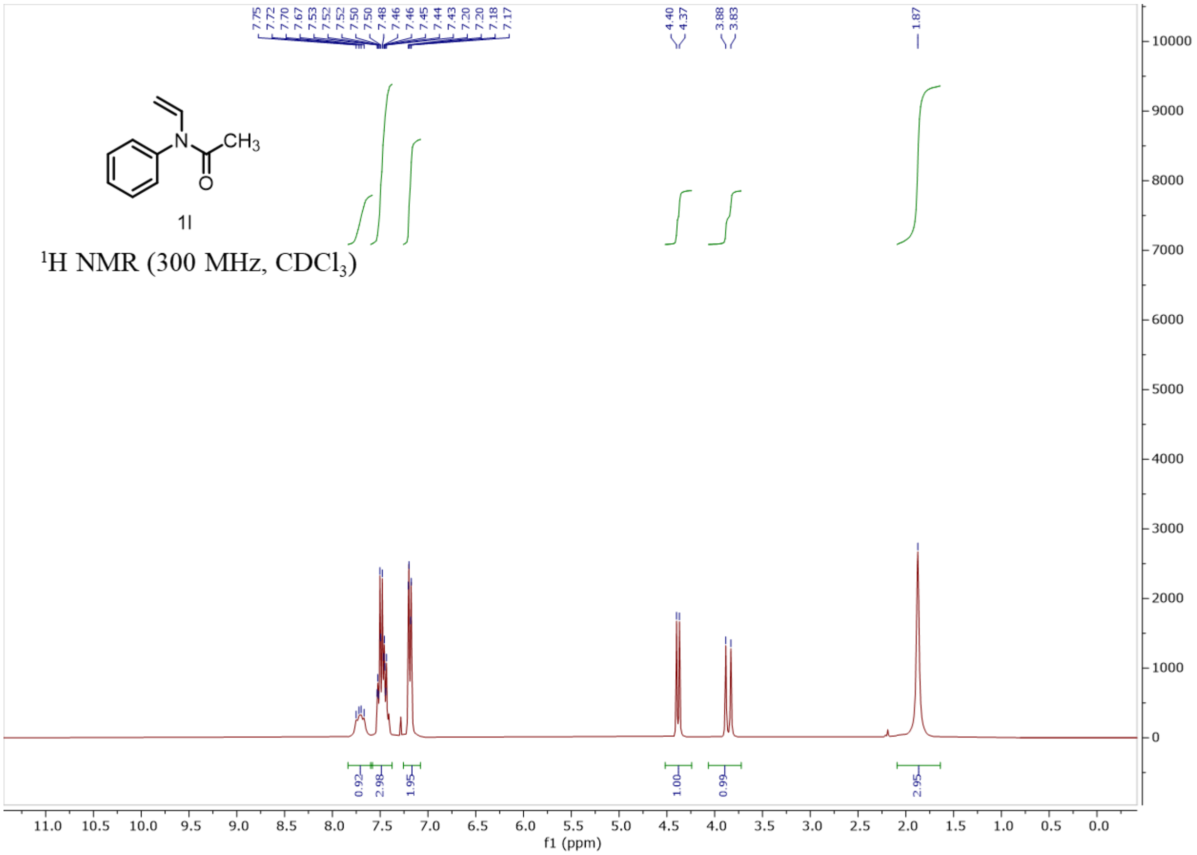
7.75 7.72 7.70 7.67 7.53 7.52 7.52 7.50 7.50 7.48 7.46 7.46 7.45 7.44 7.43 7.20 7.20 7.18 7.17
4.40 4.37
3.88 3.83
1.87
CH3
N
O
1l
1H NMR (300 MHz, CDCl3)
0.92 2.98 1.95 1.00 0.99 2.95
f1 (ppm)

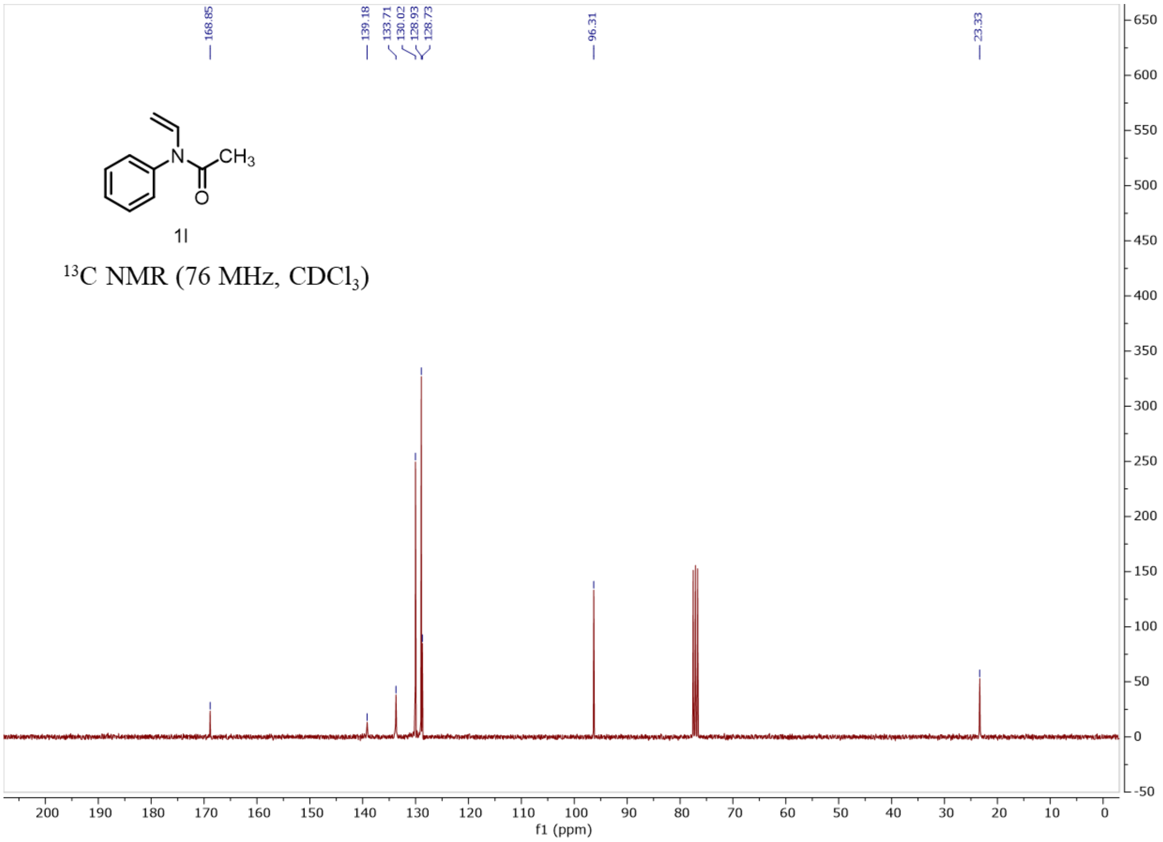
168.85
139.18 133.71 130.02 128.93 128.73
96.31
23.33
CH3
N
O
1l
13C NMR (76 MHz, CDCl3)
f1 (ppm)

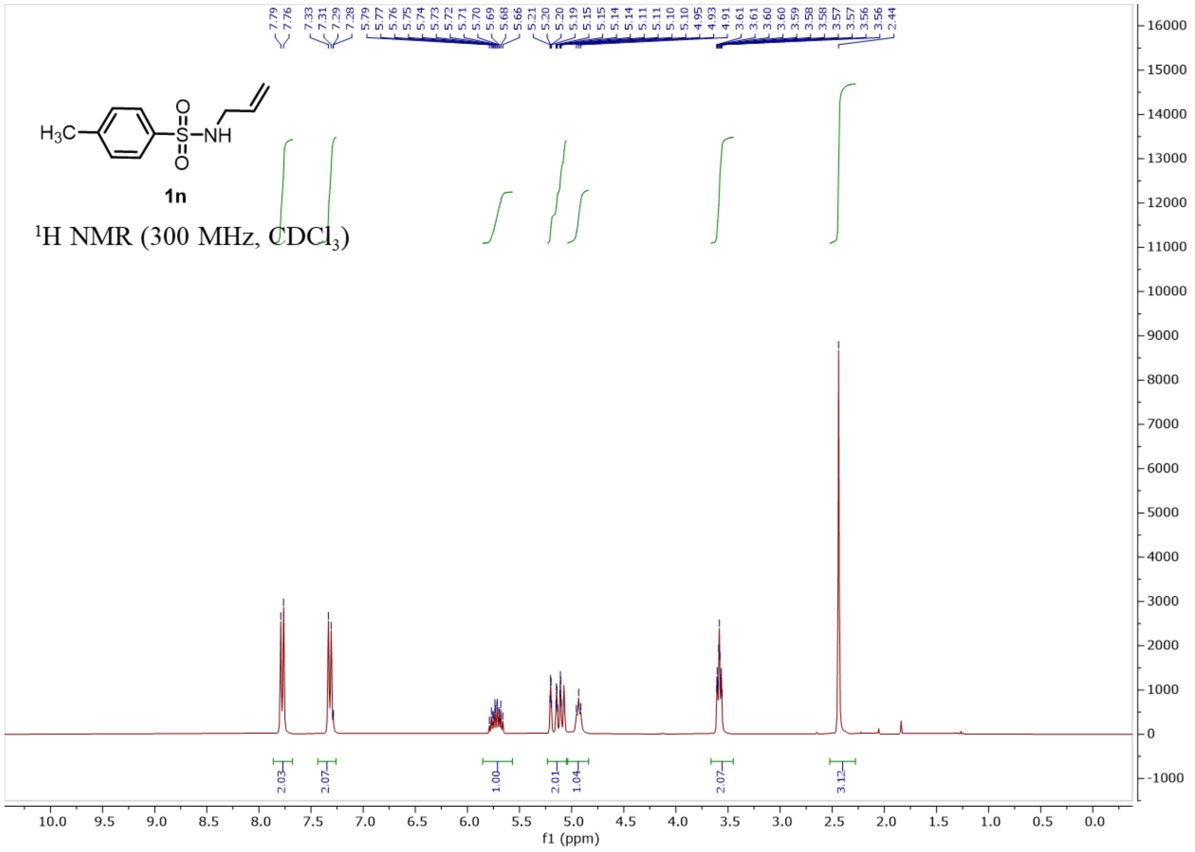

H3C
O
S
NH
O
1n
1H NMR (300 MHz, CDCl3)
f1 (ppm)


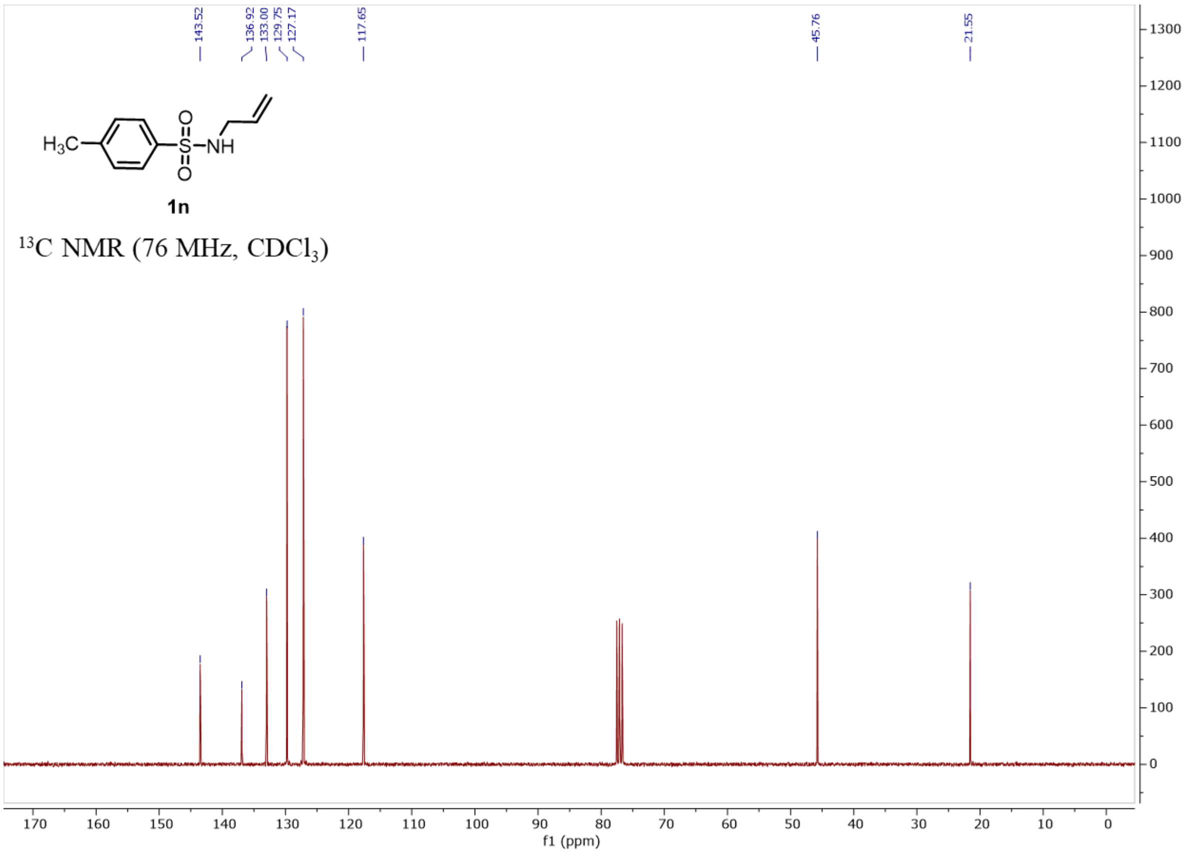

143.52
136.92
133.00
129.75
127.17
117.65
45.76
21.55
H3C
O
S
NH
O
1n
13C NMR (76 MHz, CDCl3)
f1 (ppm)

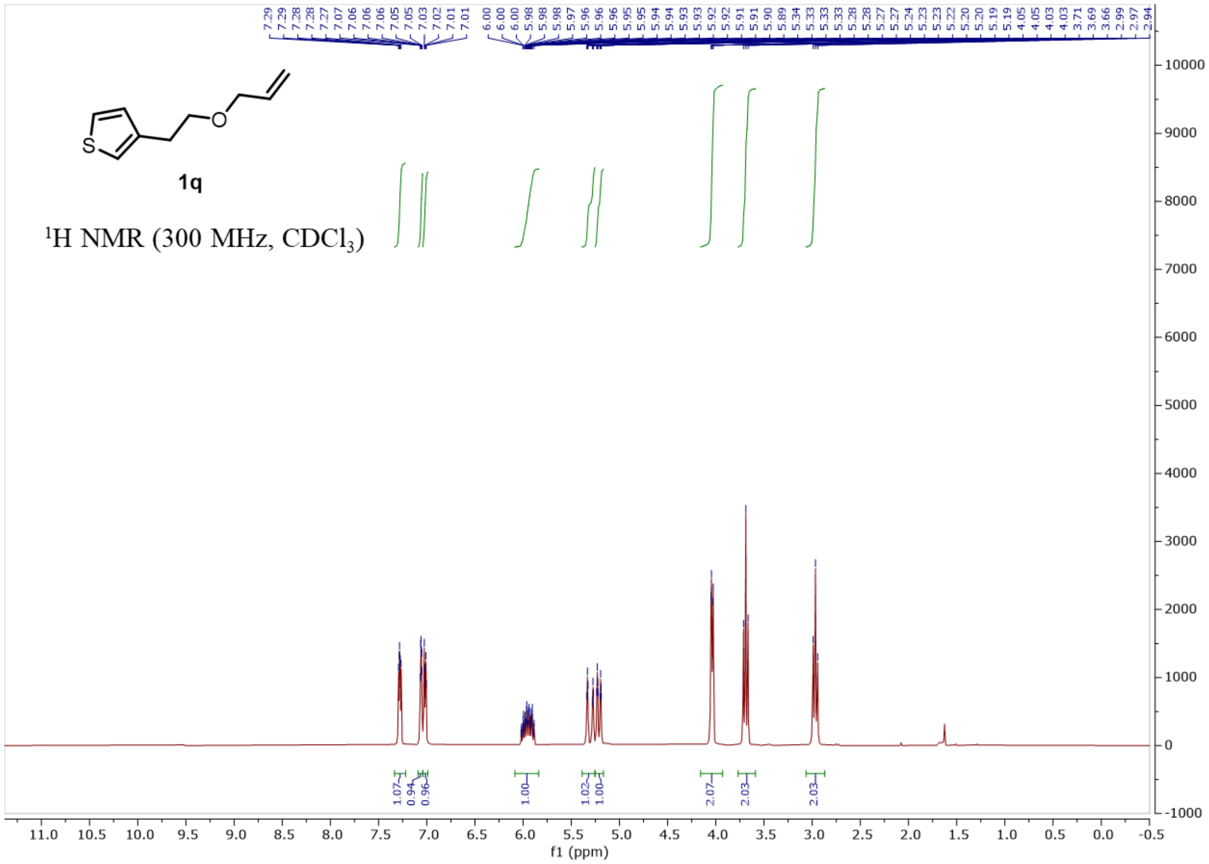

1q
¹H NMR (300 MHz, CDCl₃)
f1 (ppm)


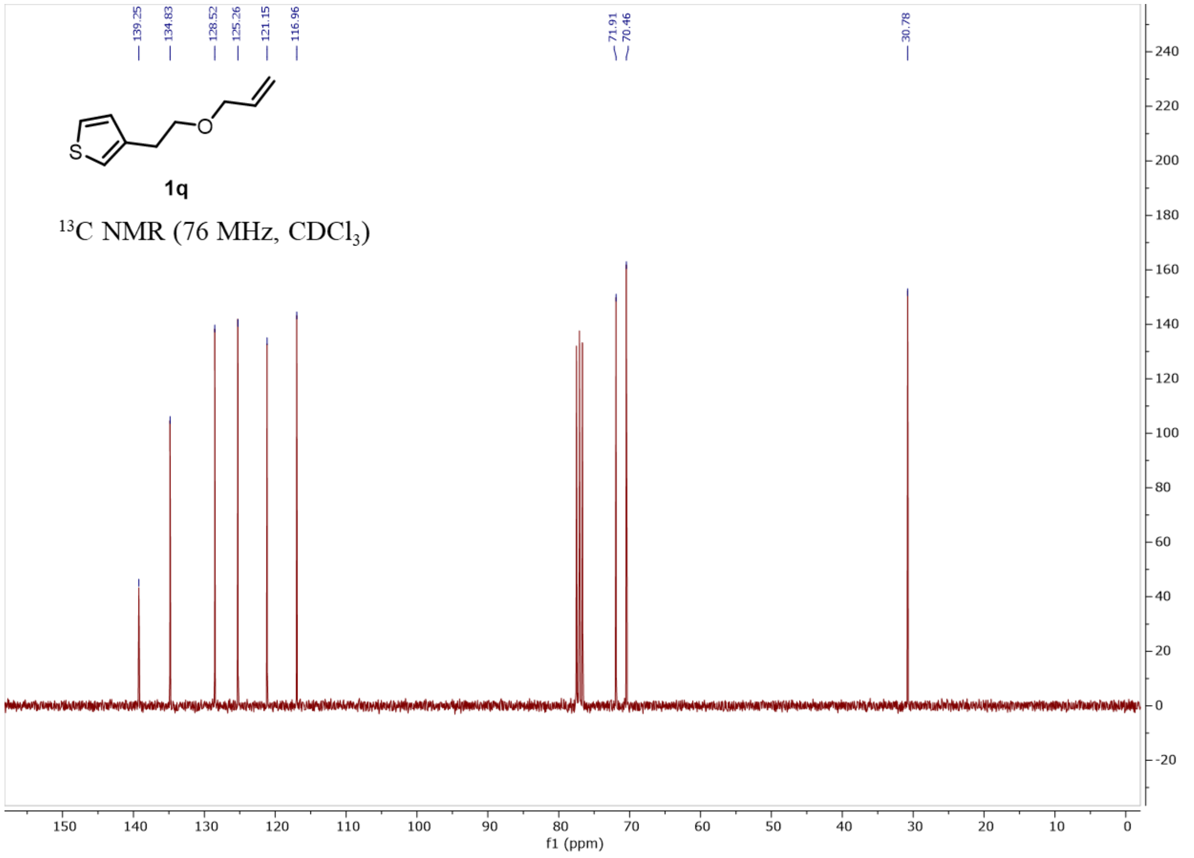

1q
¹³C NMR (76 MHz, CDCl₃)
f1 (ppm)

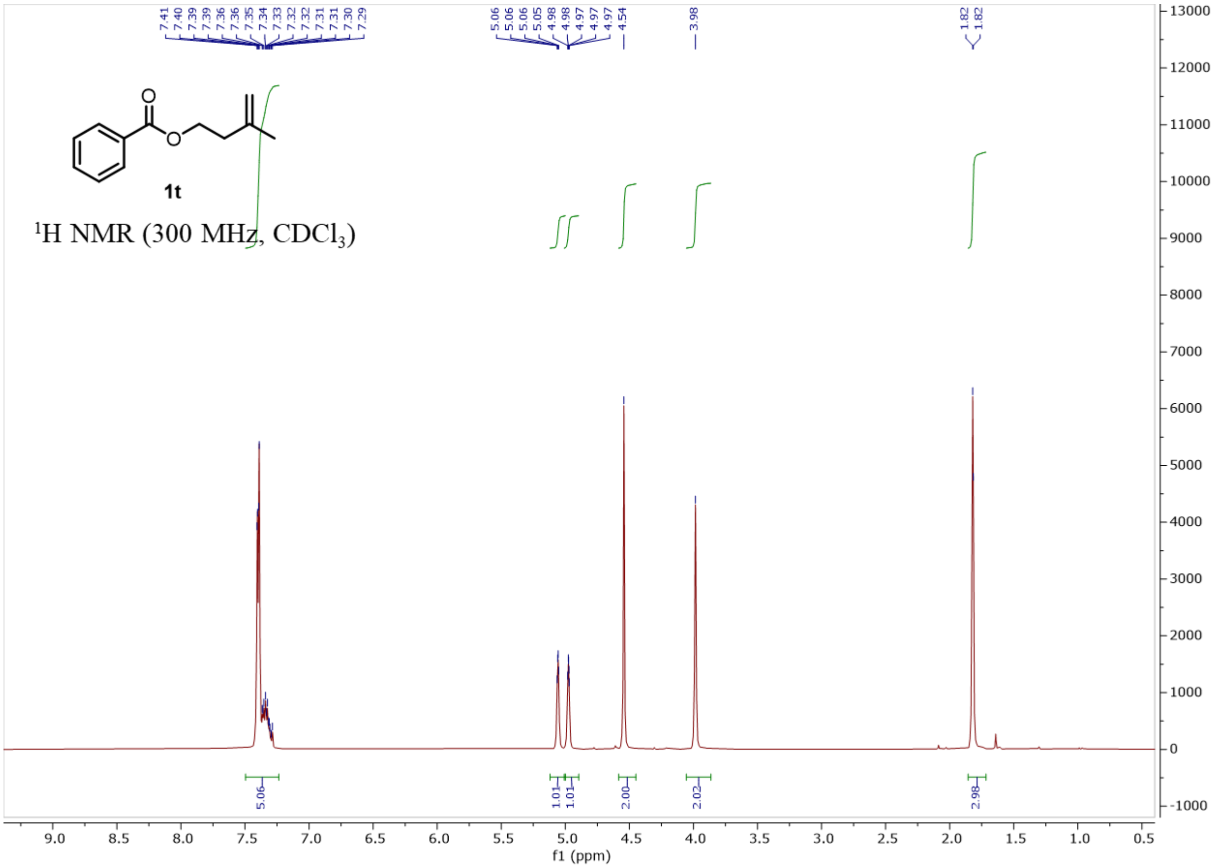
1t
1H NMR (300 MHz, CDCl3)
7.41 7.40 7.39 7.39 7.36 7.36 7.35 7.34 7.33 7.32 7.32 7.31 7.31 7.30 7.29
5.06 5.06 5.06 5.05 4.98 4.98 4.97 4.97 4.97 4.54
3.98
1.82 1.82
5.06
1.01 1.01
2.00
2.02
2.98
f1 (ppm)

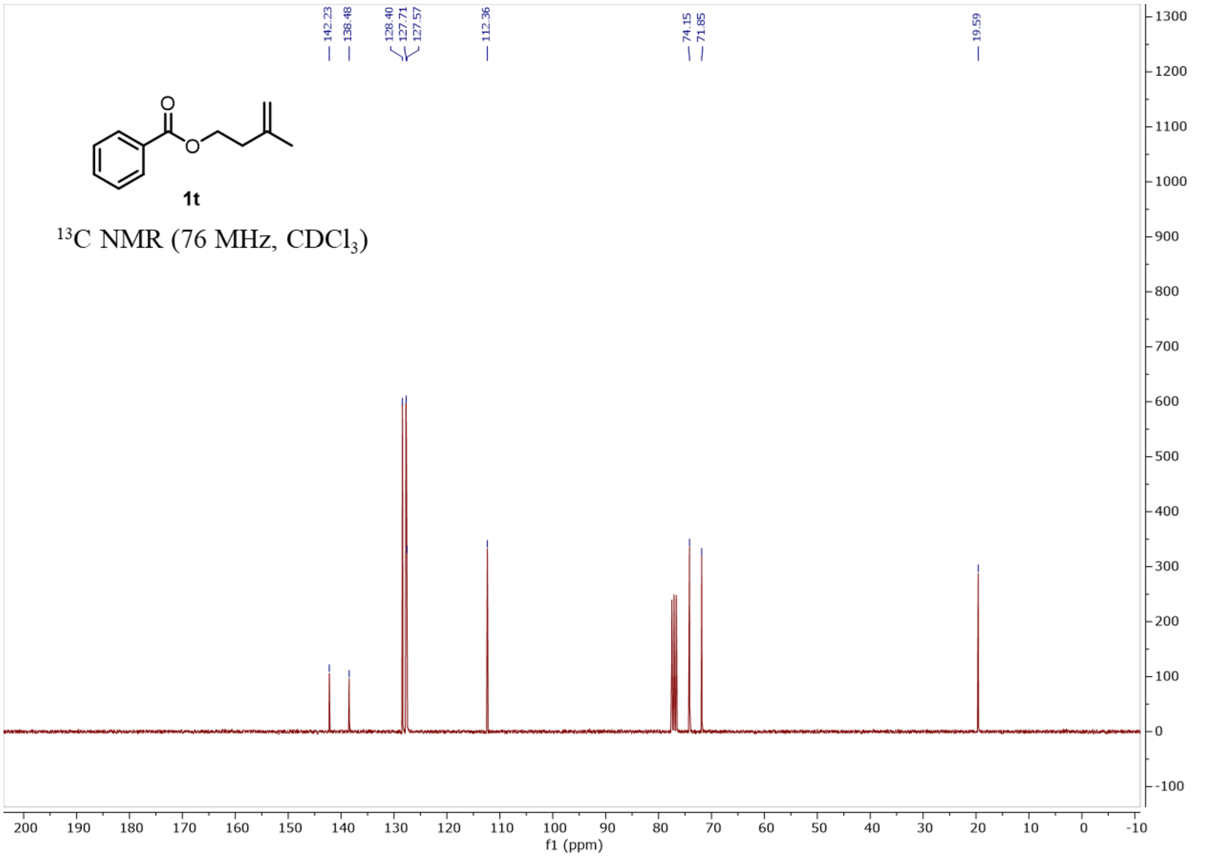
1t
13C NMR (76 MHz, CDCl3)
142.23 138.48 128.40 127.71 127.57 112.36
74.15 71.85
19.59
f1 (ppm)

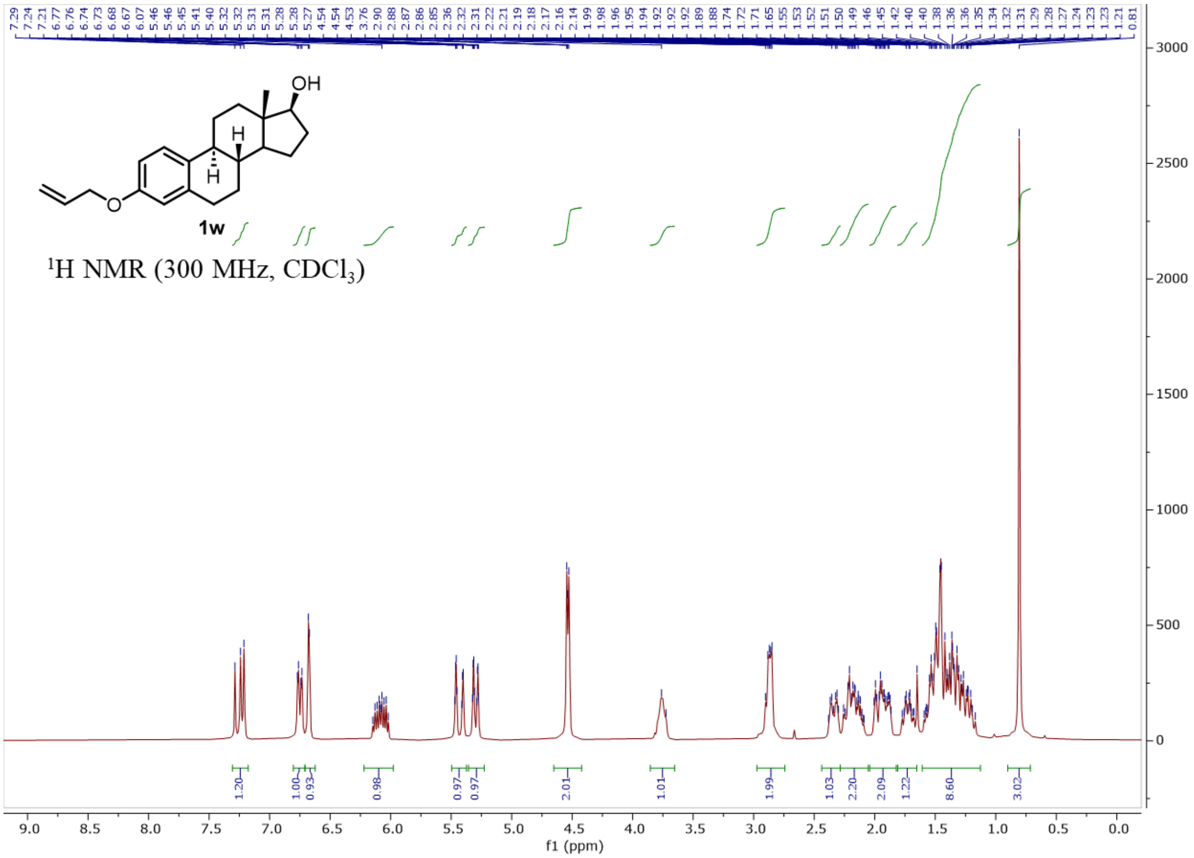

1w
$^1$H NMR (300 MHz, $CDCl_3$)
f1 (ppm)


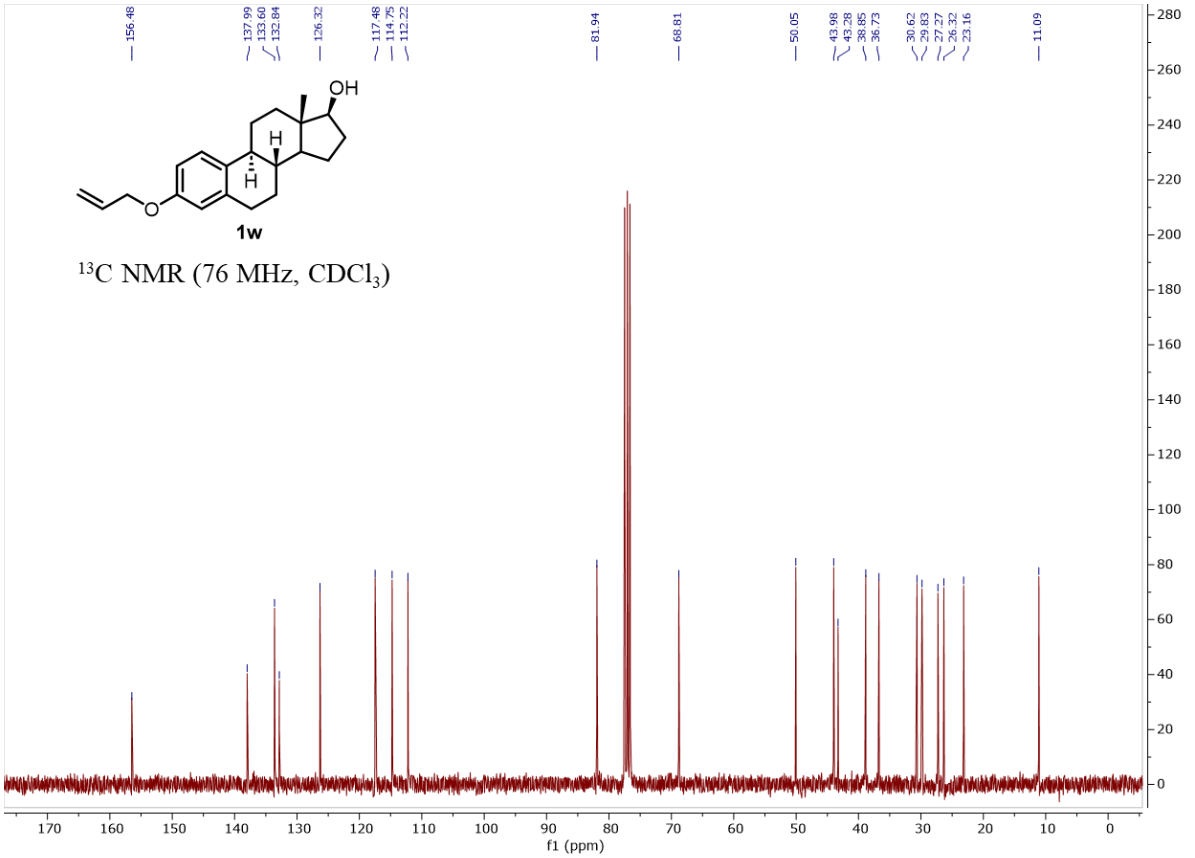

1w
$^{13}$C NMR (76 MHz, $CDCl_3$)
156.48
137.99
133.60
132.84
126.32
117.48
114.75
112.22
81.94
68.81
50.05
43.98
43.28
38.85
36.73
30.62
29.83
27.27
26.32
23.16
11.09
f1 (ppm)

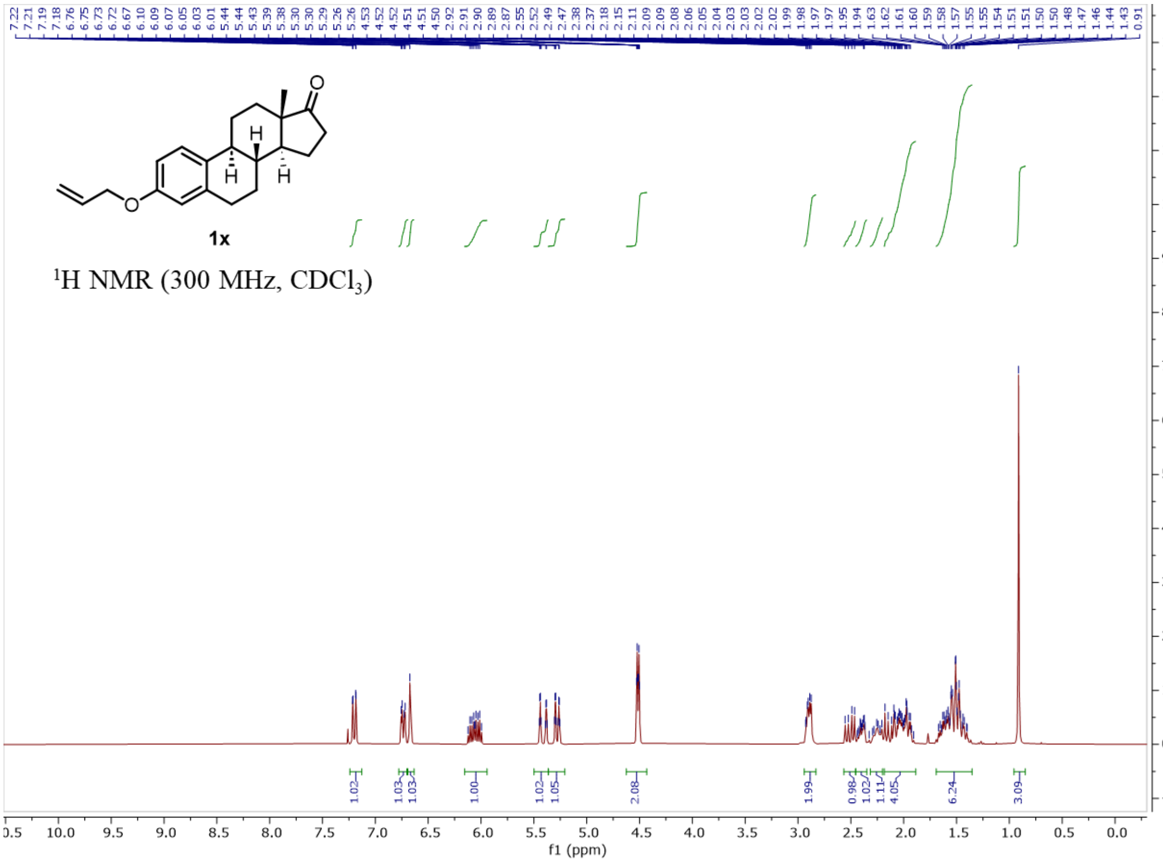
1x
$^{1}$H NMR (300 MHz, CDCl3)
7.22 7.21 7.19 7.18 6.76 6.75 6.73 6.72 6.67 6.10 6.09 6.07 6.05 6.03 6.01 5.44 5.44 5.43 5.39 5.38 5.30 5.30 5.29 5.26 5.26 4.53 4.52 4.52 4.51 4.51 4.50 2.92 2.91 2.90 2.89 2.87 2.55 2.52 2.49 2.47 2.38 2.37 2.18 2.15 2.11 2.09 2.09 2.08 2.06 2.05 2.04 2.03 2.03 2.02 2.02 1.99 1.98 1.97 1.97 1.95 1.94 1.63 1.62 1.61 1.60 1.59 1.58 1.57 1.55 1.55 1.54 1.51 1.51 1.50 1.50 1.48 1.47 1.46 1.44 1.43 0.91
1.02 1.03 1.03 1.00 1.02 1.05 2.08 1.99 0.98 1.02 1.11 4.05 6.24 3.09
f1 (ppm)

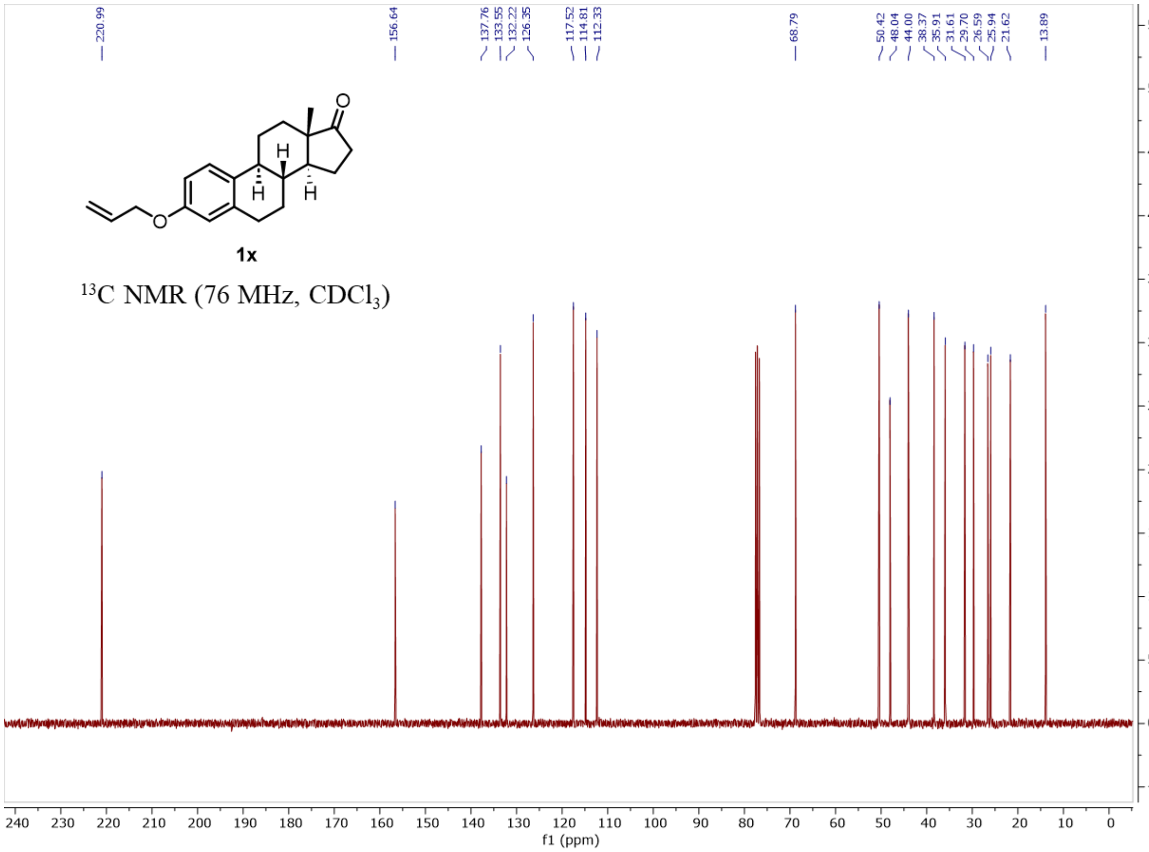
1x
$^{13}$C NMR (76 MHz, CDCl3)
220.99 156.64 137.76 133.55 132.22 126.35 117.52 114.81 112.33 68.79 50.42 48.04 44.00 38.37 35.91 31.61 29.70 26.59 25.94 21.62 13.89
f1 (ppm)

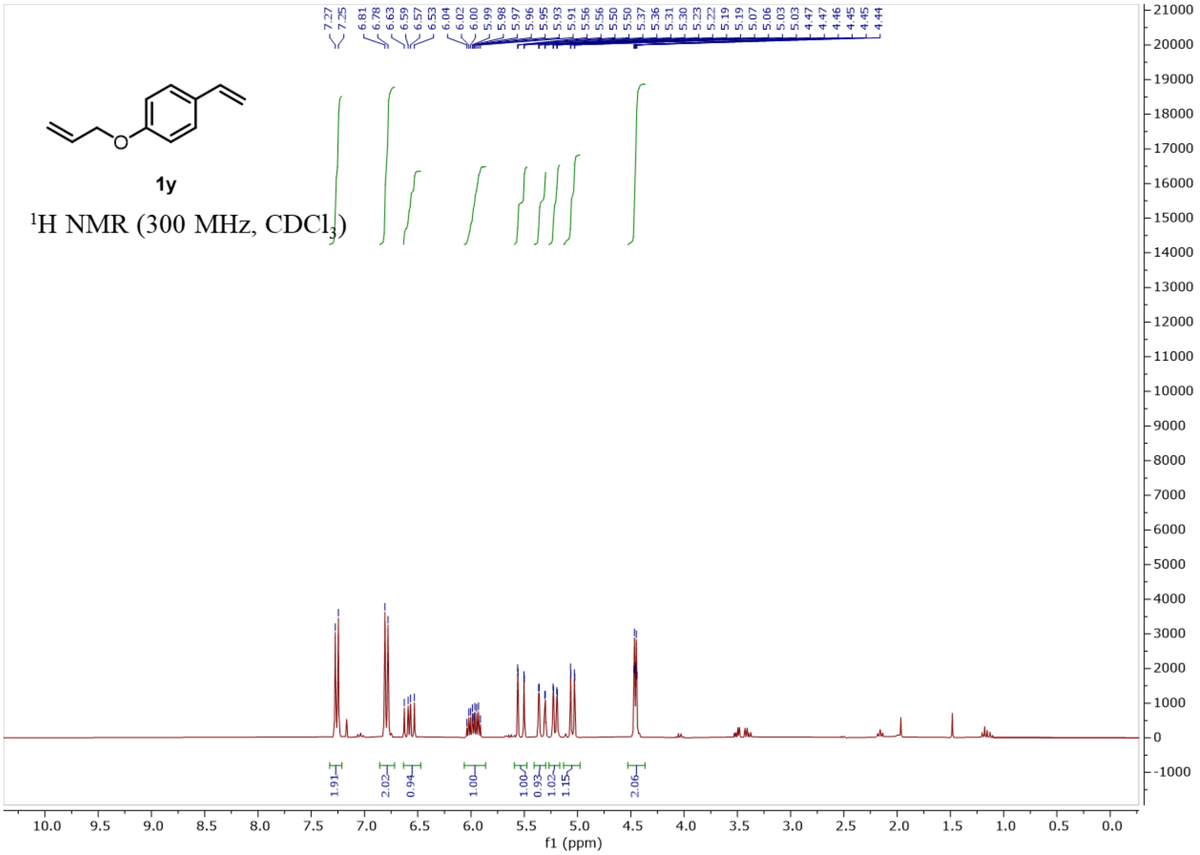
7.27 7.25 6.81 6.78 6.63 6.59 6.57 6.53 6.04 6.02 6.00 5.99 5.98 5.97 5.96 5.95 5.93 5.91 5.56 5.56 5.50 5.50 5.37 5.36 5.31 5.30 5.23 5.22 5.19 5.07 5.06 5.03 5.03 4.47 4.46 4.45 4.45 4.44
O
1y
1H NMR (300 MHz, CDCl3)
1.91 2.02 0.94 1.00 1.00 0.93 1.02 1.15 2.06
f1 (ppm)

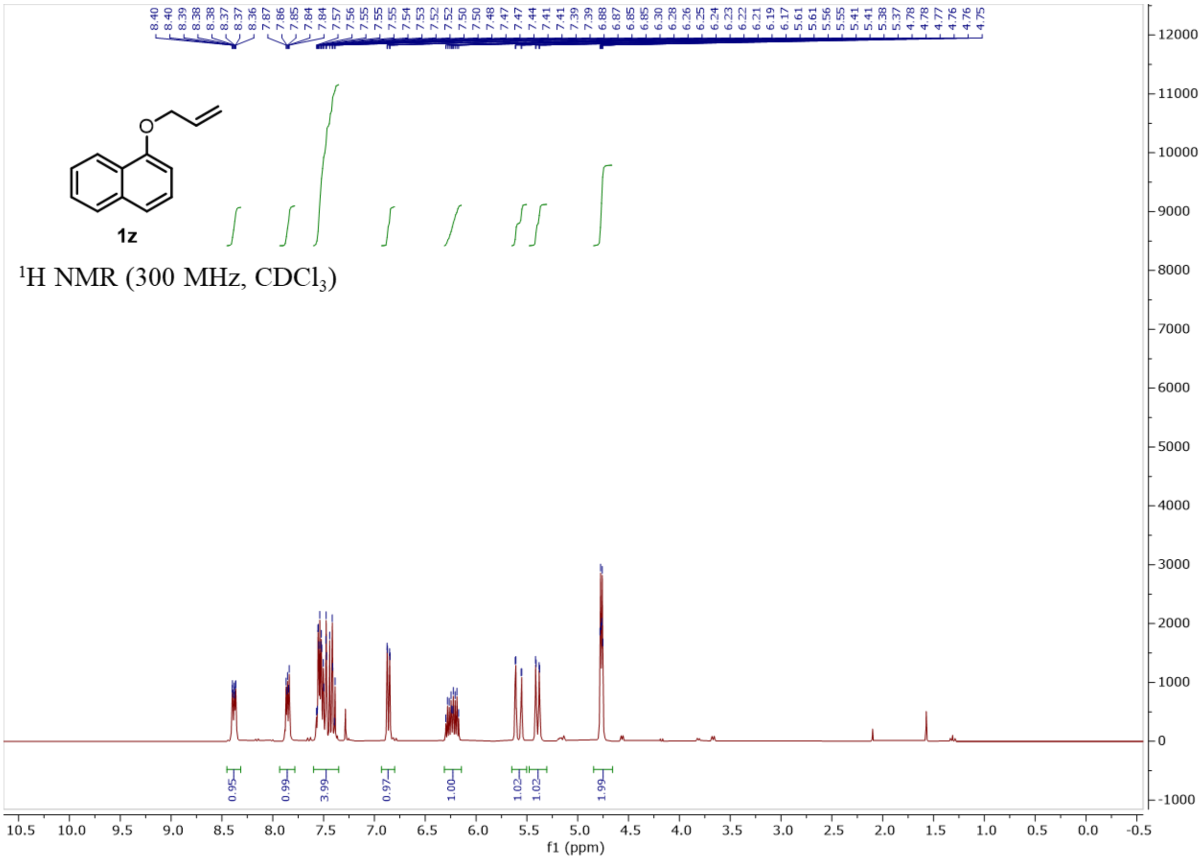
8.40 8.40 8.39 8.38 8.38 8.37 8.36 7.87 7.86 7.85 7.84 7.57 7.56 7.55 7.55 7.54 7.53 7.52 7.52 7.50 7.50 7.48 7.47 7.44 7.41 7.39 7.39 6.88 6.87 6.85 6.85 6.30 6.28 6.26 6.25 6.24 6.23 6.22 6.21 6.19 6.17 5.61 5.61 5.56 5.55 5.41 5.38 5.38 5.37 4.78 4.78 4.77 4.76 4.76 4.75
O
1z
1H NMR (300 MHz, CDCl3)
0.95 0.99 3.99 0.97 1.00 1.02 1.02 1.99
f1 (ppm)

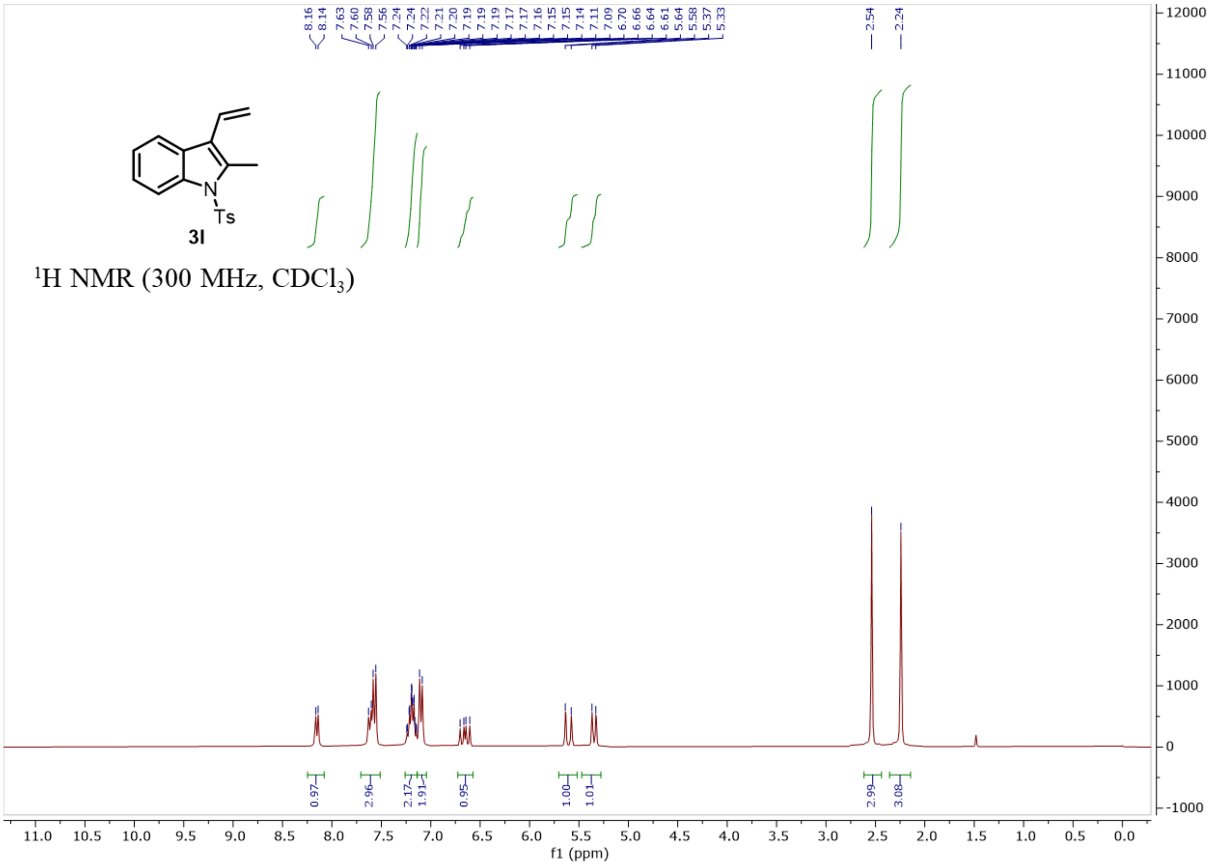

8.16
8.14
7.63
7.60
7.58
7.56
7.24
7.24
7.22
7.21
7.20
7.19
7.19
7.19
7.17
7.17
7.16
7.15
7.15
7.14
7.11
7.09
6.70
6.66
6.64
6.61
5.64
5.58
5.37
5.33
2.54
2.24
3l
$^{1}$H NMR (300 MHz, CDCl$_3$)
0.97
2.96
2.17
1.91
0.95
1.00
1.01
2.99
3.08
f1 (ppm)


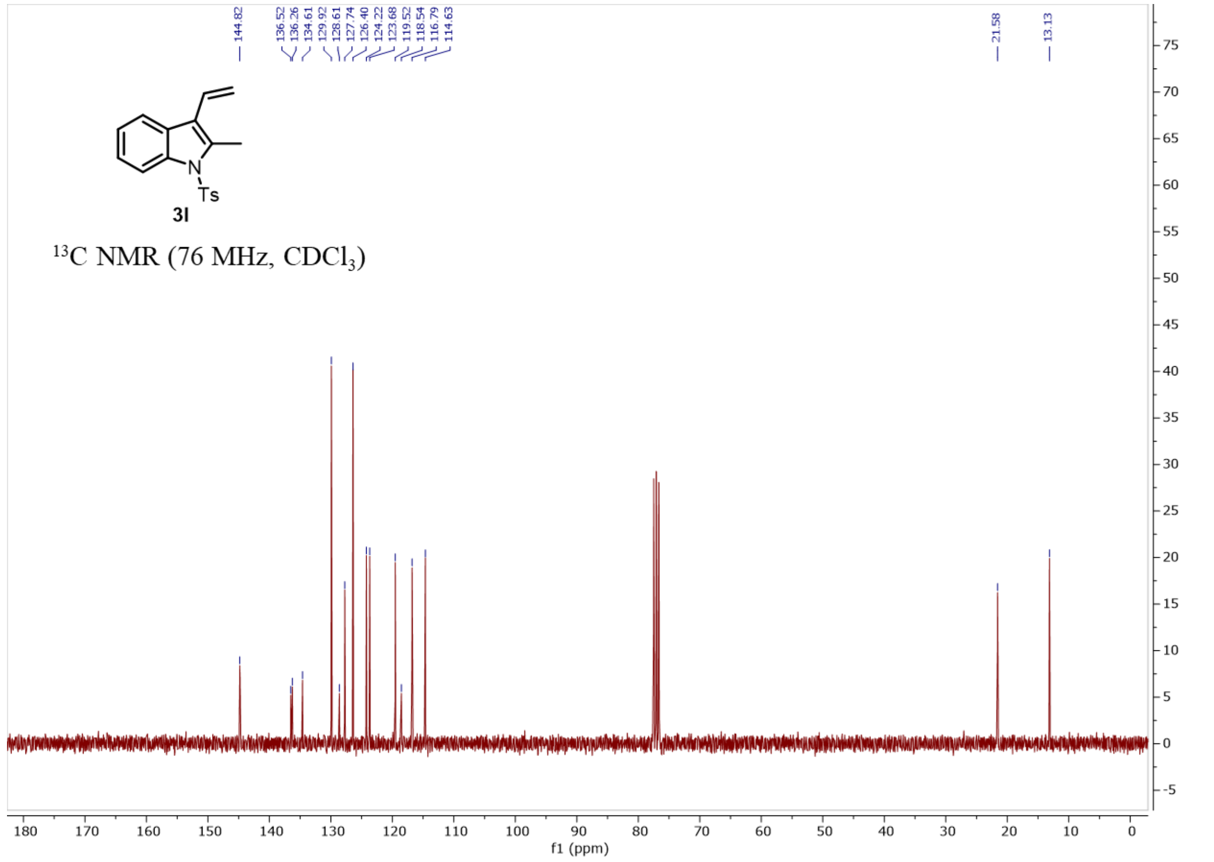

144.82
136.52
136.26
134.61
129.92
128.61
127.74
126.40
124.22
123.68
119.52
118.54
116.79
114.63
21.58
13.13
3l
$^{13}$C NMR (76 MHz, CDCl$_3$)
f1 (ppm)

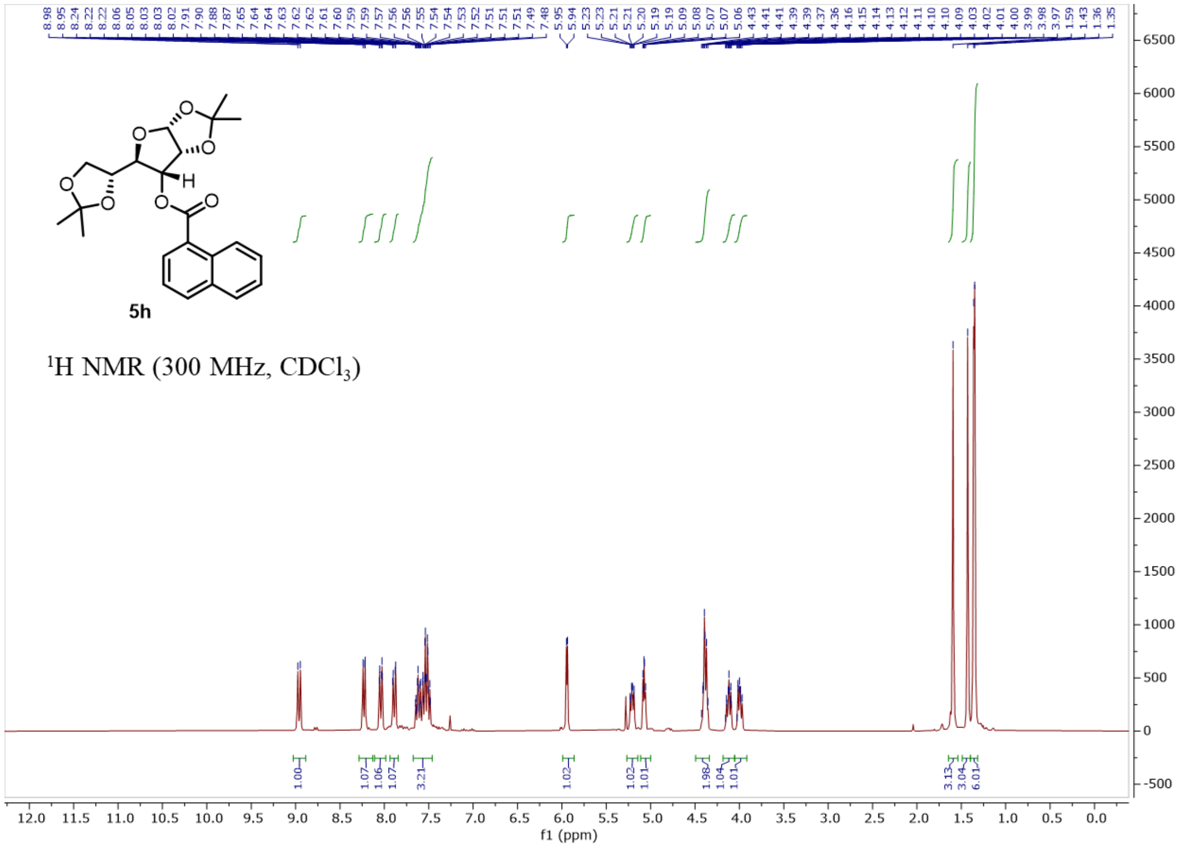
5h
1H NMR (300 MHz, CDCl3)
f1 (ppm)

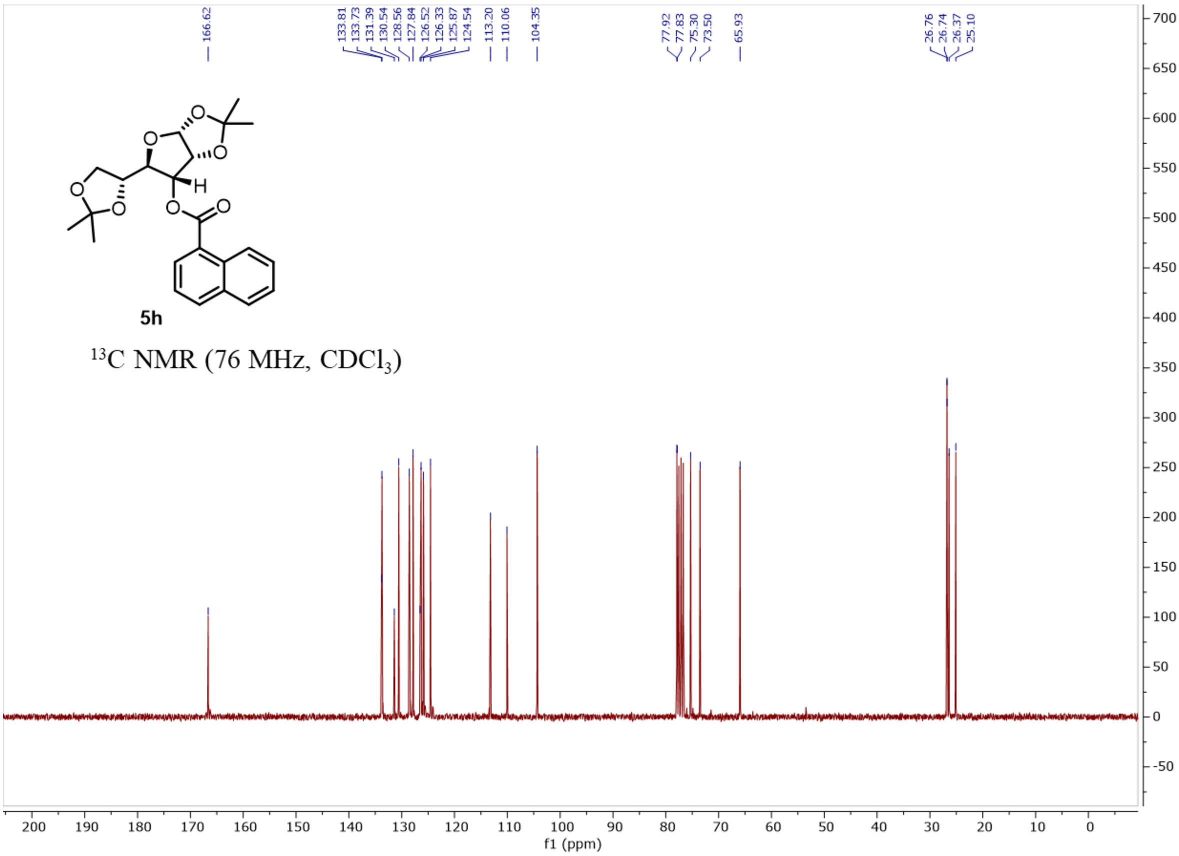
166.62
133.81
133.73
131.39
130.54
128.56
127.84
126.52
126.33
125.87
124.54
113.20
110.06
104.35
77.92
77.83
75.30
73.50
65.93
26.76
26.74
26.37
25.10
5h
13C NMR (76 MHz, CDCl3)
f1 (ppm)

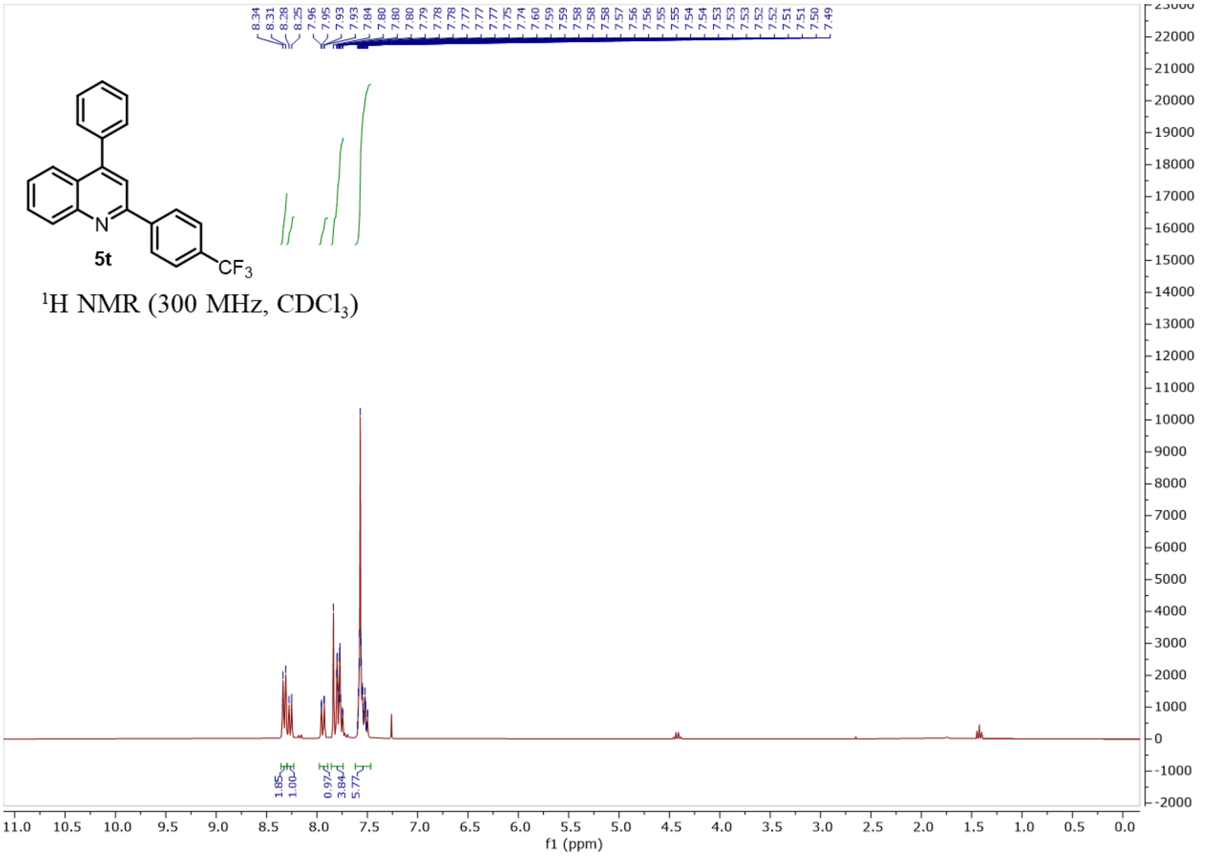
5t
¹H NMR (300 MHz, CDCl₃)
f1 (ppm)

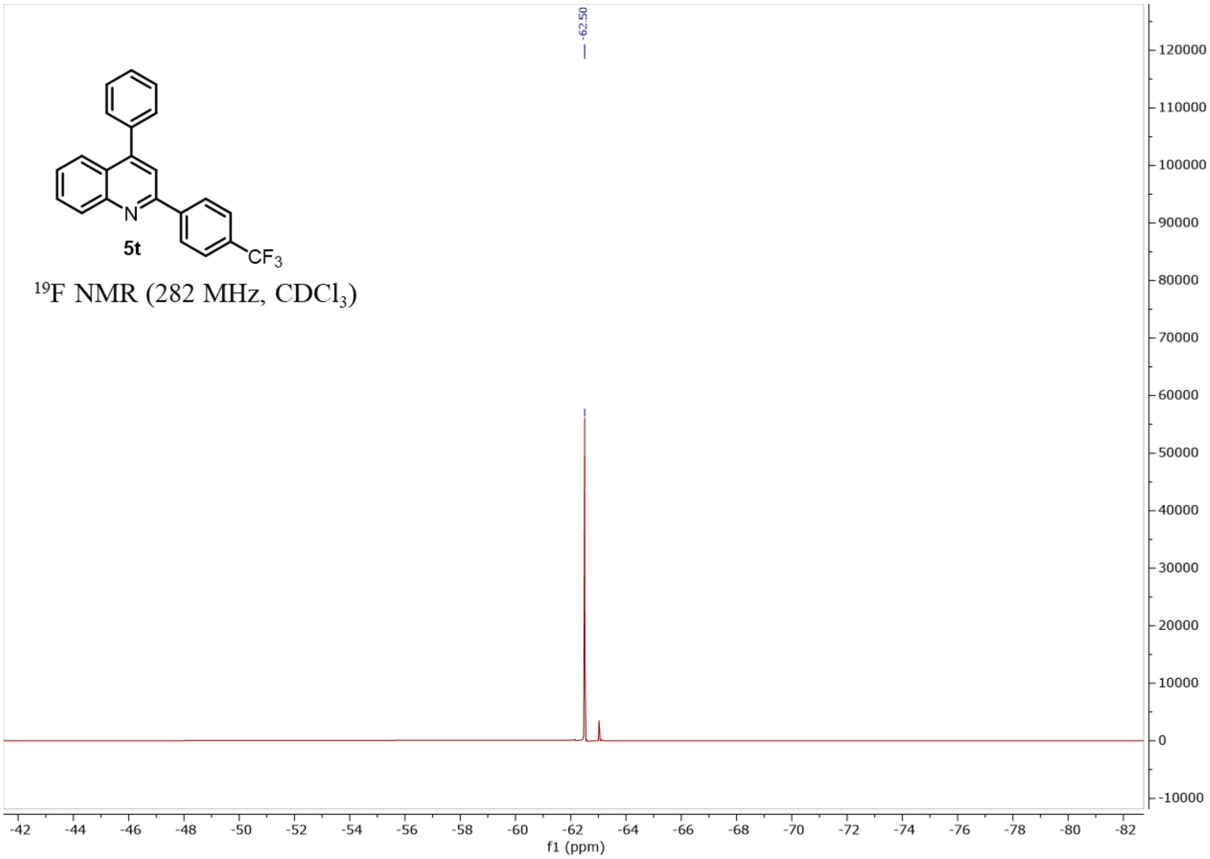
5t
¹⁹F NMR (282 MHz, CDCl₃)
-62.50
f1 (ppm)

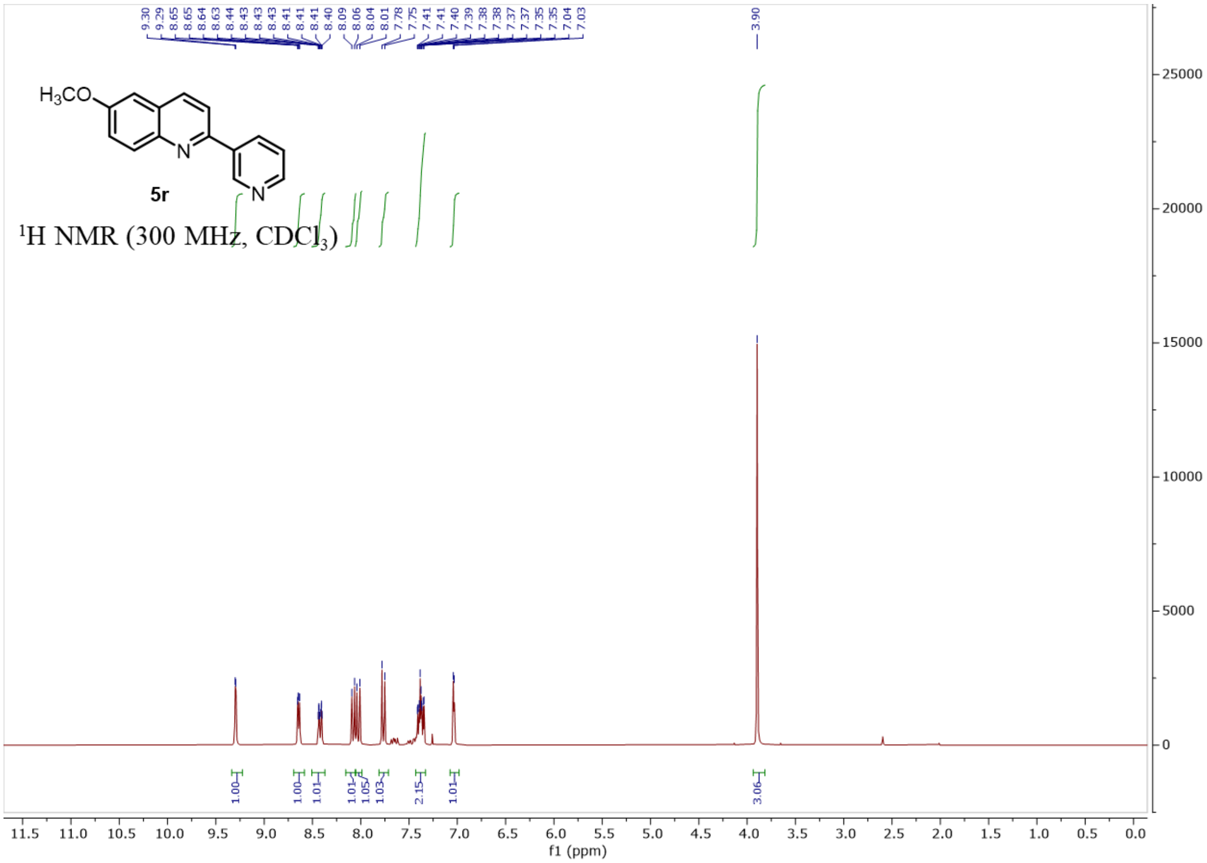
9.30
9.29
8.65
8.65
8.64
8.63
8.44
8.43
8.43
8.43
8.41
8.41
8.41
8.40
8.09
8.06
8.04
8.01
7.78
7.75
7.41
7.41
7.40
7.39
7.38
7.38
7.37
7.37
7.35
7.35
7.04
7.03
3.90
H3CO
N
N
5r
1H NMR (300 MHz, CDCl3)
1.00
1.00
1.01
1.01
1.05
1.03
2.15
1.01
3.06
f1 (ppm)

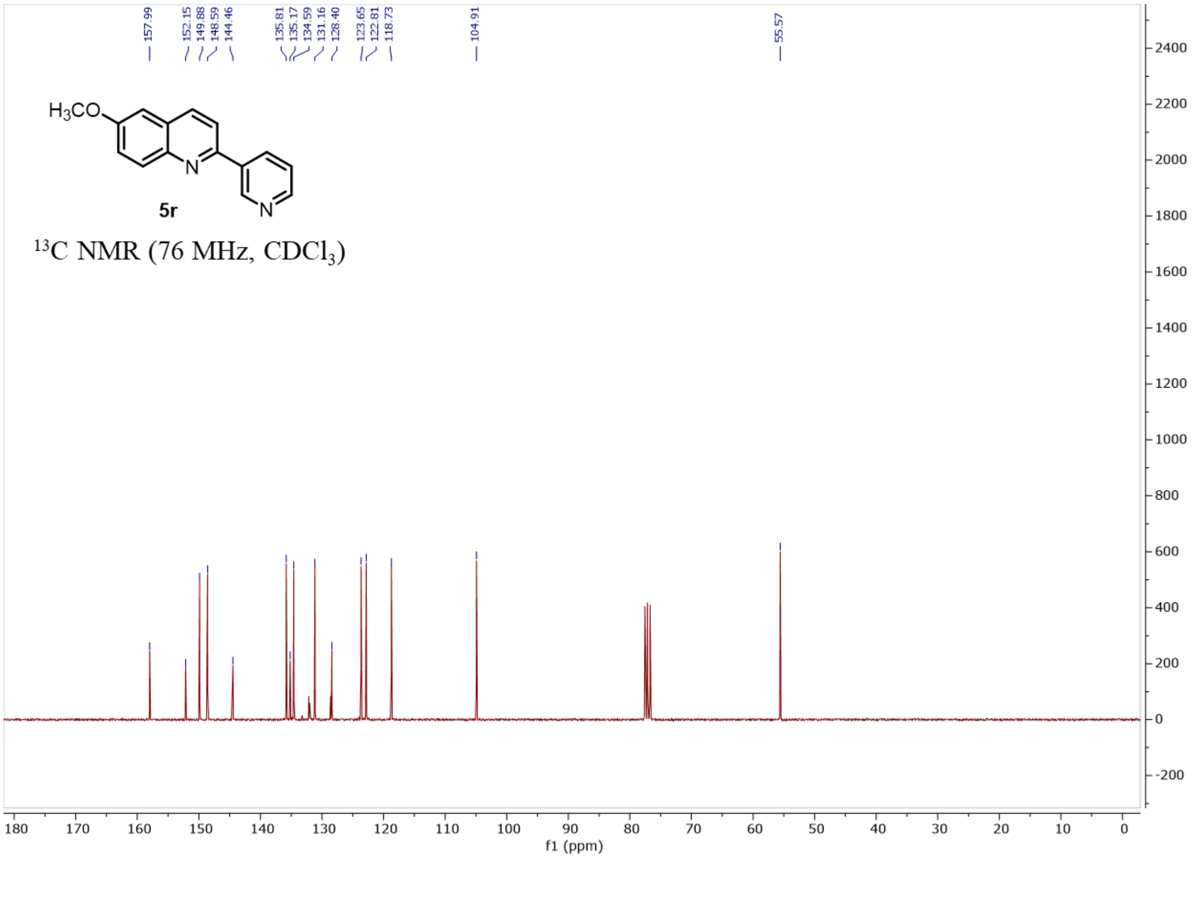
157.99
152.15
149.88
148.59
144.46
135.81
135.17
134.59
131.16
128.40
123.65
122.81
118.73
104.91
55.57
H3CO
N
N
5r
13C NMR (76 MHz, CDCl3)
f1 (ppm)

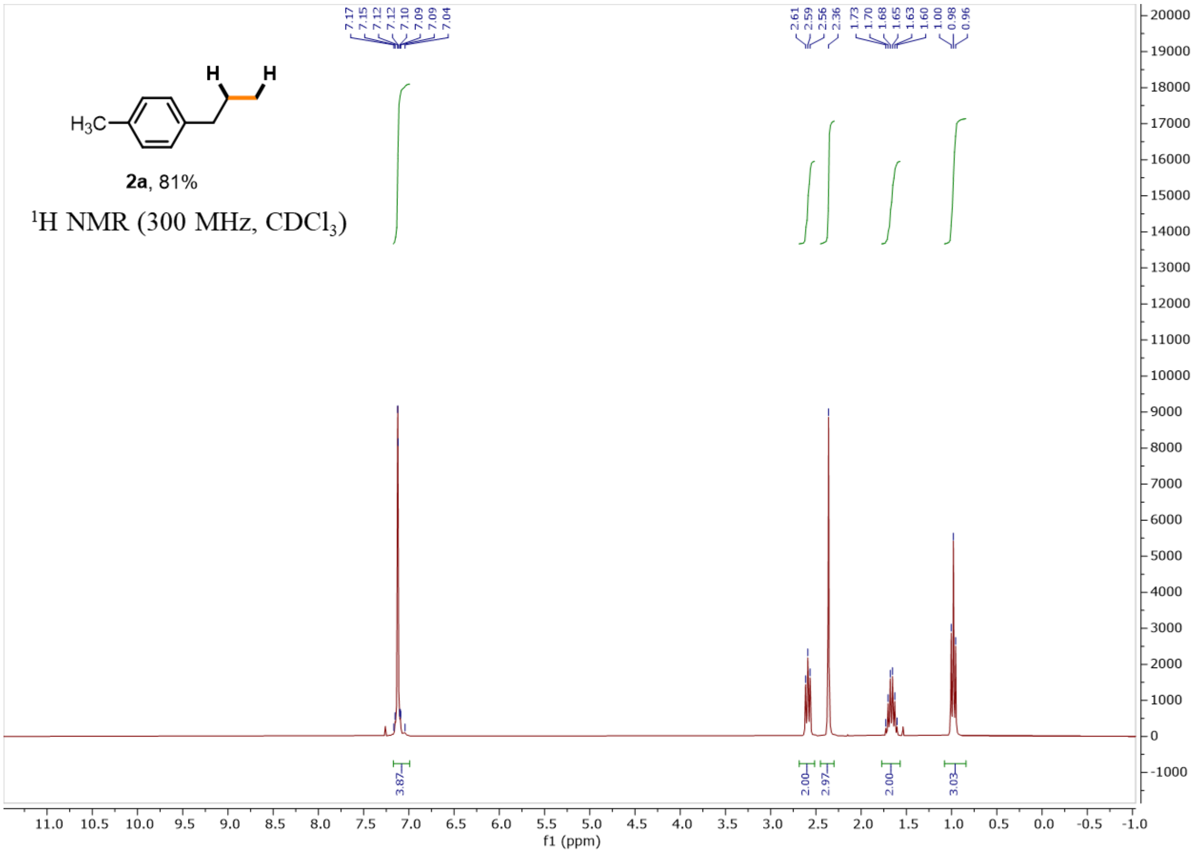
7.17 7.15 7.12 7.12 7.10 7.09 7.09 7.04
2.61 2.59 2.56 2.36 1.73 1.70 1.68 1.65 1.63 1.60 1.00 0.98 0.96
H H
$H_3C$
2a, 81%
$^1$H NMR (300 MHz, $CDCl_3$)
3.87
2.00
2.97
2.00
3.03
f1 (ppm)

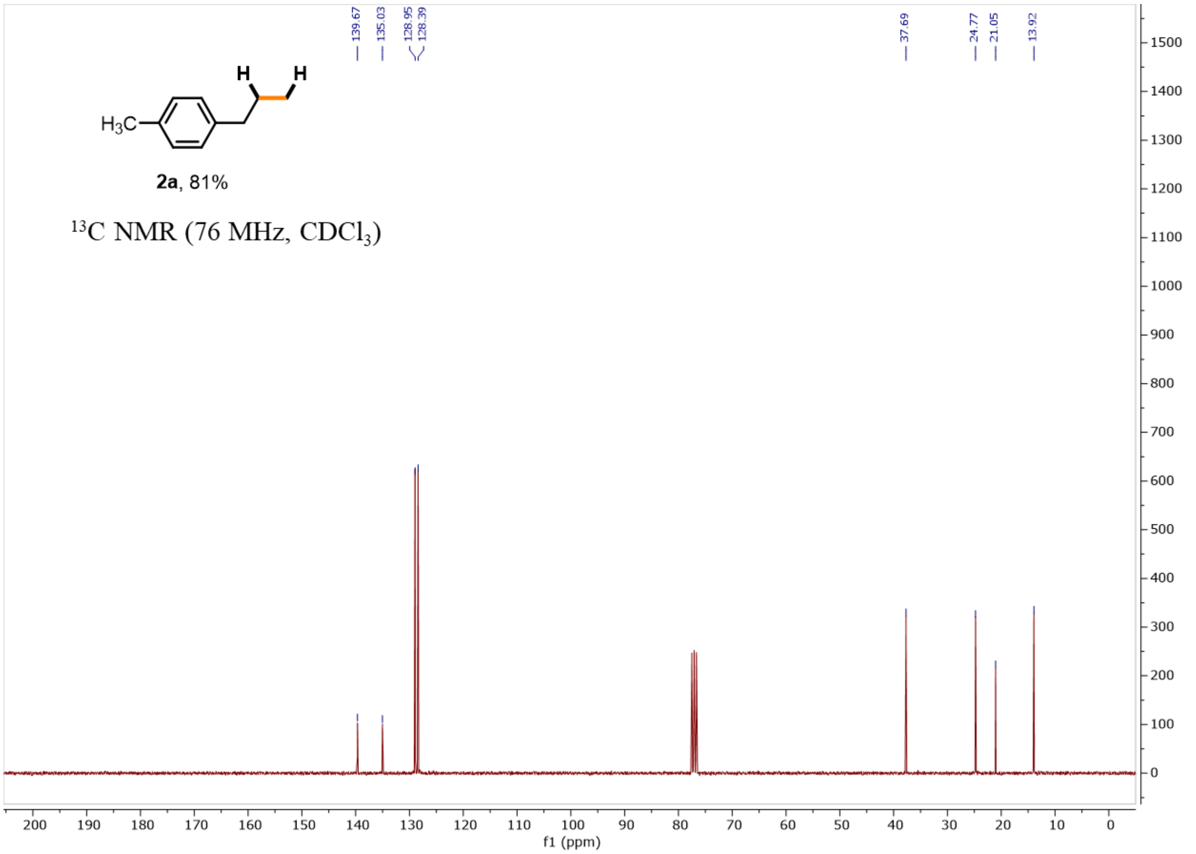
139.67
135.03
128.95
128.39
37.69
24.77
21.05
13.92
H H
$H_3C$
2a, 81%
$^{13}$C NMR (76 MHz, $CDCl_3$)
f1 (ppm)

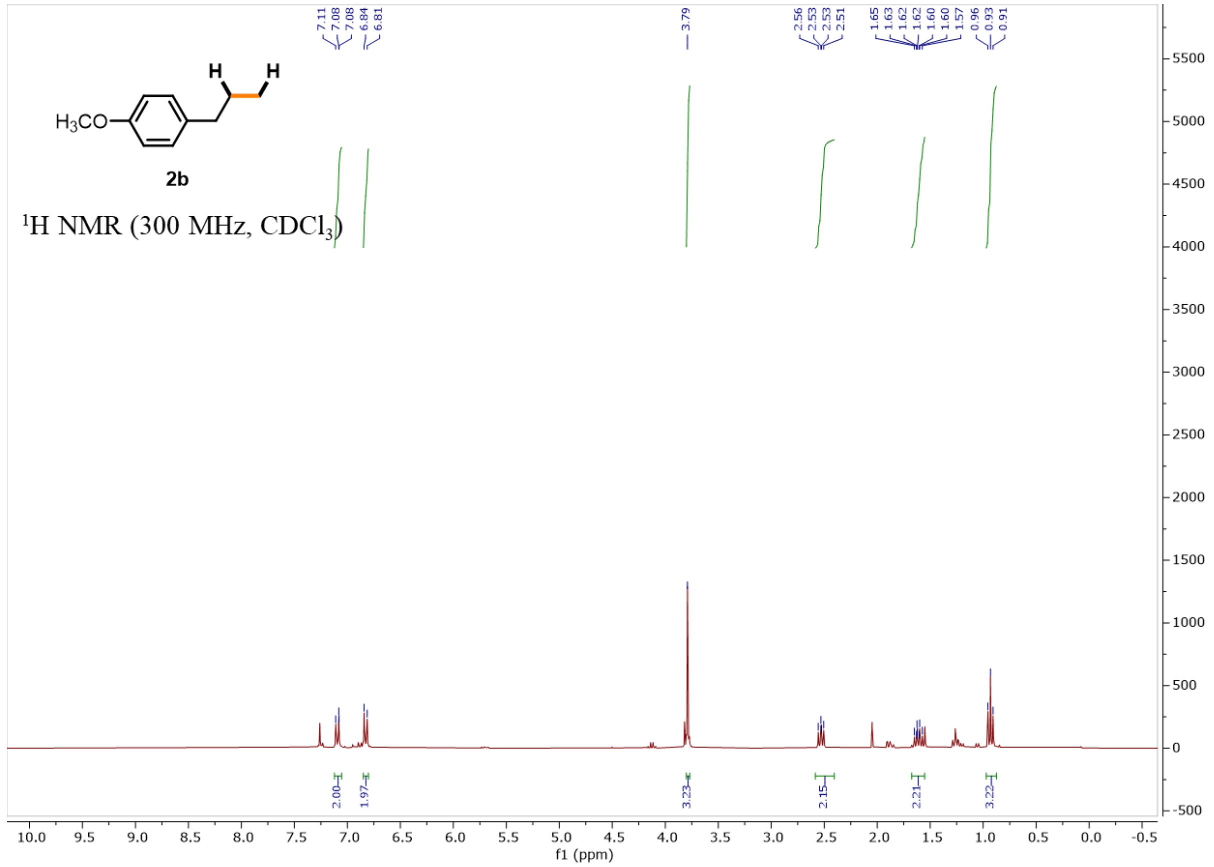
7.11
7.08
7.08
6.84
6.81
3.79
2.56
2.53
2.53
2.51
1.65
1.63
1.62
1.62
1.60
1.60
1.57
0.96
0.93
0.91
H3CO
2b
$^{1}$H NMR (300 MHz, CDCl$_3$)
2.00
1.97
3.23
2.15
2.21
3.22
f1 (ppm)

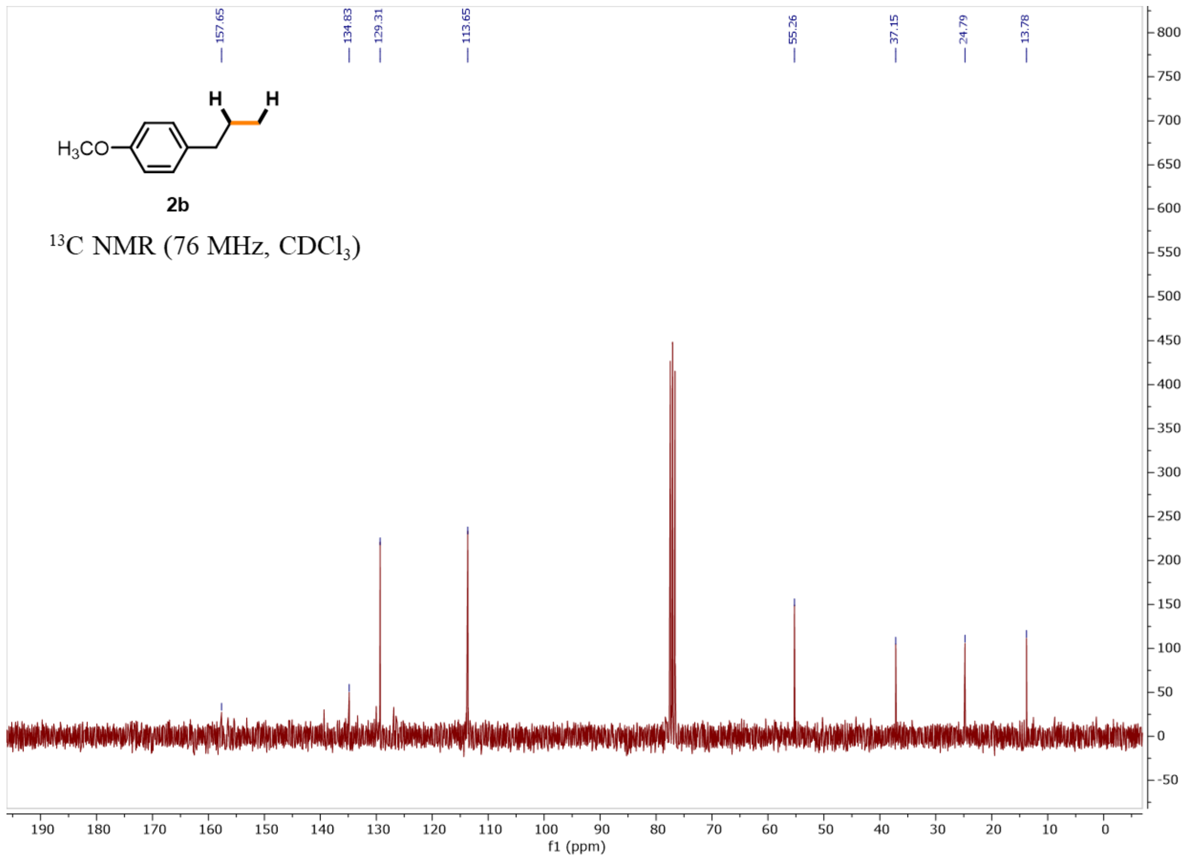
157.65
134.83
129.31
113.65
55.26
37.15
24.79
13.78
H3CO
2b
$^{13}$C NMR (76 MHz, CDCl$_3$)
f1 (ppm)

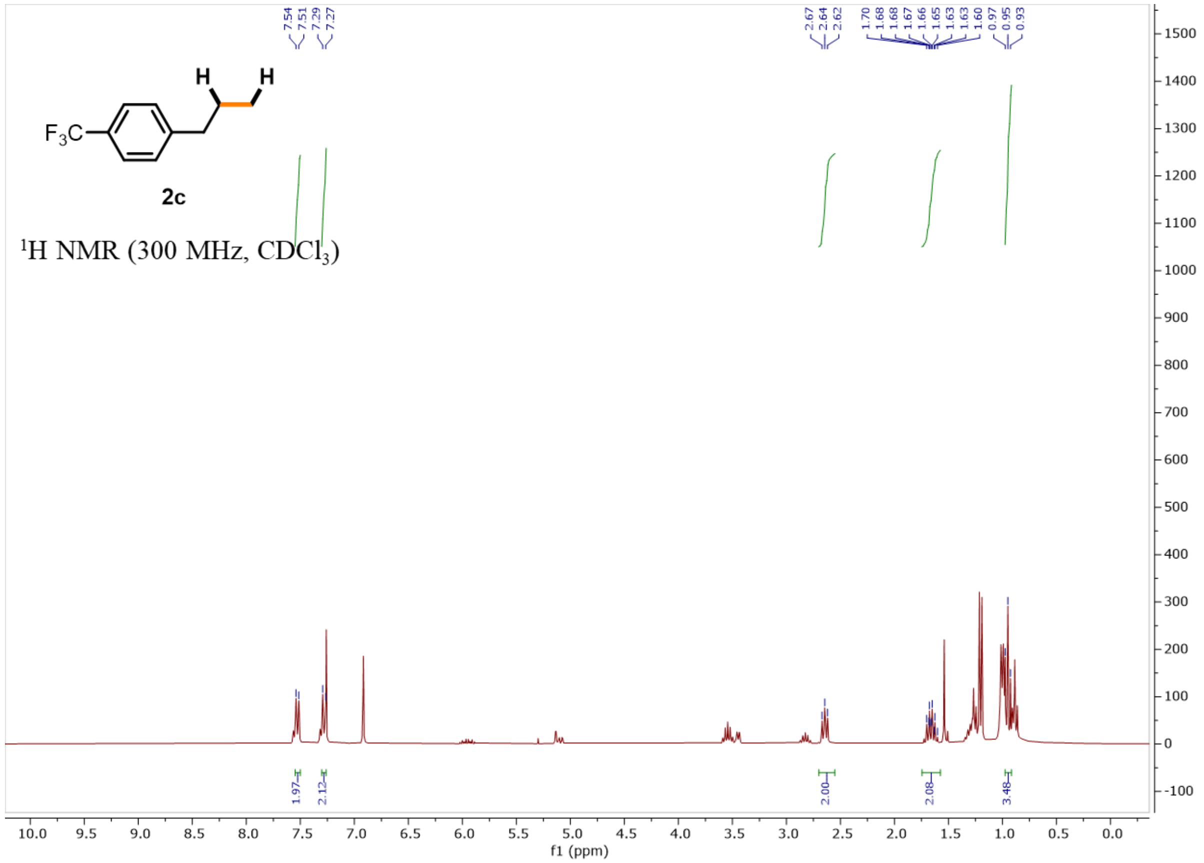

F3C
H
H
2c
1H NMR (300 MHz, CDCl3)
7.54
7.51
7.29
7.27
2.67
2.64
2.62
1.70
1.68
1.68
1.67
1.66
1.65
1.63
1.63
1.60
0.97
0.95
0.93
1.97
2.12
2.00
2.08
3.48
f1 (ppm)

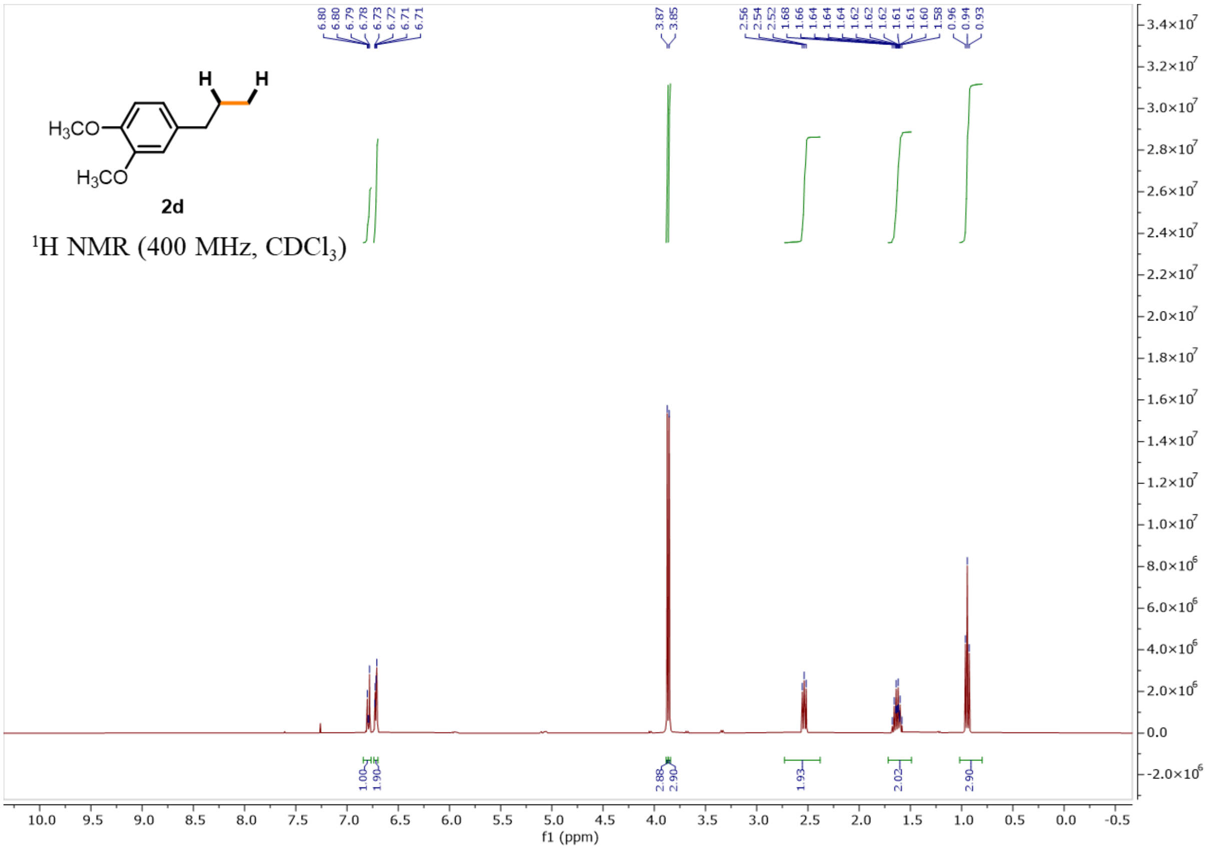

2d
$^{1}$H NMR (400 MHz, $CDCl_3$)
f1 (ppm)


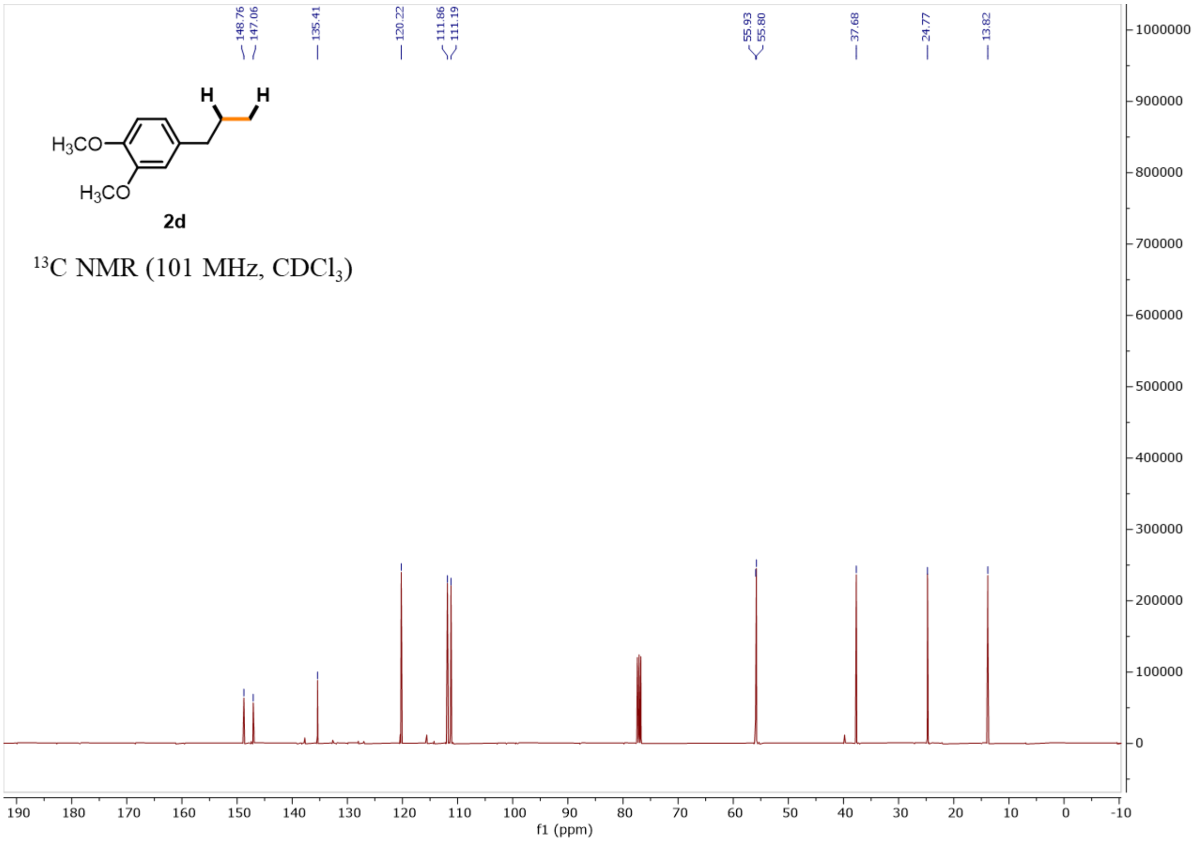

2d
$^{13}$C NMR (101 MHz, $CDCl_3$)
f1 (ppm)

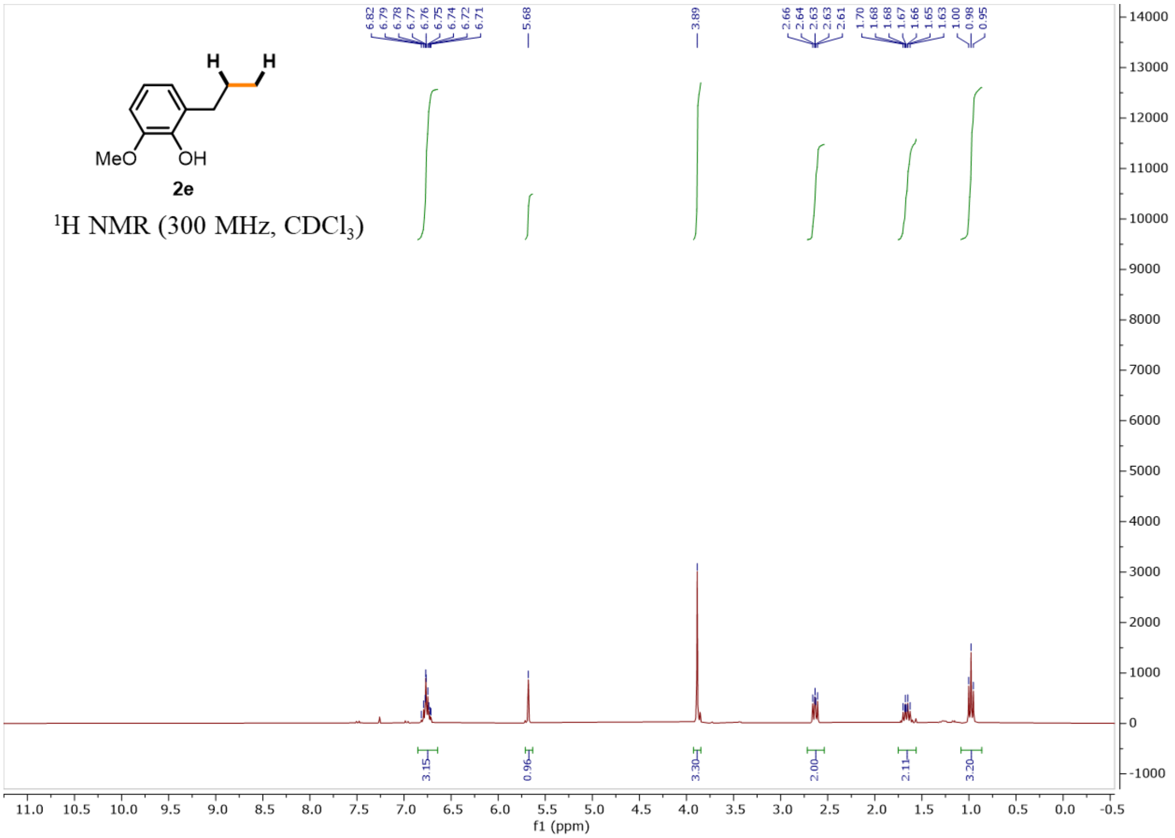

2e
$^{1}$H NMR (300 MHz, CDCl$_3$)
6.82 6.79 6.78 6.77 6.76 6.75 6.74 6.72 6.71
5.68
3.89
2.66 2.64 2.63 2.63 2.61
1.70 1.68 1.68 1.67 1.66 1.65 1.63
1.00 0.98 0.95
3.15 0.96 3.30 2.00 2.11 3.20
f1 (ppm)


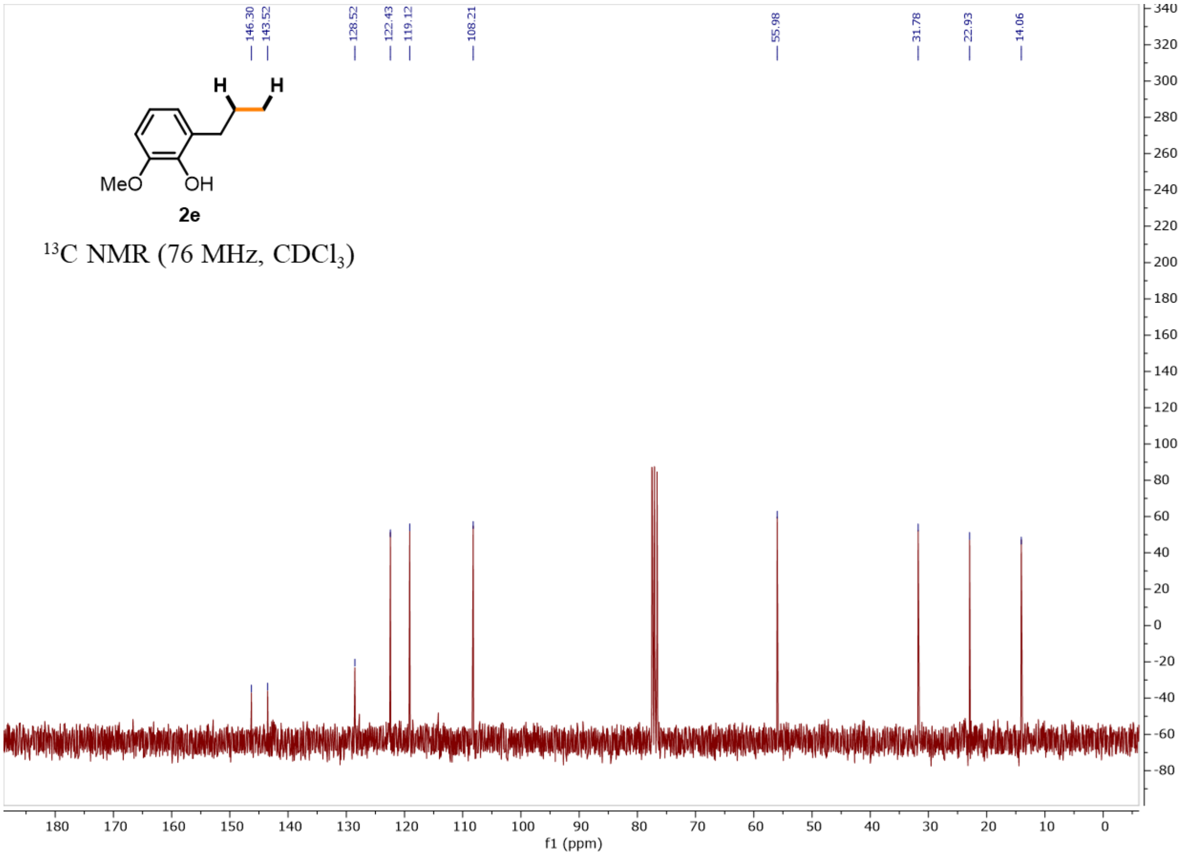

2e
$^{13}$C NMR (76 MHz, CDCl$_3$)
146.30 143.52 128.52 122.43 119.12 108.21 55.98 31.78 22.93 14.06
f1 (ppm)

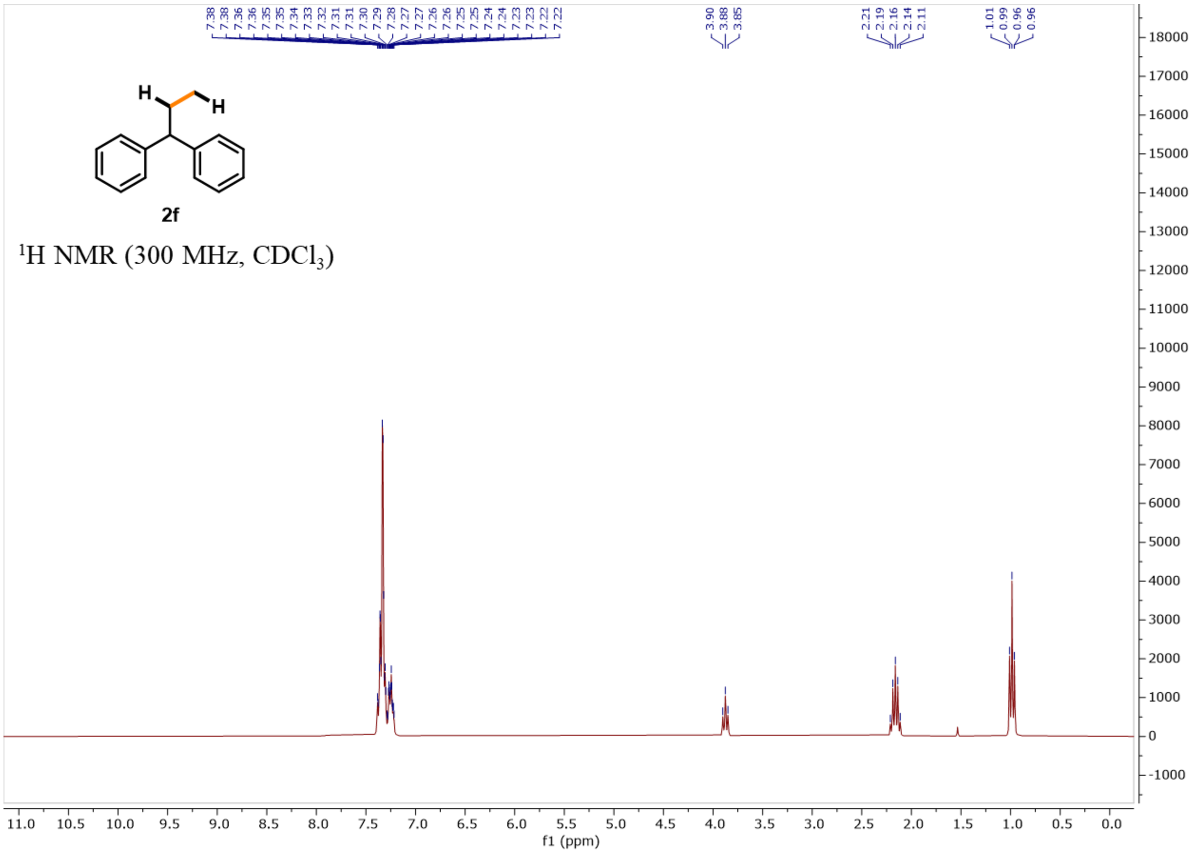


$^{1}H$ NMR (300 MHz, $CDCl_3$)

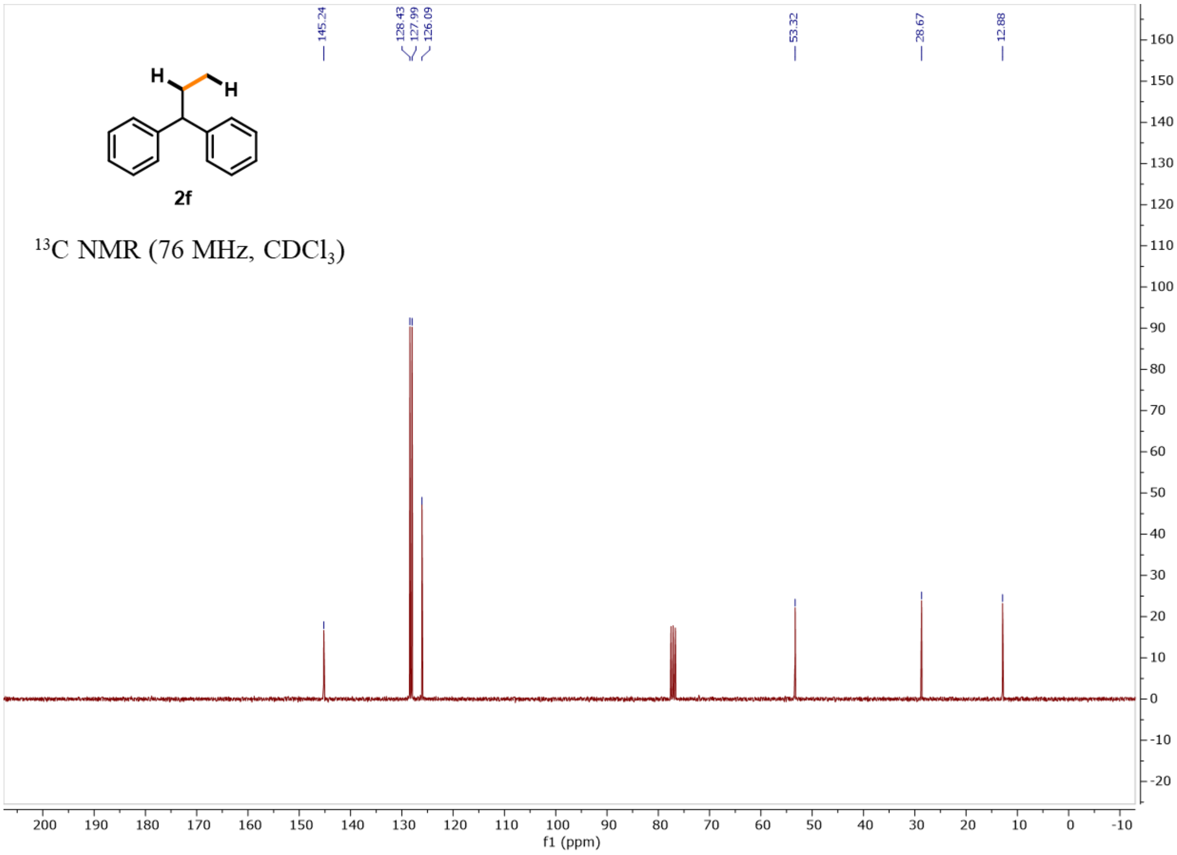


$^{13}C$ NMR (76 MHz, $CDCl_3$)

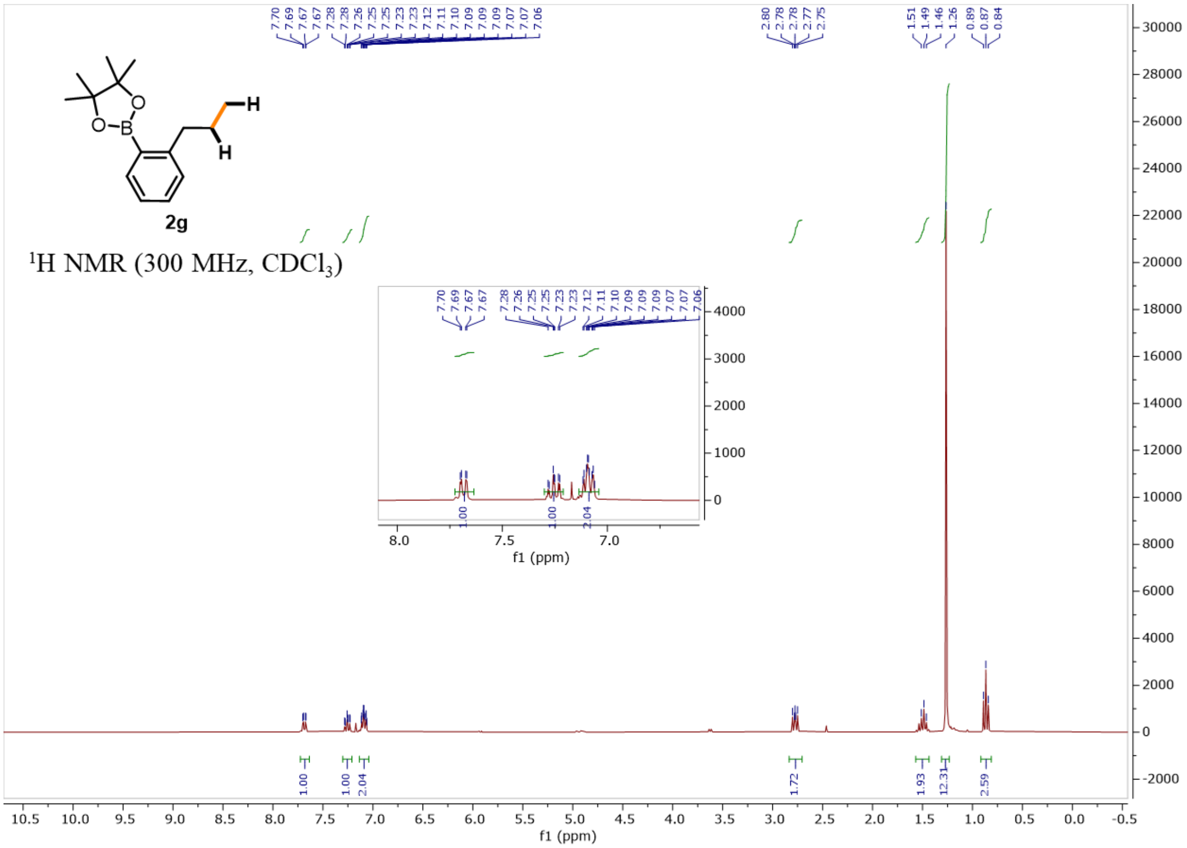
2g
1H NMR (300 MHz, CDCl3)
7.70 7.69 7.67 7.67 7.28 7.26 7.25 7.25 7.23 7.23 7.12 7.11 7.10 7.09 7.09 7.09 7.07 7.07 7.06
2.80 2.78 2.78 2.77 2.75
1.51 1.49 1.46 1.26
0.89 0.87 0.84
1.00 1.00 2.04 1.72 1.93 12.31 2.59
f1 (ppm)

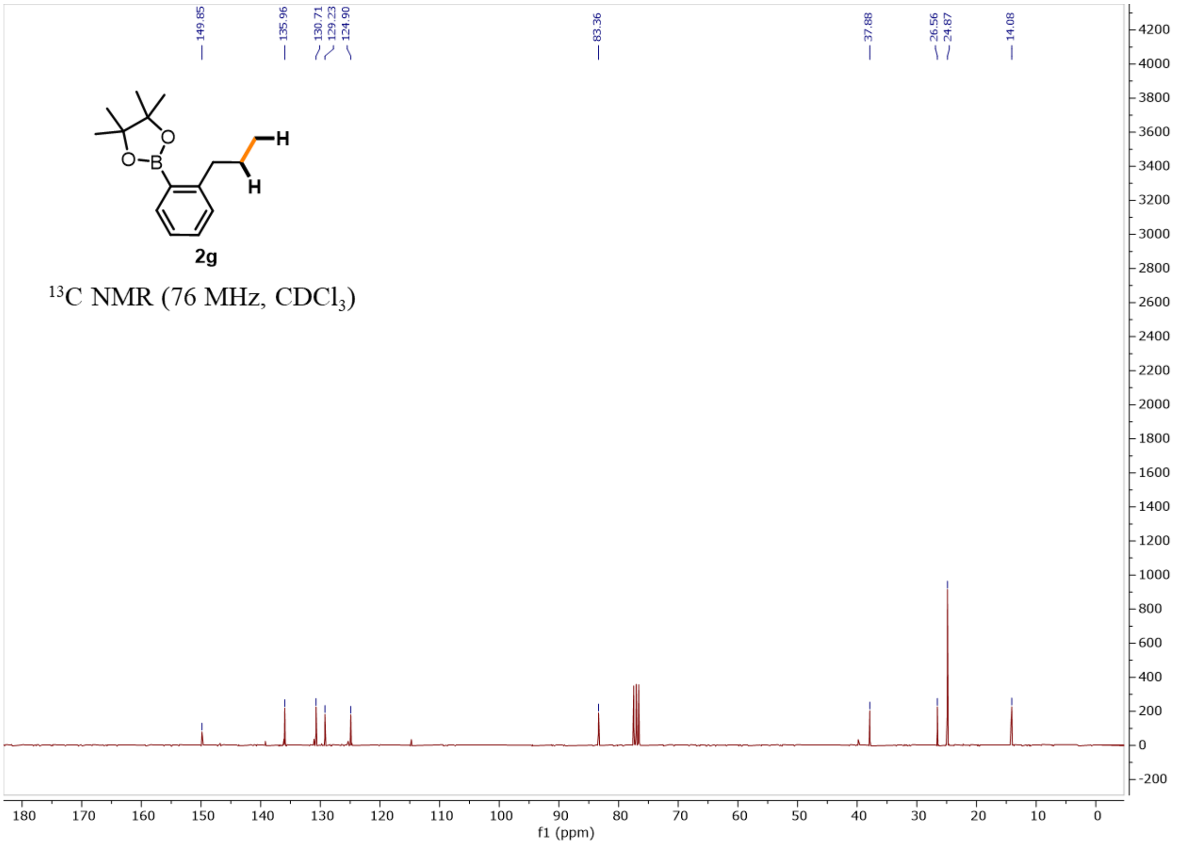
2g
13C NMR (76 MHz, CDCl3)
149.85
135.96
130.71 129.23 124.90
83.36
37.88
26.56 24.87
14.08
f1 (ppm)

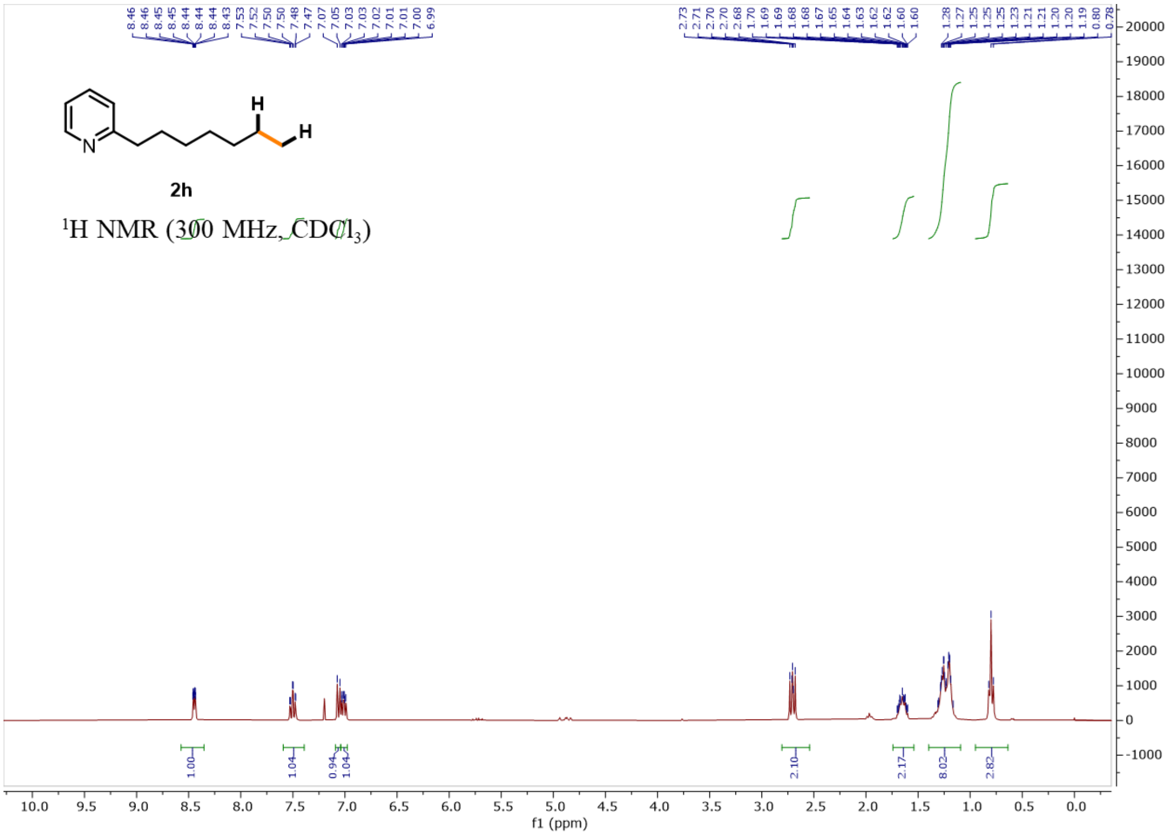

2h
$^1$H NMR (300 MHz, CDCl$_3$)
f1 (ppm)


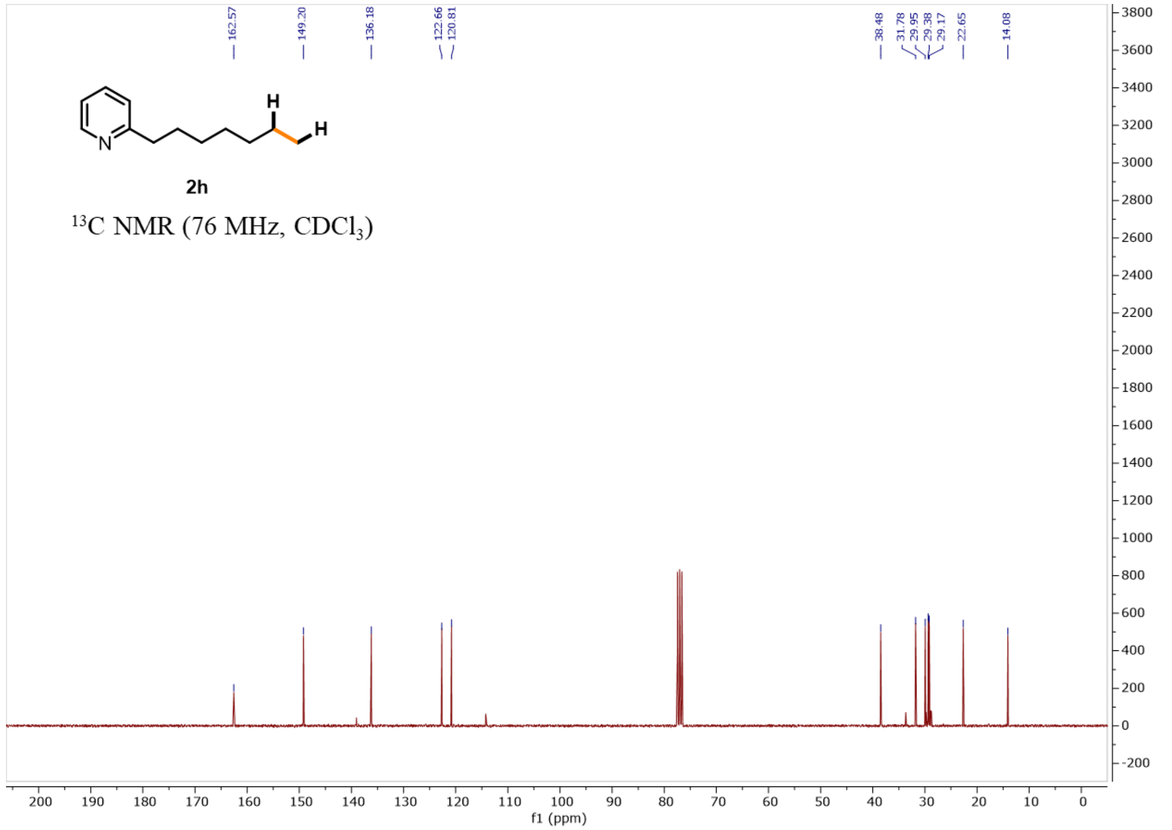

2h
$^{13}$C NMR (76 MHz, CDCl$_3$)
162.57
149.20
136.18
122.66
120.81
38.48
31.78
29.95
29.38
29.17
22.65
14.08
f1 (ppm)

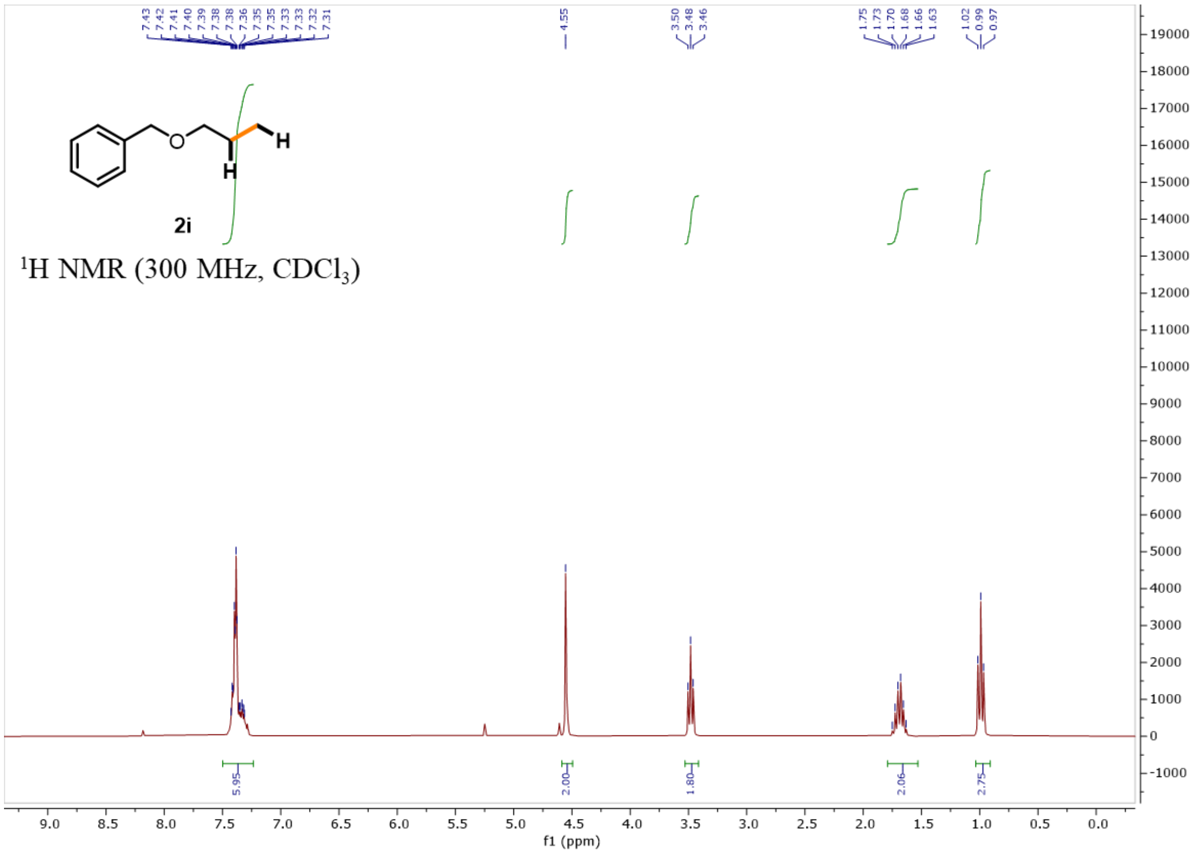
2i
1H NMR (300 MHz, CDCl3)
7.43 7.42 7.41 7.40 7.39 7.38 7.38 7.36 7.35 7.35 7.33 7.33 7.32 7.31
4.55
3.50 3.48 3.46
1.75 1.73 1.70 1.68 1.66 1.63
1.02 0.99 0.97
5.95
2.00
1.80
2.06
2.75
f1 (ppm)

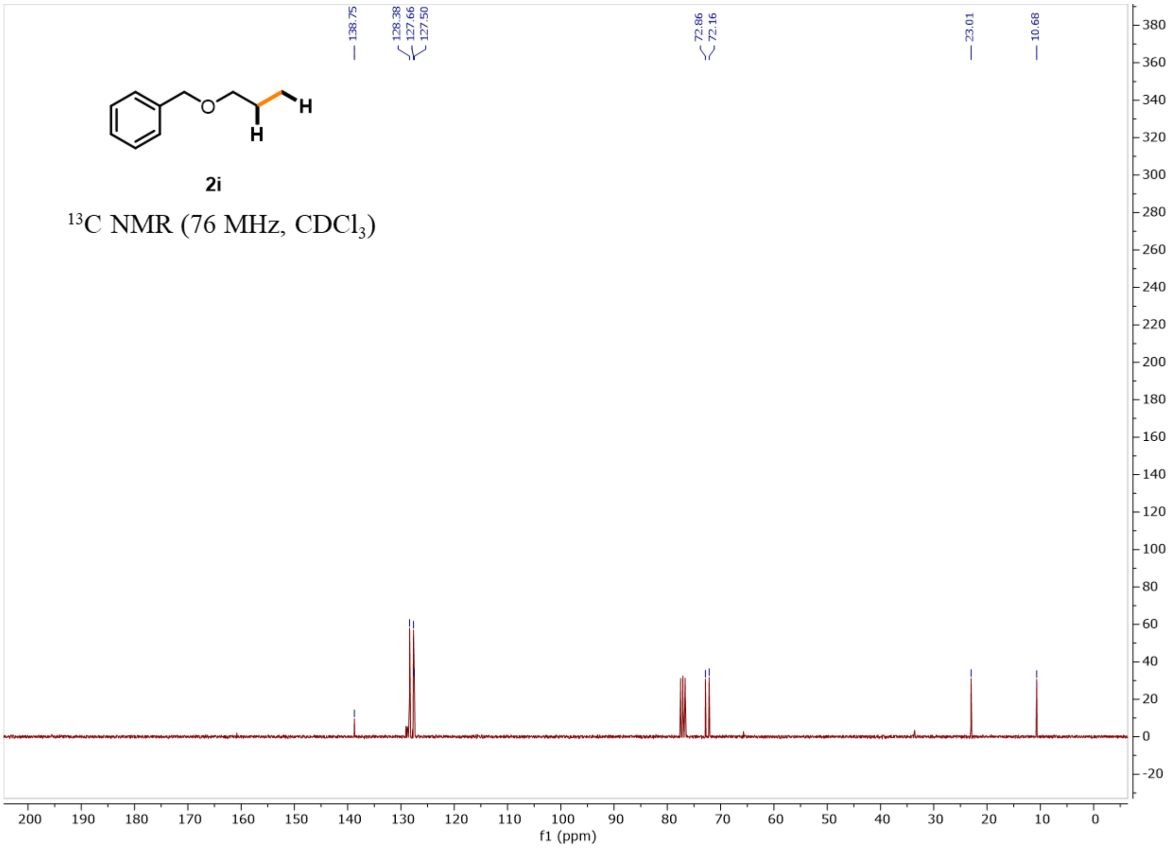
2i
13C NMR (76 MHz, CDCl3)
138.75
128.38 127.66 127.50
72.86 72.16
23.01
10.68
f1 (ppm)

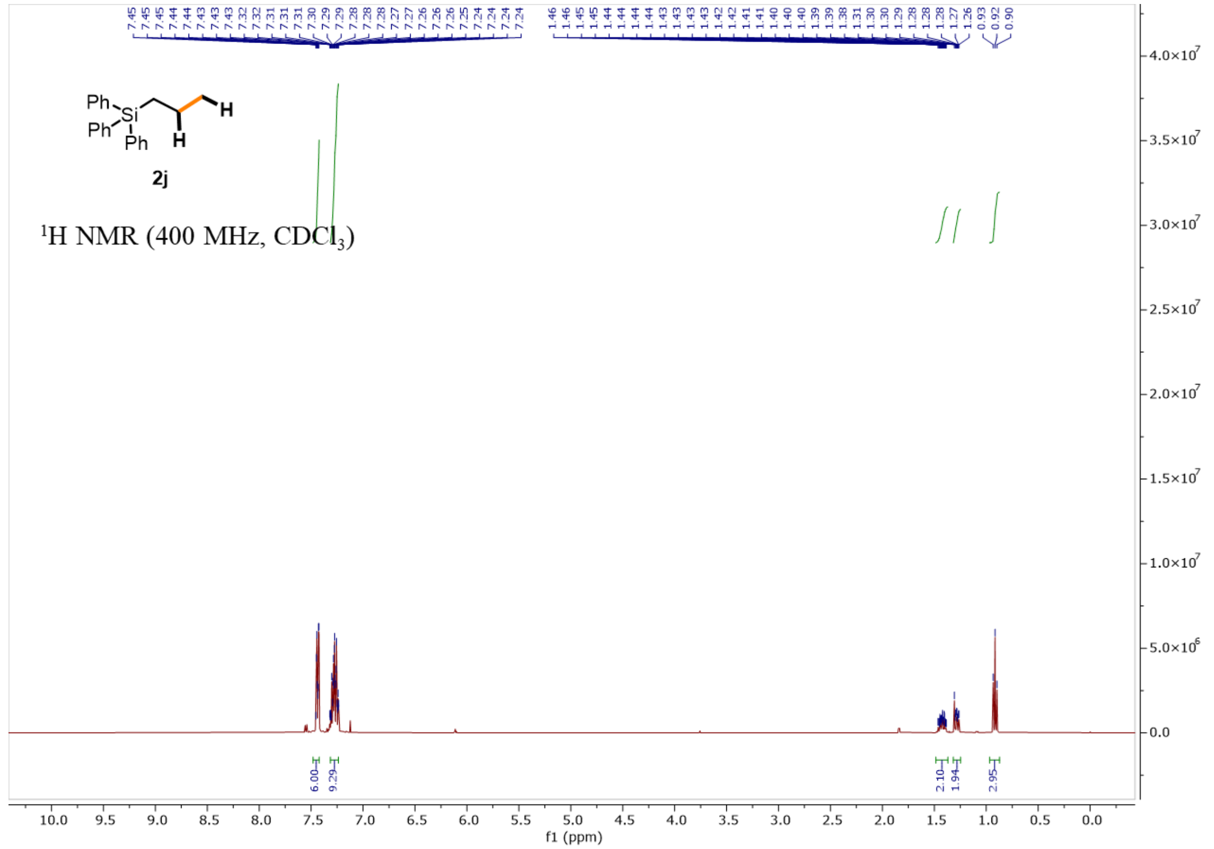

2j
$^{1}H$ NMR (400 MHz, $CDCl_3$)


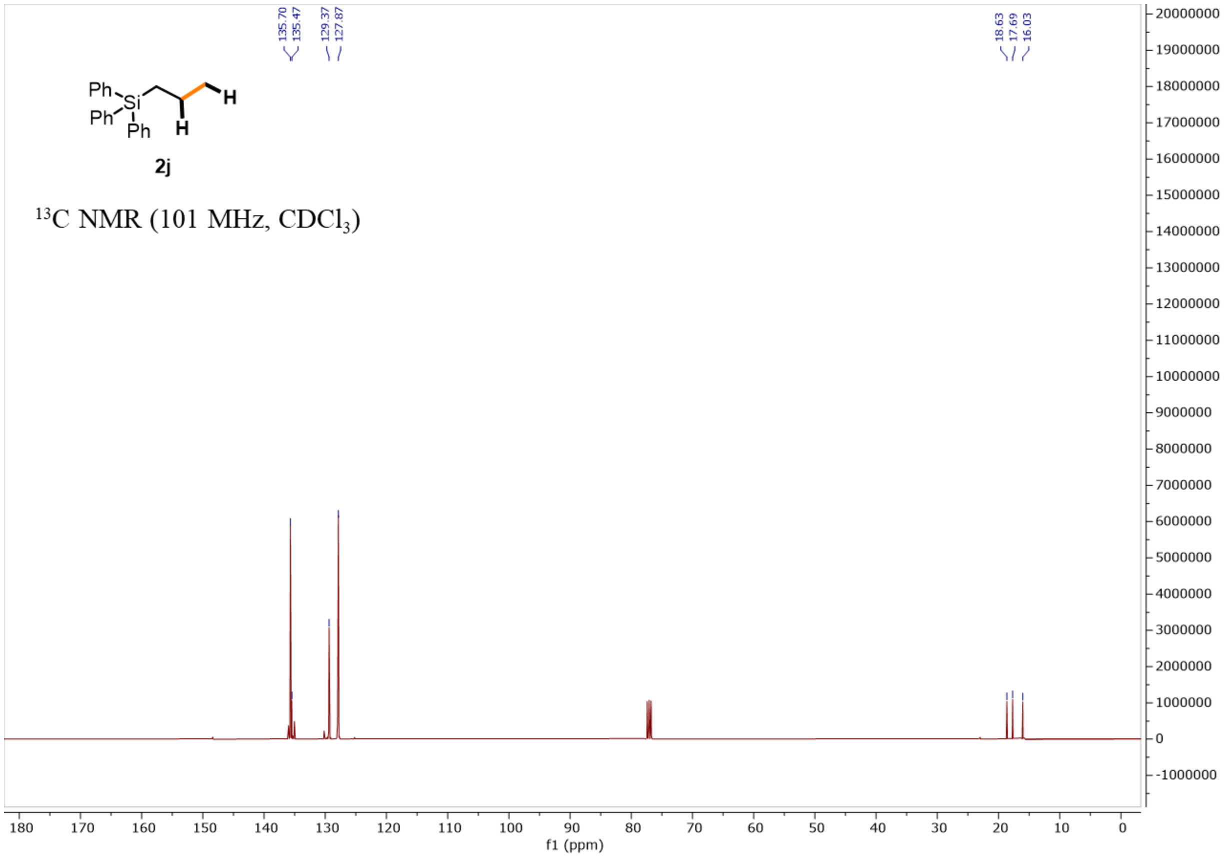

2j
$^{13}C$ NMR (101 MHz, $CDCl_3$)

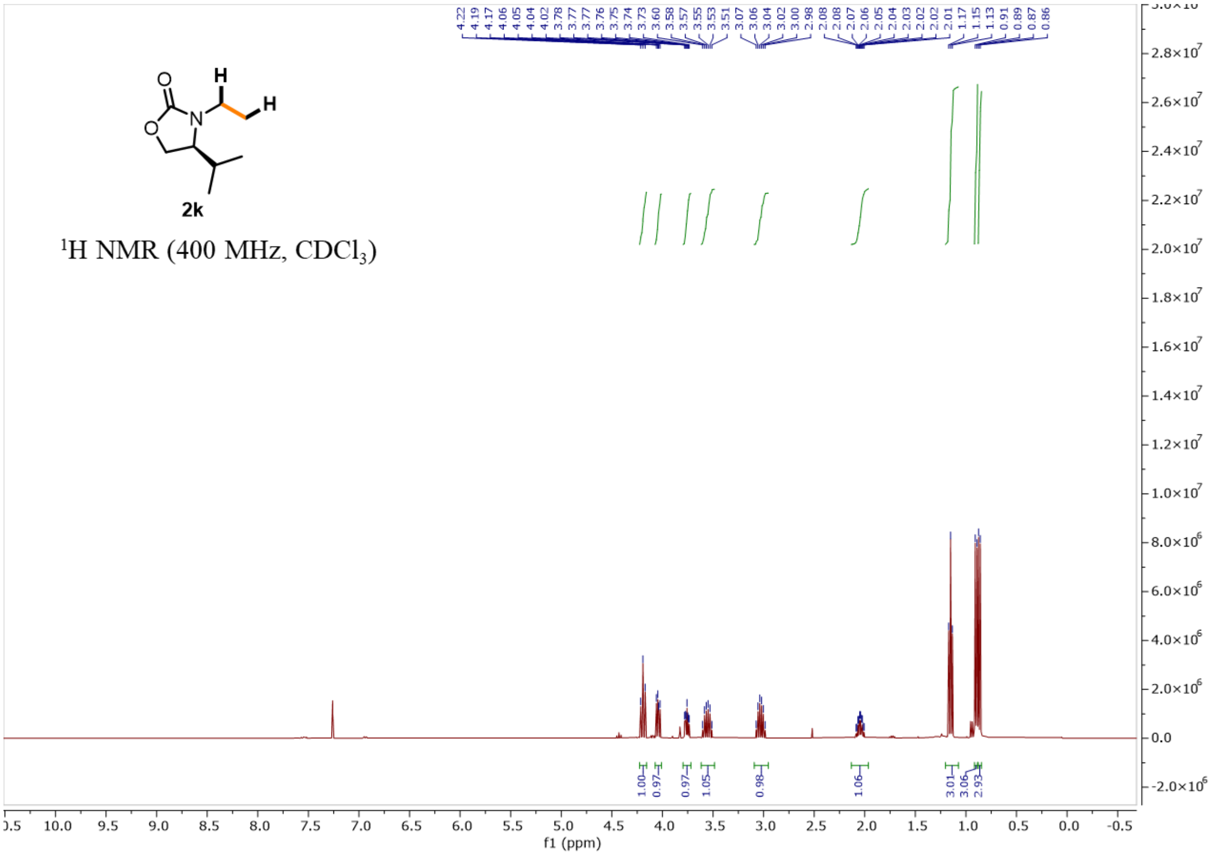
2k
1H NMR (400 MHz, CDCl3)
4.22 4.19 4.17 4.06 4.05 4.04 4.02 3.78 3.77 3.77 3.76 3.75 3.74 3.73 3.60 3.58 3.57 3.55 3.53 3.51 3.07 3.06 3.04 3.02 3.00 2.98 2.08 2.08 2.07 2.06 2.05 2.05 2.04 2.03 2.02 2.02 2.01 1.17 1.15 1.13 0.91 0.89 0.87 0.86
1.00 0.97 0.97 1.05 0.98 1.06 3.01 3.06 2.93
f1 (ppm)

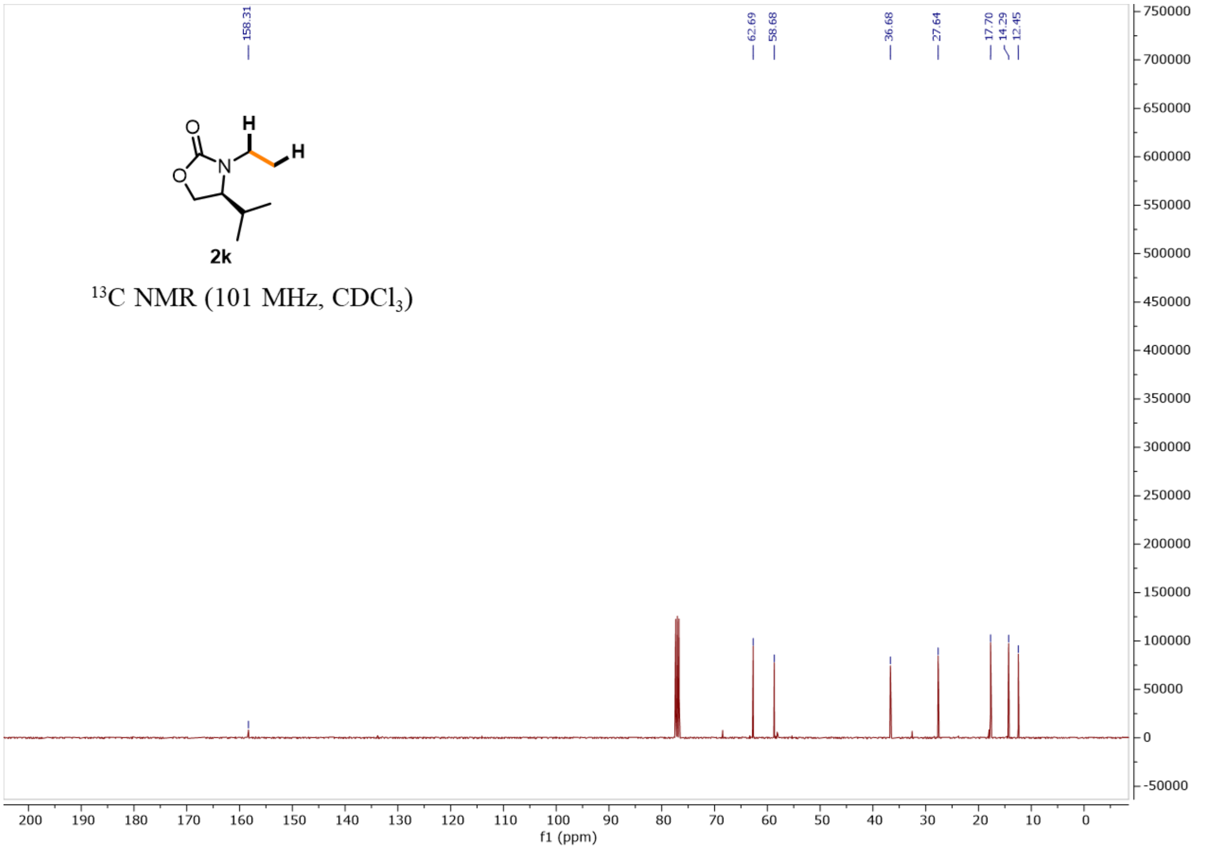
2k
13C NMR (101 MHz, CDCl3)
158.31
62.69
58.68
36.68
27.64
17.70
14.29
12.45
f1 (ppm)

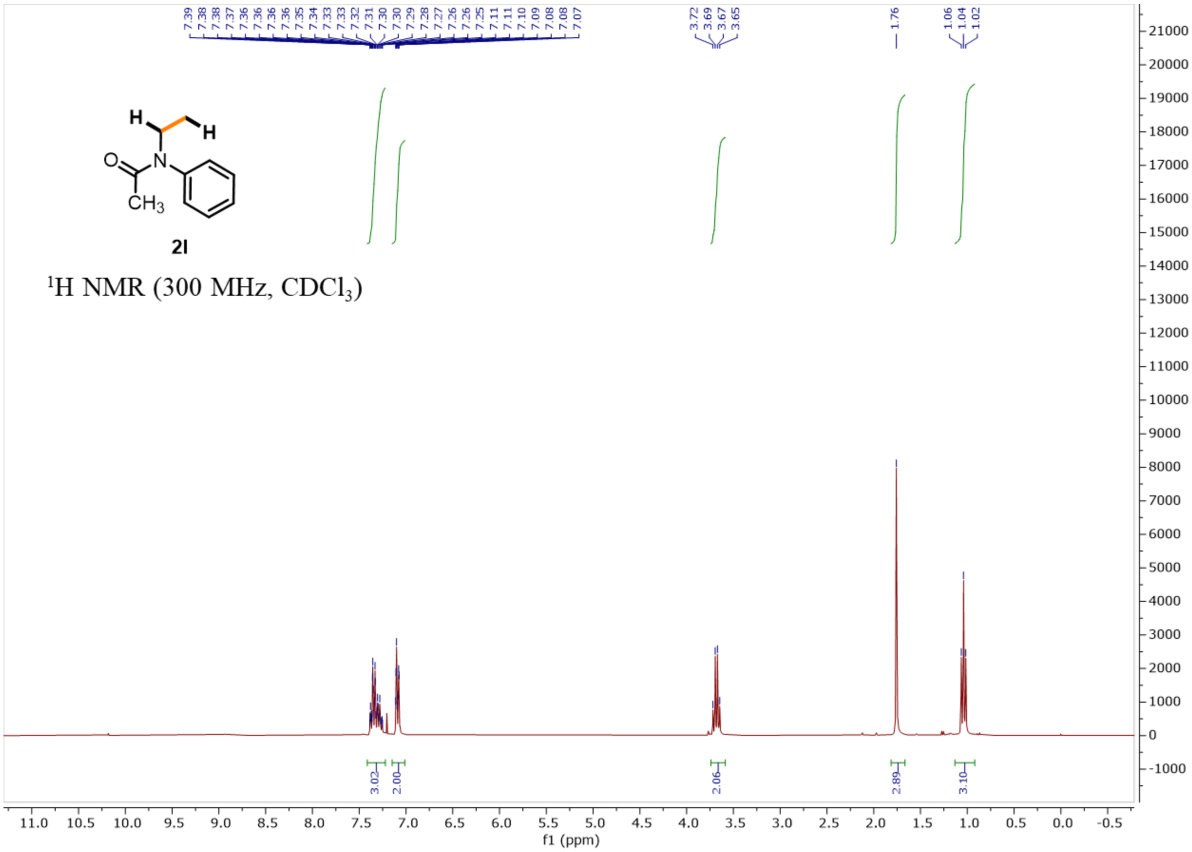
2l
1H NMR (300 MHz, CDCl3)
7.39 7.38 7.38 7.37 7.36 7.36 7.36 7.36 7.35 7.34 7.33 7.33 7.32 7.31 7.30 7.30 7.29 7.28 7.27 7.26 7.26 7.25 7.11 7.11 7.10 7.09 7.08 7.08 7.07
3.72 3.69 3.67 3.65
1.76
1.06 1.04 1.02
3.02 2.00 2.06 2.89 3.10
f1 (ppm)

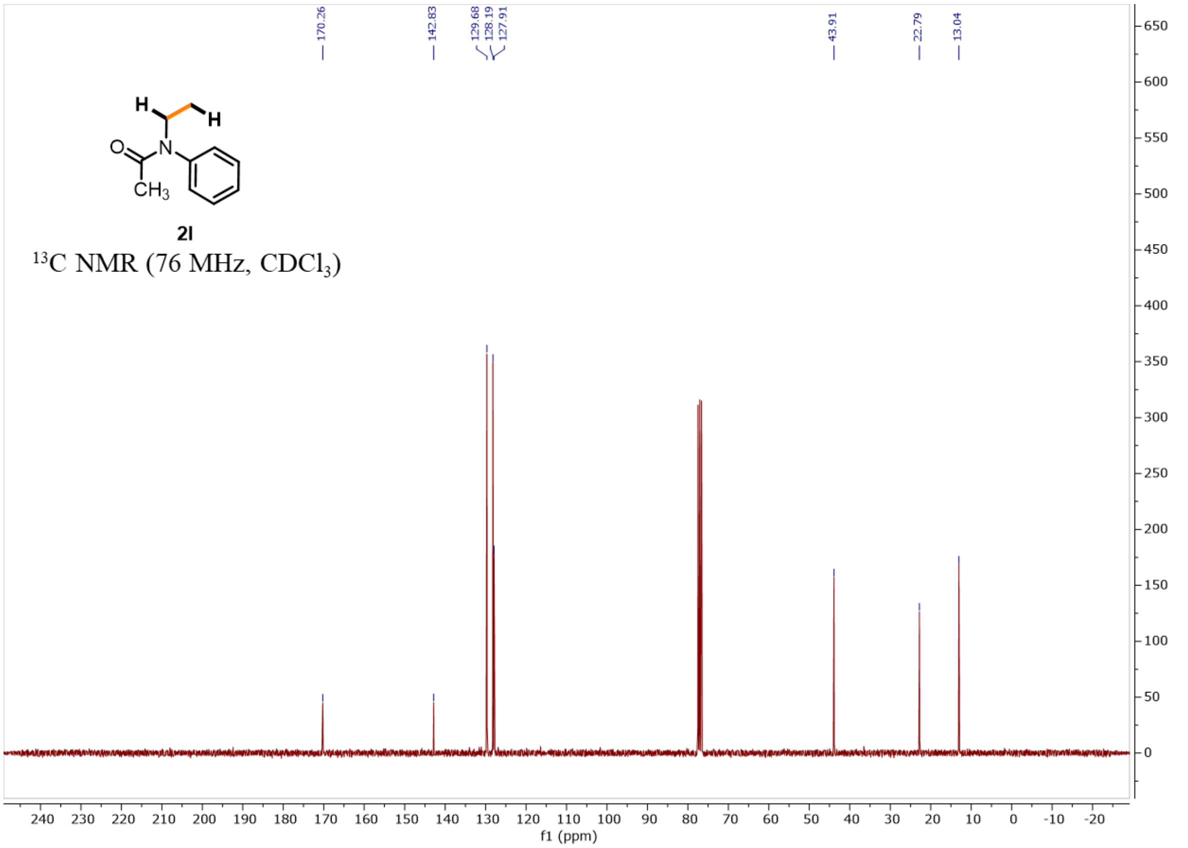
2l
13C NMR (76 MHz, CDCl3)
170.26
142.83
129.68 128.19 127.91
43.91
22.79
13.04
f1 (ppm)

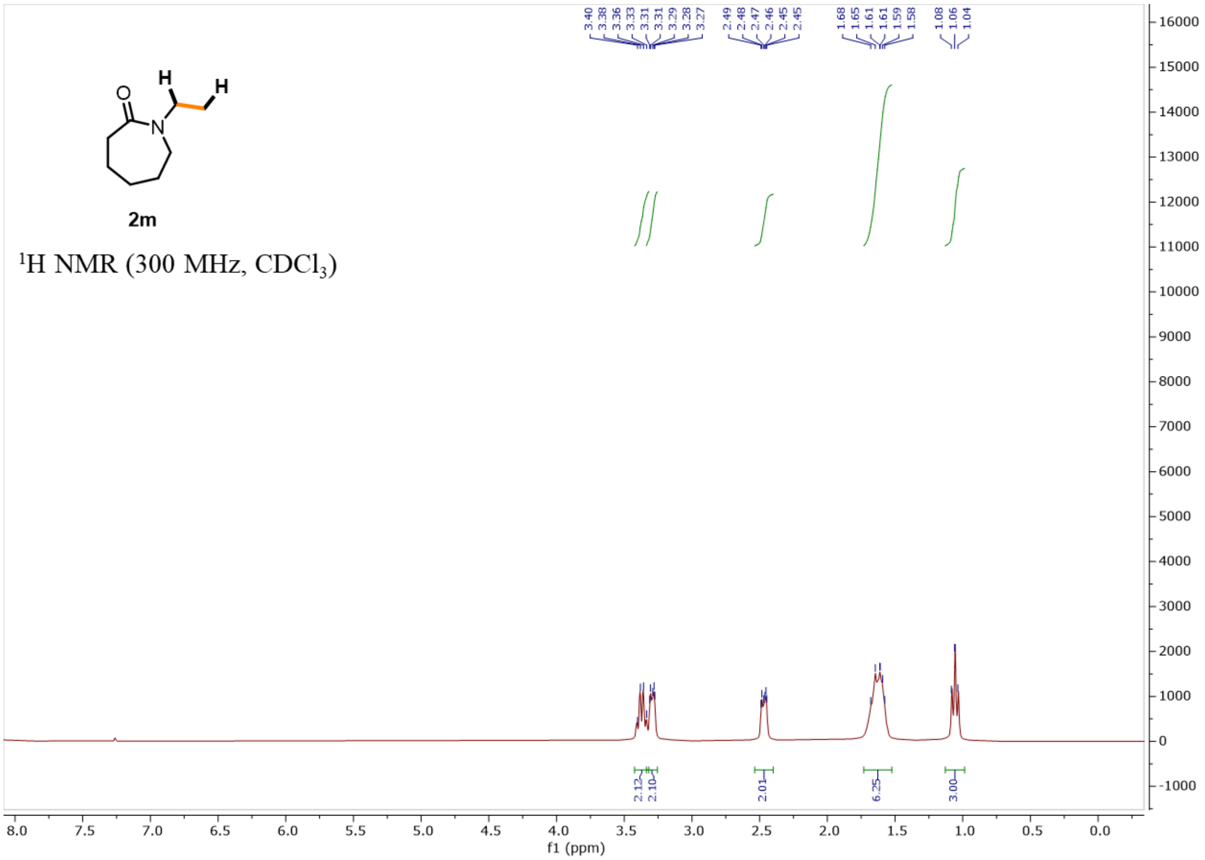

2m
$^{1}$H NMR (300 MHz, $CDCl_3$)
f1 (ppm)


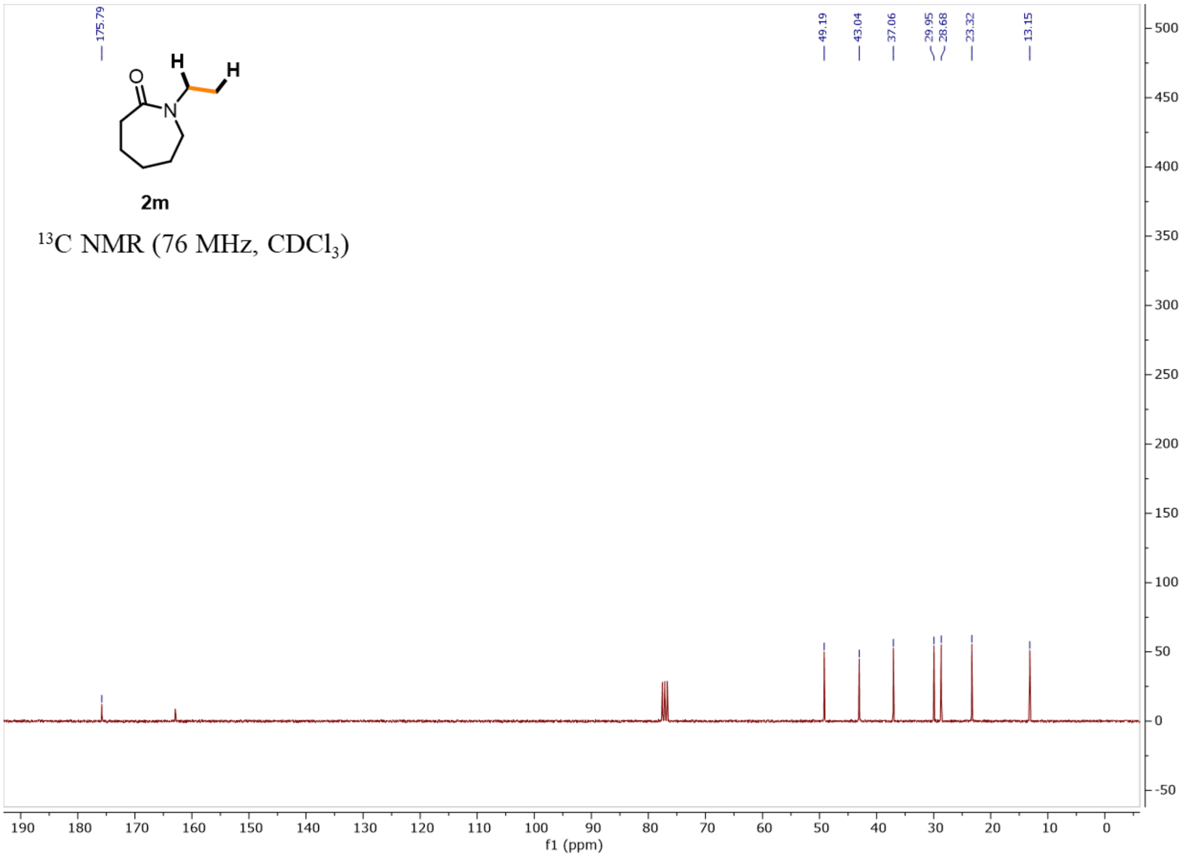

2m
$^{13}$C NMR (76 MHz, $CDCl_3$)
f1 (ppm)

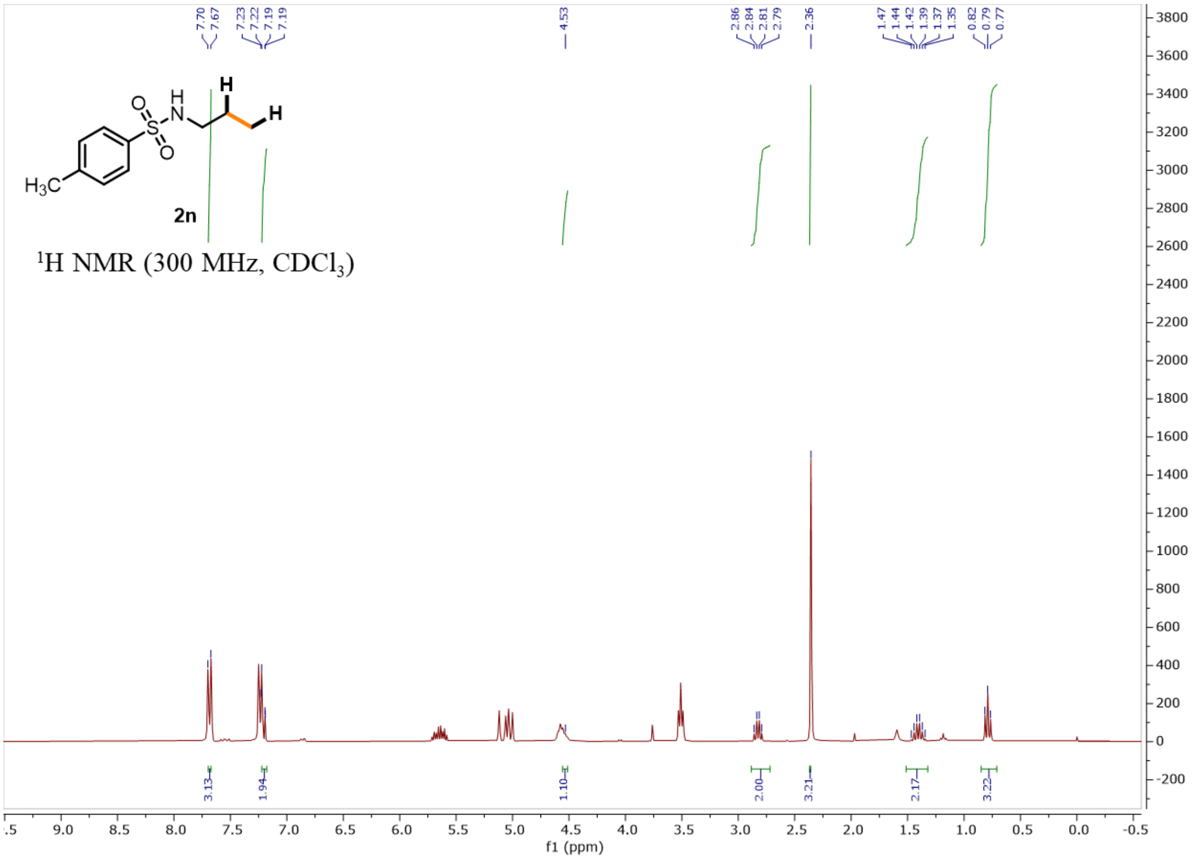

2n
$^1$H NMR (300 MHz, $CDCl_3$)
7.70
7.67
7.23
7.22
7.19
7.19
4.53
2.86
2.84
2.81
2.79
2.36
1.47
1.44
1.42
1.39
1.37
1.35
0.82
0.79
0.77
3.13
1.94
1.10
2.00
3.21
2.17
3.22
f1 (ppm)

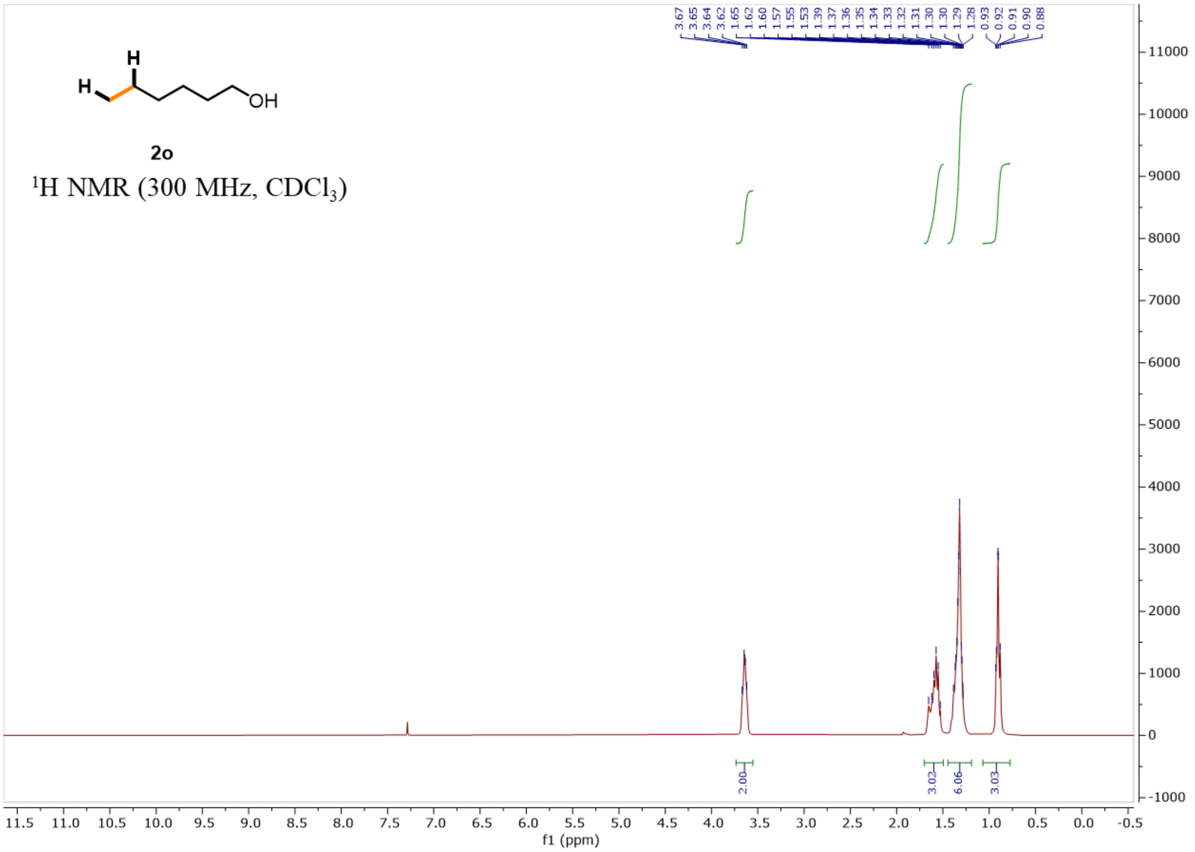

2o
$^{1}$H NMR (300 MHz, $CDCl_3$)
f1 (ppm)


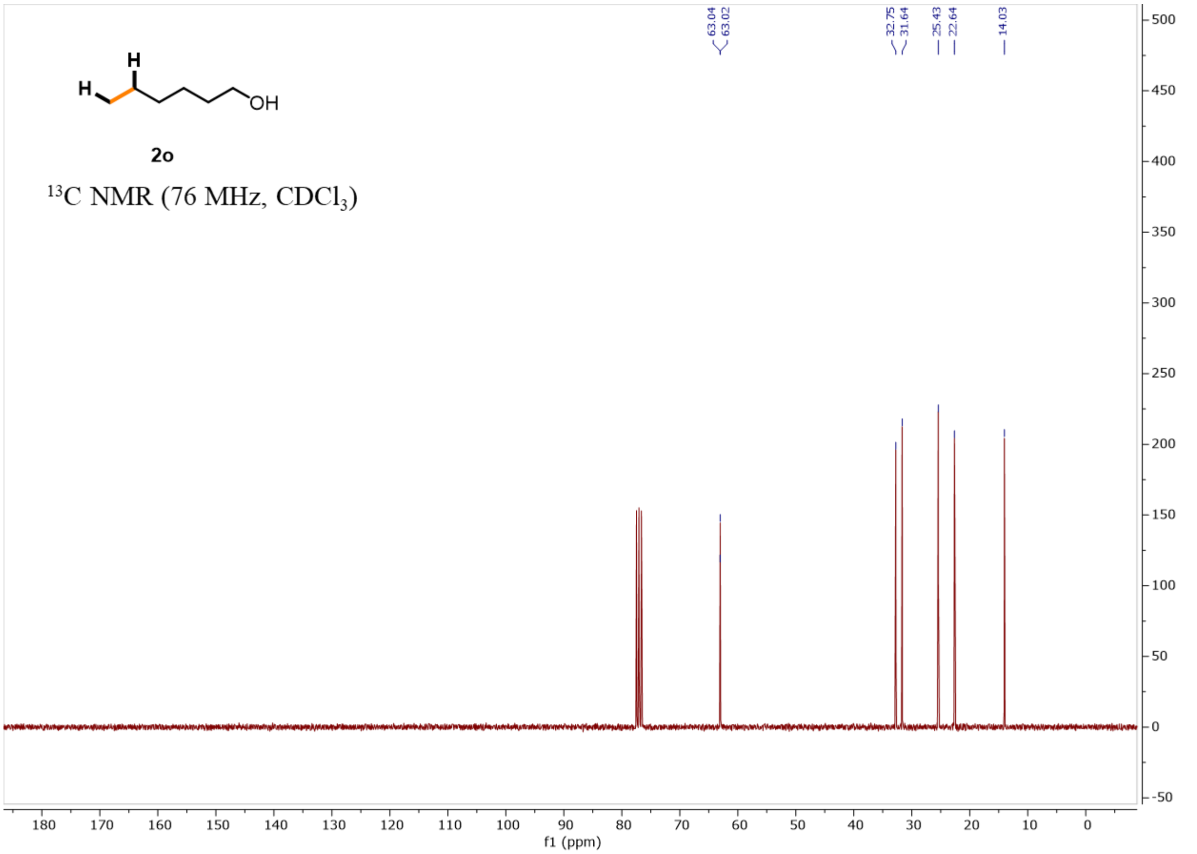

2o
$^{13}$C NMR (76 MHz, $CDCl_3$)
f1 (ppm)

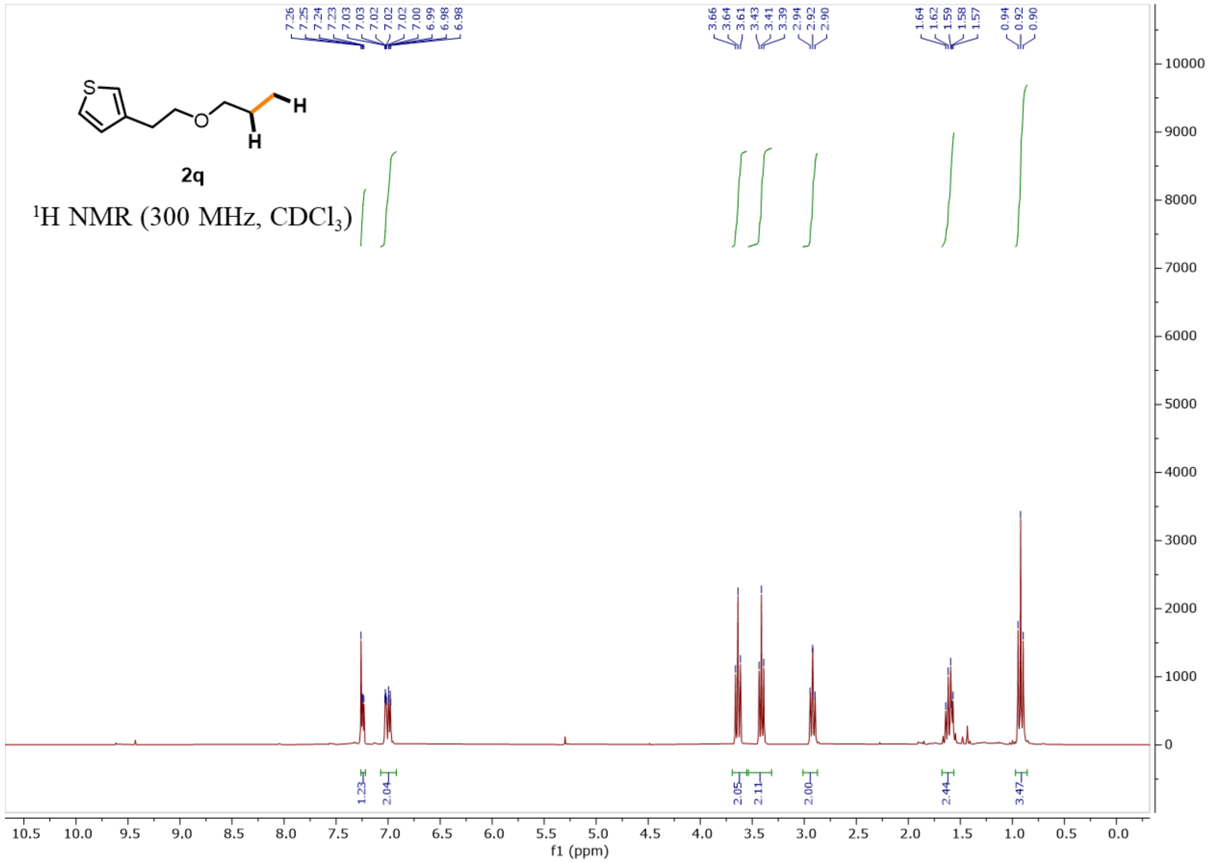
7.26 7.25 7.24 7.23 7.03 7.03 7.02 7.02 7.02 7.00 6.99 6.98 6.98
3.66 3.64 3.61 3.43 3.41 3.39 2.94 2.92 2.90
1.64 1.62 1.59 1.58 1.57 0.94 0.92 0.90
2q
$^1$H NMR (300 MHz, CDCl$_3$)
1.23 2.04 2.05 2.11 2.00 2.44 3.47
f1 (ppm)

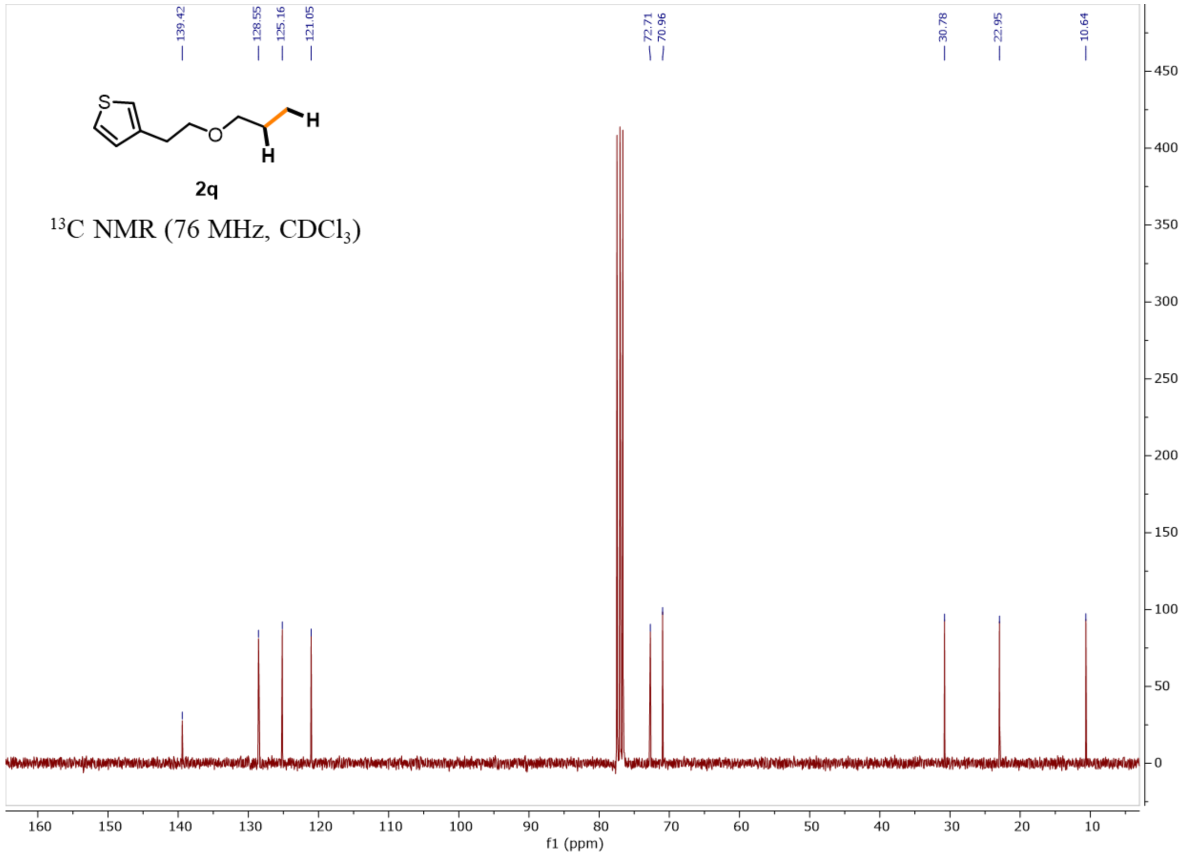
139.42 128.55 125.16 121.05 72.71 70.96 30.78 22.95 10.64
2q
$^{13}$C NMR (76 MHz, CDCl$_3$)
f1 (ppm)

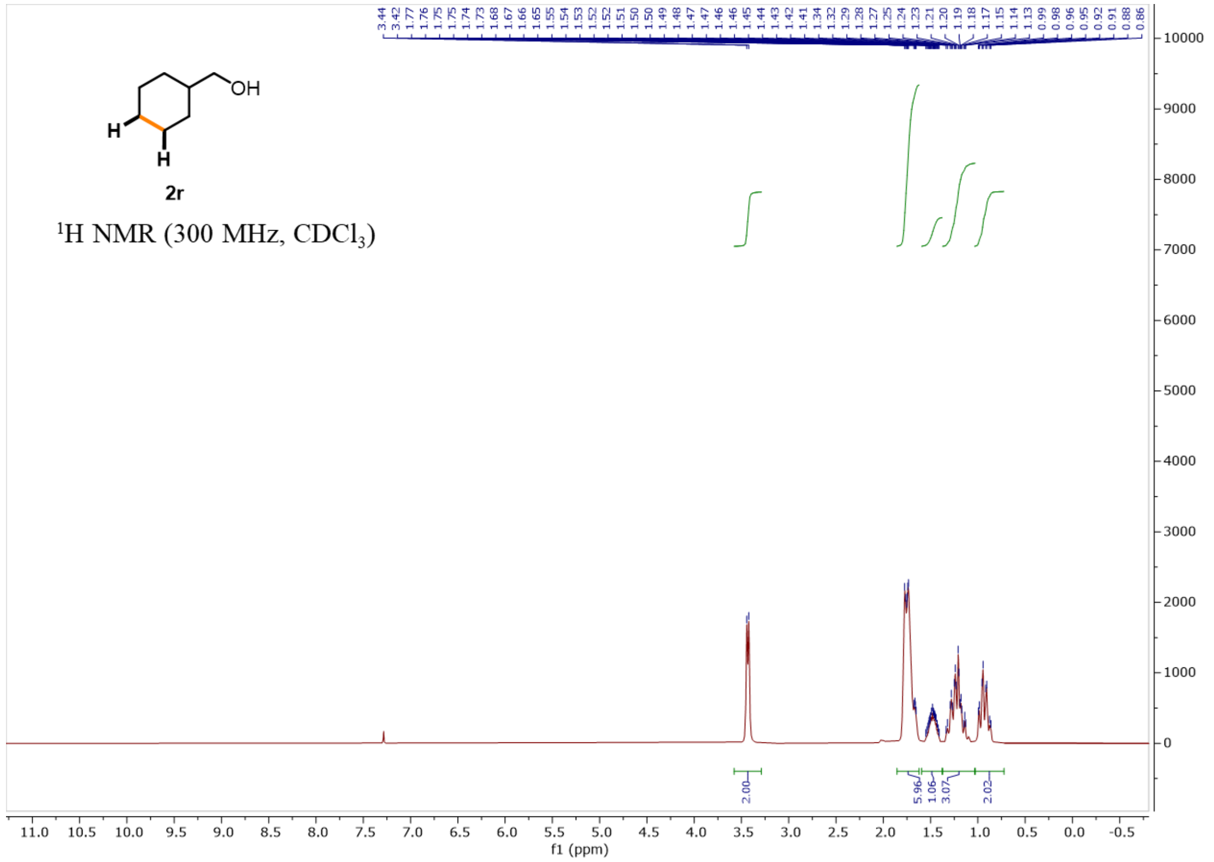
3.44 3.42 1.77 1.76 1.75 1.74 1.73 1.68 1.67 1.66 1.65 1.55 1.54 1.53 1.52 1.52 1.51 1.50 1.50 1.49 1.48 1.47 1.47 1.46 1.45 1.44 1.43 1.42 1.41 1.34 1.32 1.29 1.28 1.27 1.25 1.24 1.23 1.21 1.20 1.19 1.18 1.17 1.15 1.14 1.13 0.99 0.98 0.96 0.95 0.92 0.91 0.88 0.86
OH
H
H
2r
1H NMR (300 MHz, CDCl3)
2.00
5.96
1.06
3.07
2.02
f1 (ppm)

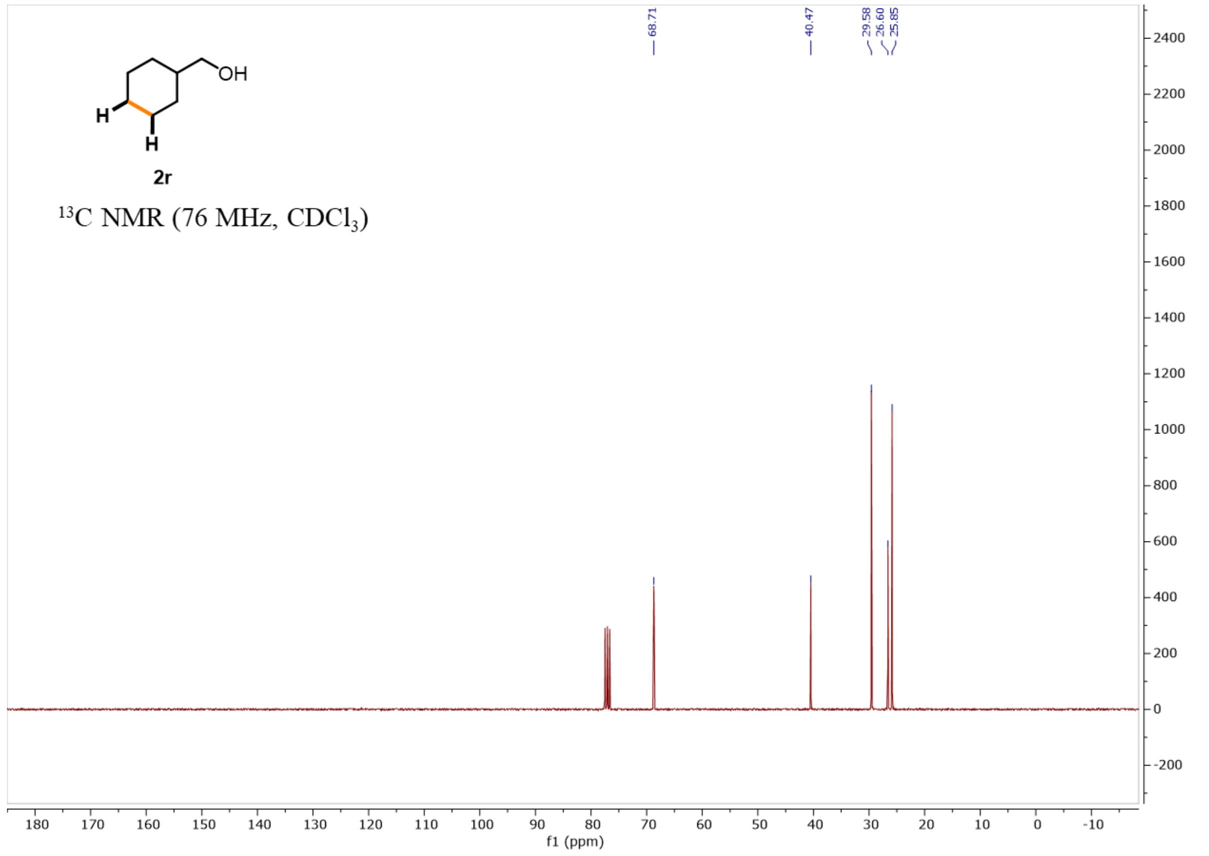
68.71
40.47
29.58
26.60
25.85
OH
H
H
2r
13C NMR (76 MHz, CDCl3)
f1 (ppm)

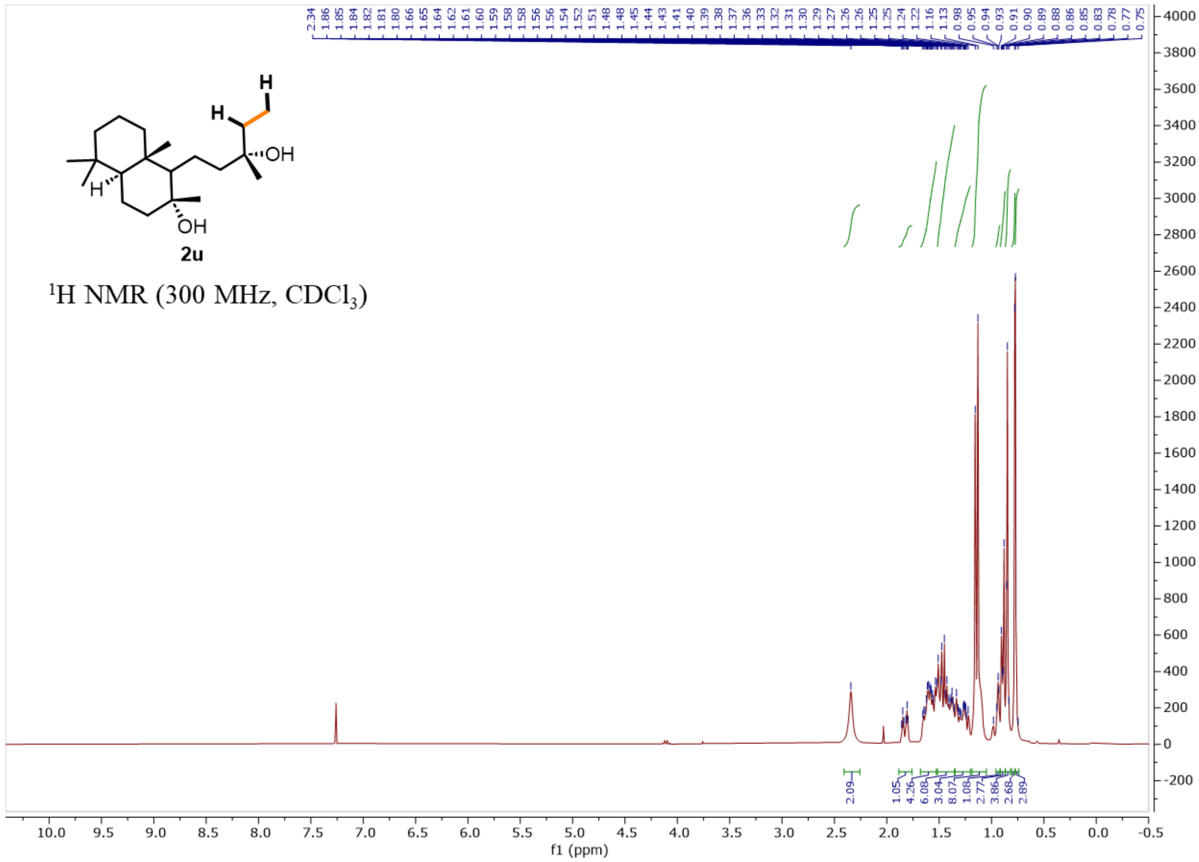
2u
$^{1}H$ NMR (300 MHz, $CDCl_3$)
2.34 1.86 1.85 1.84 1.82 1.81 1.80 1.66 1.65 1.64 1.62 1.61 1.60 1.59 1.58 1.58 1.56 1.56 1.54 1.52 1.51 1.48 1.48 1.45 1.44 1.43 1.41 1.40 1.39 1.38 1.37 1.36 1.33 1.32 1.31 1.30 1.29 1.27 1.26 1.26 1.25 1.25 1.24 1.22 1.16 1.13 0.98 0.95 0.94 0.93 0.91 0.90 0.89 0.88 0.86 0.85 0.83 0.78 0.77 0.75
2.09 1.05 4.26 6.08 3.04 8.07 1.08 2.77 3.86 2.68 2.89
f1 (ppm)

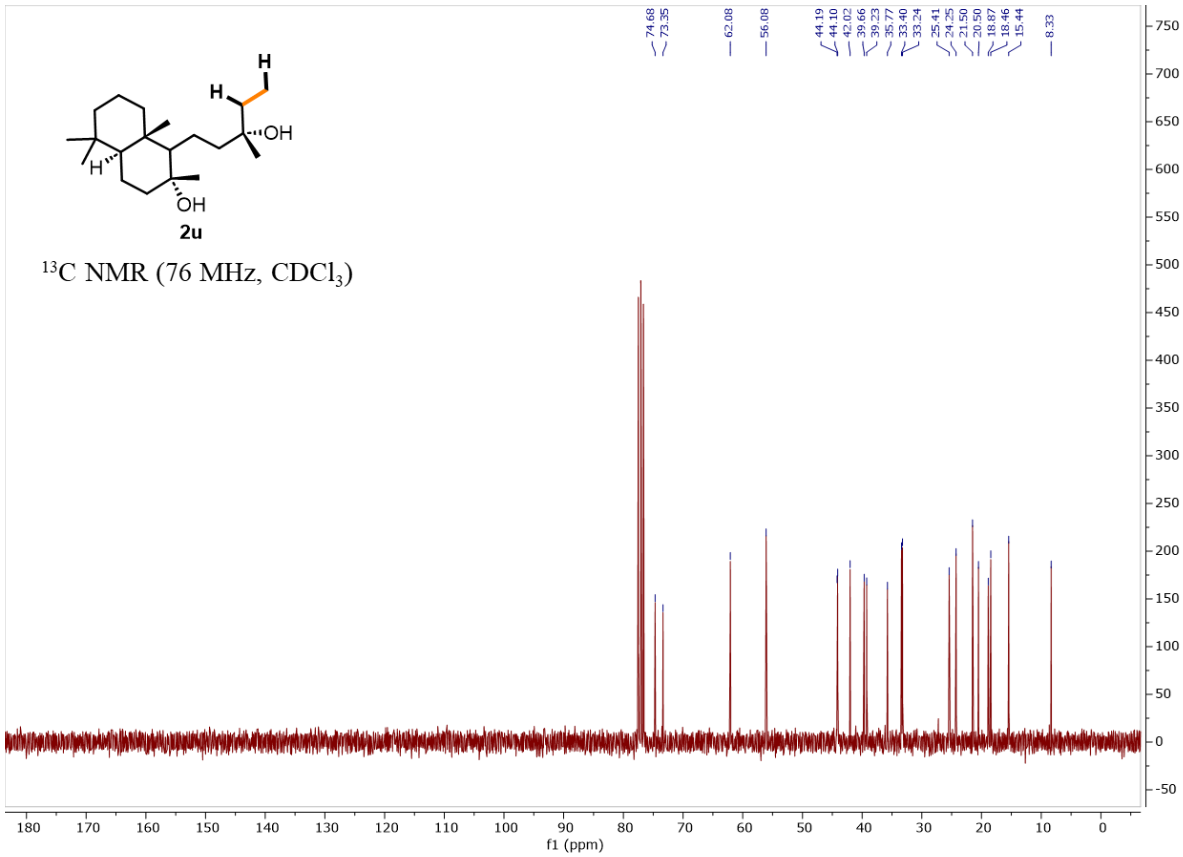
2u
$^{13}C$ NMR (76 MHz, $CDCl_3$)
74.68 73.35 62.08 56.08 44.19 44.10 42.02 39.66 39.23 35.77 33.40 33.24 25.41 24.25 21.50 20.50 18.87 18.46 15.44 8.33
f1 (ppm)

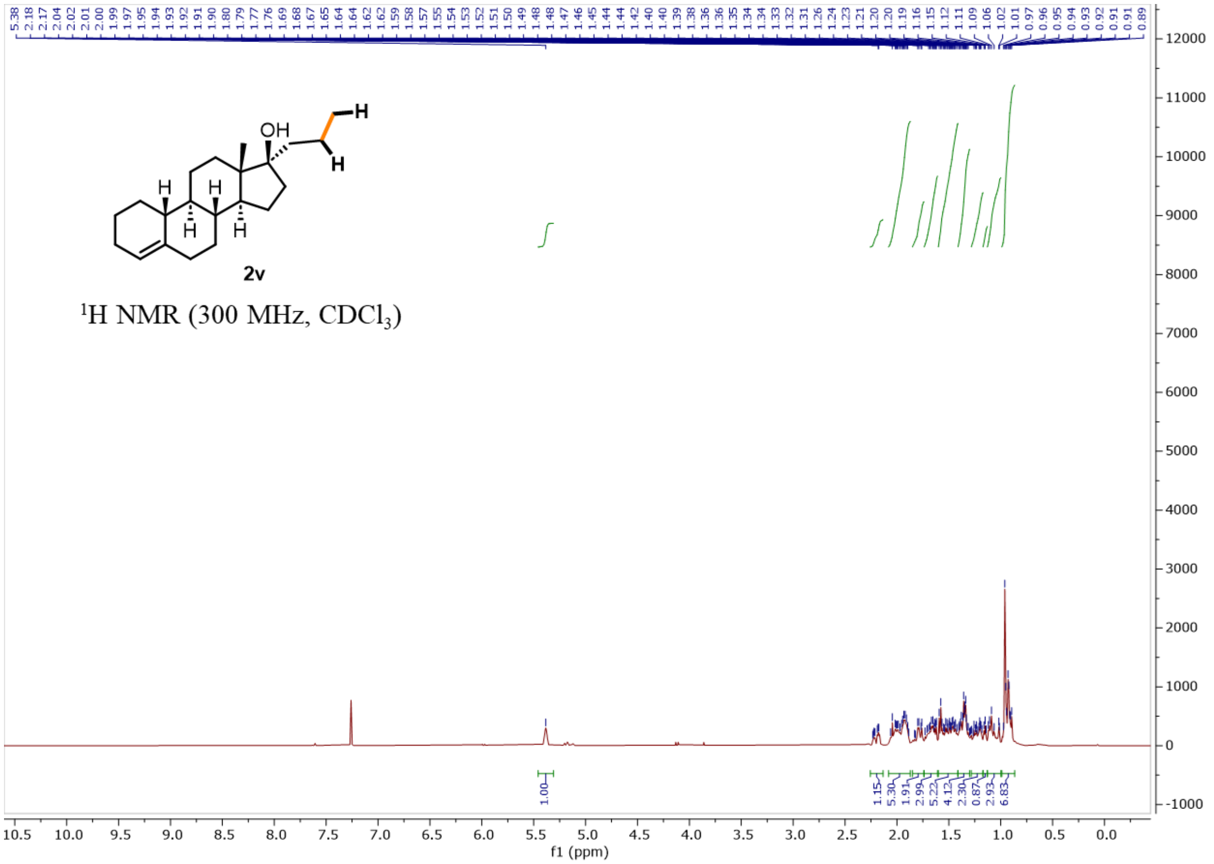
2v
$^1$H NMR (300 MHz, CDCl$_3$)
f1 (ppm)

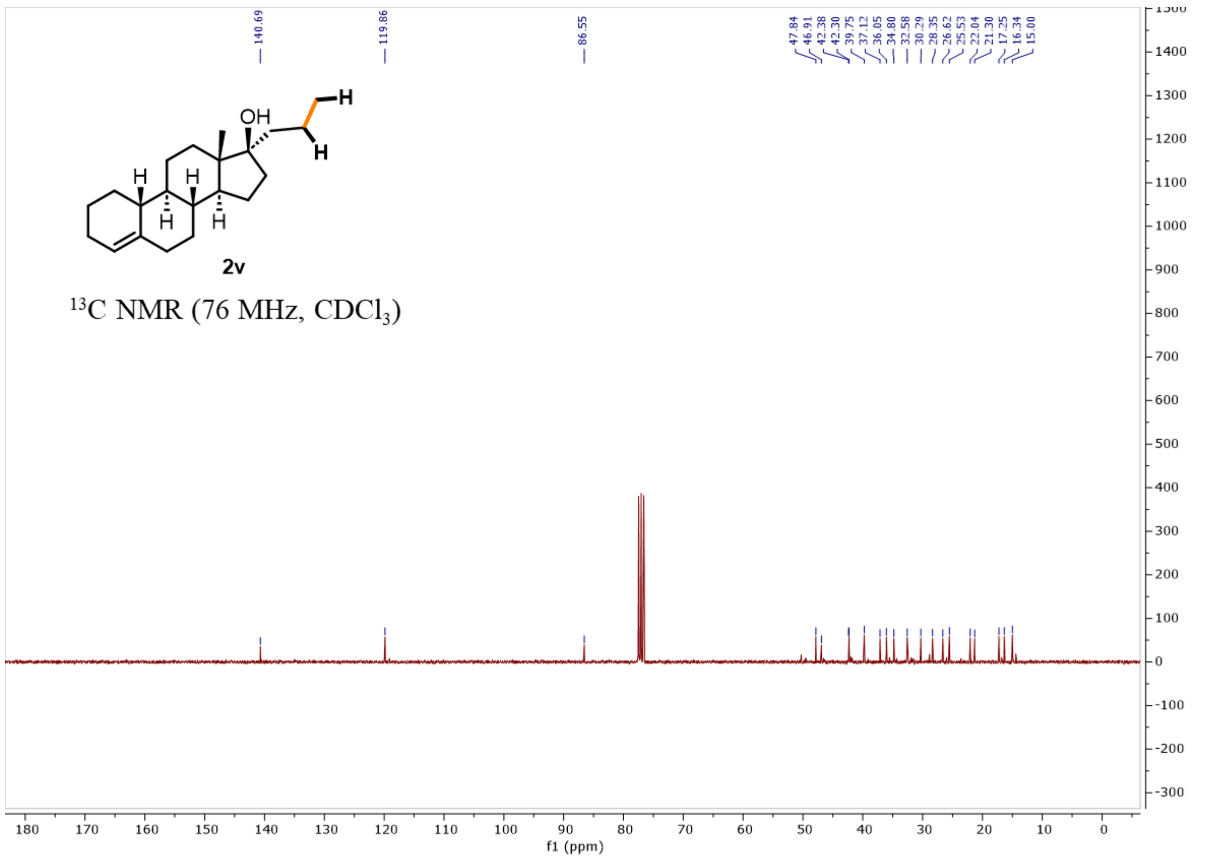
2v
$^{13}$C NMR (76 MHz, CDCl$_3$)
f1 (ppm)

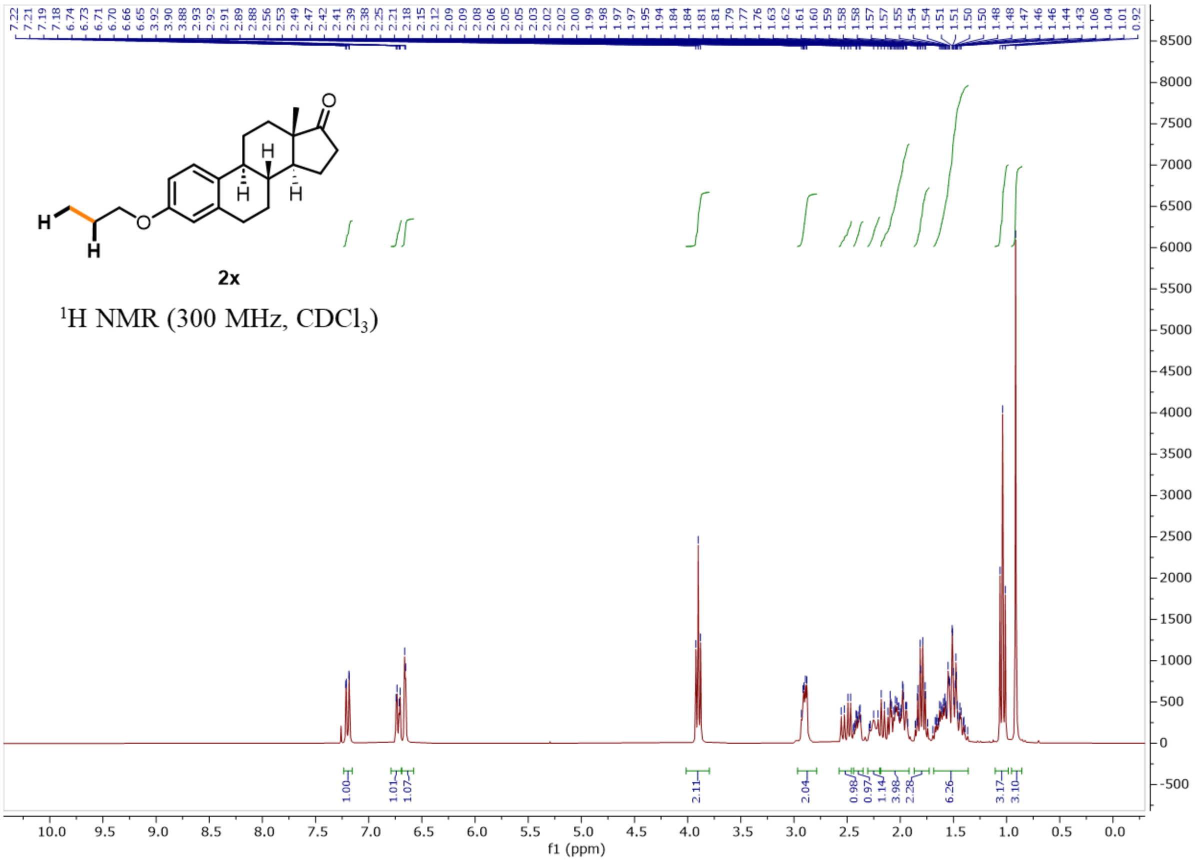

2x
¹H NMR (300 MHz, CDCl3)
f1 (ppm)


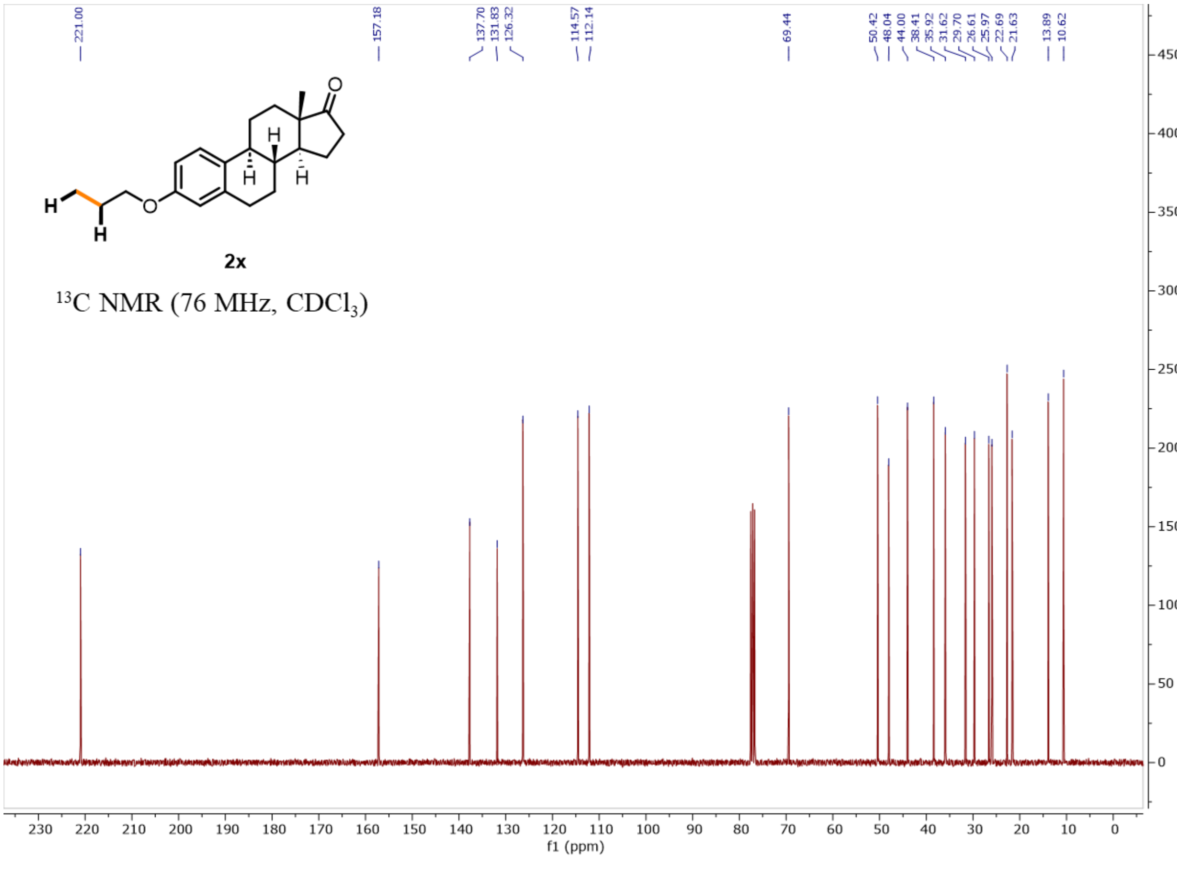

221.00
157.18
137.70
131.83
126.32
114.57
112.14
69.44
50.42
48.04
44.00
38.41
35.92
31.62
29.70
26.61
25.97
22.69
21.63
13.89
10.62
2x
¹³C NMR (76 MHz, CDCl3)
f1 (ppm)

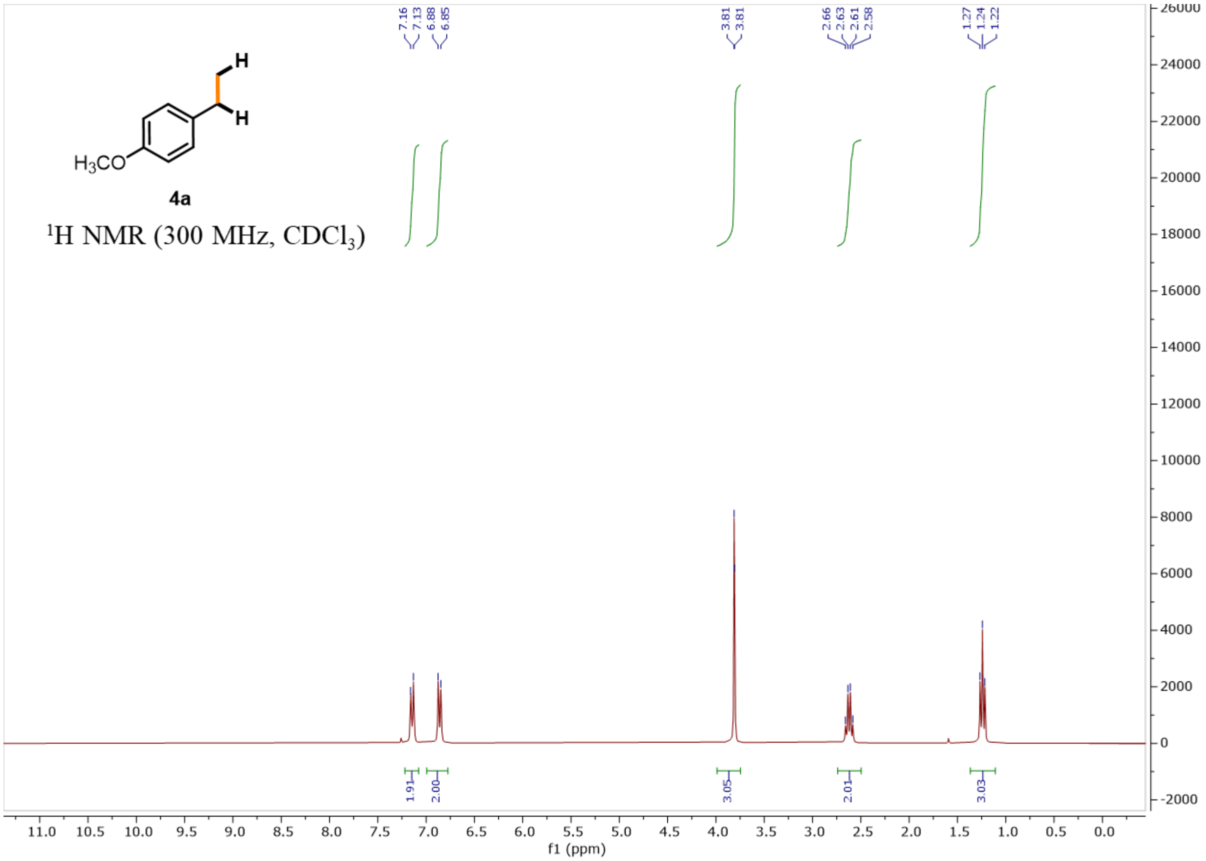

H3CO
4a
1H NMR (300 MHz, CDCl3)
7.16
7.13
6.88
6.85
3.81
3.81
2.66
2.63
2.61
2.58
1.27
1.24
1.22
1.91
2.00
3.05
2.01
3.03
f1 (ppm)


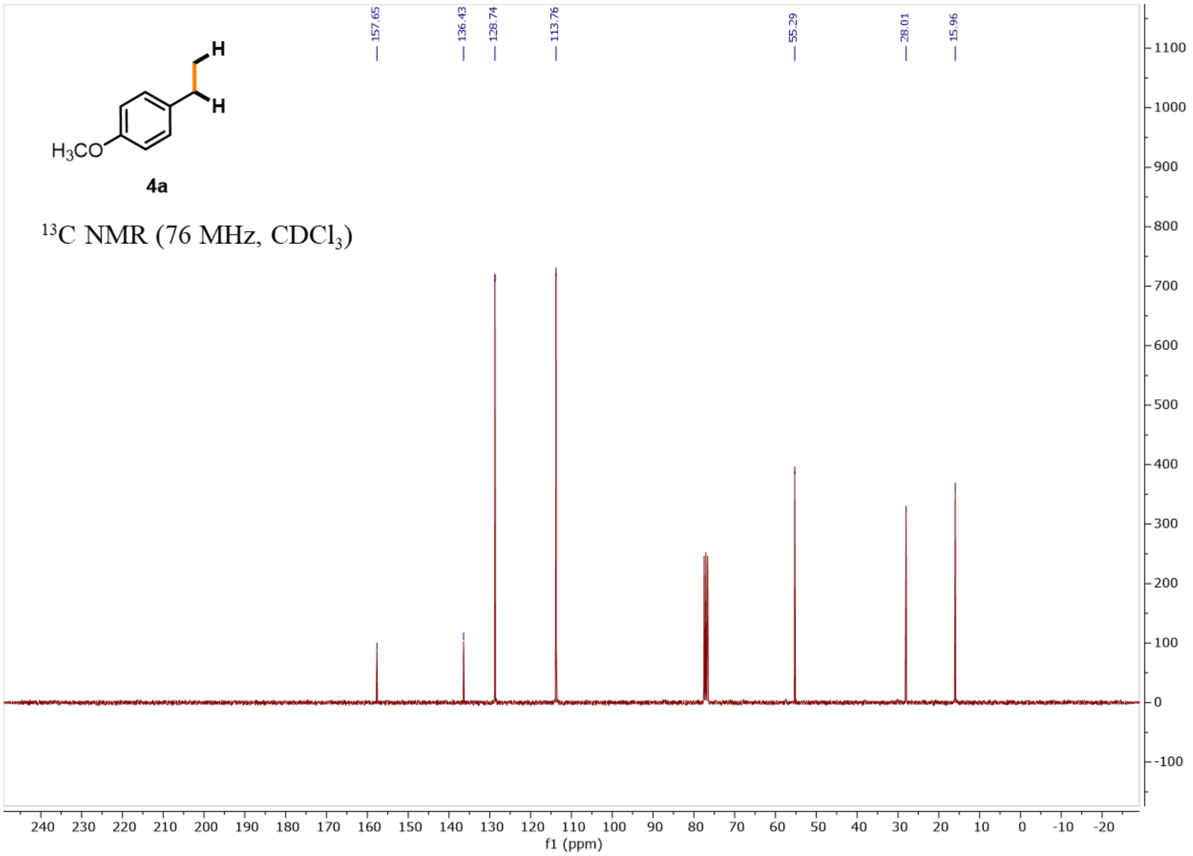

H3CO
4a
13C NMR (76 MHz, CDCl3)
157.65
136.43
128.74
113.76
55.29
28.01
15.96
f1 (ppm)

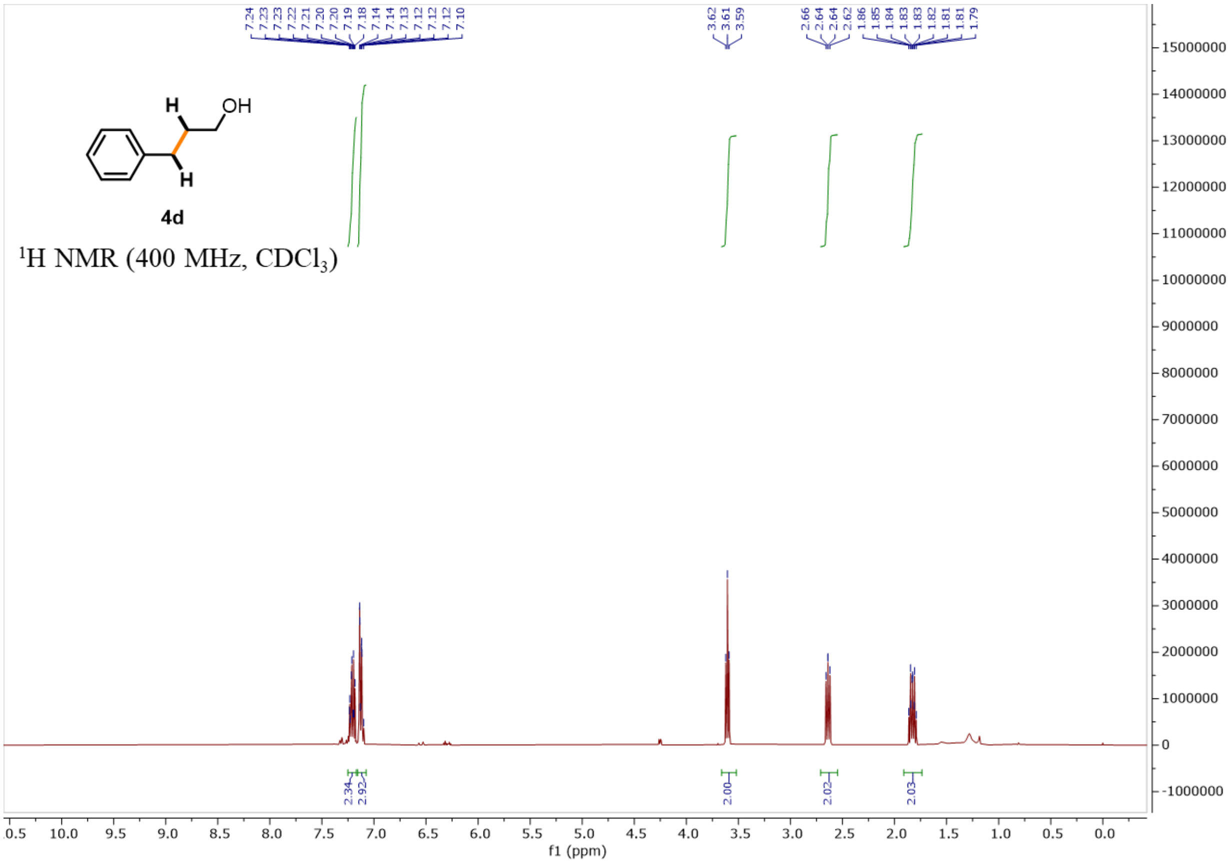

4d
$^{1}H$ NMR (400 MHz, $CDCl_3$)


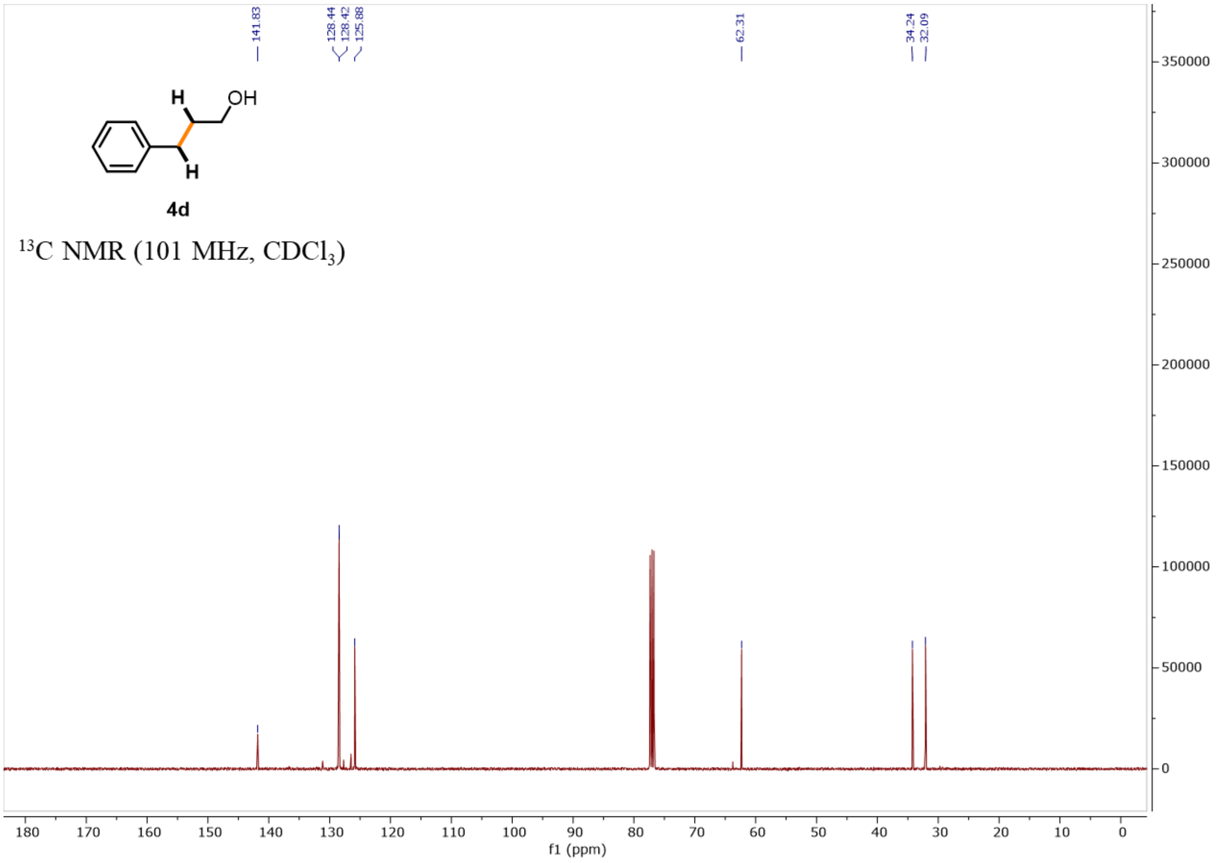

4d
$^{13}C$ NMR (101 MHz, $CDCl_3$)
141.83
128.44
128.42
125.88
62.31
34.24
32.09
f1 (ppm)

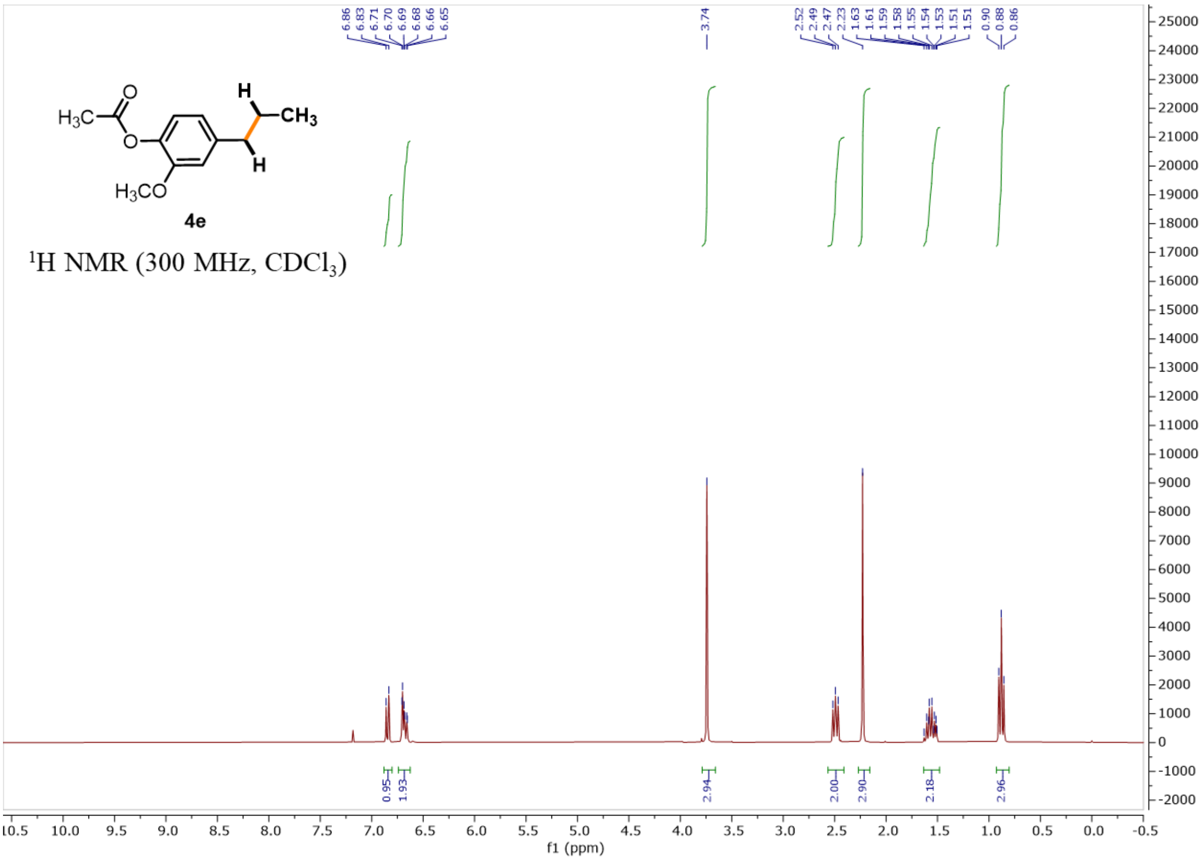


$^{1}H$ NMR (300 MHz, $CDCl_3$)

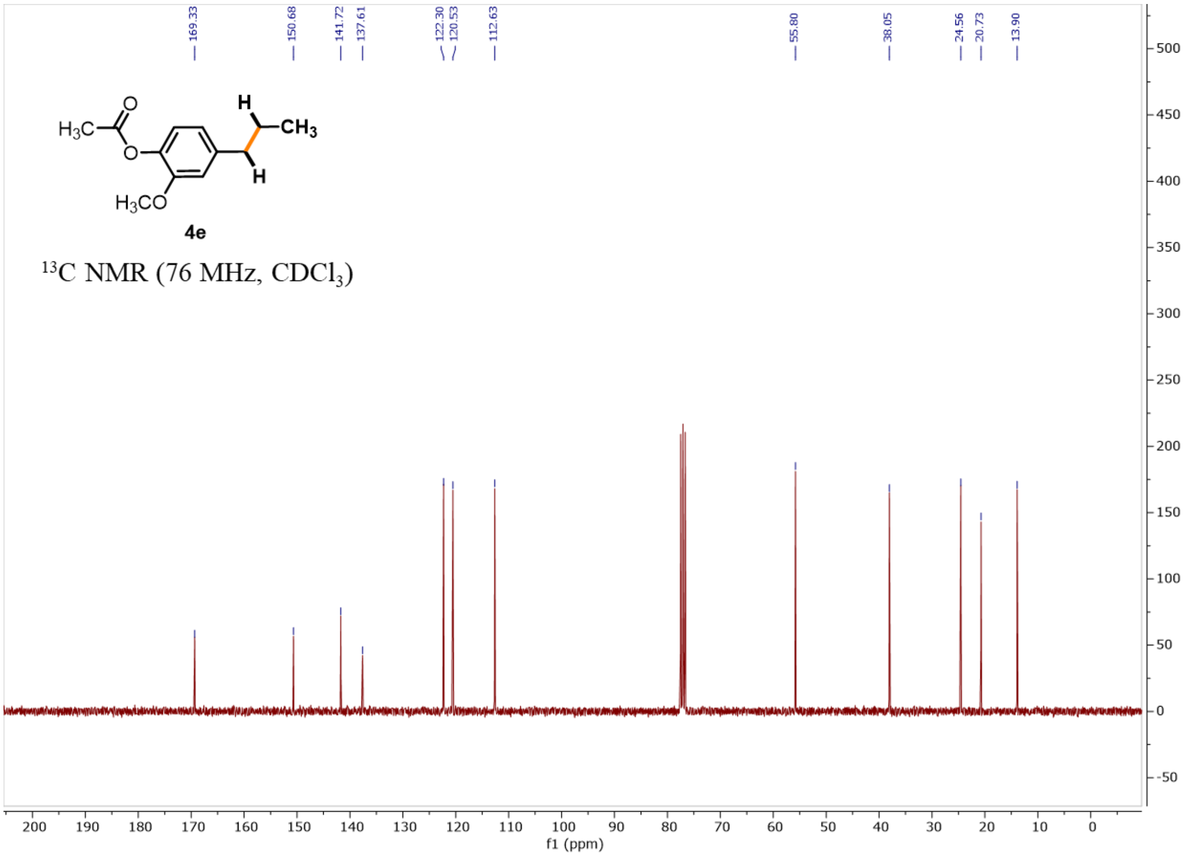


$^{13}C$ NMR (76 MHz, $CDCl_3$)

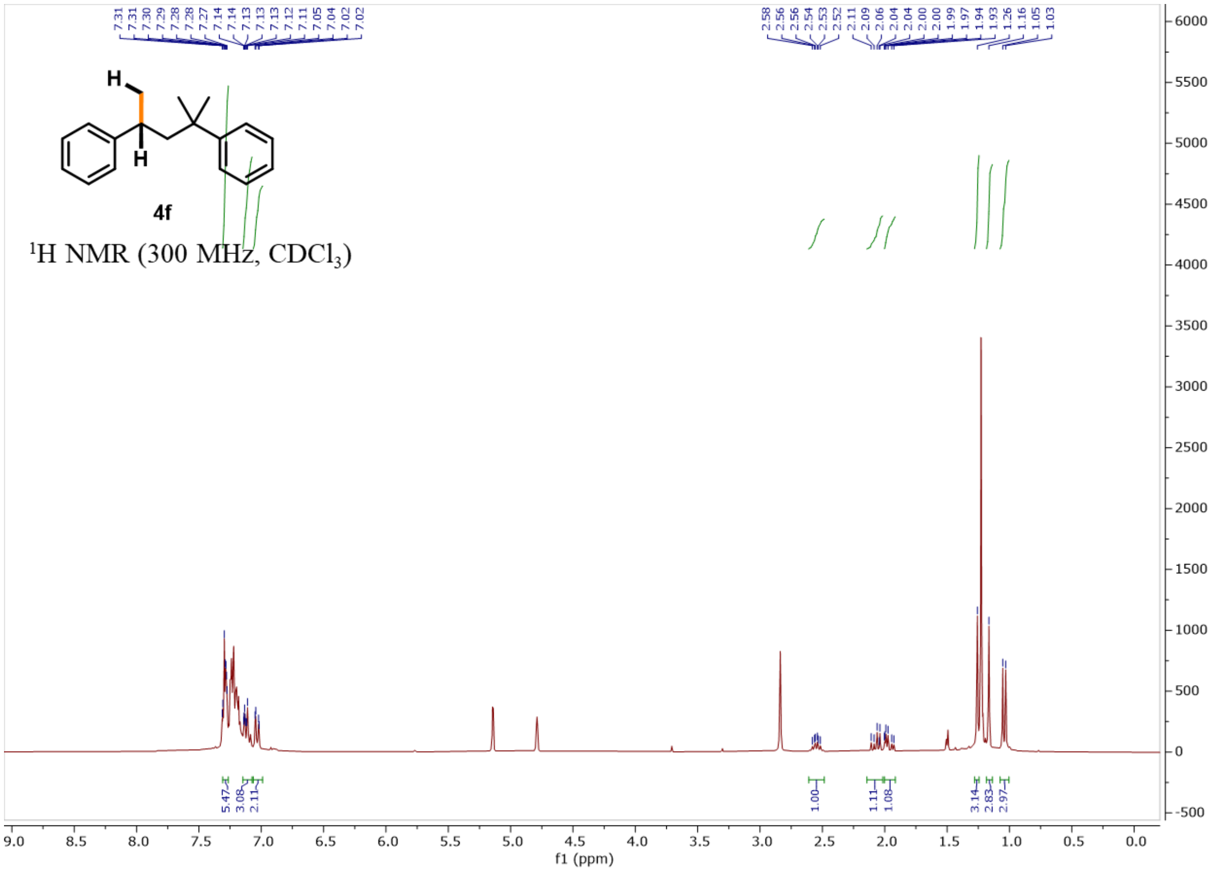

4f
$^{1}$H NMR (300 MHz, CDCl$_3$)
f1 (ppm)

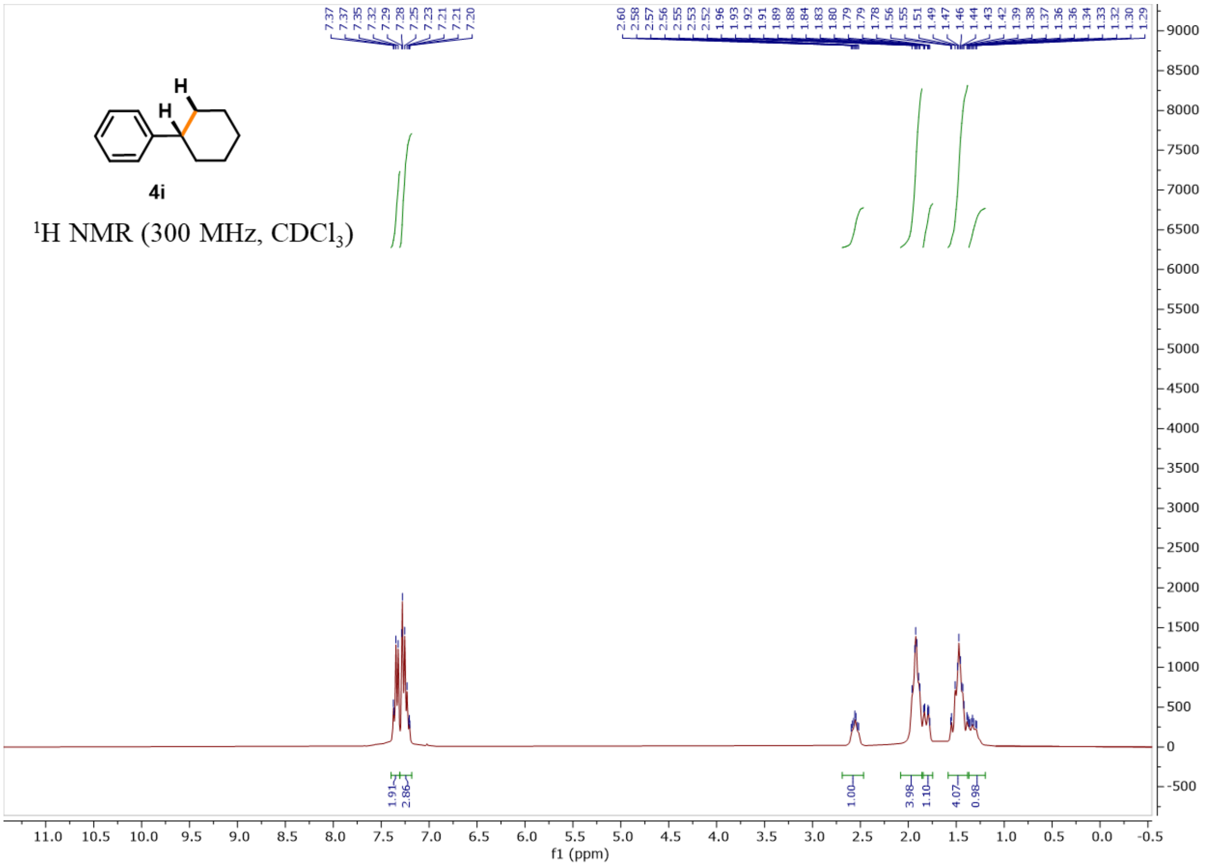

H
H
4i
$^1$H NMR (300 MHz, CDCl$_3$)
f1 (ppm)


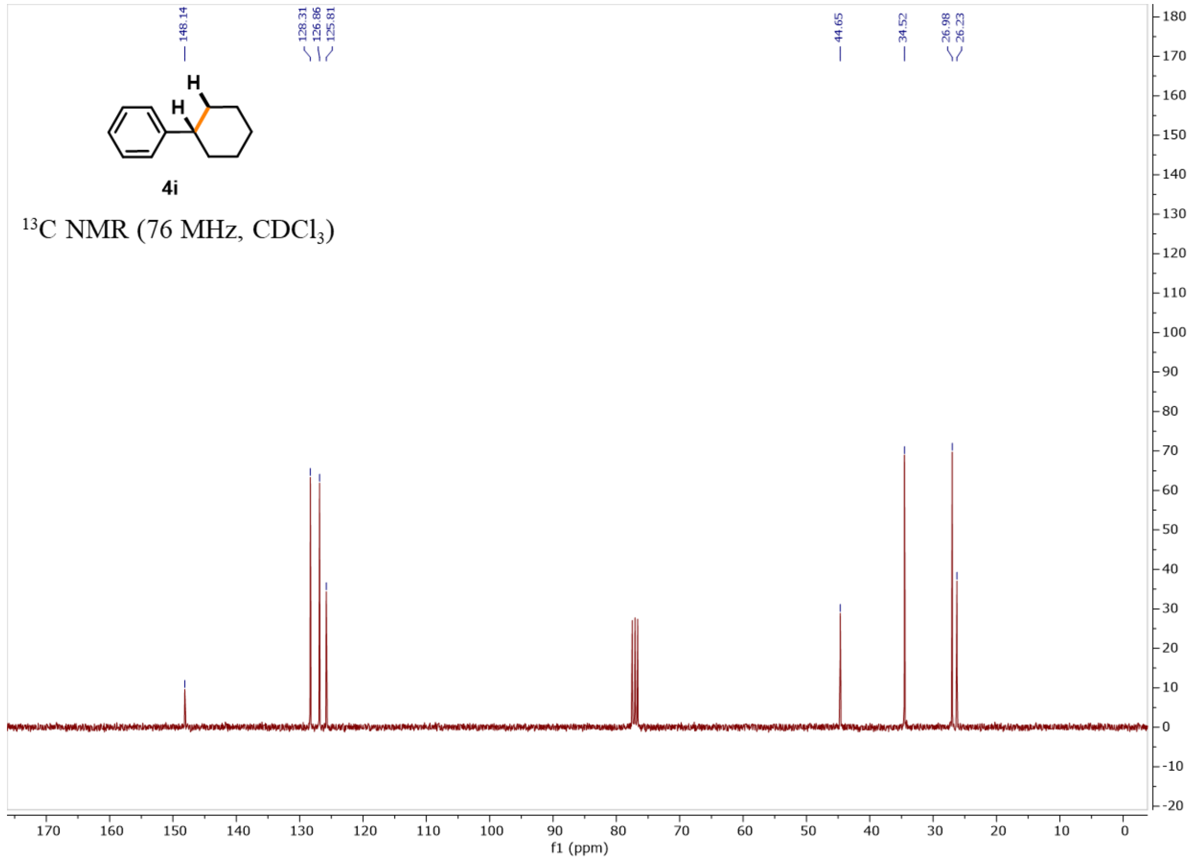

H
H
4i
$^{13}$C NMR (76 MHz, CDCl$_3$)
148.14
128.31
126.86
125.81
44.65
34.52
26.98
26.23
f1 (ppm)

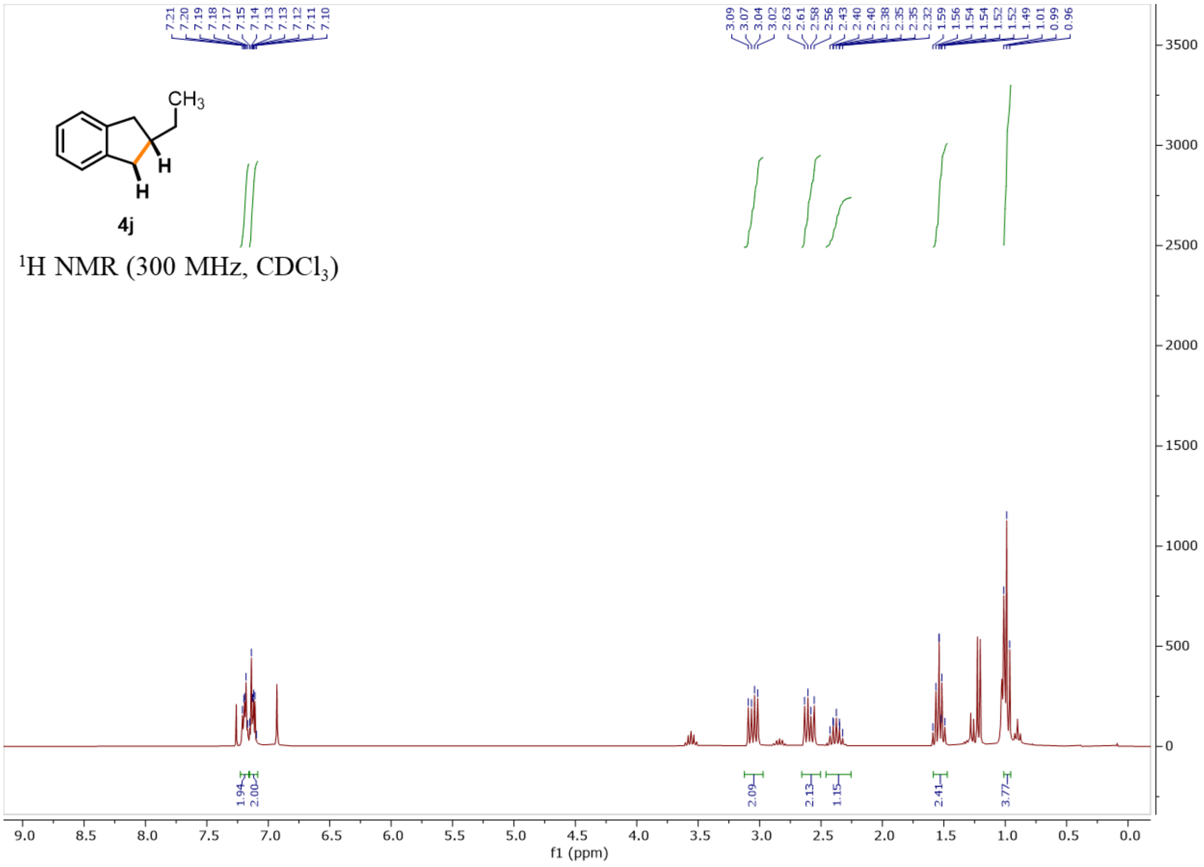

CH3
H
H
4j
1H NMR (300 MHz, CDCl3)
7.21 7.20 7.19 7.18 7.17 7.15 7.14 7.13 7.13 7.12 7.11 7.10
3.09 3.07 3.04 3.02 2.63 2.61 2.58 2.56 2.43 2.40 2.40 2.38 2.35 2.35 2.32 1.59 1.56 1.54 1.54 1.52 1.52 1.49 1.01 0.99 0.96
1.94 2.00 2.09 2.13 1.15 2.41 3.77
9.0 8.5 8.0 7.5 7.0 6.5 6.0 5.5 5.0 4.5 4.0 3.5 3.0 2.5 2.0 1.5 1.0 0.5 0.0
f1 (ppm)
3500 3000 2500 2000 1500 1000 500 0

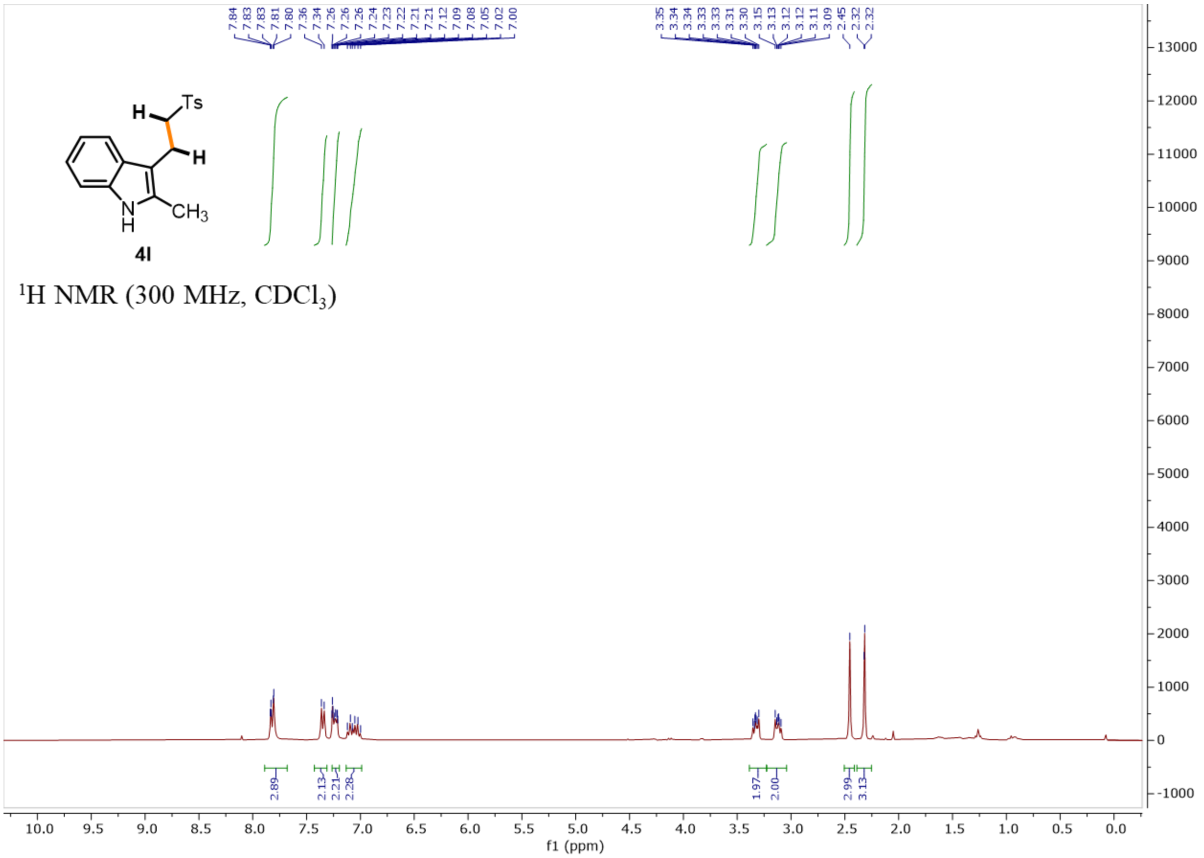

Ts
H
H
N
H
CH3
4l
1H NMR (300 MHz, CDCl3)
7.84 7.83 7.83 7.81 7.80 7.36 7.34 7.26 7.26 7.26 7.24 7.23 7.22 7.21 7.21 7.12 7.09 7.08 7.05 7.02 7.00
3.35 3.34 3.34 3.33 3.33 3.31 3.30 3.15 3.13 3.12 3.12 3.11 3.09 2.45 2.32 2.32
2.89 2.13 2.21 2.28 1.97 2.00 2.99 3.13
f1 (ppm)


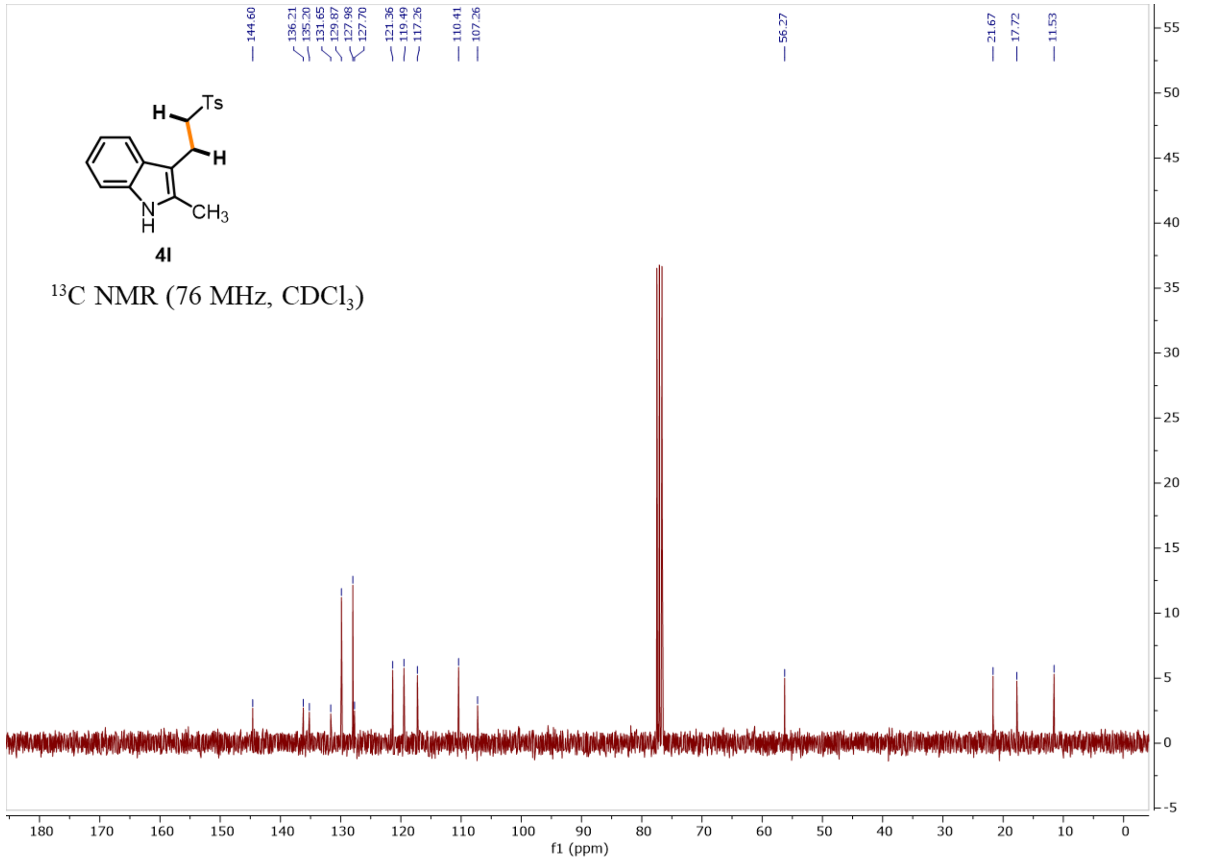

Ts
H
H
N
H
CH3
4l
13C NMR (76 MHz, CDCl3)
144.60 136.21 135.20 131.65 129.87 127.98 127.70 121.36 119.49 117.26 110.41 107.26
56.27
21.67 17.72 11.53
f1 (ppm)

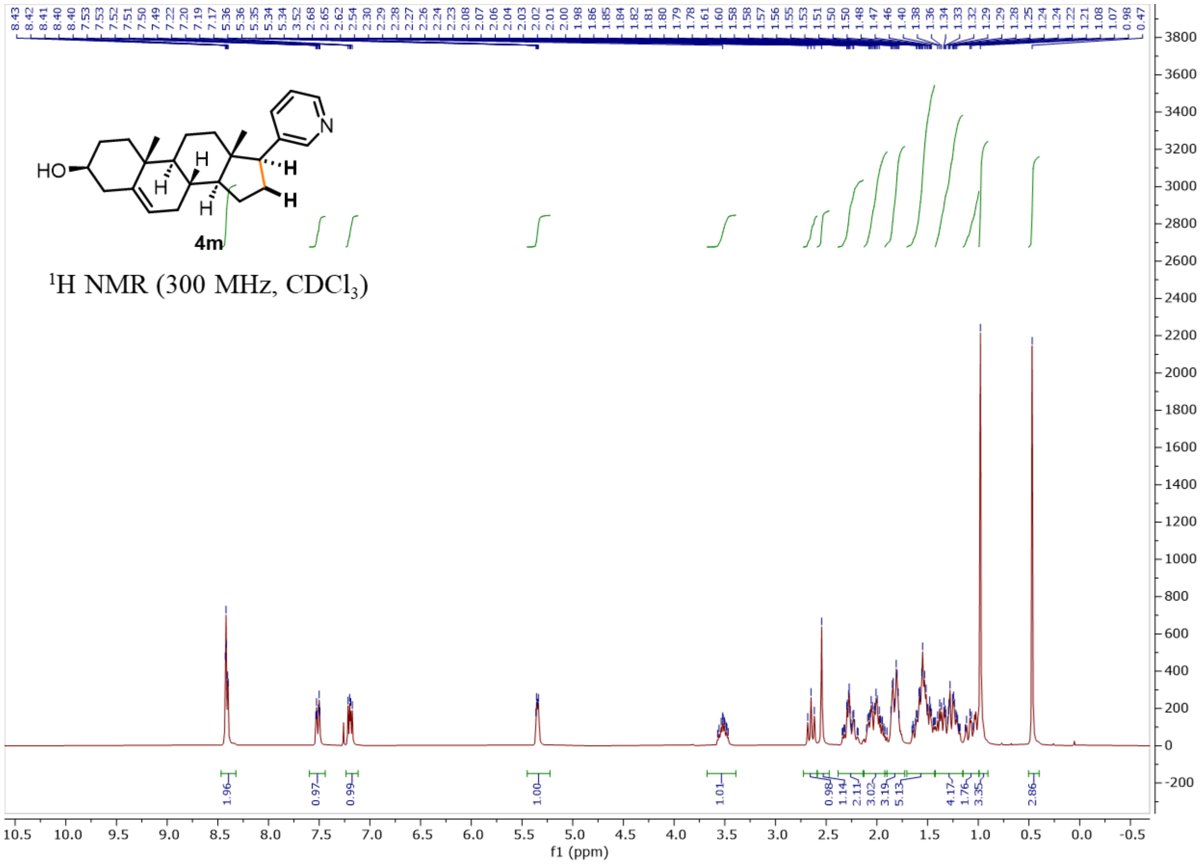
HO
4m
¹H NMR (300 MHz, CDCl₃)
f1 (ppm)

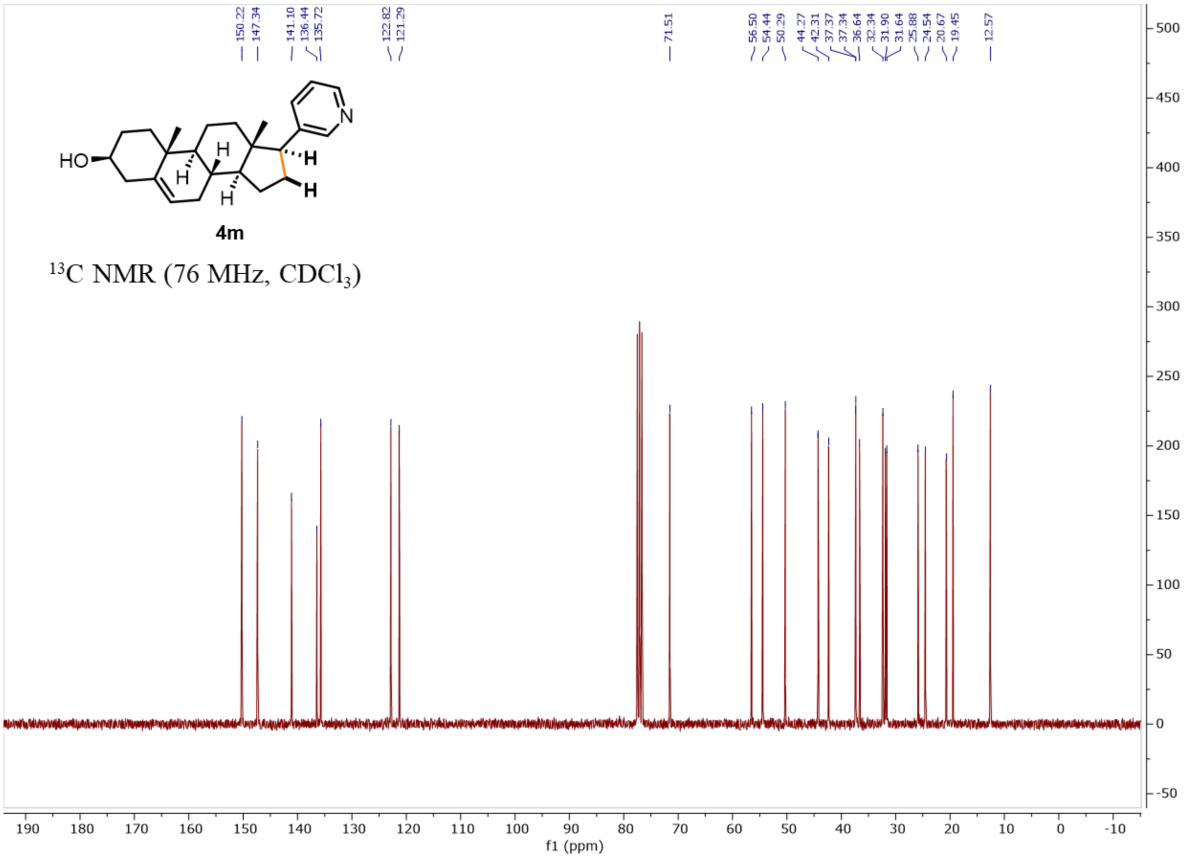
HO
4m
¹³C NMR (76 MHz, CDCl₃)
f1 (ppm)

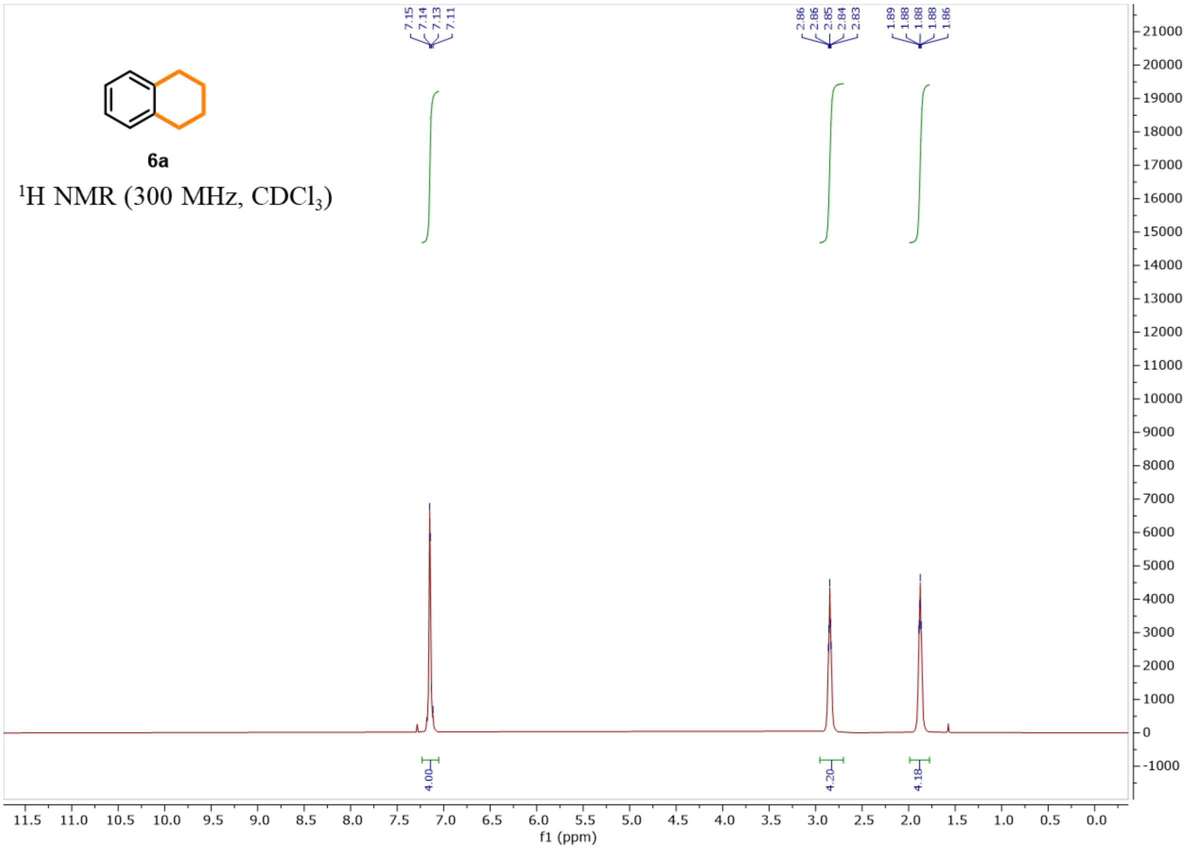
6a
$^{1}$H NMR (300 MHz, $CDCl_3$)
7.15
7.14
7.13
7.11
2.86
2.86
2.85
2.84
2.83
1.89
1.88
1.88
1.88
1.86
4.00
4.20
4.18
f1 (ppm)

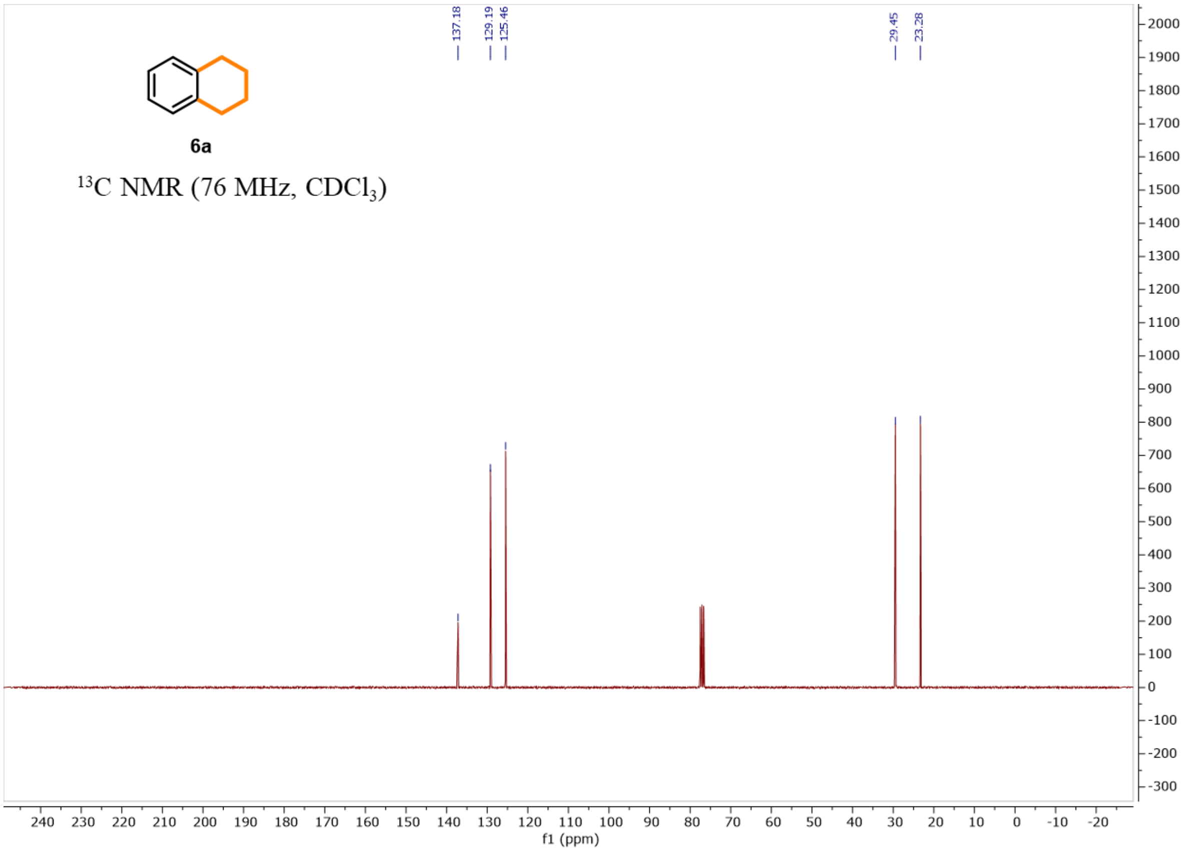
6a
$^{13}$C NMR (76 MHz, $CDCl_3$)
137.18
129.19
125.46
29.45
23.28
f1 (ppm)

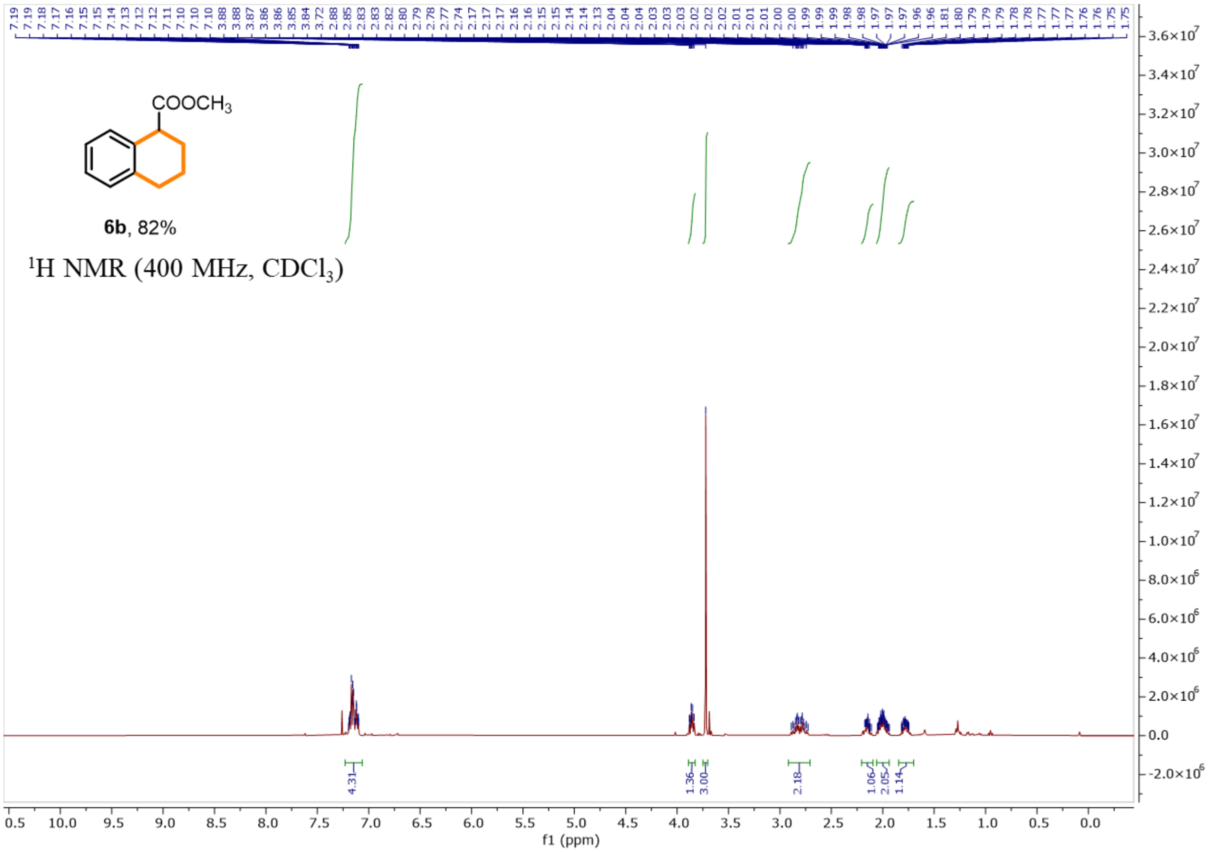
COOCH3
6b, 82%
1H NMR (400 MHz, CDCl3)
f1 (ppm)

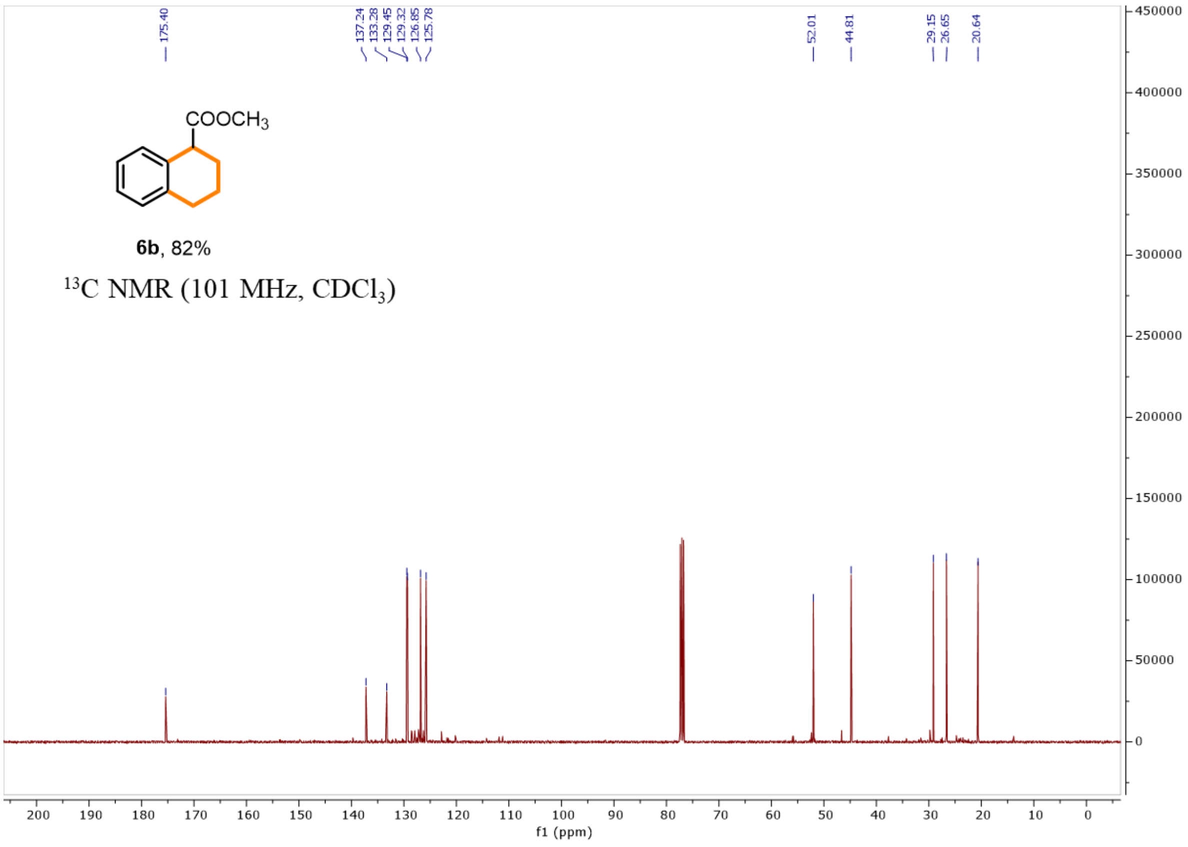
175.40
137.24
133.28
129.45
129.32
126.85
125.78
52.01
44.81
29.15
26.65
20.64
COOCH3
6b, 82%
13C NMR (101 MHz, CDCl3)
f1 (ppm)

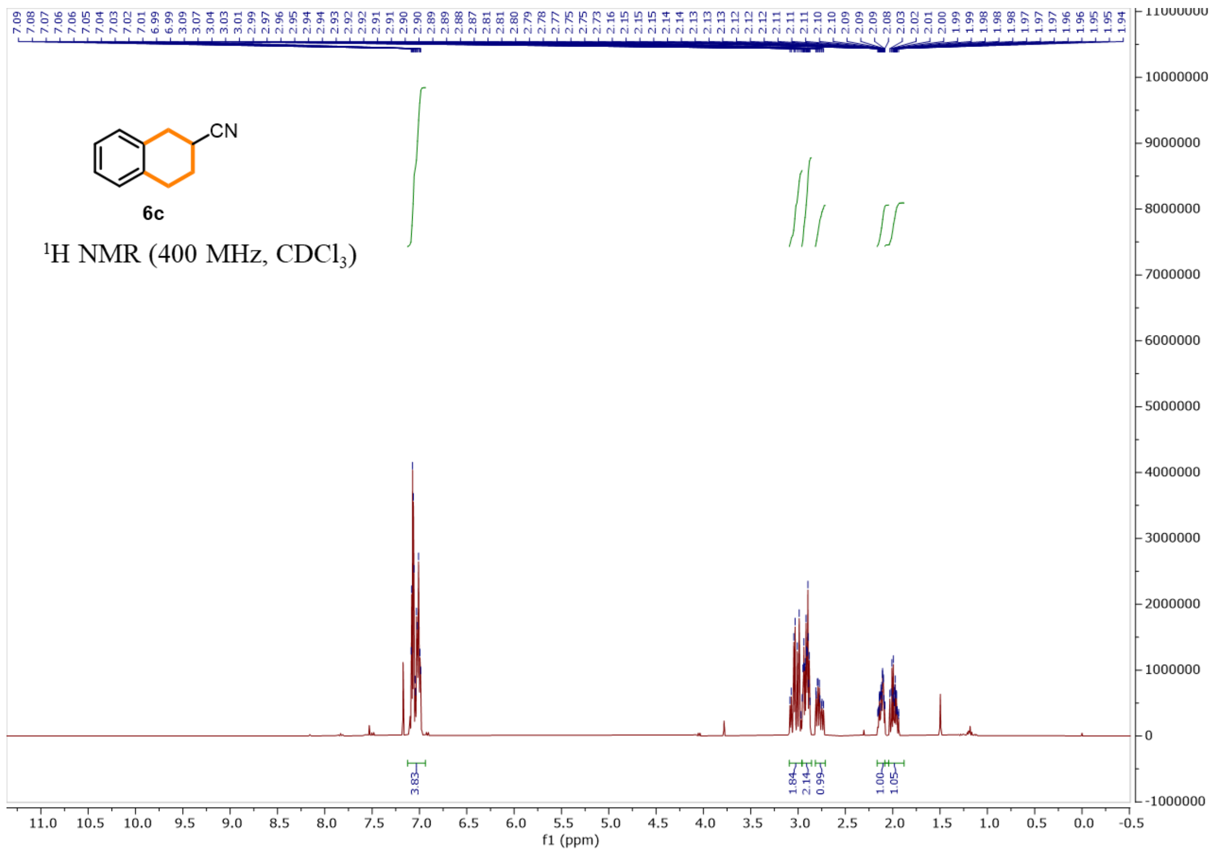
CN
6c
1H NMR (400 MHz, CDCl3)
3.83
1.84
2.14
0.99
1.00
1.05
f1 (ppm)

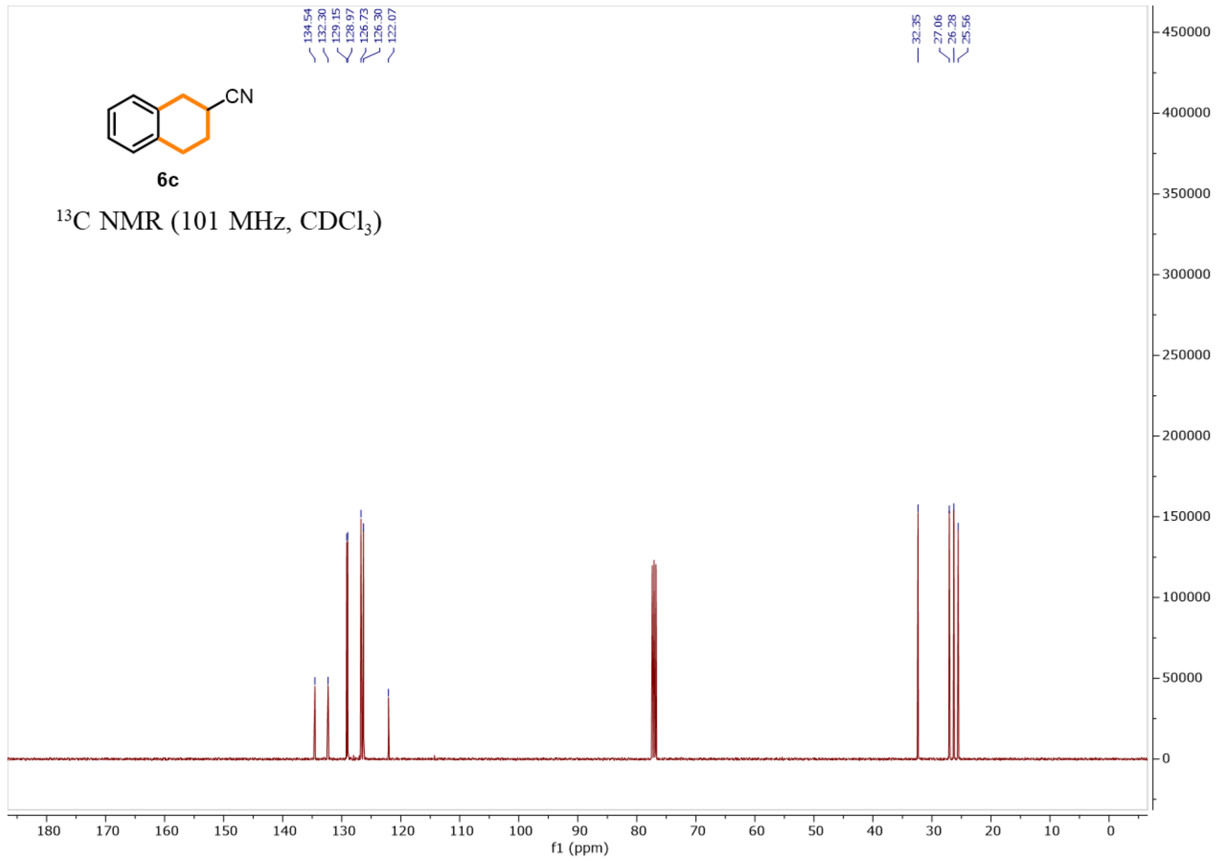
134.54
132.30
129.15
128.97
126.73
126.30
122.07
32.35
27.06
26.28
25.56
CN
6c
13C NMR (101 MHz, CDCl3)
f1 (ppm)

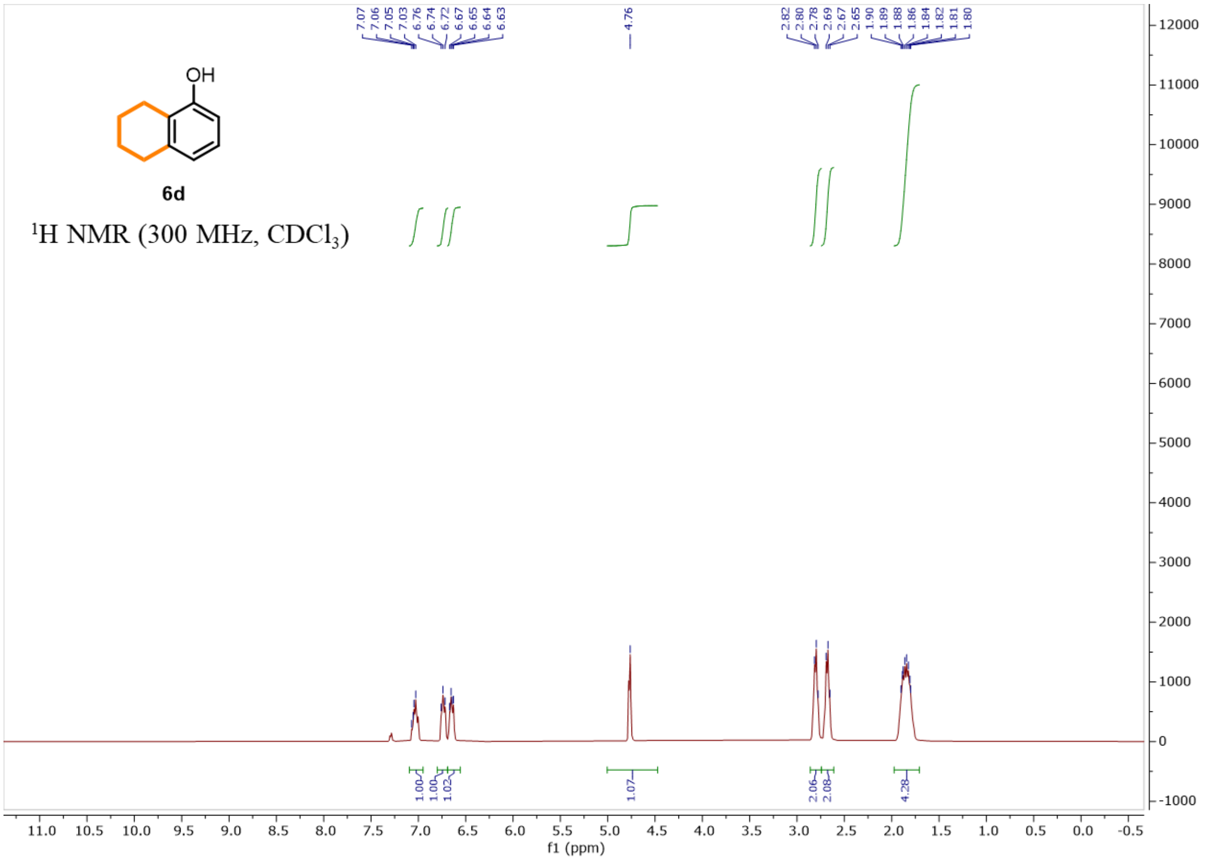

7.07 7.06 7.05 7.03 6.76 6.74 6.72 6.67 6.65 6.64 6.63
4.76
2.82 2.80 2.78 2.69 2.67 2.65 1.90 1.89 1.88 1.86 1.84 1.82 1.81 1.80
OH
6d
$^{1}H$ NMR (300 MHz, $CDCl_3$)
1.00 1.00 1.02 1.07 2.06 2.08 4.28
f1 (ppm)


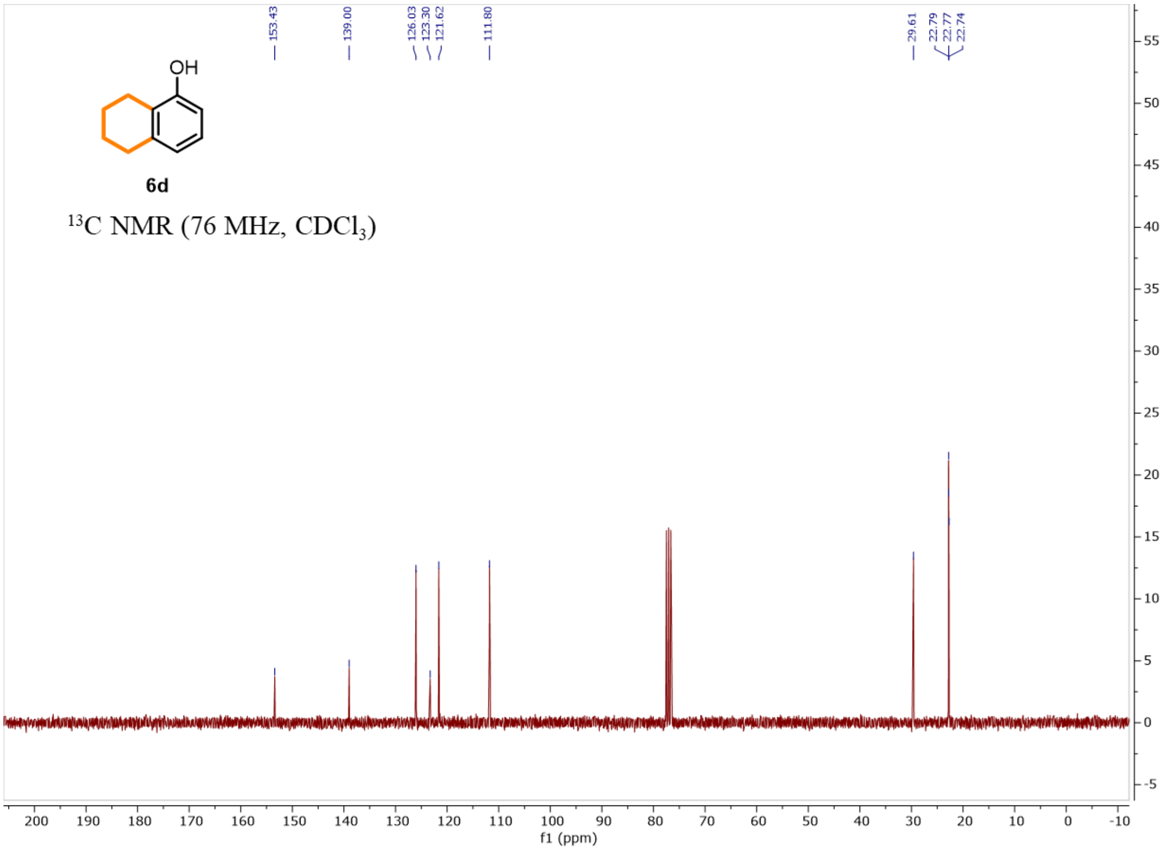

153.43
139.00
126.03 123.30 121.62
111.80
29.61 22.79 22.77 22.74
OH
6d
$^{13}C$ NMR (76 MHz, $CDCl_3$)
f1 (ppm)

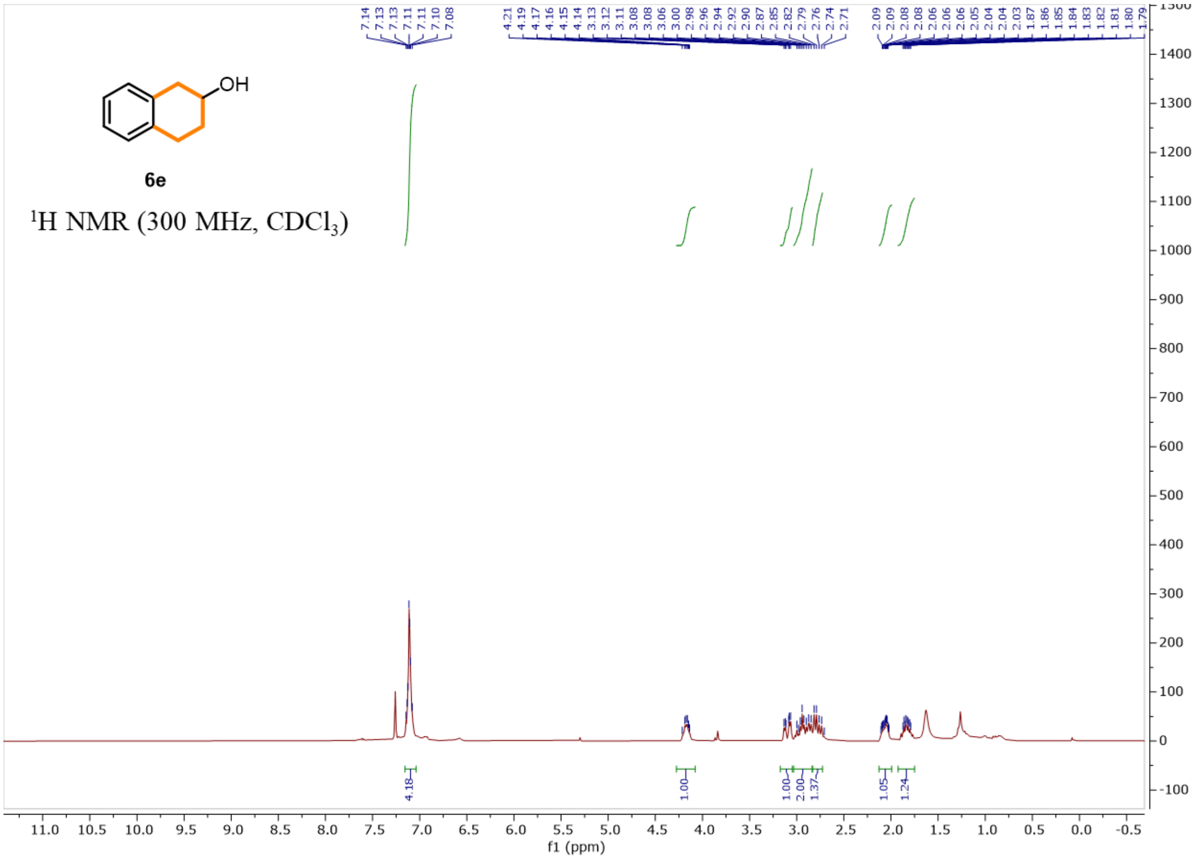
OH
6e
1H NMR (300 MHz, CDCl3)
f1 (ppm)

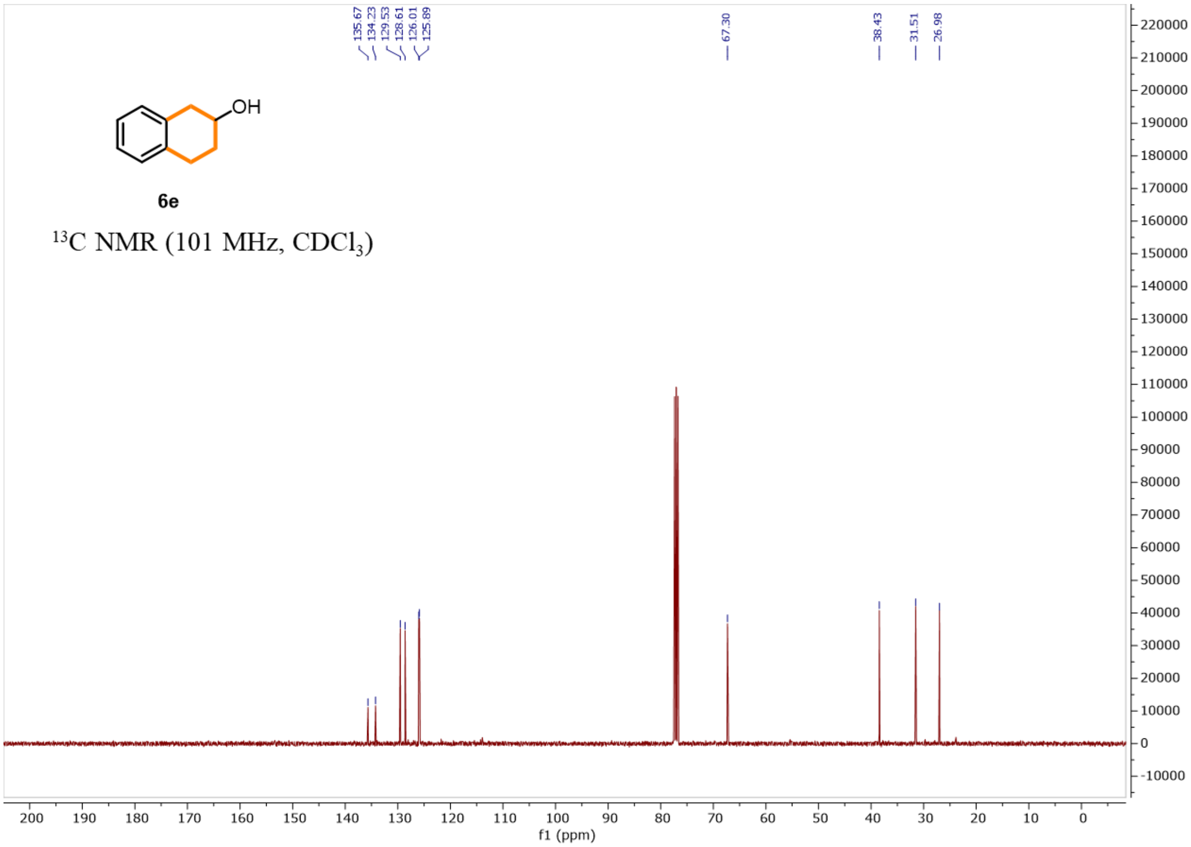
OH
6e
13C NMR (101 MHz, CDCl3)
135.67
134.23
129.53
128.61
126.01
125.89
67.30
38.43
31.51
26.98
f1 (ppm)

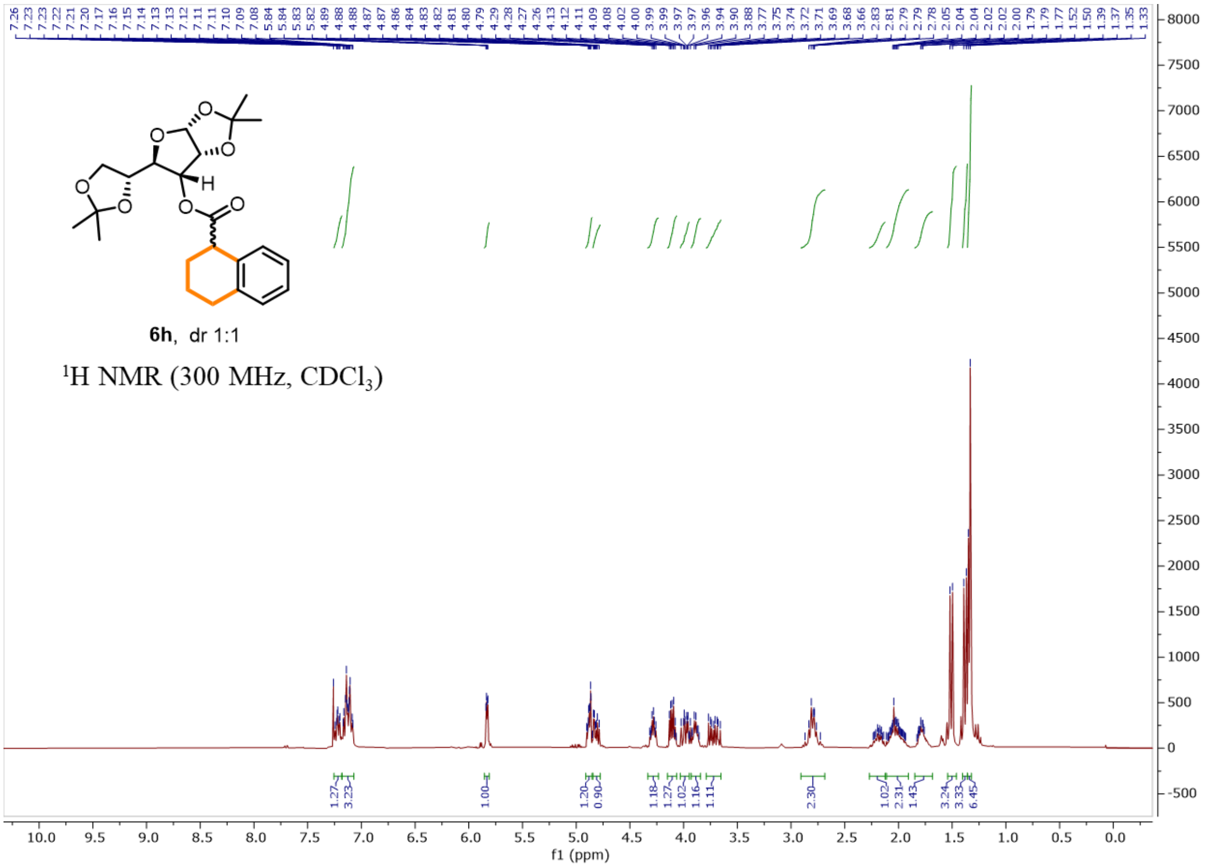
7.26 7.23 7.23 7.22 7.21 7.20 7.17 7.16 7.15 7.14 7.13 7.13 7.12 7.11 7.11 7.10 7.09 7.08 5.84 5.84 5.83 5.82 4.89 4.88 4.88 4.87 4.87 4.86 4.84 4.83 4.82 4.81 4.80 4.79 4.29 4.28 4.27 4.26 4.13 4.12 4.11 4.09 4.08 4.02 4.00 3.99 3.99 3.97 3.97 3.96 3.94 3.90 3.88 3.77 3.75 3.74 3.72 3.71 3.69 3.68 3.66 2.83 2.81 2.79 2.78 2.05 2.04 2.04 2.04 2.02 2.02 2.00 1.79 1.77 1.52 1.50 1.39 1.37 1.35 1.33
H
6h, dr 1:1
1H NMR (300 MHz, CDCl3)
1.27 3.23 1.00 1.20 0.90 1.18 1.27 1.02 1.16 1.11 2.30 1.02 2.31 1.43 3.24 3.33 6.45
f1 (ppm)

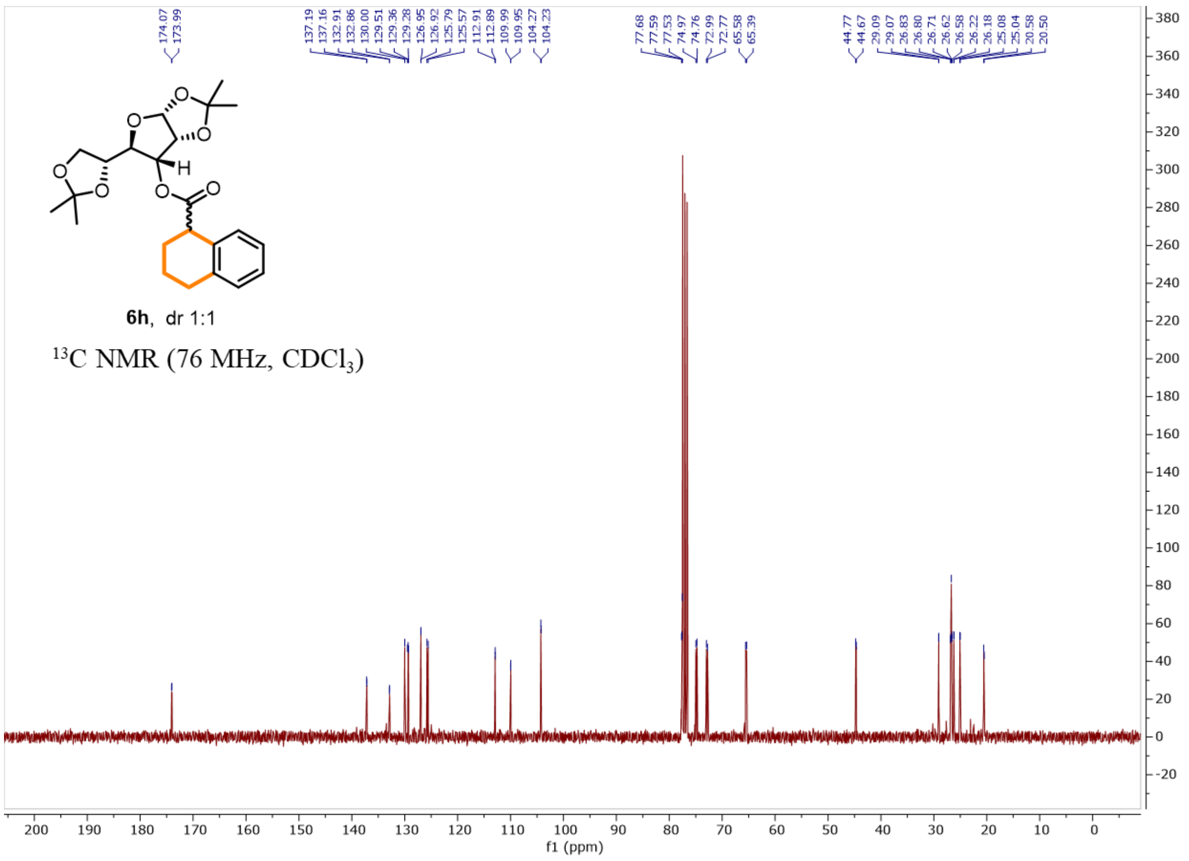
174.07 173.99 137.19 137.16 132.91 132.86 130.00 129.51 129.36 129.28 126.95 126.92 125.79 125.57 112.91 112.89 109.99 109.95 104.27 104.23 77.68 77.59 77.53 74.97 74.76 72.99 72.77 65.58 65.39 44.77 44.67 29.09 29.07 26.83 26.80 26.71 26.62 26.58 26.22 26.18 25.08 25.04 20.58 20.50
H
6h, dr 1:1
13C NMR (76 MHz, CDCl3)
f1 (ppm)

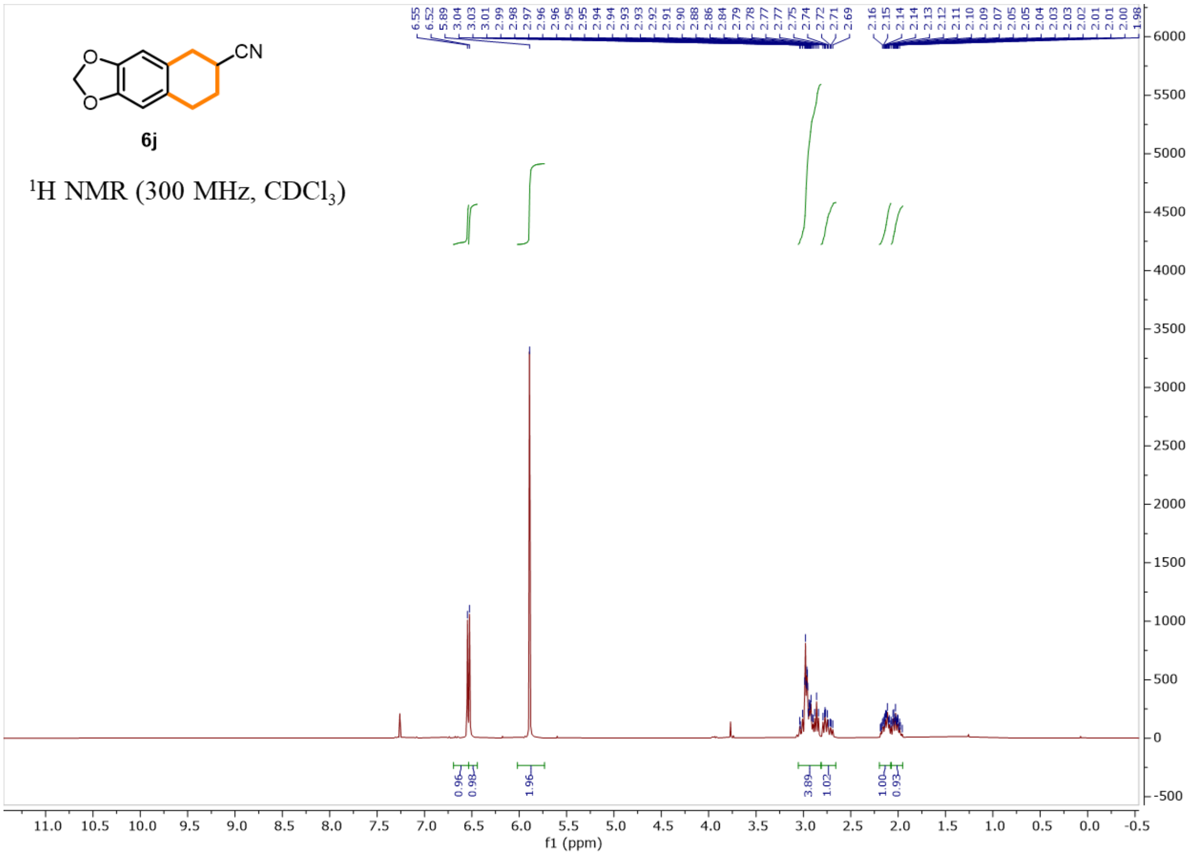

6.55
6.52
5.89
3.04 3.03 3.01 2.99 2.98 2.97 2.96 2.96 2.95 2.95 2.94 2.94 2.93 2.93 2.92 2.91 2.90 2.88 2.86 2.84 2.79 2.78 2.77 2.77 2.75 2.74 2.72 2.71 2.69
2.16 2.15 2.14 2.14 2.13 2.12 2.11 2.10 2.09 2.07 2.05 2.05 2.04 2.03 2.03 2.02 2.01 2.01 2.00 1.98
CN
6j
1H NMR (300 MHz, CDCl3)
0.96
0.98
1.96
3.89
1.02
1.00
0.93
f1 (ppm)


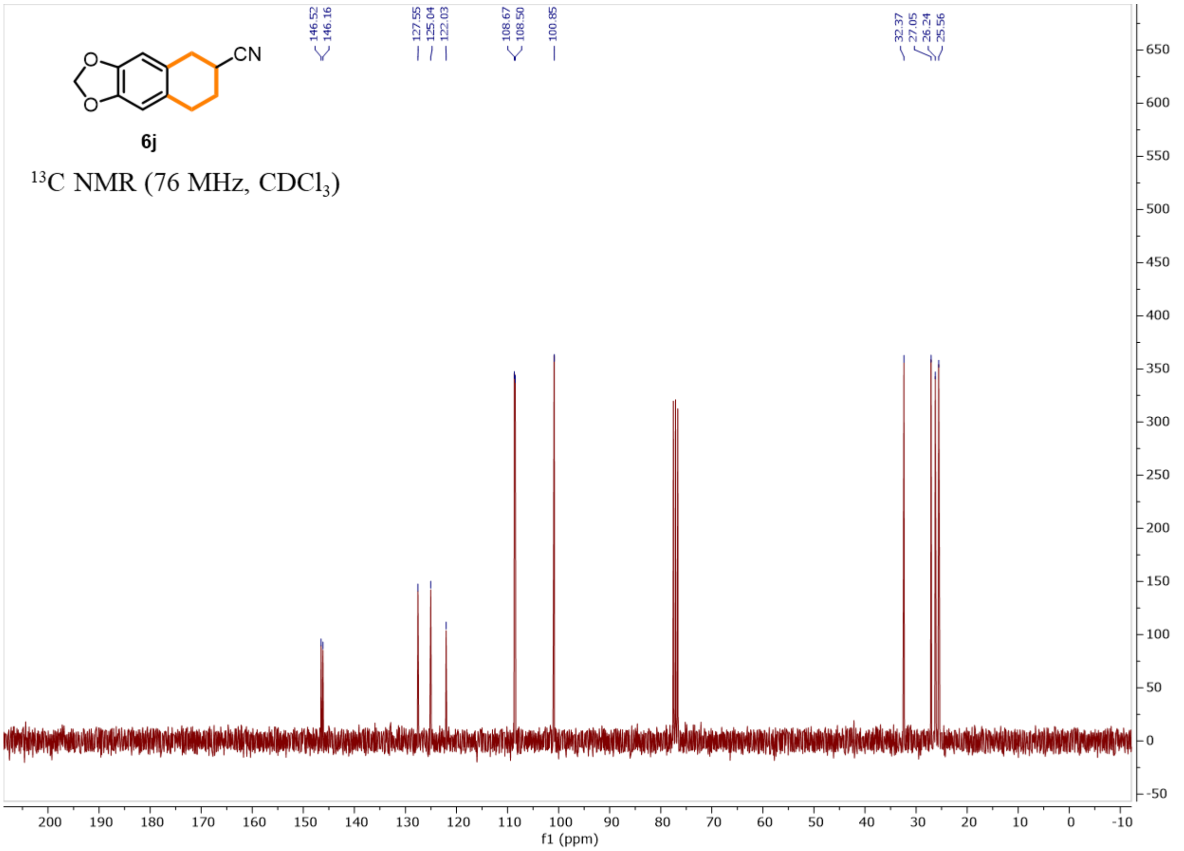

146.52
146.16
127.55
125.04
122.03
108.67
108.50
100.85
32.37
27.05
26.24
25.56
CN
6j
13C NMR (76 MHz, CDCl3)
f1 (ppm)

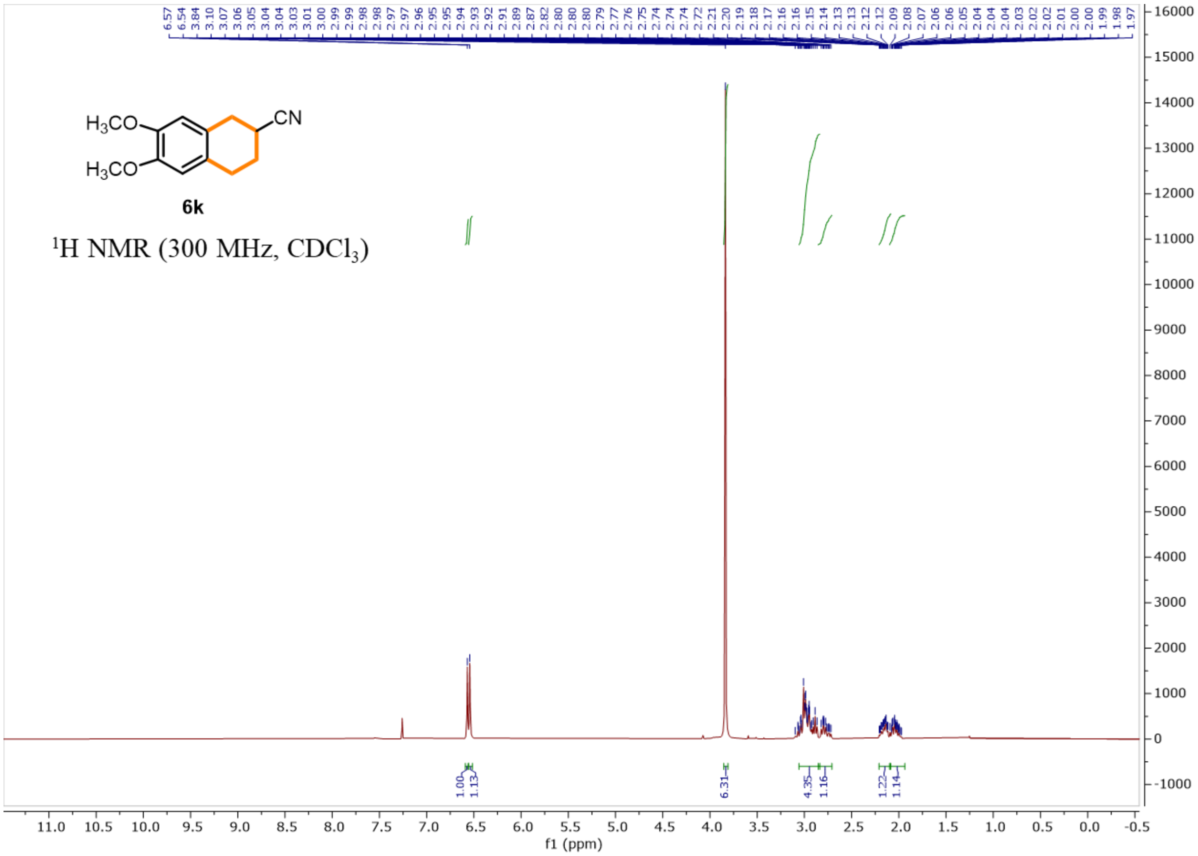
H3CO
H3CO
CN
6k
1H NMR (300 MHz, CDCl3)
f1 (ppm)

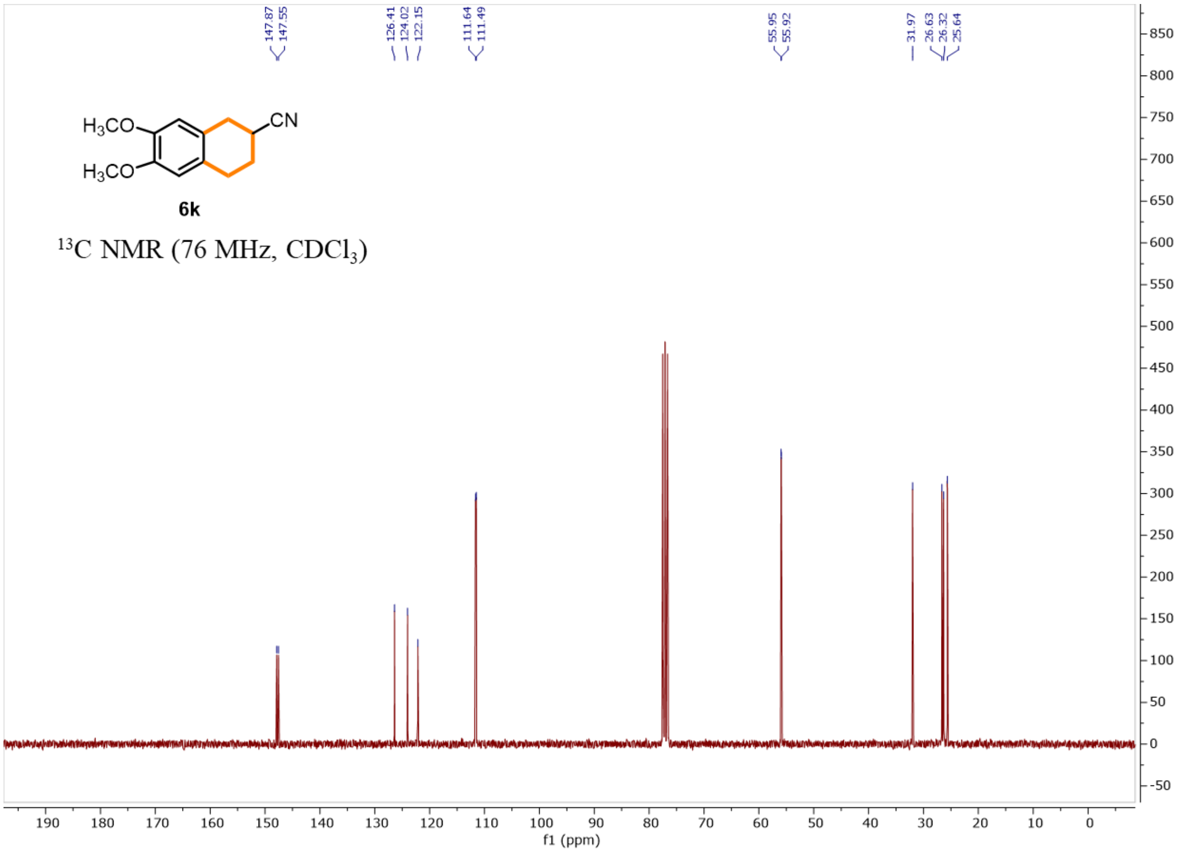
H3CO
H3CO
CN
6k
13C NMR (76 MHz, CDCl3)
147.87
147.55
126.41
124.02
122.15
111.64
111.49
55.95
55.92
31.97
26.63
26.32
25.64
f1 (ppm)

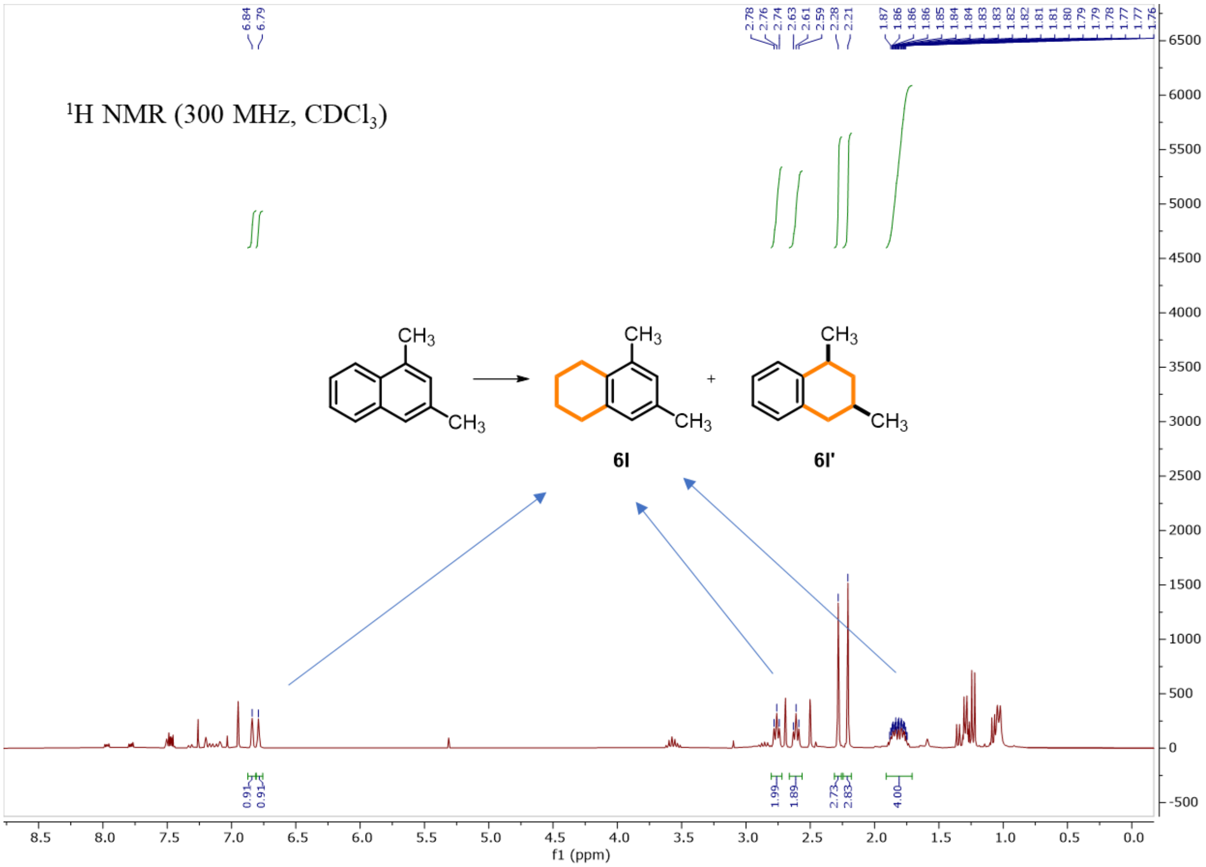

¹H NMR (300 MHz, CDCl3)
6.84
6.79
2.78
2.76
2.74
2.63
2.61
2.59
2.28
2.21
1.87
1.86
1.86
1.86
1.85
1.84
1.84
1.83
1.83
1.82
1.82
1.81
1.81
1.80
1.79
1.79
1.78
1.77
1.77
1.76
CH3
CH3
6l
6l'
0.91
0.91
1.99
1.89
2.73
2.83
4.00
f1 (ppm)

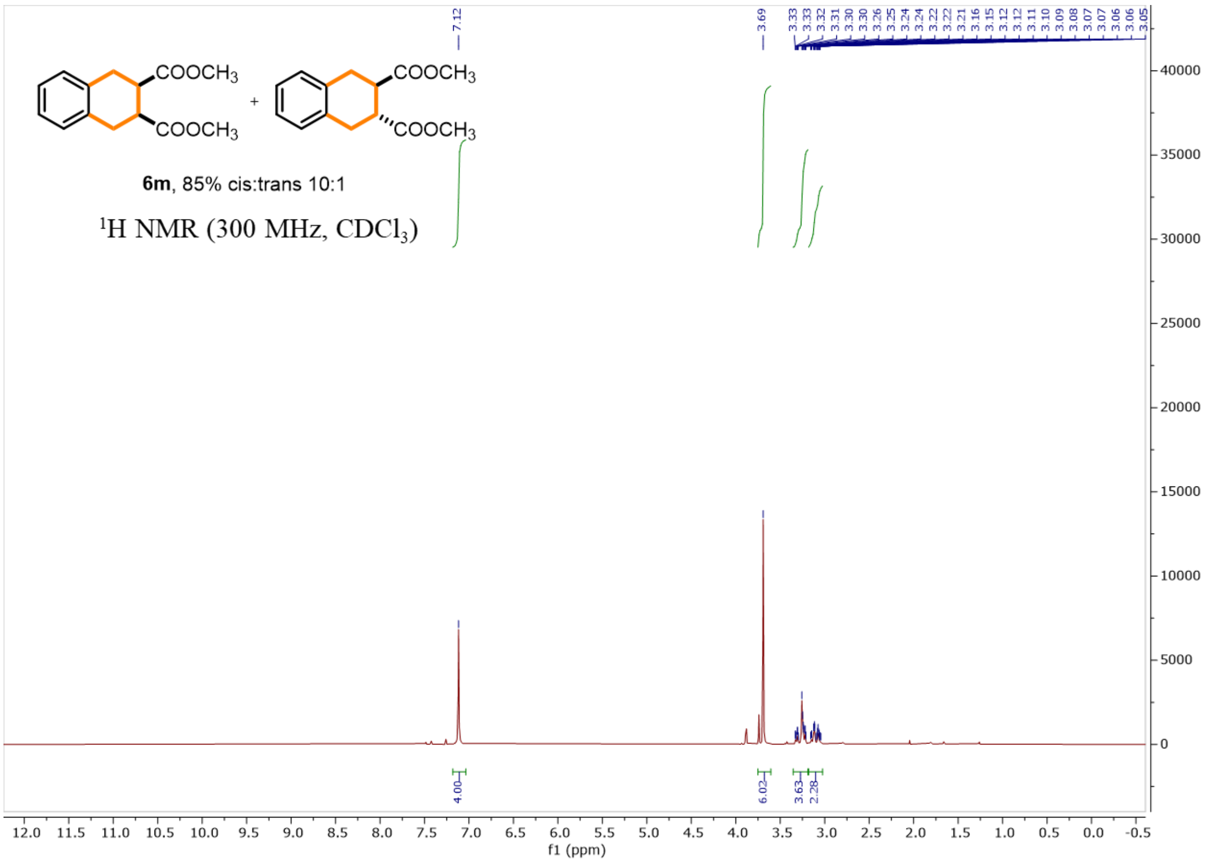
COOCH3
COOCH3
+
COOCH3
COOCH3
6m, 85% cis:trans 10:1
1H NMR (300 MHz, CDCl3)
7.12
3.69
3.33
3.33
3.32
3.31
3.30
3.30
3.26
3.25
3.24
3.24
3.22
3.22
3.21
3.16
3.15
3.12
3.12
3.11
3.10
3.09
3.08
3.07
3.06
3.05
4.00
6.02
3.63
2.28
f1 (ppm)

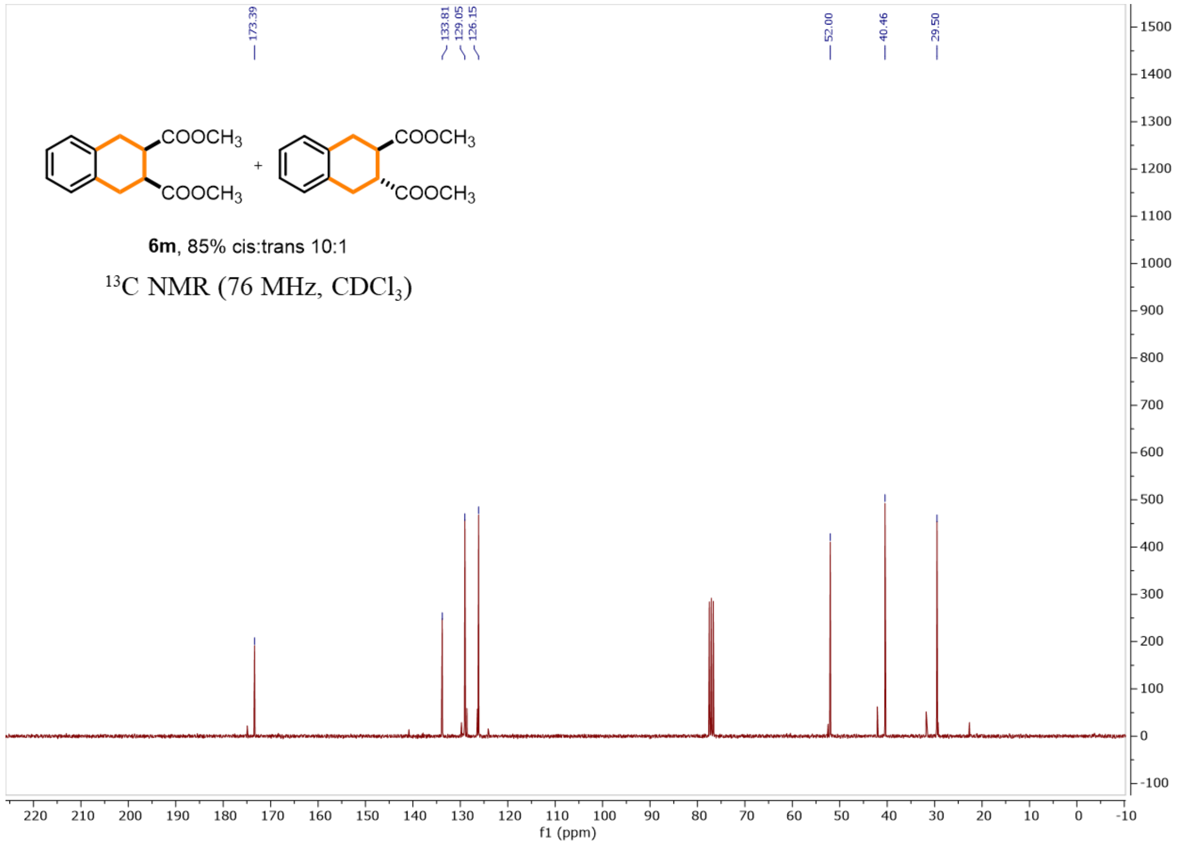
173.39
133.81
129.05
126.15
52.00
40.46
29.50
COOCH3
COOCH3
+
COOCH3
COOCH3
6m, 85% cis:trans 10:1
13C NMR (76 MHz, CDCl3)
f1 (ppm)

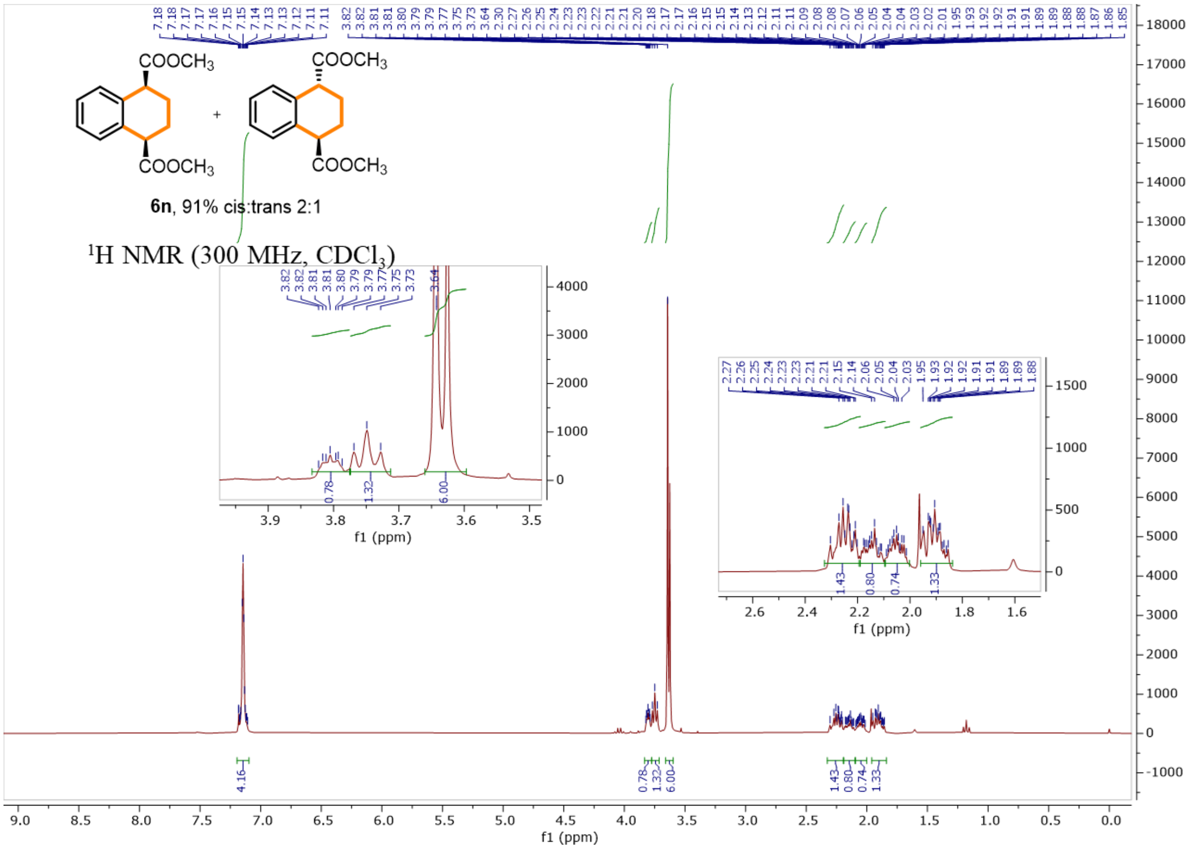

COOCH3
COOCH3
COOCH3
COOCH3
+
6n, 91% cis:trans 2:1
1H NMR (300 MHz, CDCl3)
f1 (ppm)


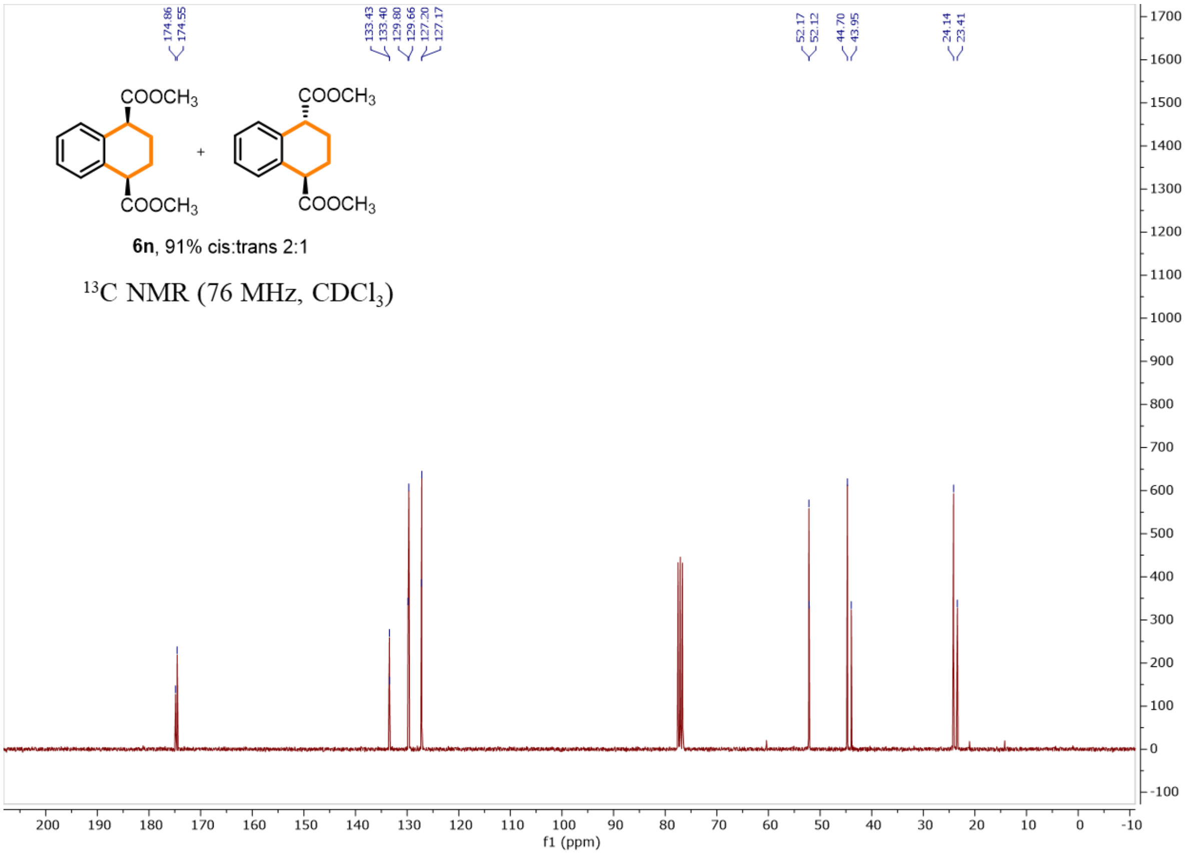

174.86
174.55
133.43
133.40
129.80
129.66
127.20
127.17
52.17
52.12
44.70
43.95
24.14
23.41
COOCH3
COOCH3
COOCH3
COOCH3
+
6n, 91% cis:trans 2:1
13C NMR (76 MHz, CDCl3)
f1 (ppm)

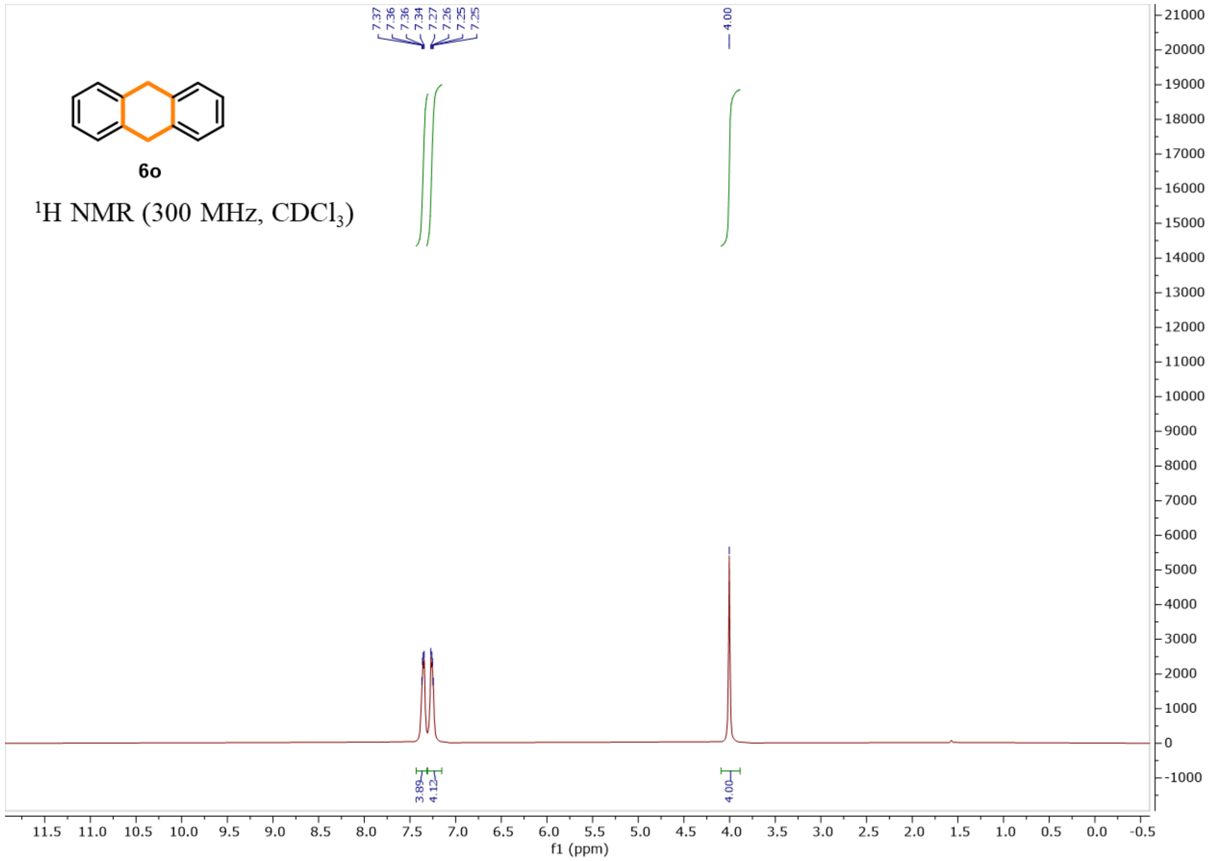

7.37
7.36
7.36
7.34
7.27
7.26
7.25
7.25
4.00
6o
¹H NMR (300 MHz, CDCl₃)
3.89
4.12
4.00
f1 (ppm)


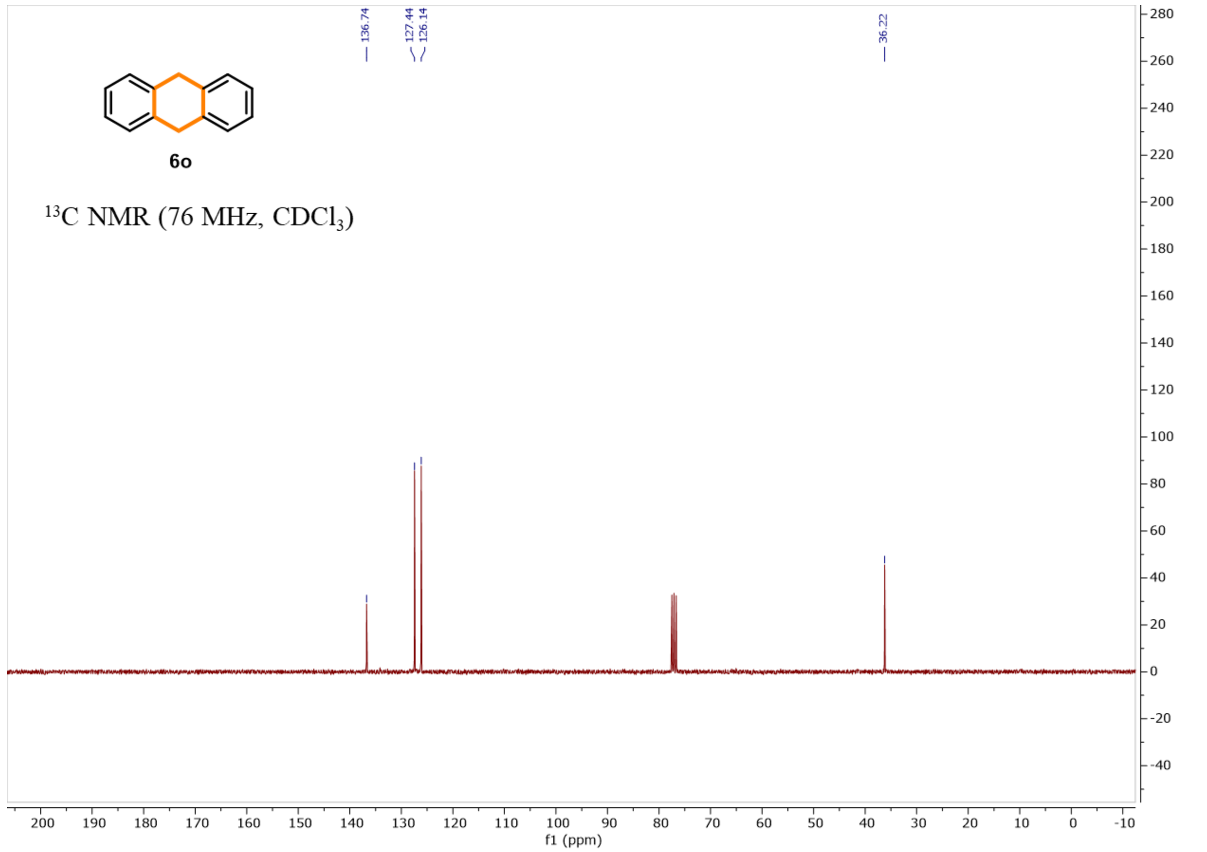

136.74
127.44
126.14
36.22
6o
¹³C NMR (76 MHz, CDCl₃)
f1 (ppm)

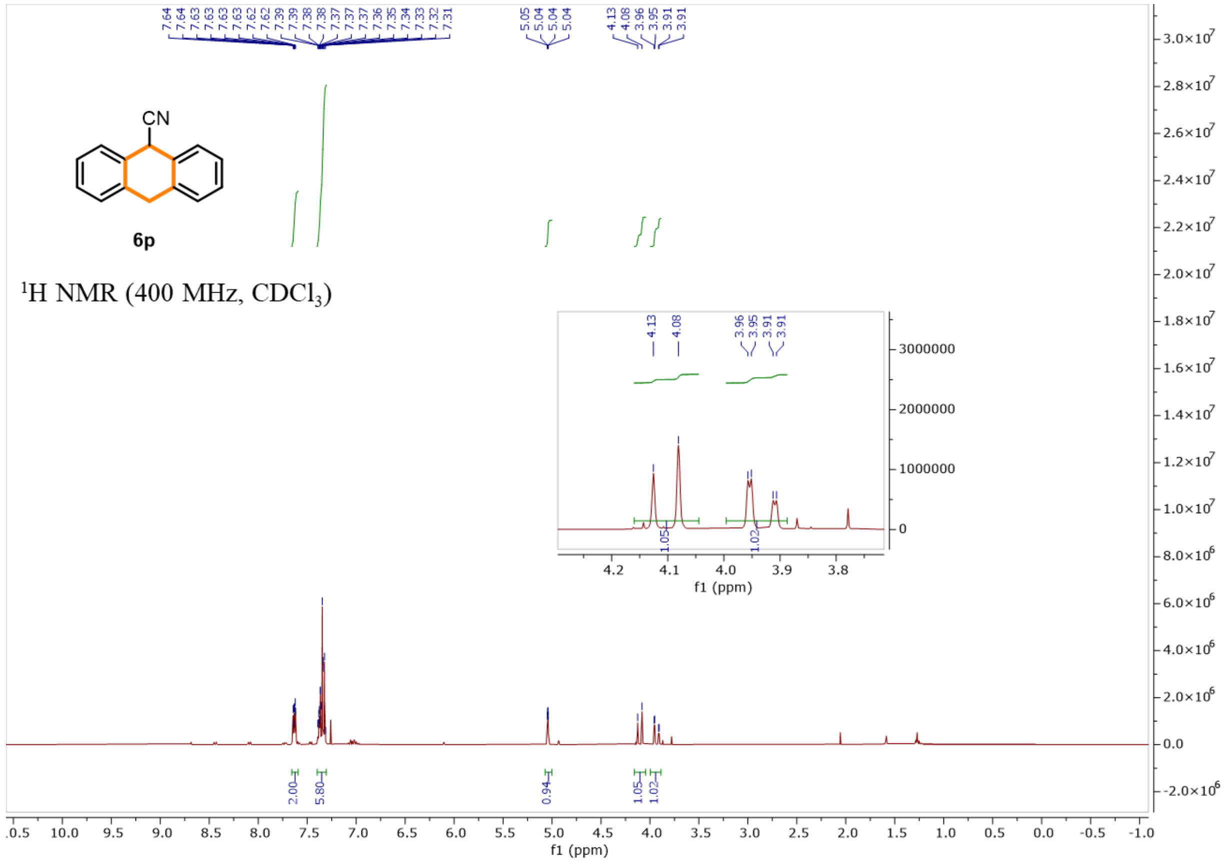
CN
6p
1H NMR (400 MHz, CDCl3)
f1 (ppm)

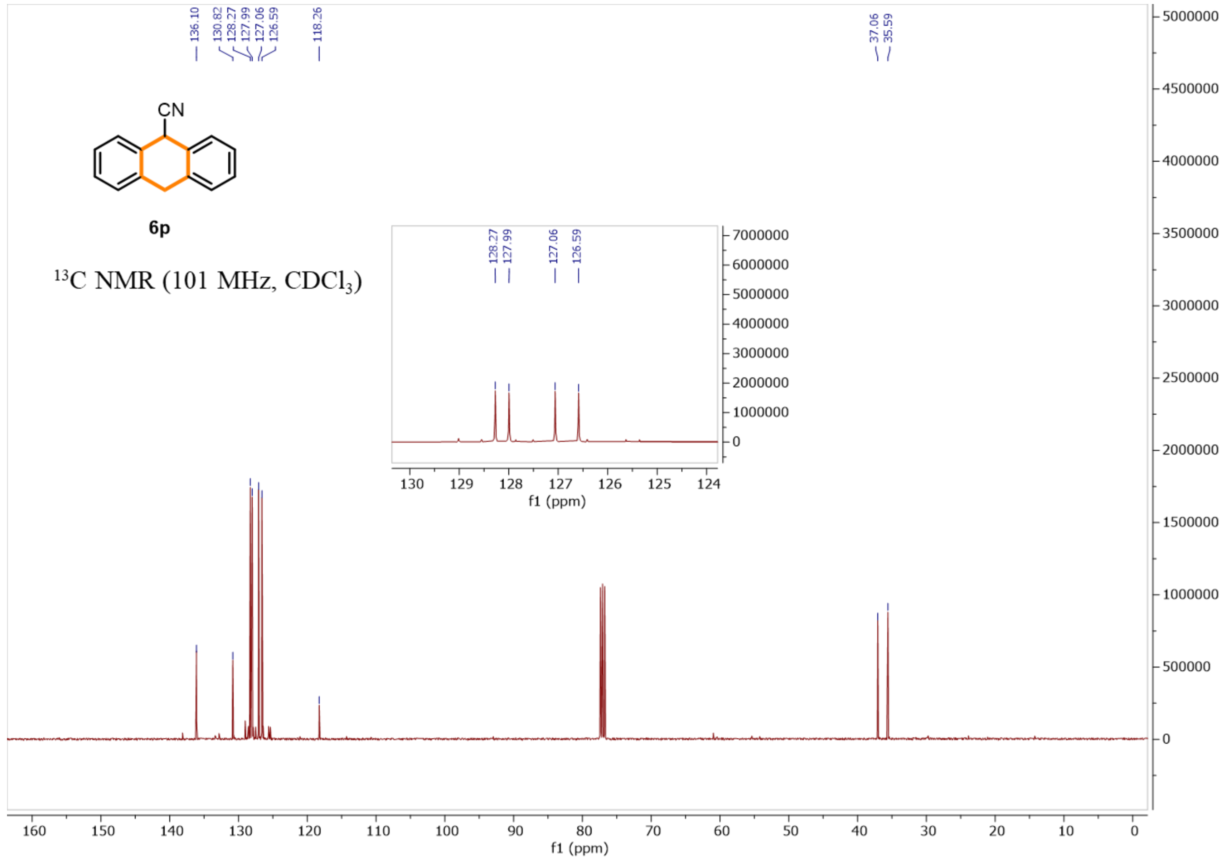
CN
6p
13C NMR (101 MHz, CDCl3)
f1 (ppm)

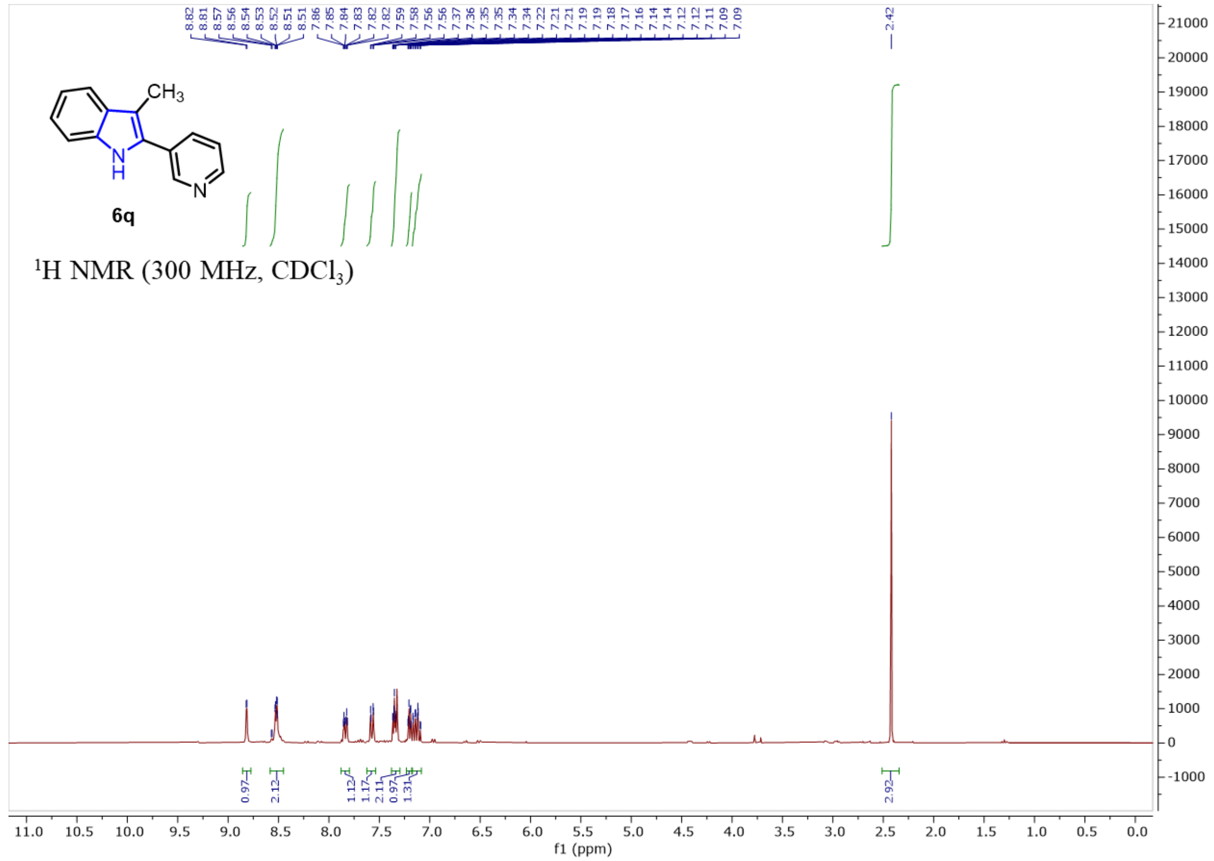

6q
¹H NMR (300 MHz, CDCl3)
f1 (ppm)


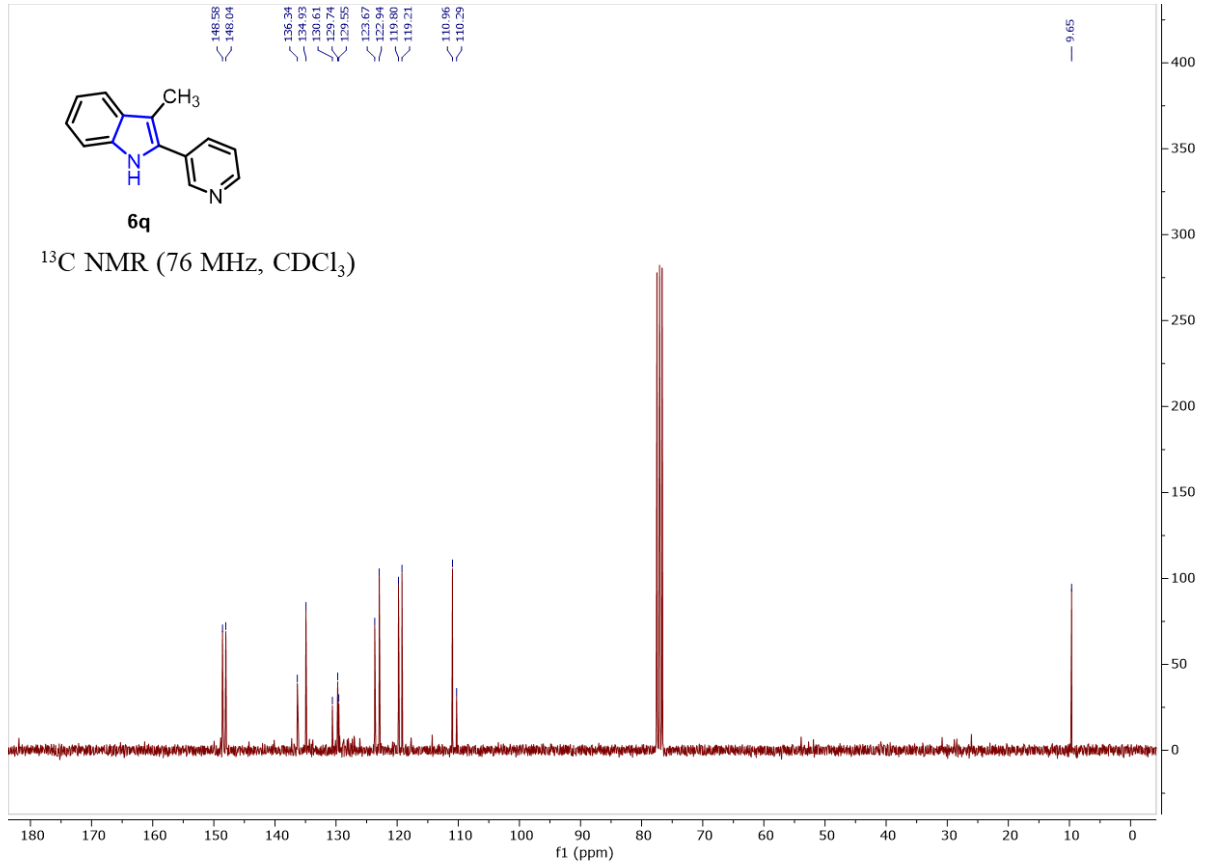

6q
¹³C NMR (76 MHz, CDCl3)
f1 (ppm)

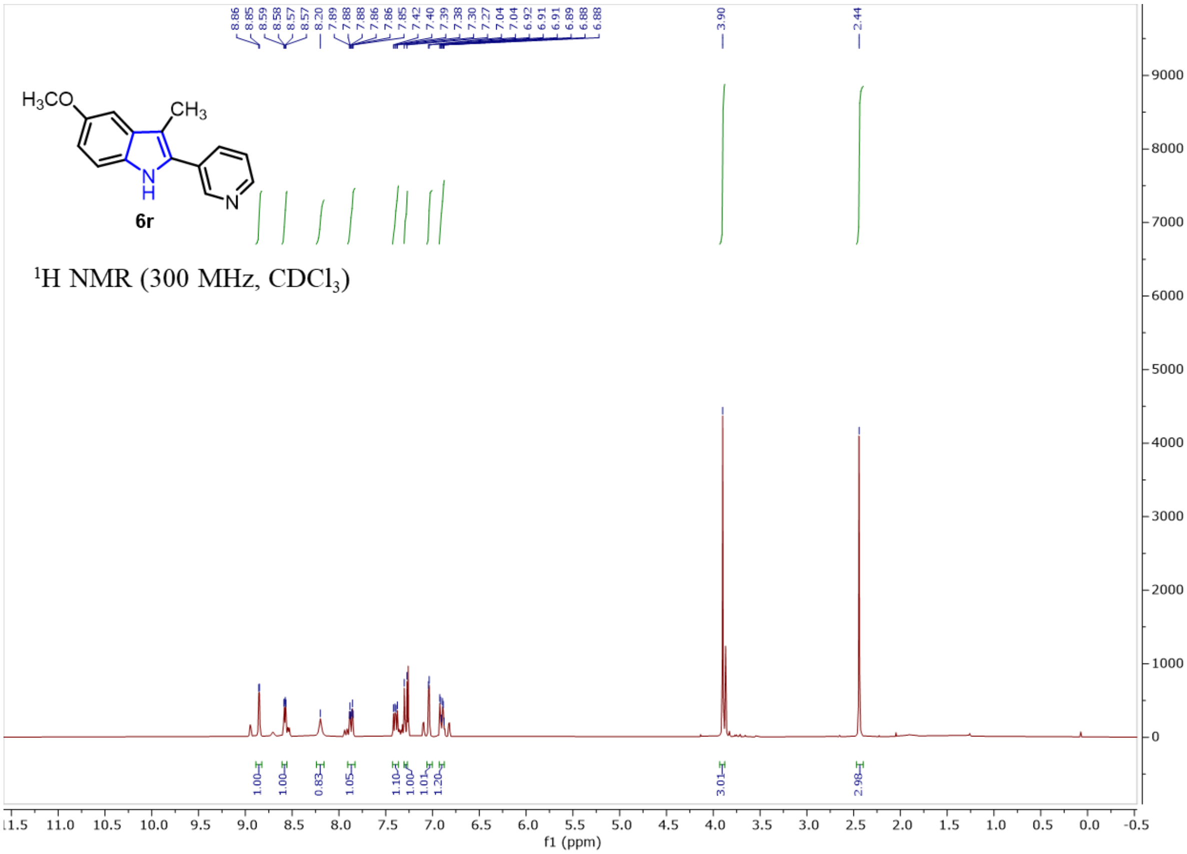


$^{1}H$ NMR (300 MHz, $CDCl_3$)

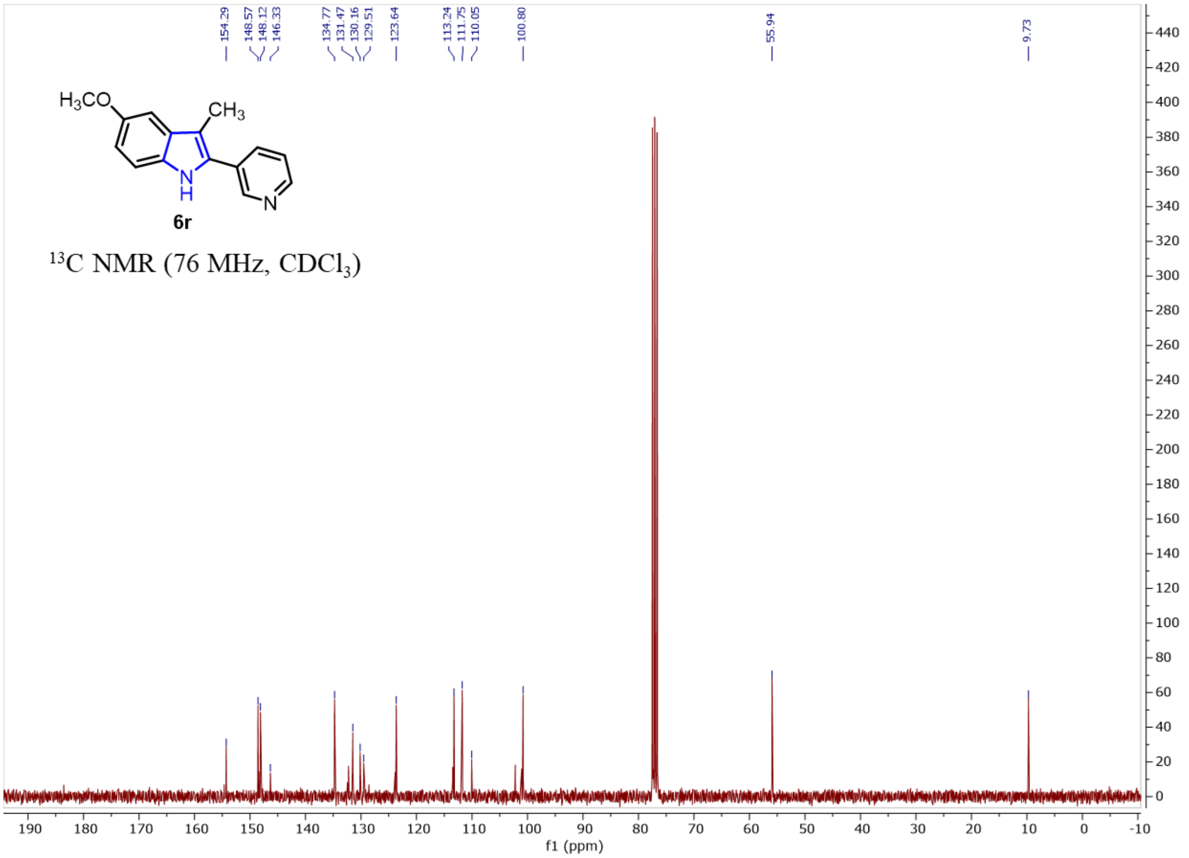


$^{13}C$ NMR (76 MHz, $CDCl_3$)

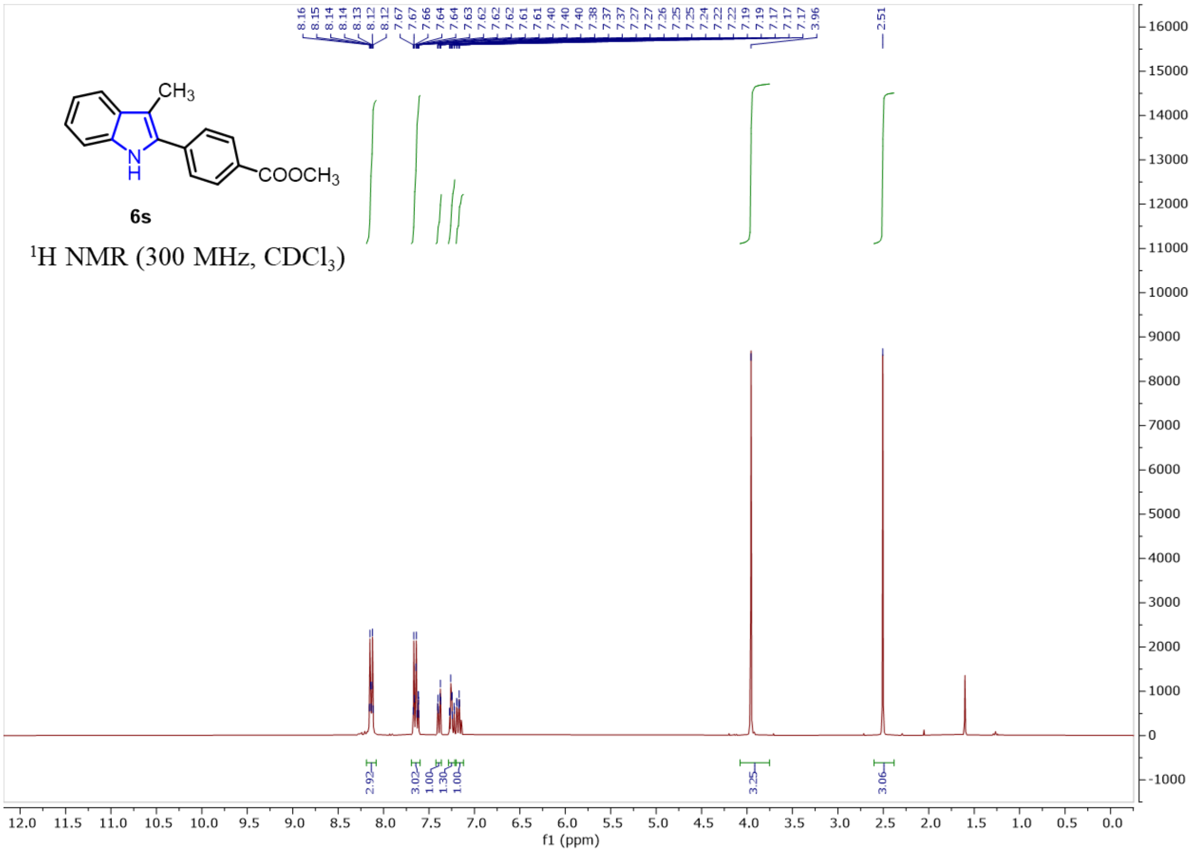
CH3
N
H
COOCH3
6s
1H NMR (300 MHz, CDCl3)
f1 (ppm)

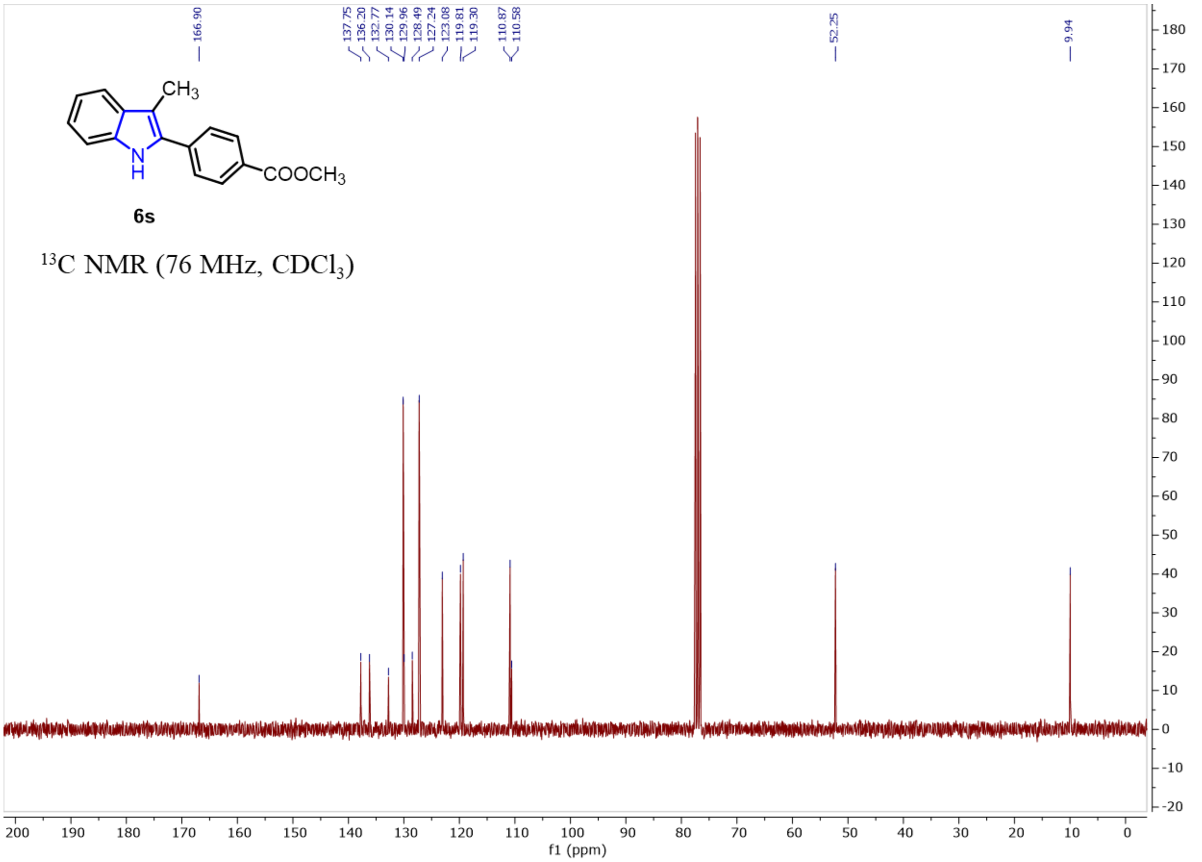
CH3
N
H
COOCH3
6s
13C NMR (76 MHz, CDCl3)
f1 (ppm)

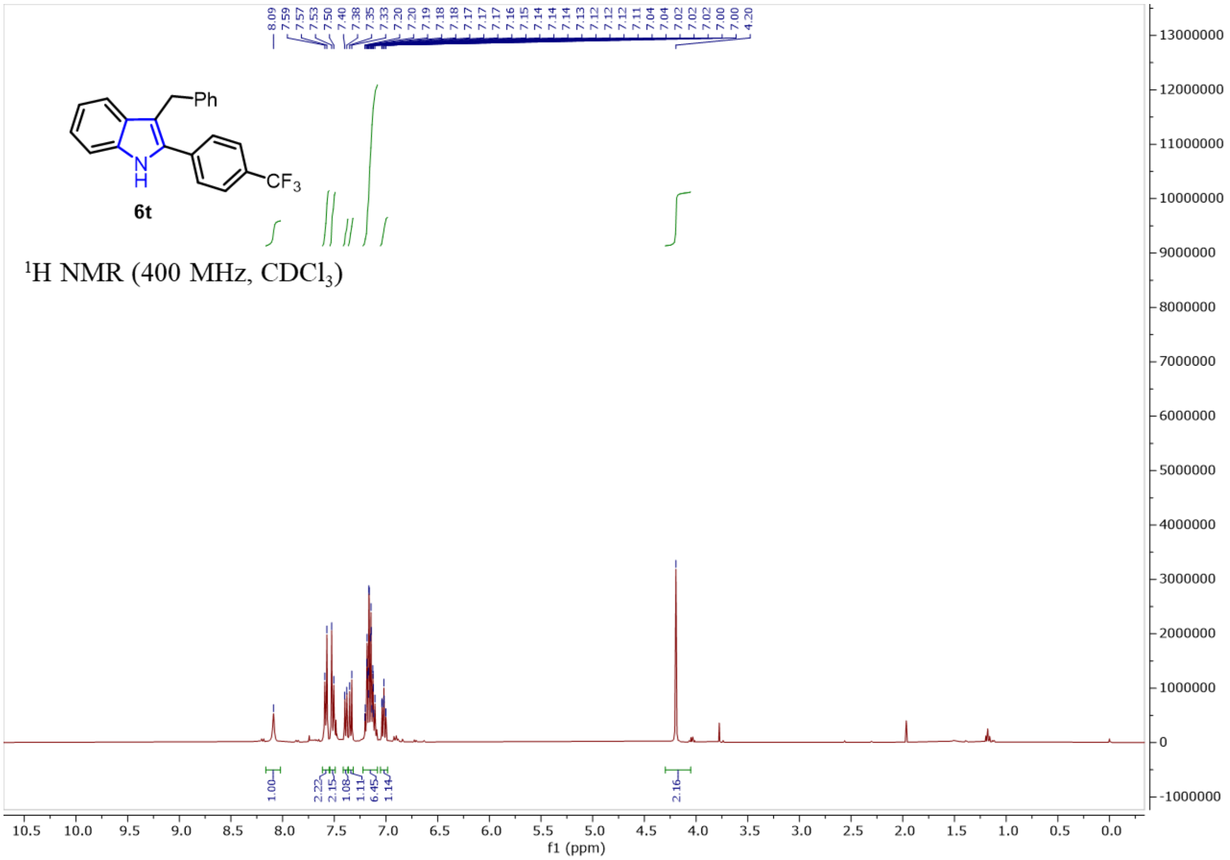


$^{1}$H NMR (400 MHz, $CDCl_3$)

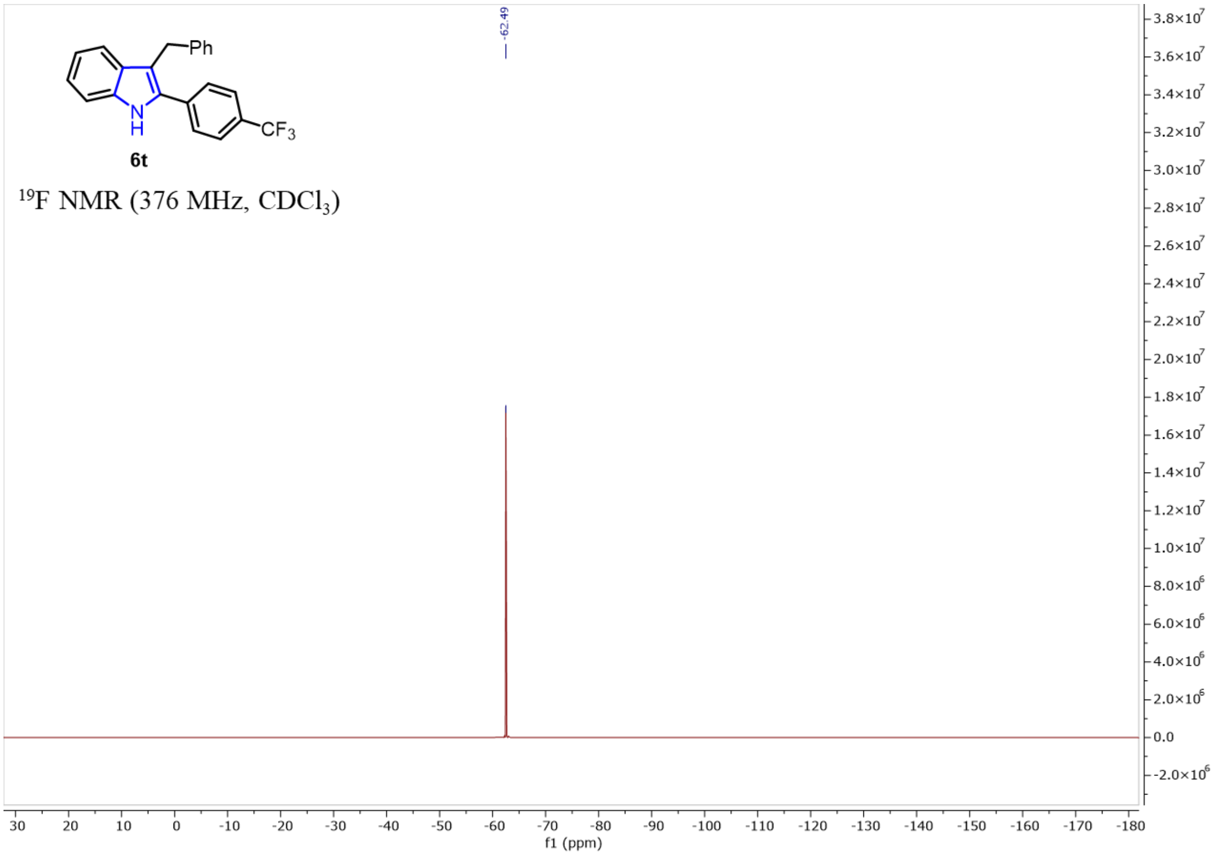


$^{19}$F NMR (376 MHz, $CDCl_3$)

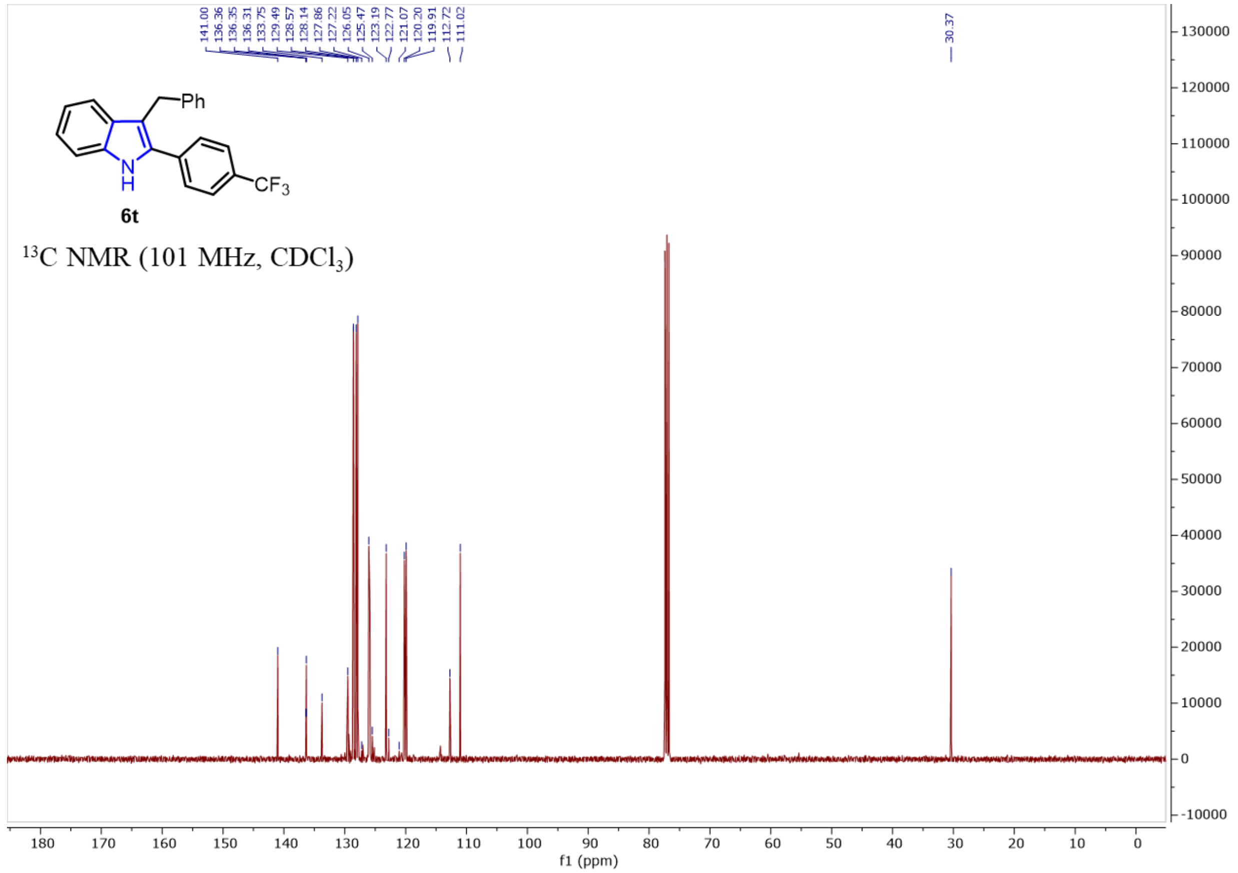

141.00
136.36
136.35
136.31
133.75
129.49
128.57
128.14
127.86
127.22
126.05
125.47
123.19
122.77
121.07
120.20
119.91
112.72
111.02
30.37
Ph
N
H
CF3
6t
13C NMR (101 MHz, CDCl3)
f1 (ppm)

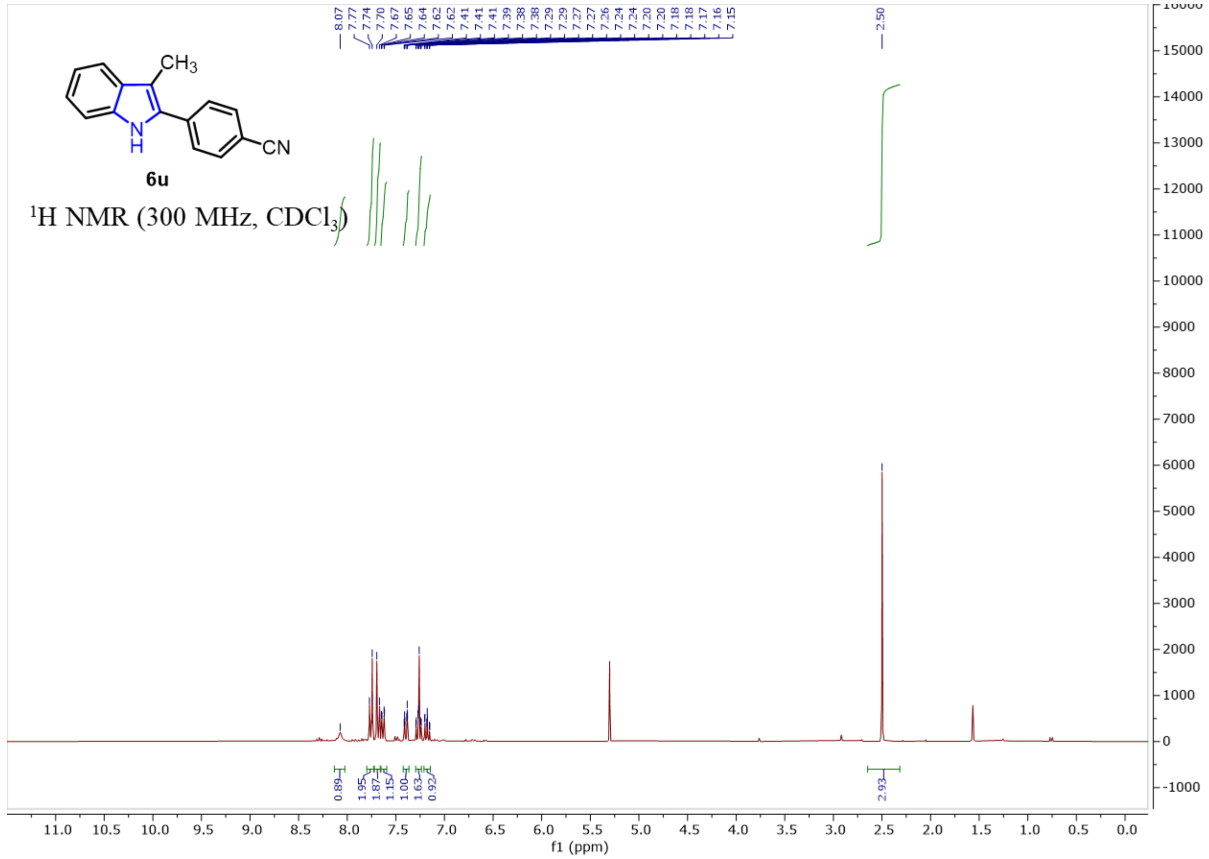
8.07
7.77
7.74
7.70
7.67
7.65
7.64
7.62
7.62
7.41
7.41
7.39
7.38
7.38
7.29
7.29
7.27
7.27
7.26
7.24
7.24
7.20
7.20
7.18
7.17
7.16
7.15
2.50
CH3
N
H
CN
6u
1H NMR (300 MHz, CDCl3)
0.89
1.95
1.87
1.15
1.00
1.63
0.92
2.93
f1 (ppm)

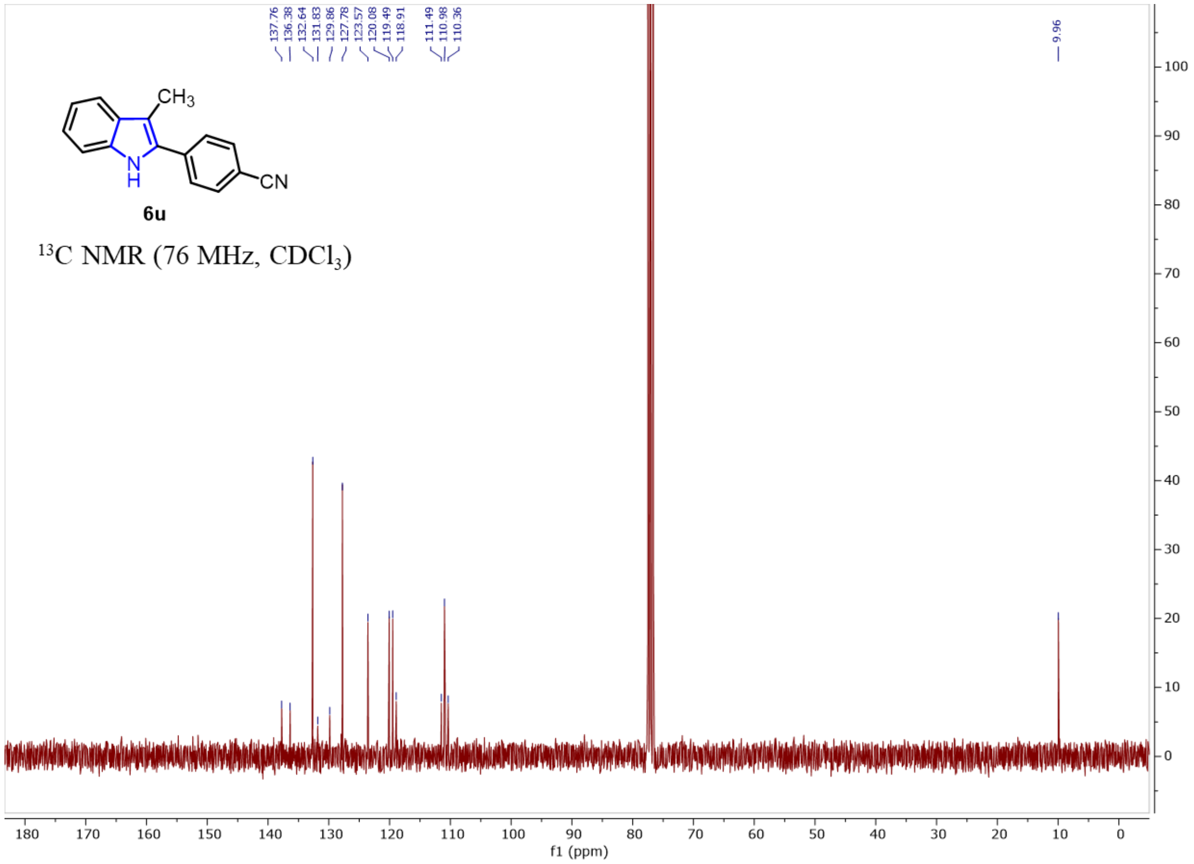
137.76
136.38
132.64
131.83
129.86
127.78
123.57
120.08
119.49
118.91
111.49
110.98
110.36
9.96
CH3
N
H
CN
6u
13C NMR (76 MHz, CDCl3)
f1 (ppm)

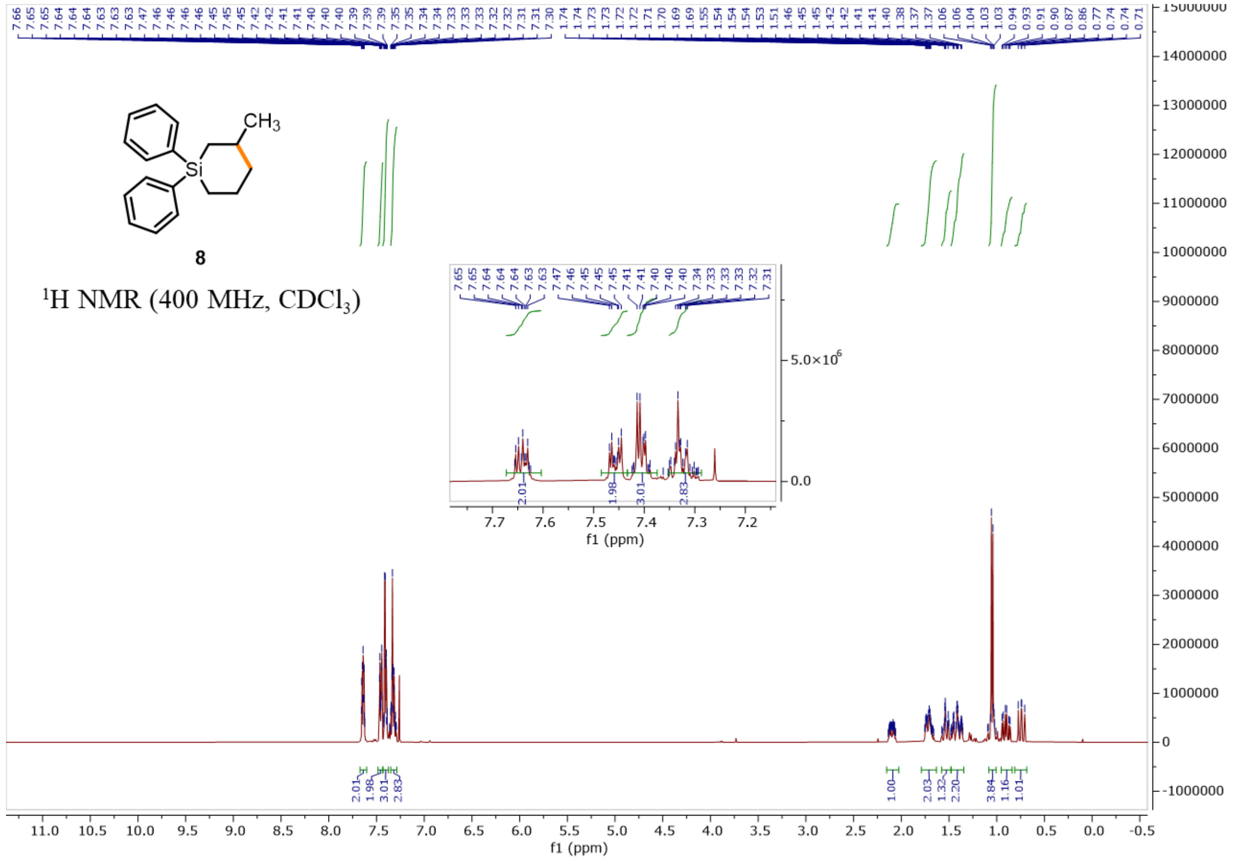

CH3
Si
8
1H NMR (400 MHz, CDCl3)
f1 (ppm)


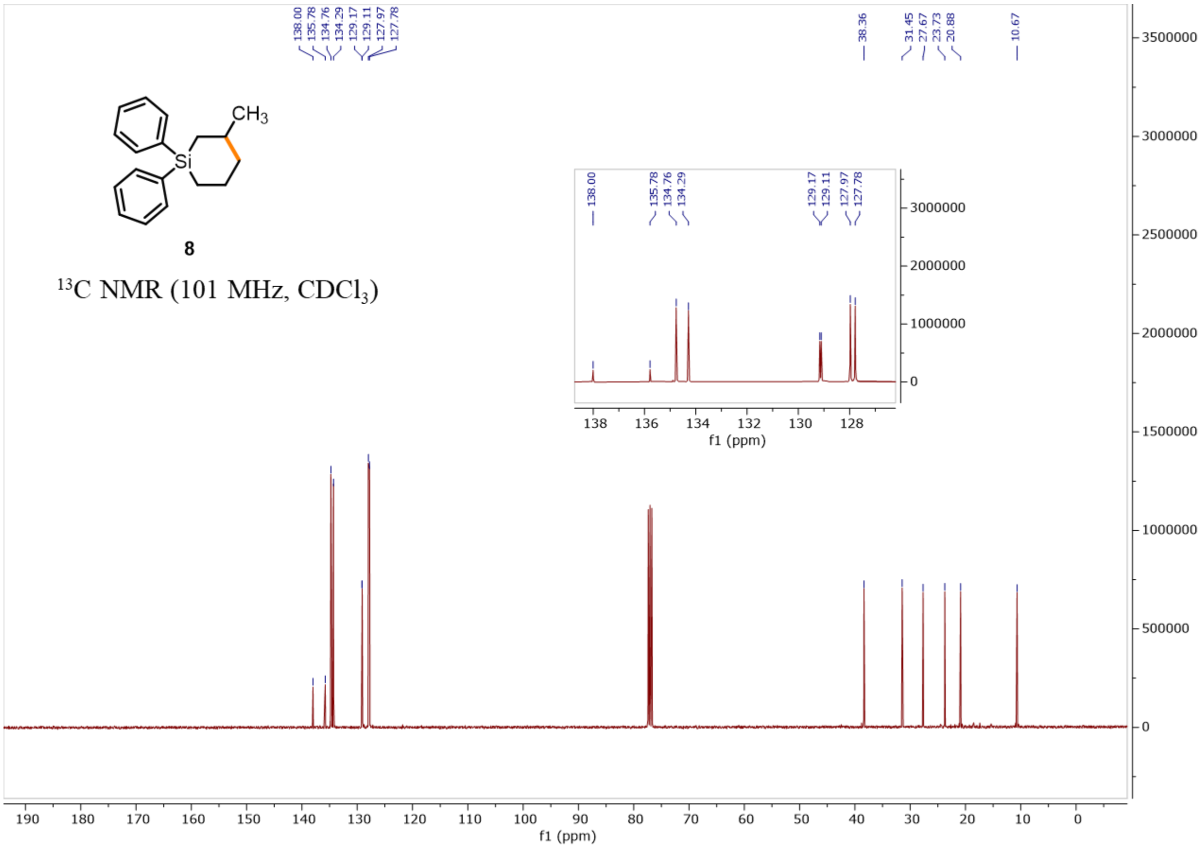

CH3
Si
8
13C NMR (101 MHz, CDCl3)
138.00
135.78
134.76
134.29
129.17
129.11
127.97
127.78
38.36
31.45
27.67
23.73
20.88
10.67
f1 (ppm)

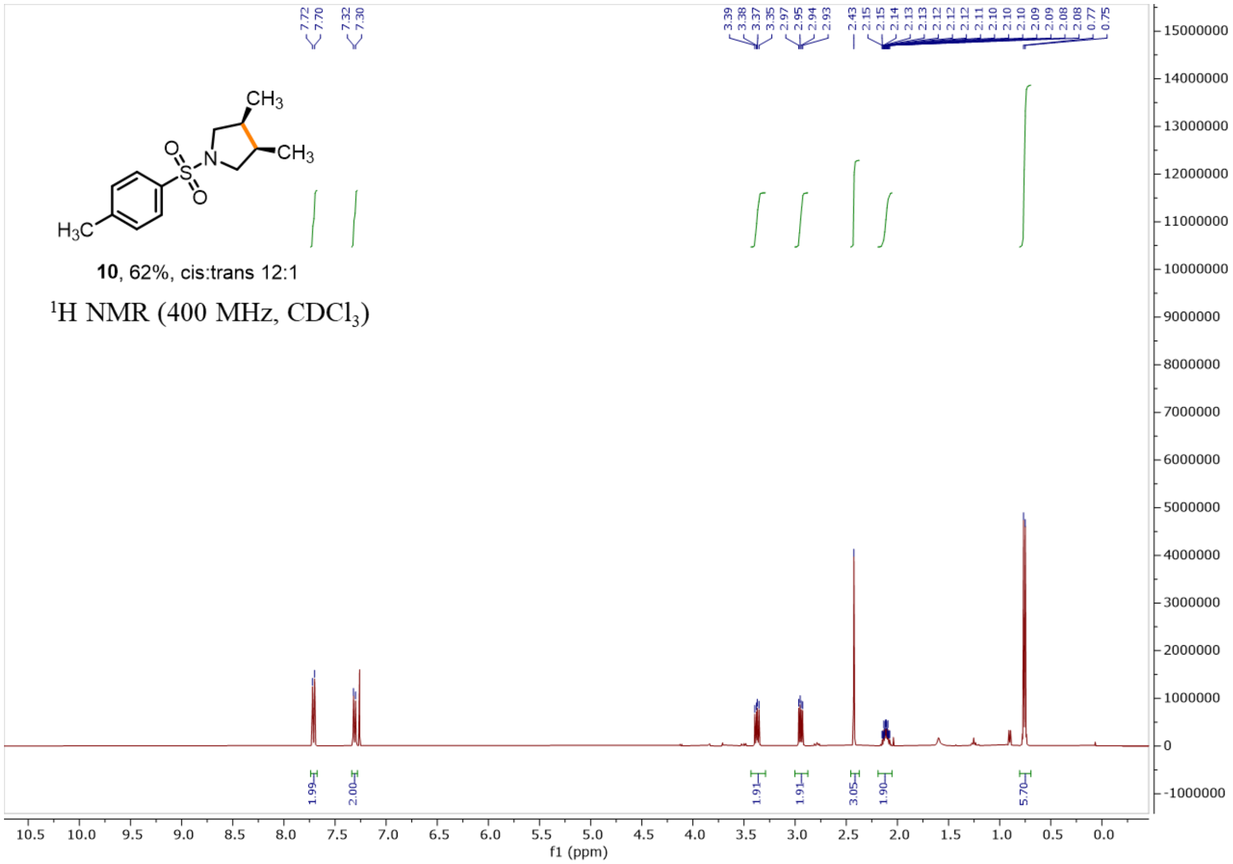
10, 62%, cis:trans 12:1
$^{1}$H NMR (400 MHz, $CDCl_3$)
f1 (ppm)

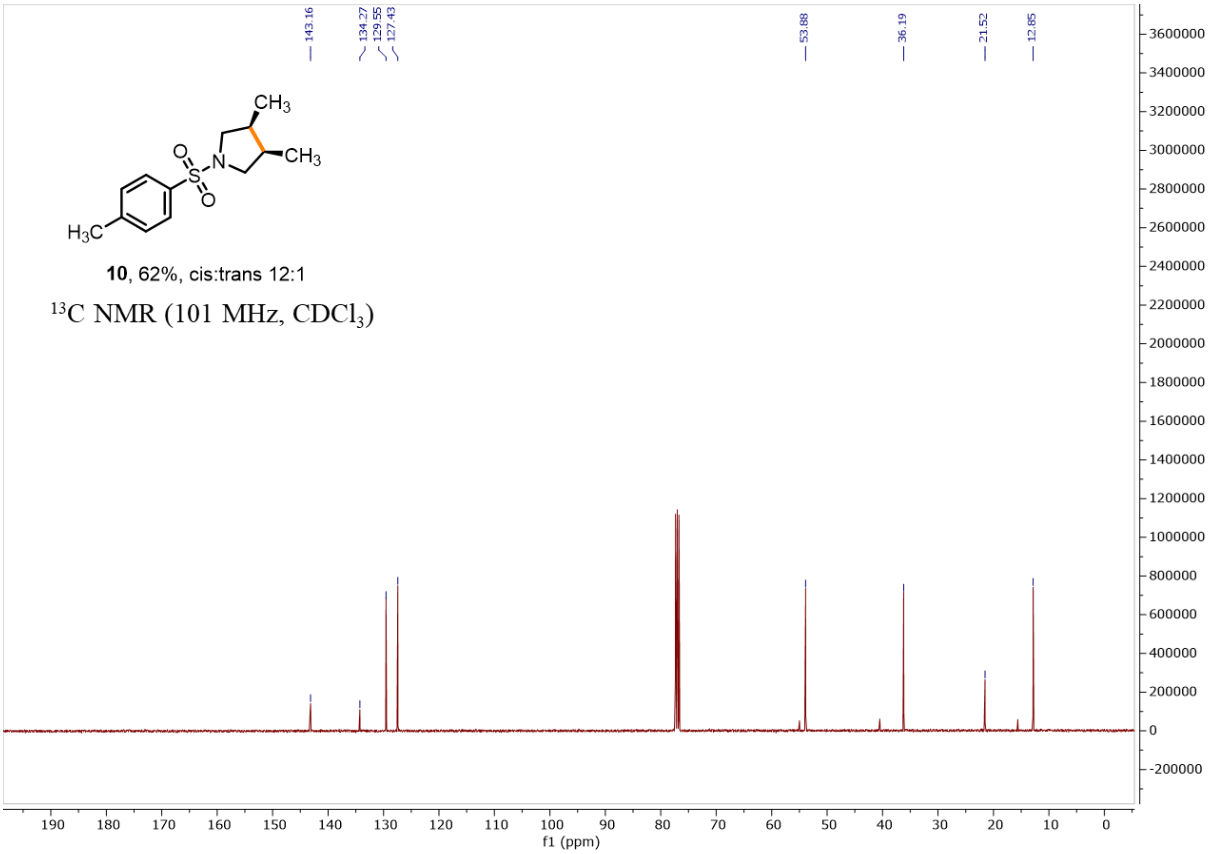
10, 62%, cis:trans 12:1
$^{13}$C NMR (101 MHz, $CDCl_3$)
143.16
134.27
129.55
127.43
53.88
36.19
21.52
12.85
f1 (ppm)

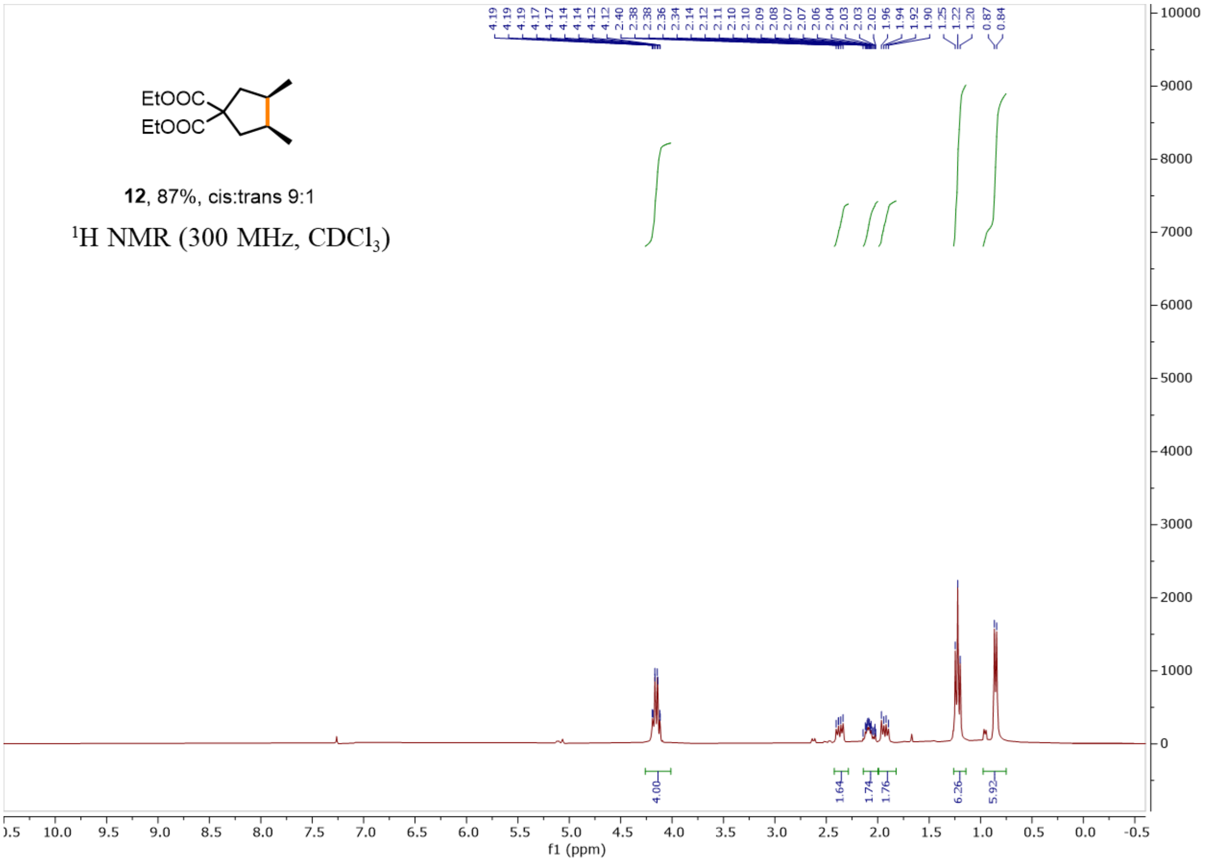
EtOOC
EtOOC
12, 87%, cis:trans 9:1
1H NMR (300 MHz, CDCl3)
4.19 4.19 4.19 4.17 4.17 4.14 4.14 4.12 4.12 2.40 2.38 2.38 2.36 2.34 2.14 2.12 2.11 2.10 2.10 2.09 2.08 2.07 2.07 2.06 2.04 2.03 2.03 2.02 1.96 1.94 1.92 1.90 1.25 1.22 1.20 0.87 0.84
4.00 1.64 1.74 1.76 6.26 5.92
f1 (ppm)

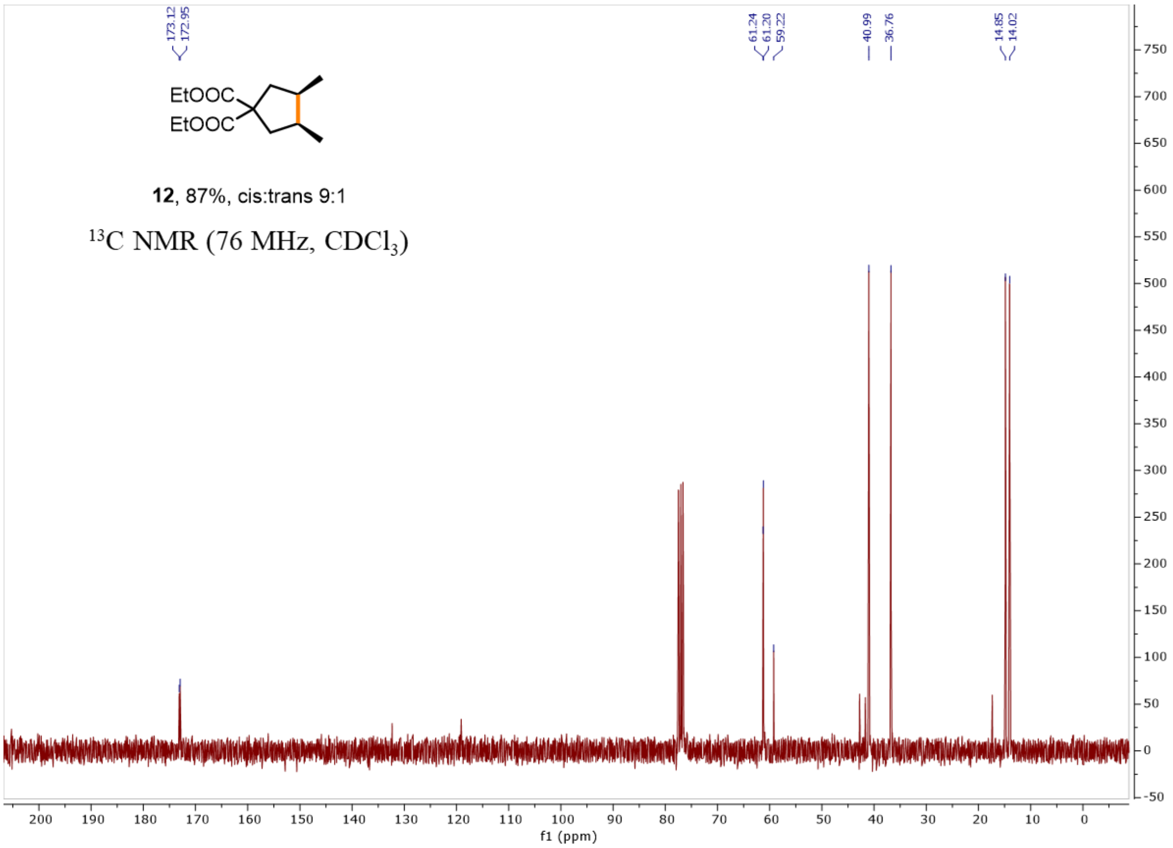
173.12 172.95 61.24 61.20 59.22 40.99 36.76 14.85 14.02
EtOOC
EtOOC
12, 87%, cis:trans 9:1
13C NMR (76 MHz, CDCl3)
f1 (ppm)

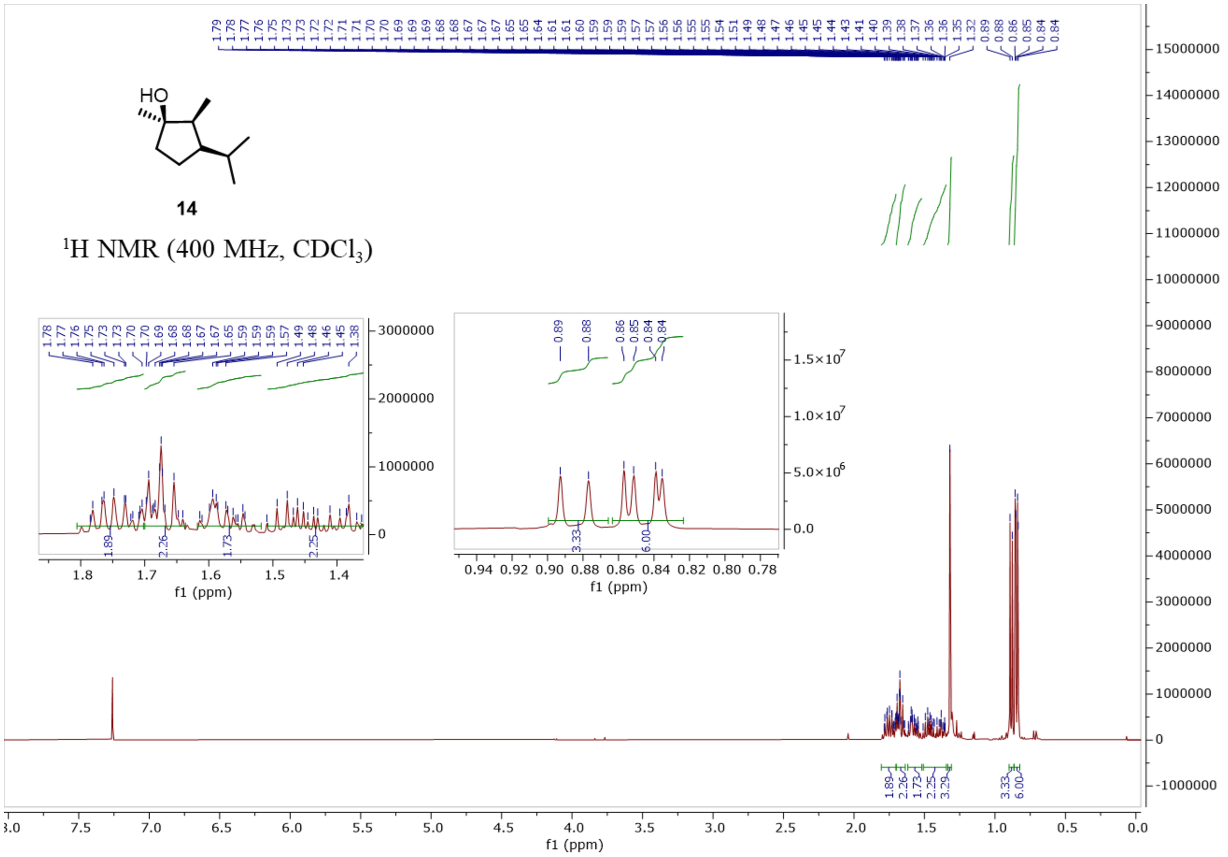

14
$^{1}H$ NMR (400 MHz, $CDCl_3$)
f1 (ppm)


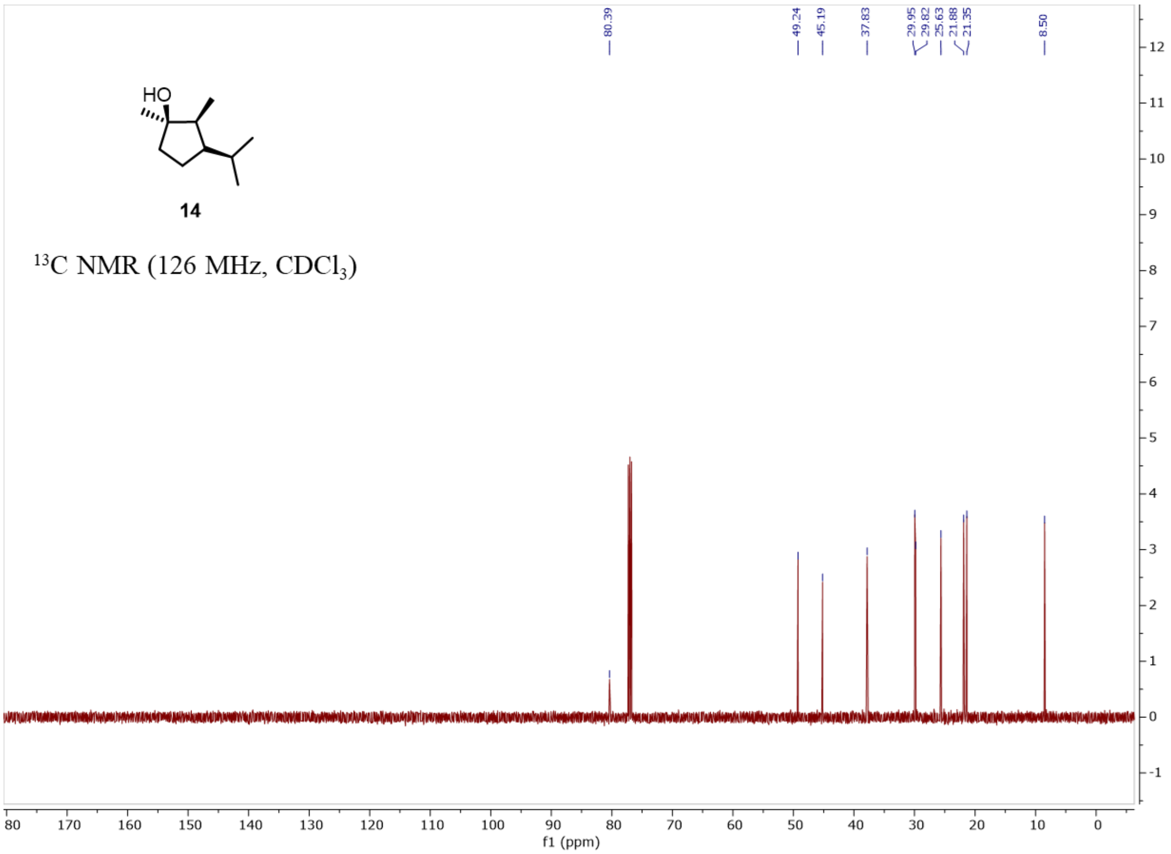

14
$^{13}C$ NMR (126 MHz, $CDCl_3$)
80.39
49.24
45.19
37.83
29.95
29.82
25.63
21.88
21.35
8.50
f1 (ppm)

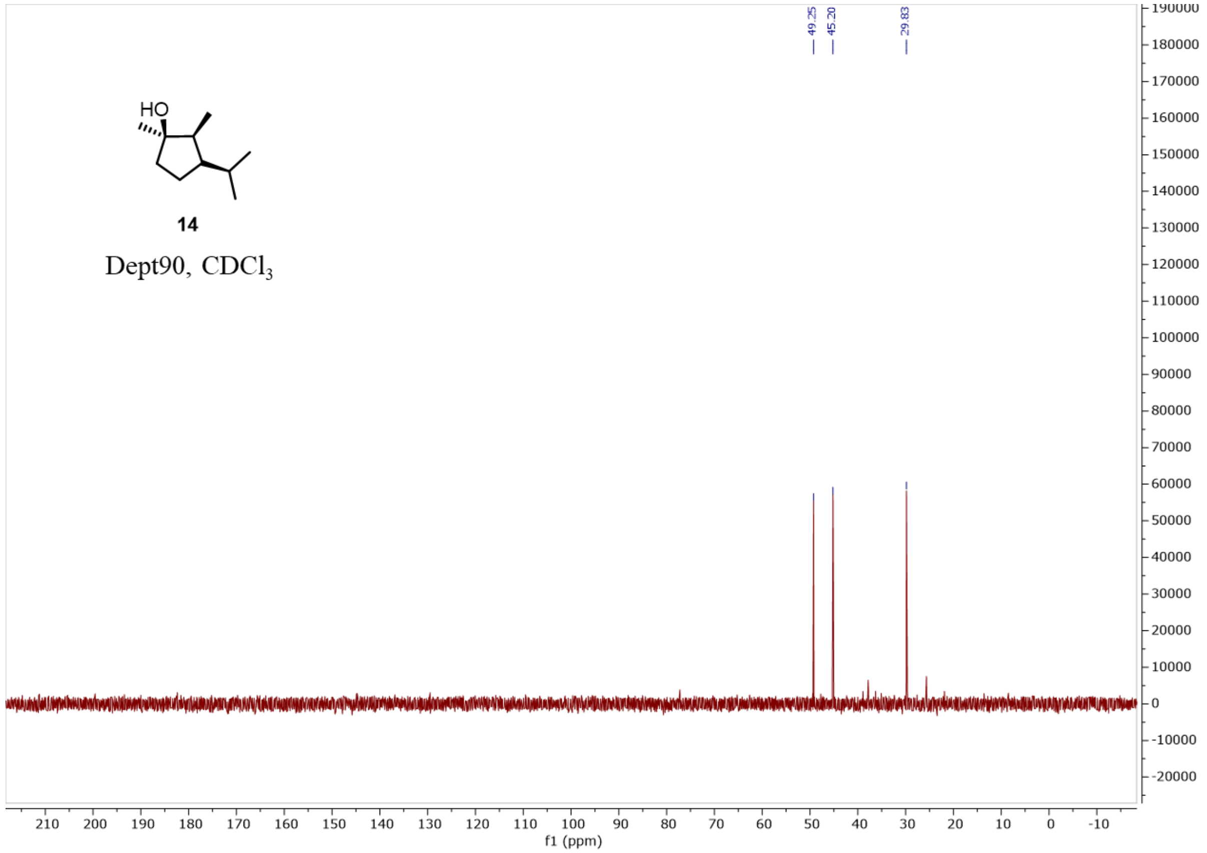
14
Dept90, $CDCl_3$
49.25
45.20
29.83
f1 (ppm)

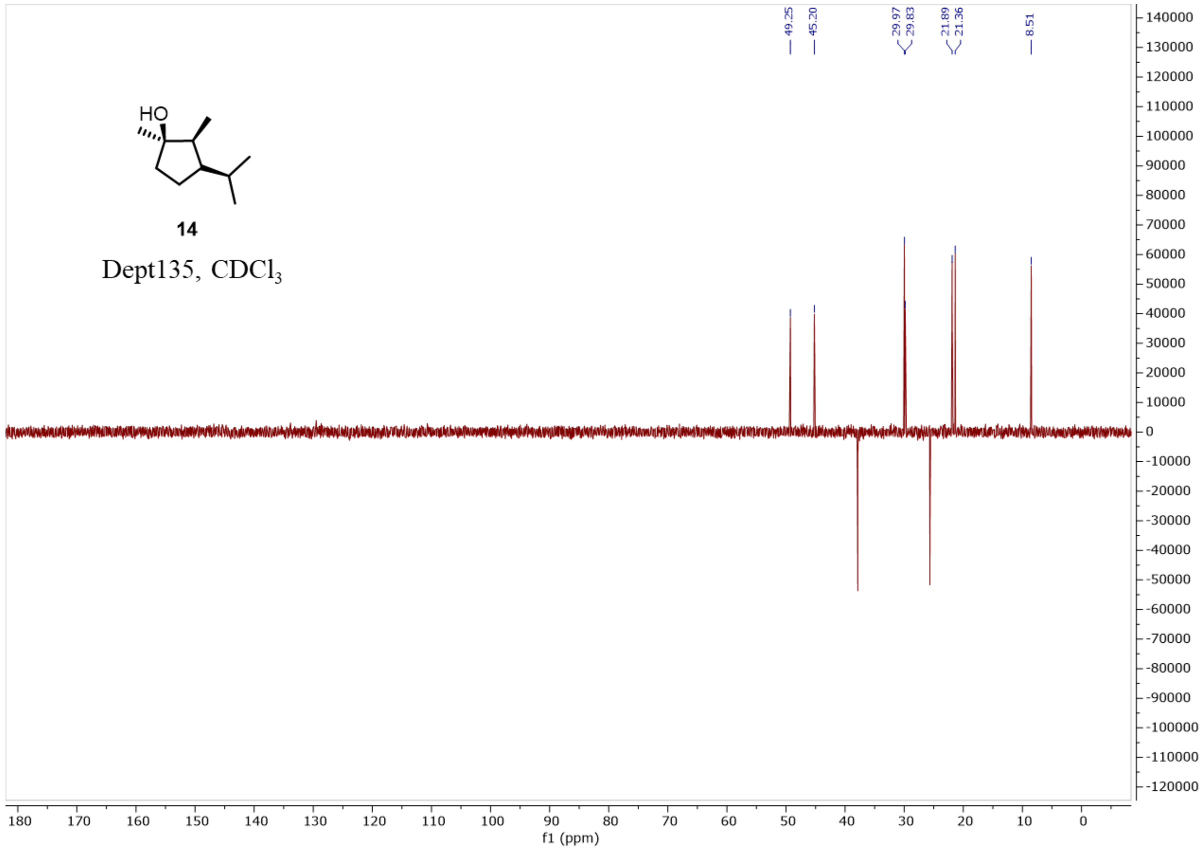
14
Dept135, $CDCl_3$
49.25
45.20
29.97
29.83
21.89
21.36
8.51
f1 (ppm)

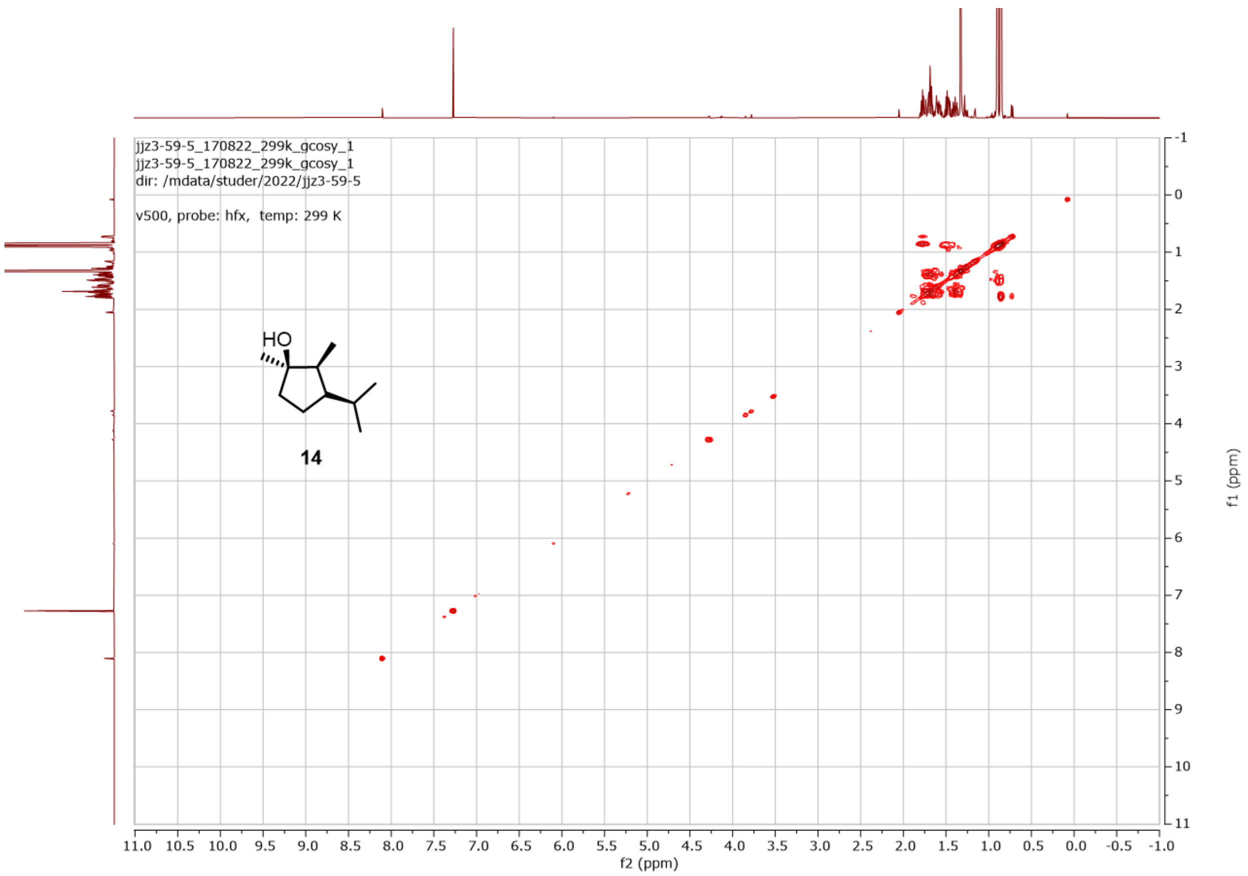
jjz3-59-5_170822_299k_gcosy_1
jjz3-59-5_170822_299k_gcosy_1
dir: /mdata/studer/2022/jjz3-59-5
v500, probe: hfx, temp: 299 K
HO
f2 (ppm)
f1 (ppm)

**14**

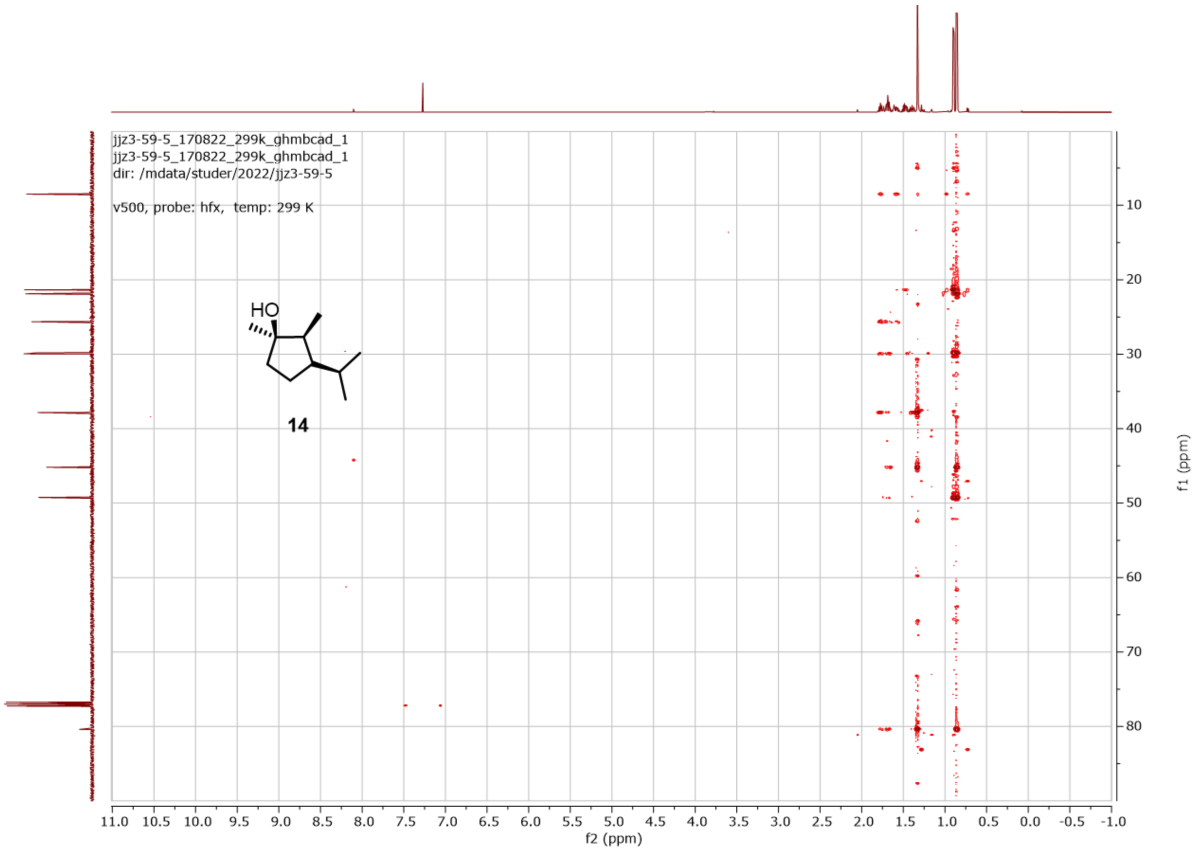
jjz3-59-5_170822_299k_ghmbcad_1
jjz3-59-5_170822_299k_ghmbcad_1
dir: /mdata/studer/2022/jjz3-59-5
v500, probe: hfx, temp: 299 K
HO
f2 (ppm)
f1 (ppm)

**14**

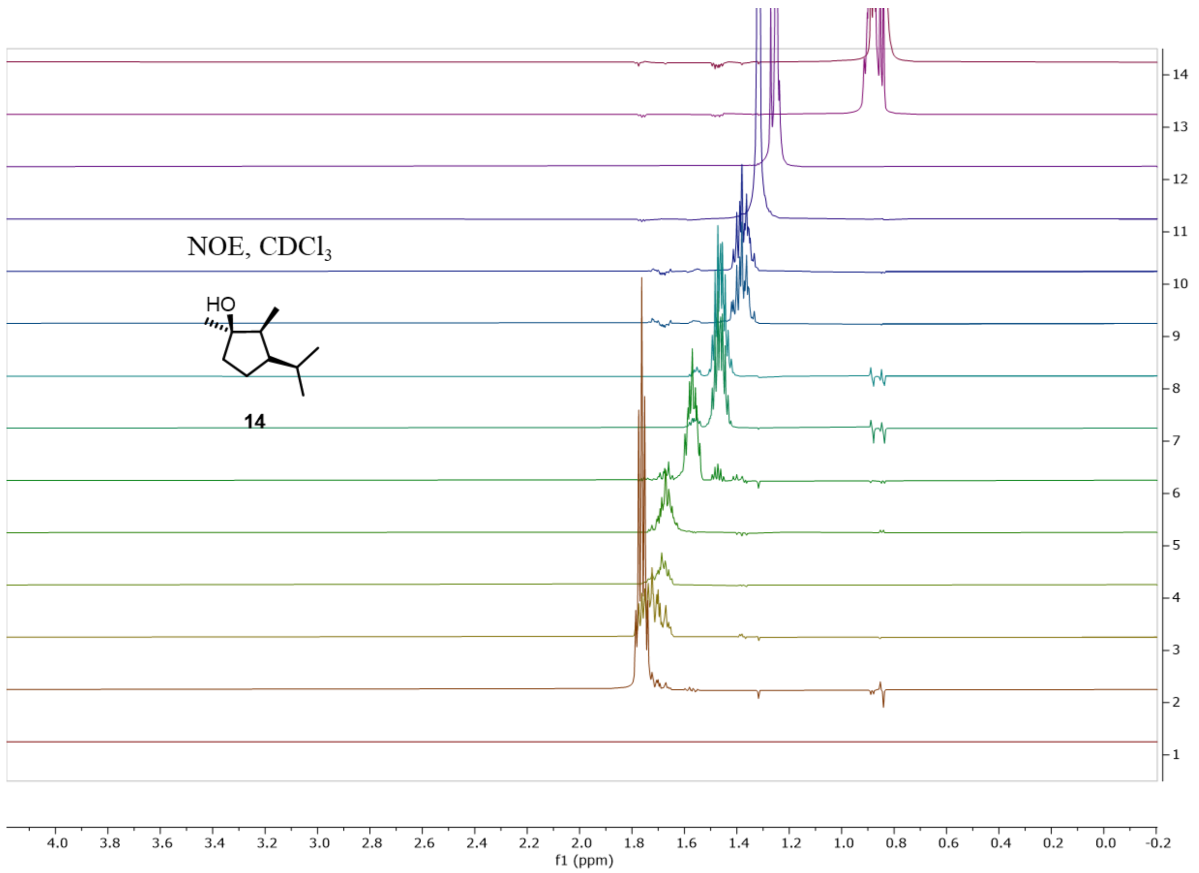
NOE, CDCl3
HO
14
14
13
12
11
10
9
8
7
6
5
4
3
2
1
4.0
3.8
3.6
3.4
3.2
3.0
2.8
2.6
2.4
2.2
2.0
1.8
1.6
1.4
1.2
1.0
0.8
0.6
0.4
0.2
0.0
-0.2
f1 (ppm)

## 14. References


1 Zhang, G. *et al.* Cobalt-Catalyzed α-Alkylation of Ketones with Primary Alcohols. *Org. Lett.* **19**, 1080-1083 (2017).

2 Cook, X. A. F. *et al.* Base-Activated Latent Heteroaromatic Sulfinates as Nucleophilic Coupling Partners in Palladium-Catalyzed Cross-Coupling Reactions. *Angew. Chem. Int. Ed.* **60**, 22461-22468 (2021).

3 Maji, A., Gupta, S., Maji, M. & Kundu, S. Well-Defined Phosphine-Free Manganese(II)-Complex-Catalyzed Synthesis of Quinolines, Pyrroles, and Pyridines. *J. Org. Chem.* **87**, 8351-8367 (2022).

4 Nagashima, Y., Takita, R., Yoshida, K., Hirano, K. & Uchiyama, M. Design, Generation, and Synthetic Application of Borylzincate: Borylation of Aryl Halides and Borylzincation of Benzynes/Terminal Alkyne. *J. Am. Chem. Soc.* **135**, 18730-18733 (2013).

5 Edelstein, E. K., Grote, A. C., Palkowitz, M. D. & Morken, J. P. A Protocol for Direct Stereospecific Amination of Primary, Secondary, and Tertiary Alkylboronic Esters. *Synlett* **29**, 1749-1752 (2018).

6 Yan, L. & Morken, J. P. Site-Selective Mono-Oxidation of 1,2-Bis(boronates). *Org. Lett.* **21**, 3760-3763 (2019).

7 Yamasaki, R. *et al.* N-Ethynylation of Anilides Decreases the Double-Bond Character of Amide Bond while Retaining trans-Conformation and Planarity. *Chem. Eur. J.* **25**, 10118-10122 (2019).

8 Pickford, H. D. *et al.* Twofold Radical-Based Synthesis of N,C-Difunctionalized Bicyclo[1.1.1]pentanes. *J. Am. Chem. Soc.* **143**, 9729-9736 (2021).

9 Kathe, P. M., Caciuleanu, A., Berkefeld, A. & Fleischer, I. Tandem Olefin Isomerization/Cyclization Catalyzed by Complex Nickel Hydride and Brønsted Acid. *J. Org. Chem.* **85**, 15183-15196 (2020).

10 Xie, Y. *et al.* Ligand-Promoted Iron(III)-Catalyzed Hydrofluorination of Alkenes. *Angew. Chem. Int. Ed.* **58**, 7097-7101 (2019).

11 Santra, S. & Guin, J. Enhanced Reactivity of Aerobic Diimide Olefin Hydrogenation with Arylboronic Compounds: An Efficient One-Pot Reduction/Oxidation Protocol. *Eur. J. Org. Chem.* **2015**, 7253-7257 (2015).

12 Fujihara, T., Semba, K., Terao, J. & Tsuji, Y. Copper-Catalyzed Hydrosilylation with a Bowl-Shaped Phosphane Ligand: Preferential Reduction of a Bulky Ketone in the Presence of an Aldehyde. *Angew. Chem. Int. Ed.* **49**, 1472-1476 (2010).

13 Paul, C. E. *et al.* Expanding the Regioselective Enzymatic Repertoire: Oxidative Mono-cleavage of Dialkenes Catalyzed by Trametes Hirsuta. *Chem. Commun.* **48**, 3303-3305 (2012).

14 Arndt, T., Raina, A. & Breugst, M. Iodine-Catalyzed Claisen-Rearrangements of Allyl Aryl Ethers and Subsequent Iodocyclizations. *Chem. Asian J.* **2023**, e202201279 (2023).

15 Marcin, L. R., Denhart, D. J. & Mattson, R. J. Catalytic Asymmetric Diazoacetate Cyclopropanation of 1-Tosyl-3-vinylindoles. A Route to Conformationally Restricted Homotryptamines. *Org. Lett.* **7**, 2651-2654 (2005).

16 Liu, Y. *et al.* Mn(III)-Mediated Regioselective 6-endo-trig Radical Cyclization of o-Vinylaryl Isocyanides to Access 2-Functionalized Quinolines. *Adv. Synth. Catal.* **362**, 688-694 (2020).

17 Liu, J. *et al.* Visible-light-Induced Triple Catalysis for A Ring-opening Cyanation of Cyclopropyl Ketones. *Chem. Commun.* **56**, 11508-11511 (2020).

18 Linford-Wood, T. G., Coles, N. T. & Webster, R. L. Room Temperature Iron Catalyzed Transfer Hydrogenation Using n-Butanol and Poly(methylhydrosiloxane). *Green Chem.* **23**, 2703-2709 (2021).

19 Motsch, B. J. & Wengryniuk, S. E. Site-Selective Synthesis of N-Benzyl 2,4,6-Collidinium Salts by Electrooxidative C–H Functionalization. *Org. Lett.* **24**, 6060-6065 (2022).

20 Ranolia, D. *et al.* Triazolium Salts as Lewis Acid Catalysts. *Org. Lett.* **24**, 3915-3919 (2022).

21 Kawasaki, T., Ishida, N. & Murakami, M. Dehydrogenative Coupling of Benzylic and Aldehydic C–H Bonds. *J. Am. Chem. Soc.* **142**, 3366-3370 (2020).

22 Jin, L. *et al.* Unsymmetrical CNN-Palladacycles with Geometry-Constrained Iminopyridyl Ligands: An Efficient Precatalyst in Suzuki Coupling for Accessing 1,1-Diarylalkanes from Secondary Benzylic Bromides. *Org. Chem. Front.* **5**, 2484-2491 (2018).

23 Journal of the American Chemical SocietyKalvet, I., Sperger, T., Scattolin, T., Magnin, G. & Schoenebeck, F. Palladium(I) Dimer Enabled Extremely Rapid and Chemoselective Alkylation of Aryl Bromides over Triflates and Chlorides in Air. *Angew. Chem. Int. Ed.* **56**, 7078-7082 (2017).

24 Gladfelder, J. J. *et al.* Enantioselective Alkylation of 2-Alkylpyridines Controlled by Organolithium Aggregation. *J. Am. Chem. Soc.* **141**, 15024-15028 (2019).

25 Kim, K. D. & Lee, J. H. Visible-Light Photocatalyzed Deoxygenation of N-Heterocyclic N-Oxides. *Org. Lett.* **20**, 7712-7716 (2018).

26 Felpin, F.-X. & Fouquet, E. A Useful, Reliable and Safer Protocol for Hydrogenation and the Hydrogenolysis of O-Benzyl Groups: The In Situ Preparation of an Active $Pd^0$/C Catalyst with Well-Defined Properties. *Chem. Eur. J.* **16**, 12440-12445 (2010).

27 Cheng, H.-C., Hou, W.-J., Li, Z.-W., Liu, M.-Y. & Guan, B.-T. The Copper-Catalyzed Aerobic Oxidative Amidation of Tertiary Amines. *Chem. Commun.* **51**, 17596-17599 (2015).

28 Cao, P. *et al.* Hydroamination of Unactivated Alkenes Catalyzed by Novel Platinum(II) N-Heterocyclic Carbene Complexes. *Organometallics* **31**, 921-929 (2012).

29 Laha, J. K., Sharma, S. & Dayal, N. Palladium-Catalyzed Regio- and Chemo-selective Reactions of 2-Bromobenzyl Bromides: Expanding the Scope for the Synthesis of Biaryls Fused to a Seven-Membered Sultam. *Eur. J. Org. Chem.* **2015**, 7885-7891 (2015).

30 Ghaffari, B., Mendes-Burak, J., Chan, K. W. & Copéret, C. Silica-Supported $Mn^{II}$ Sites as Efficient Catalysts for Carbonyl Hydroboration, Hydrosilylation, and Transesterification. *Chem. Eur. J.* **25**, 13869-13873 (2019).

31 Song, C. K., Eckstein, B. J., Tam, T. L. D., Trahey, L. & Marks, T. J. Conjugated Polymer Energy Level Shifts in Lithium-Ion Battery Electrolytes. *ACS Appl. Mater. Interfaces* **6**, 19347-19354 (2014).

32 Li, X.-G. *et al.* Hydrogenation of Esters by Manganese Catalysts. *Adv. Synth. Catal.* **364**, 744-749 (2022).

33 Decorzant, R., Vial, C., Näf, F. & Whitesides, G. M. A Short Synthesis of Ambrox® from Sclareol. *Tetrahedron* **43**, 1871-1879 (1987).

34 Shibuya, M., Orihashi, T., Li, Y. & Yamamoto, Y. N-Hydroxyphthalimide-Catalyzed Chemoselective Intermolecular Benzylic C–H Amination of Unprotected Arylalkanols. *Chem. Commun.* **57**, 8742-8745 (2021).

35 De Smet, G. *et al.* Selective Nickel-Catalyzed Hydrodeacetoxylation of Aryl Acetates. *Angew. Chem. Int. Ed.* **61**, e202201751 (2022).

36 Kobayashi, J. *et al.* A Microfluidic Device for Conducting Gas-Liquid-Solid Hydrogenation Reactions. *Science* **304**, 1305-1308 (2004).

37 Newman, S. G. *et al.* Rapid Wolff–Kishner Reductions in A Silicon Carbide Microreactor. *Green Chem.* **16**, 176-180 (2014).

38 Li, H. *et al.* Ni-Catalyzed Electrochemical Decarboxylative C–C Couplings in Batch and Continuous Flow. *Org. Lett.* **20**, 1338-1341 (2018).

39 Franz, J. A. & Camaioni, D. M. Radical Pathways of Coal Dissolution in Hydrogen Donor Media. 2. .Beta. Scission and 1,2 Aryl Migration Reactions of Radicals Derived from Methylindans and Tetralin at 327-627.Degree.C. *J. Org. Chem.* **45**, 5247-5255 (1980).

40 Yeh, P.-P. *et al.* N- to C-Sulfonyl Photoisomerisation of Dihydropyridinones: A Synthetic and Mechanistic study. *Org. Biomol. Chem.* **15**, 8914-8922 (2017).

41 Chakrabarti, A., Biswas, G. K. & Chakraborty, D. P. Photo-Fries Rearrangements in N-Sulphonyl Carbazoles. *Tetrahedron* **45**, 5059-5064 (1989).

42 Potter, G. A., Barrie, S. E., Jarman, M. & Rowlands, M. G. Novel Steroidal Inhibitors of Human Cytochrome P45017.alpha.-Hydroxylase-C17,20-lyase): Potential Agents for the Treatment of Prostatic Cancer. *J. Med. Chem.* **38**, 2463-2471 (1995).

43 Fuse, H., Kojima, M., Mitsunuma, H. & Kanai, M. Acceptorless Dehydrogenation of Hydrocarbons by Noble-Metal-Free Hybrid Catalyst System. *Org. Lett.* **20**, 2042-2045 (2018).

44 Kuwano, R. & Shige, T. Palladium-Catalyzed Formal [4+2] Cycloaddtion of o-Xylylenes with Olefins. *J. Am. Chem. Soc.* **129**, 3802-3803 (2007).

45 He, Y., Tang, J., Luo, M. & Zeng, X. Regioselective and Chemoselective Reduction of Naphthols Using Hydrosilane in Methanol: Synthesis of the 5,6,7,8-Tetrahydronaphthol Core. *Org. Lett.* **20**, 4159-4163 (2018).

46 Bertuletti, S. *et al.* Insights into the Substrate Promiscuity of Novel Hydroxysteroid Dehydrogenases. *Adv. Synth. Catal.* **362**, 2474-2485 (2020).

47 Ito, A. & Honda, E. Preparation of 2,6-Tetralindicarboxylic Acid Dialkyl Esters by Hydrogenation of 2,6-Naphthalenedicarboxylic Acid Dialkyl Esters in the Presence of Noble Metal Catalysts. JP2015059117A (2015).

48 Kurteva, V. B., Santos, A. G. & Afonso, C. A. M. Microwave Accelerated Facile Synthesis of Fused Polynuclear Hydrocarbons in Dry Media by Intramolecular Friedel–Crafts Alkylation. *Org. Biomol. Chem.* **2**, 514-523 (2004).

49 M¨ller, P. & Rey, M. Synthesis and Properties of 1, 1-Dihalogenocycloprop[b]Anthracenes. *Helv. Chim. Acta* **65**, 1157-1166 (1982).

50 Chciuk, T. V., Li, A. M., Vazquez-Lopez, A., Anderson, W. R., Jr. & Flowers, R. A., Secondary Amides as Hydrogen Atom Transfer Promoters for Reactions of Samarium Diiodide. *Org. Lett.* **19**, 290-293 (2017).

51 Findlay, N. J. *et al.* Reductions of Challenging Organic Substrates by a Nickel Complex of a Noninnocent Crown Carbene Ligand. *J. Am. Chem. Soc.* **132**, 15462-15464 (2010).

52 Shen, N. *et al.* Photocatalytic Cross-Couplings of Aryl Halides Enabled by o-Phosphinophenolate and o-Phosphinothiophenolate. *ACS Catal.* **12**, 2788-2795 (2022).

53 Lee, D. S. *et al.* Organophotocatalytic Arene Functionalization: C–C and C–B Bond Formation. *Org. Lett.* **21**, 9950-9953 (2019).

54 Li, Z.-J. *et al.* Visible-light Induced Disproportionation of Pyrrole Derivatives for Photocatalyst-free Aryl Halides Reduction. *Green Chem.* **22**, 1911-1918 (2020).

55 Widenhoefer, R. A., Krzyzanowska, B. & Webb-Wood, G. Palladium-Catalyzed Intramolecular Hydrosilylation of Alkenylsilanes: Selective Formation of Six-Membered Silicon Heterocycles. *Organometallics* **17**, 5124-5127 (1998).

56 Kim, R., Kwon, K. & Lee, H.-Y. A Free Radical Cyclization Catalyzed by Ruthenium Hydride Species. *Chem. Asian J.* **16**, 3909-3913 (2021).

57 Curran, D. P. & Shen, W. Radical Translocation Reactions of Vinyl Radicals: Substituent Effects on 1,5-Hydrogen-transfer Reactions. *J. Am. Chem. Soc.* **115**, 6051-6059 (1993).

58 He, R.-D. *et al.* Reductive Alkylation of Alkenyl Acetates with Alkyl Bromides by Nickel Catalysis. *Angew. Chem. Int. Ed.* **61**, e202114556 (2022).

59 Wu, Z. *et al.* Stereospecific Synthesis of Alkenes by Eliminative Cross-Coupling of Enantioenriched sp3-Hybridized Carbenoids. *Angew. Chem. Int. Ed.* **55**, 12285-12289 (2016).

60 Kuhn, H. J., Braslavsky, S. E. & Schmidt, R. Chemical Actinometry (IUPAC Technical Report). **76**, 2105-2146 (2004).

61 Cismesia, M. A. & Yoon, T. P. Characterizing Chain Processes in Visible Light Photoredox Catalysis. *Chem. Sci.* **6**, 5426-5434 (2015).

62 Wegner, E. E. & Adamson, A. W. Photochemistry of Complex Ions. III. Absolute Quantum Yields for the Photolysis of Some Aqueous Chromium(III) Complexes. Chemical Actinometry in the Long Wavelength Visible Region. *J. Am. Chem. Soc.* **88**, 394-404 (1966).

63 Hatchard, C. G., & Parker, C. A. A New Sensitive Chemical Actinometer - II. Potassium Ferrioxalate as a Standard Chemical Actinometer. *Proc. R. Soc. Lond. A* **235**, 518-536 (1956).

64 Perdew, J. P., Ernzerhof, M. & Burke, K. Rationale for Mixing Exact Exchange with Density Functional Approximations. *J. Chem. Phys.* **105**, 9982-9985 (1996).

65 Adamo, C. & Barone, V. Toward Reliable Density Functional Methods without Adjustable Parameters: The PBE0 model. *J. Chem. Phys.* **110**, 6158-6170 (1999).

66 Grimme, S., Antony, J., Ehrlich, S. & Krieg, H. A Consistent and Accurate ab Initio Parametrization of Density Functional Dispersion Correction (DFT-D) for the 94 Elements H-Pu. *J. Chem. Phys.* **132**, 154104 (2010).

67 Grimme, S., Ehrlich, S. & Goerigk, L. Effect of the Damping Function in Dispersion Corrected Density Functional Theory. *J. Comput. Chem.* **32**, 1456-1465 (2011).

68 Weigend, F. & Ahlrichs, R. Balanced Basis Sets of Split Valence, Triple Zeta Valence and Quadruple Zeta Valence Quality for H to Rn: Design and Assessment of Accuracy. *Phys. Chem. Chem. Phys.* **7**, 3297-3305 (2005).

69 Grimme, S. Supramolecular Binding Thermodynamics by Dispersion-Corrected Density Functional Theory. *Chem. Eur. J.* **18**, 9955-9964 (2012).

70 Goerigk, L. & Grimme, S. Efficient and Accurate Double-Hybrid-Meta-GGA Density Functionals—Evaluation with the Extended GMTKN30 Database for General Main Group Thermochemistry, Kinetics, and Noncovalent Interactions. *J. Chem. Theory Comput.* **7**, 291-309 (2011).

71 Klamt, A. Conductor-like Screening Model for Real Solvents: A New Approach to the Quantitative Calculation of Solvation Phenomena. *J. Phys. Chem.* **99**, 2224-2235 (1995).

72 Klamt, A., Jonas, V., Bürger, T. & Lohrenz, J. C. W. Refinement and Parametrization of COSMO-RS. *The J. Phys. Chem. A* **102**, 5074-5085 (1998).

73 TURBOMOLE V7.5.1 (2021), A Development of University of Karlsruhe and Forschungszentrum Karlsruhe GmbH, 1989-2007, TURBOMOLE GmbH, since 2007; available from *http://www.turbomole.com*.

74 Neese, F. The ORCA program system, Wiley Interdisciplinary Reviews: Computational Molecular *Science*, **2**, 73-78 (2012)
